# Using biomarkers to predict TB treatment duration (Predict TB): a prospective, randomized, noninferiority, treatment shortening clinical trial

**DOI:** 10.12688/gatesopenres.12750.1

**Published:** 2017-11-06

**Authors:** Ray Y. Chen, Laura E. Via, Lori E. Dodd, Gerhard Walzl, Stephanus T. Malherbe, André G. Loxton, Rodney Dawson, Robert J. Wilkinson, Friedrich Thienemann, Michele Tameris, Mark Hatherill, Andreas H. Diacon, Xin Liu, Jin Xing, Xiaowei Jin, Zhenya Ma, Shouguo Pan, Guolong Zhang, Qian Gao, Qi Jiang, Hong Zhu, Lili Liang, Hongfei Duan, Taeksun Song, David Alland, Michael Tartakovsky, Alex Rosenthal, Christopher Whalen, Michael Duvenhage, Ying Cai, Lisa C. Goldfeder, Kriti Arora, Bronwyn Smith, Jill Winter, Clifton E. Barry III, Predict TB Study Group

**Affiliations:** 1 Tuberculosis Research Section, Laboratory of Clinical Immunology and Microbiology, Division of Intramural Research, National Institute of Allergy and Infectious Diseases (NIAID), National Institutes of Health (NIH), Bethesda, MD, USA; 2 Wellcome Centre for Infectious Diseases Research in Africa,Institute of Infectious Disease and Molecular Medicine, University of Cape Town (UCT), Cape Town, South Africa; 3 Biostatistics Research Branch, Division of Clinical Research, National Institute of Allergy and Infectious Diseases (NIAID), National Institutes of Health (NIH), Bethesda, MD, USA; 4 South Africa Department of Science and Technology - National Research Foundation Centre of Excellence for Biomedical Tuberculosis Research, South African Medical Research Council Centre for Tuberculosis Research, Division of Molecular Biology and Human Genetics, Faculty of Medicine and Health Sciences, Stellenbosch University, Cape Town, South Africa; 5 Division of Pulmonology, Department of Medicine, University Of Cape Town Lung Institute, University of Cape Town (UCT), Cape Town, South Africa; 6 Francis Crick Institute, London, NW1 2AT, UK; 7 Department of Medicine, Imperial College London, London, W2 1PG, UK; 8 Department of Internal Medicine, University Hospital Zurich, Zurich, Switzerland; 9 South African Tuberculosis Vaccine Initiative, University of Cape Town (UCT), Cape Town, South Africa; 10 TASK Applied Science and Stellenbosch University, Cape Town, South Africa; 11 Henan Provincial Chest Hospital, Zhengzhou, Henan, China; 12 Henan Provincial Institute of Tuberculosis and Prevention, Henan Center for Disease Control, Zhengzhou, Henan, China; 13 Xinmi City Institute of Tuberculosis Prevention and Control, Xinmi, Henan, China; 14 Kaifeng City Institute of Tuberculosis Prevention and Control, Kaifeng, Henan, China; 15 Zhongmu County Health and Epidemic Prevention Station, Zhongmu, Henan, China; 16 Fudan University, Shanghai, China; 17 Sino-US Tuberculosis Collaborative Research Program, Zhengzhou, Henan, China; 18 Beijing Chest Hospital, Beijing, China; 19 Institute of Infectious Disease and Molecular Medicine, University of Cape Town (UCT), Cape Town, South Africa; 20 Division of Infectious Diseases, Department of Medicine, Rutgers New Jersey Medical School, Newark, NJ, USA; 21 Office of Cyber Infrastructure and Computational Biology, National Institute of Allergy and Infectious Diseases (NIAID), National Institutes of Health (NIH), Bethesda, MD, USA; 22 Catalysis Foundation for Health, Emeryville, CA, USA

**Keywords:** pulmonary tuberculosis, drug sensitive, PET/CT, GeneXpert, cycle threshold, treatment shortening, biomarkers, MERM

## Abstract

**Background**: By the early 1980s, tuberculosis treatment was shortened from 24 to 6 months, maintaining relapse rates of 1-2%. Subsequent trials attempting shorter durations have failed, with 4-month arms consistently having relapse rates of 15-20%. One trial shortened treatment only among those without baseline cavity on chest x-ray and whose month 2 sputum culture converted to negative. The 4-month arm relapse rate decreased to 7% but was still significantly worse than the 6-month arm (1.6%, P<0.01).  We hypothesize that PET/CT characteristics at baseline, PET/CT changes at one month, and markers of residual bacterial load will identify patients with tuberculosis who can be cured with 4 months (16 weeks) of standard treatment.

**Methods**: This is a prospective, multicenter, randomized, phase 2b, noninferiority clinical trial of pulmonary tuberculosis participants. Those eligible start standard of care treatment. PET/CT scans are done at weeks 0, 4, and 16 or 24. Participants who do not meet early treatment completion criteria (baseline radiologic severity, radiologic response at one month, and GeneXpert-detectable bacilli at four months) are placed in Arm A (24 weeks of standard therapy). Those who meet the early treatment completion criteria are randomized at week 16 to continue treatment to week 24 (Arm B) or complete treatment at week 16 (Arm C). The primary endpoint compares the treatment success rate at 18 months between Arms B and C.

**Discussion**: Multiple biomarkers have been assessed to predict TB treatment outcomes. This study uses PET/CT scans and GeneXpert (Xpert) cycle threshold to risk stratify participants. PET/CT scans are not applicable to global public health but could be used in clinical trials to stratify participants and possibly become a surrogate endpoint. If the Predict TB trial is successful, other immunological biomarkers or transcriptional signatures that correlate with treatment outcome may be identified. Trial Registration: NCT02821832

## Background

Tuberculosis (TB) is one of the top three global causes of infectious diseases and one of the top ten global causes of death, recently surpassing even HIV/AIDS ^1^. Treatment of drug sensitive TB (DS-TB) is long, typically requiring six months with good adherence to achieve cure and prevent relapse after therapy is stopped. However, treatment adherence and completion rates are not optimal, which could probably be improved by shorter and more effective treatments. Multiple studies conducted over the past 40 years have attempted to reduce the treatment duration of TB. In the 1970s, successful combination chemotherapy lasted 24 months, based on a series of studies conducted by the British Medical Research Council (BMRC). Follow-up studies in the early 1980s successfully reduced treatment duration to 6 months using pyrazinamide and rifampin, achieving relapse rates of 1–2%. Trials that reduced the duration to below 6 months experienced increasing relapse rates, roughly 12% at 4 months and up to 20% at 3 months ^2^, establishing 6 months as the accepted treatment duration for all cases of DS-TB.

Three recent randomized-controlled trials attempted to shorten treatment using a 4-month experimental arm with a fluoroquinolone substituted for one of the four standard drugs ^3^,^5^. In all three trials, the 4-month treatment arms had relapse rates of approximately 15–20%, significantly higher than the standard of care 6-month arms but similar to the BMRC 4-month treatment trials (12%, 95% CI 9-16 among 364 patients) conducted 30+ years earlier ^2^. These trials suggest that, with currently available drugs, roughly 80–85% of drug-sensitive TB patients are cured with 4 months of standard therapy but 15–20% will relapse if not treated for at least 6 months and, further, that a subset of those with less severe disease could be cured at 4 months. Identifying this subset prospectively could lead to: (1) new treatment guidelines for eligible lower risk patients; (2) criteria for selection of high-risk patients that might be candidates for Phase 2b studies with novel regimens with proposed shorter durations; and (3) quantitative estimates of the rate of change of markers associated with durable cure at specific timepoints to establish milestones for even shorter regimens. The hypothesis that patients with less severe disease can be cured earlier was tested in a separate treatment shortening trial, in which only those with less severe disease and good initial treatment response were randomized to 4 vs. 6 months of treatment ^6^. In this trial, less severe disease was defined as absence of pulmonary cavities on baseline chest x-ray, and good treatment response was defined as sputum culture conversion to negative by 2 months of treatment. Only participants who met these 2 criteria were randomized to 4 vs. 6 months of treatment with standard of care drugs. This trial was stopped early by its Data and Safety Monitoring Board (DSMB) because those in the 6-month arm had a relapse rate of 1.6% while the 4-month arm had a significantly higher relapse rate of 7% (P<0.01). Despite this trial not meeting its target, the disease stratification criteria did increase the treatment success rate of the 4-month arm from 80–85% in the prior non-stratified trials to 93% (95% CI 89%, 96%), approaching 6-month treatment relapse rates. If the stratification criteria could be further refined, a successful 4-month treatment arm may be feasible. The hypothesis of the Predict TB trial is that a combination of radiographic characteristics at baseline, the rate of change of these features at one month, and markers of residual bacterial load at 4 months will identify patients with tuberculosis who are cured within 4 months (16 weeks) of standard treatment.

## Methods

### Study design

This is a prospective, multicenter, randomized, phase 2b, noninferiority clinical trial of pulmonary DS-TB participants conducted at 4 sites in Henan Province, China (Henan Provincial Chest Hospital, Xinmi City Institute of Tuberculosis Prevention and Control, Kaifeng City Institute of Tuberculosis Prevention and Control, and Zhongmu County Health and Epidemic Prevention Station) and 5 sites in and around Cape Town, South Africa (Stellenbosch University, University of Cape Town Lung Institute, University of Cape Town South African Tuberculosis Vaccine Initiative, Khayelitsha Site B, and TASK Applied Science). Inclusion criteria are:

1) Age 18 to 65 years, with body weight from 35 kg to 90 kg
2) Not been treated for active TB within the past 3 years
3) Not yet on TB treatment
4) Xpert positive for *Mycobacterium tuberculosis*
5) Rifampin-sensitive pulmonary tuberculosis as indicated by Xpert
6) Laboratory parameters within previous 14 days before enrollment:
  a. Serum AST and ALT <3x upper limit of normal (ULN)
  b. Creatinine <2x ULN
  c. Hemoglobin >7.0 g/dL
  d. Platelet count >50 x10 cells/L
7) Able and willing to return for follow-up visits
8) Able and willing to provide informed consent to participate in the study
9) Willing to undergo an HIV test
10) Willing to have samples, including DNA, stored
11) Willing to consistently practice a highly reliable, non-hormonal method of pregnancy prevention (e.g., condoms) during treatment if participant is a premenopausal female, unless she has had a hysterectomy or bilateral tubal ligation or her male partner has had a vasectomy

Exclusion criteria are:

1) Extrapulmonary TB, including pleural TB
2) Pregnant or desiring/trying to become pregnant in the next 6 months or breastfeeding
3) HIV infected
4) Unable to take oral medications
5) Diabetes as defined by point of care HbA1c ≥6.5%, random glucose ≥200 mg/dL (or 11.1 mmol/L), fasting plasma glucose ≥126 mg/dL (or 7.0 mmol/L), or the presence of any anti-diabetic agent (including traditional medicines) as a concomitant medicine
6) Disease complications or concomitant illnesses that may compromise safety or interpretation of trial endpoints, such as known diagnosis of chronic inflammatory condition (e.g. sarcoidosis, rheumatoid arthritis, connective tissue disorder)
7) Use of immunosuppressive medications, such as TNF-alpha inhibitors or systemic or inhaled corticosteroids, within the past 2 weeks
8) Use of any investigational drug in the previous 3 months
9) Substance or alcohol abuse that in the opinion of the investigator may interfere with the participant’s adherence to study procedures
10) Any person for whom the physician feels this study is not appropriate

Eligible participants who sign the informed consent are started on standard of care treatment with fixed-dose combination tablets composed of isoniazid (H), rifampin (R), pyrazinamide (Z), and ethambutol (E) during the initial 8-week intensive phase and HR during the subsequent continuation phase, using national weight-based guidelines ( Table 1). PET/CT scans are done at baseline, week 4, and either week 16 or 24. Participants are followed through month 18 and a fourth PET/CT scan is done if recurrent TB develops during follow-up. All PET/CT scans are read by 2 readers using MIM Software (v. 6.6 or higher, MIM Software Inc, Cleveland, Ohio, USA). Scans with discrepant arm assignments by the initial 2 readers are read by a third reader. All PET/CT scan readers underwent a prequalification training process with 15 practice baseline and week 4 scans to ensure consistency among all readers in how the scans are read (see Supplementary File 5 for PET/CT reading SOP). The practice PET/CT scans were obtained from a previously conducted study at Stellenbosch University funded by the Bill and Melinda Gates Foundation ( *Mycobacterium tuberculosis* biomarkers for diagnosis and cure, OPP51919).

**Table 1.** Adult weight-based dosing guidelines for intensive phase and continuation phase TB treatment using fixed-dose combination (FDC) tablets by country. These are the dosing guidelines used in the Predict TB study and are taken from the South African national TB guidelines ^39^ and the Chinese fixed drug combination tablet package insert from the Shenyang Hongqi Pharmaceutical Company, Ltd.

	Intensive Phase (initial 8 weeks)	Continuation Phase (weeks 9–24)	
	HRZE (75/150/400/275 mg)	HR (75/150 mg)	HR (150/300 mg)
Weight	China and South Africa	South Africa	
**30–37 kg**	2 tablets daily	2 tablets daily	--
**38–54 kg**	3 tablets daily	3 tablets daily	--
**55–70 kg**	4 tablets daily	--	2 tablets daily
**>70 kg**	5 tablets daily	--	2 tablets daily
		China	
**<50 kg**		--	individual drug tablets (non-FDC)
**≥50 kg**		--	2 tablets daily

Note: H=isoniazid; R=rifampin; Z=pyrazinamide; E=ethambutol

**Figure 1.**
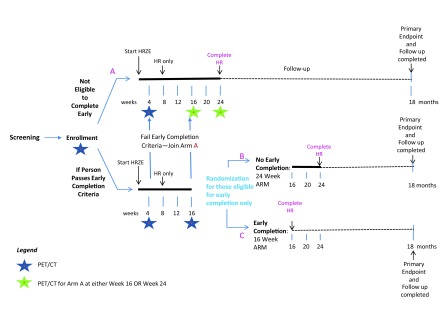
Predict TB study schematic.

Participants are stratified into treatment arms by the early treatment completion criteria, composed of baseline PET/CT criteria, the change in these measurements at week 4, and a minimum adherence dose count and Xpert cycle threshold as a marker of residual sputum bacterial load at week 16 ( Table 2). Those with more severe disease (who do not meet all early treatment completion criteria) are placed in Arm A (standard of care arm, treatment completion at week 24). Those with less severe disease (who meet all early treatment completion criteria) are randomized either to Arm B (standard treatment duration of 24 weeks) or Arm C (standard treatment shortened to 16 weeks) at week 16. The third PET/CT scan is done at week 16 for participants in Arms B or C and randomized to either week 16 or 24 for participants in Arm A. All participants are followed to week 72 for final treatment outcomes ( Figure 1), with sputum, blood, and urine samples collected per the schedule in Table 3. Study enrollment began in Cape Town in June 2017 and is expected to begin in Henan in October 2017. Enrollment is expected to take about 3 years and the study is expected to complete within 5 years.

**Table 2.** Predict TB early treatment completion criteria. These are the criteria used at baseline, week 4, and week 16 to stratify enrolled participants to Arm A or Arm B vs C. Participants must meet all criteria to be eligible for randomization to Arm B vs C at week 16. Participants who do not meet all criteria are placed into Arm A.

Category	Criteria
Radiographic criteria	Baseline PET/CT: • No total lung collapse of a single side, AND • No pleural effusion, AND • No single cavity air volume on CT scan >30 mL, AND • CT scan hard volume (-100 to +100 HU density) <200 mL, AND • PET total activity <1500 units Week 4 PET/CT: • All individual cavities decrease by >20% (unless cavity <2 mL), AND • CT scan hard volume does not increase by >10% unless the increase is <5 mL, AND • PET total activity does not increase by >30% unless the increase is <50 units
Bacterial load criterion	Week 16 Xpert cycle threshold ≥30
Adherence criterion	Minimum of 100 doses received by week 16

**Table 3.** Predict TB study timeline. Study enrollment began in Cape Town in June 2017 and is expected to begin in Henan in October 2017. Enrollment is expected to take about 3 years and the study is expected to complete within 5 years.

	Screening	D0	W1 ^V^ (D7)	W2 (D14)	W4 (D28)	W8 ^D^ (D56)	W12 (D84)	At Week 16	W16 (D112)	W20 (D140)	W24 (D168)	W36 (D252)	W48 (D336)	W60 (D420)	W72 (D504)	TB Recurrence
**Main Study** **Informed Consent** **(Plus Genetic and** **HIV in RSA)**	X							RANDOMIZE to Arm B or C								
**Medical History/** **Focused History**	X	X	X	X	X	X	X		X	X	X	X	X	phone call	X	X
**Physical Exam/** **Focused Physical** **Exam**	X	X	X	X	X	X	X		X	X	X	X	X		X	X
**Sputum Collection ^ξ^**																
Smear/Culture	XX ^H^	X	X	X	X	X	X	then	X	X	X	X	X		X	X
GeneXpert	X	(X) ^S^			X	X			X ^E^		X (arms A and B only)					X
Biomarkers		X		X	X ^g^	X ^g^			X ^g^		X ^g^		X ^g^		X ^g^	X ^g^
Saliva		X			X				X		X					X
**Blood collection**																
CBC/Chems/LFT ^G^	X							and if not assigned to Arm A,								X
Biomarkers		X	X	X	X	X			X		X		X		X	X
Pregnancy Test ^P^ (serum at screening, urine for others)	x ^h^(serum)															
HIV Testing	X															X
Plasma drug levels										X (all arms)						
**Finger Stick**		X			X				Arms B/C and randomized A		Randomized for scan in Arm A only					X
**Urine collection**																
Biomarkers		X	X	X	X	X		Review treatment completion criteria	X		X		X		X	X
Pregnancy Test ^P^ (serum at screening, urine for others)	x (can do if desired before CXR)	X			X				Arms B and C only		Arm A only					X
**FDG-PET/CT ^W^**		X			X				Arms B/C and randomized A		Randomized for scan in Arm A only					X
**Adherence** **monitoring**			X	X	X	X	X		X	Arms A and B only	Arms A and B only					

*Additional sputum may be collected if contaminated or otherwise compromised* *A subject could be called back for this additional sputa collection, if necessary.* *Sputum at screening will also be used for screening Xpert* *May not be done if within 7 days of screening* *Pregnancy testing from screening may be used for the D0 PET/CT scan if D0 is within 2 days of the screen* *These will be performed at any visit if clinically significant.* *Before any PET/CT scan or CXR is done, a pregnancy test will be done for applicable females. If the pregnancy test is positive, the PET/CT scan will not be performed.* *For those eligible for randomization to Arms B/C* *At week 8, ethambutol and pyrazinamide will be discontinued* ** ***PET/CT scan windows***: *baseline w/i 7 days after treatment initiation; W4 must be at least 4 wks after baseline scan with a -3/+7 d window; W16 and 24 scan w/i 14 d of visit; relapse ASAP, but w/i 2 wks of recurrence* ** ***Visit windows***: *Week 1–2: +/- 3 days; Week 4–24: +/- 7 d, noting that Weeks 16 and 24 should be as close as possible to actual date; Week 36–72: +/- 30 days.* *If sputum is not available for biomarkers, it will not be a protocol deviation.*

### Treatment adherence

Total treatment duration will be determined by dose counts. Participants will receive either 16 weeks of treatment (Arm C) or 24 weeks of treatment (Arms A and B). (Arm A participants may be treated longer at the discretion of the treating physician.) Participants receiving 16 weeks of treatment will receive 112 doses with a minimum total of 100 doses. Participants who do not meet this minimum dosing requirement within the week 16 visit window will not be eligible for randomization and will be moved to Arm A. Approximately 90% adherence is used, as missing more than this has been associated with an increased risk of poor outcomes ^7^. Participants receiving 24 weeks of treatment will receive 168 doses with a minimum total of 150 doses. Missed doses during the initial 8-week intensive phase will be added on to the end of the intensive phase, replacing continuation phase dosing. Arm B participants who do not achieve the minimum 150 doses within the Week 24 visit window will be allowed to complete a minimum of 150 doses even if this exceeds the visit window.

All possible and available forms of adherence monitoring are encouraged as much as local resources allow. This includes (but does not require) directly observed therapy (DOT), whether by a healthcare worker, an outreach worker, or a family member. The vast majority of participants will not receive formal DOT; these participants will be provided an electronic pill box, the Medication Event Reminder Monitor (MERM; Wisepill Technologies, South Africa), which has been shown to improve treatment adherence among TB patients in China ^8^. The MERM is a box that stores dispensed medication and also contains a cartridge that monitors box openings and sounds an alarm daily at a set time to remind participants to take their medicine. Box open/close data will be downloaded on follow-up visits.

### Treatment outcome definitions

Treatment success is defined as a participant with at least 2 consecutive negative cultures on solid medium over a span of at least 4 weeks, achieved by the end of therapy, with no subsequent confirmed positive cultures during follow-up. Participants who remain culture positive on solid medium at Week 24 in Arm A will be considered treatment failures, will be taken off study as meeting a study endpoint and referred to continue treatment per the local standard of care (SOC). Participants who convert to solid culture negative who subsequently have a single solid culture positive for *Mtb* before or at week 24 need to have a subsequent culture positive for *Mtb* to be confirmed as treatment failures. Isolated positive cultures that are not confirmed on a subsequent sputum sample are not considered failures as these may have arisen from processing error or laboratory contamination ^9^. Solid culture results will be used for the primary endpoint analysis. Liquid culture results may be used for secondary analyses.

Participants randomized to Arms B or C who are subsequently found to have a positive culture for *Mtb* on solid medium from weeks 16–24, confirmed on a subsequent culture, will be considered treatment failures. These participants will be referred to continue treatment per local SOC and will be followed observationally until the end of their treatment to determine outcomes. Participants who convert their sputum to culture negative (2 consecutive negatives over ≥4 weeks) and who subsequently become culture positive for *Mtb* again on solid medium during follow-up after week 24, confirmed by a second positive sputum culture, will be considered recurrences. Isolated positive cultures that are negative on follow-up will not be considered recurrences. Relapses will be distinguished from re-infections by DNA strain typing and only relapses will be considered a study endpoint. Participants who are treatment failures and relapses will have drug sensitivity testing done to inform subsequent treatment. Relapses on Arms B and C will have observational follow-up until the end of retreatment to determine outcomes. 

### Statistical analyses

 This is a non-inferiority study, with the primary endpoint being a comparison of the rate of treatment successes at 18 months (after treatment initiation) between Arms B and C. Final study treatment outcome data from participants who are unable to return at 18 months but do return during the 1 year following will be imputed back to the 18-month time point for the primary endpoint. The primary analysis will estimate the lower bound of a 95% confidence interval of the difference in success rates between arms B and C. If the lower bound is greater than -7%, this will be evidence that the treatment-shortening arm is not inferior to the standard duration arm. Confidence intervals will be constructed using Wald intervals, with inverse weighting according to site-estimated variances, as a stratified analysis. Additional analyses of the primary endpoint will consider a non-stratified-based confidence interval of the difference.

The sample size is determined for the comparison between Arms B and C. Because these are lower risk participants, we expect a treatment success rate of 97%. Table 4 provides power calculations for a total enrollment of 117 and 140 per group. With true success rates of 97% in both arms, study power is greater than 90% with only 117 participants per group. However, to increase power to accommodate a scenario in which the true success rate in the four-month treatment arm is slightly lower than the six-month arm, a sample size of 140 per treatment arm was selected, corresponding to 155 subjects per arm after adjusting for a 10% loss to follow-up. We expect that approximately 50% of participants will be classified as higher risk and be placed into Arm A, giving a total study sample size of 620 participants.

**Table 4.** Sample size power calculations for the Predict TB trial. Power calculations are shown for total sample sizes of 117 and 140 per group (Arms B and C) for different success rates across and between treatment arms. Because these are lower-risk participants, a 97% success rate was targeted. A sample size of 140/arm was selected to increase power in case the shortened treatment arm has a slightly lower success rate. This sample size was then increased by 10% to 155/arm to account for those lost to follow-up.

Success rate by study arm		Power for concluding NI with 7% margin, 5% type I error rate	
Arm B: 6-month tx	Arm C: 4-month tx	Sample size 117 per group	Sample size 140 per group
0.99	0.99	0.999	1
0.99	0.98	0.984	0.994
0.99	0.97	0.863	0.912
0.98	0.98	0.985	0.994
0.98	0.97	0.903	0.942
0.98	0.96	0.726	0.792
0.97	0.97	0.932	0.963
0.97	0.96	0.803	0.862
0.97	0.95	0.621	0.689
0.96	0.96	0.862	0.911
0.96	0.95	0.716	0.782
0.96	0.94	0.545	0.609
0.95	0.95	0.792	0.851
0.95	0.94	0.644	0.711
0.95	0.93	0.487	0.547

### PK-MIC substudy

Typically, bacteria are termed “resistant” to a drug if the bacteria are able to grow at concentrations above the established "breakpoint" for that drug and resistance is a well-known determinant of TB treatment outcome ^10^. Conventional drug susceptibility testing for isoniazid and rifampin will be employed during the trial to confirm that patients have susceptible isolates. The minimum inhibitory concentration (MIC) for a specific bacterial isolate is the drug concentration at which the growth of the bacteria is inhibited by approximately 95%. For each patient enrolled into the substudy, we will determine the specific isoniazid and rifampin MIC for their *Mtb* isolate. In addition, TB patients are known to have widely variable serum PK values, and these differences appear to affect treatment outcome ^11^,^13^. Because a given patient’s serum drug concentration achieved will affect the clinical interpretation of a given MIC result, we hypothesize that a model incorporating both of these parameters may predict outcomes better than either one alone. This hypothesis will be tested in a substudy among study participants believed to be at higher risk of relapse based on preliminary data, those who move to Arm A due to an inadequate treatment response on the week 4 PET/CT scan. After substudy informed consent is signed, two substudy visits will occur where a baseline blood sample is drawn, TB medication for that day is dosed, then blood is again drawn at 1, 2, and 6 hours post-dose for pharmacokinetic (PK) analysis for isoniazid and rifampin. For every Arm A participant who agrees to join the substudy, a control participant from the combined B/C arm will also be recruited to join. This will be a convenience sample of participants willing to participate in the substudy and no specific sample size is targeted.

### Data and Safety Monitoring Board (DSMB)

The standing NIAID DSMB with three global TB experts added as ad hoc members will provide oversight of the study. The DSMB will meet at least twice per year to evaluate safety, study conduct, and scientific validity and integrity of the trial. **

### Ethical statement

Informed consent is conducted in the local language of the participant (Chinese, English, Afrikaans, or Xhosa). The study radiation dose was reviewed and approved by the Radiation Safety Committee of the U.S. National Institutes of Health (NIH). The study protocol was reviewed and approved by the institutional review board (IRB) of the National Institute of Allergy and Infectious Diseases (NIAID), NIH (085315; most recent approval numbers listed here and below, Henan sites do not use approval numbers) and all local IRB/ethics committees (Henan Provincial Chest Hospital [HPCH], Henan Center for Disease Control [Henan CDC], Stellenbosch University (M16/08/031, M16/10/039), and the University of Cape Town (645/2016, 646/2017, 647/2016)), as well as the South African Medicines Control Council.

## Discussion

With currently available drugs for TB, all published trials attempting to shorten TB treatment below 6 months have failed. The trial that came closest to success randomized only those with less severe disease and favorable early treatment response (baseline chest x-ray without cavity and a month 2 sputum culture that had converted to negative). Multiple tuberculosis biomarkers potentially predictive of treatment outcomes are being studied to improve the risk stratification of patients ^14^. From a microbiological standpoint, sputum culture conversion at 2 months of treatment is most commonly used to predict non-relapsing cure ^15^ but its true predictive ability is poor, with one meta-analysis showing a pooled sensitivity and specificity for predicting relapse of 40% (95% CI 25%-56%) and 85% (95% CI 77%-91%), respectively ^16^. A review of data from the BMRC trials from the 1970s and 1980s found only a weak correlation (R =0.36) for this marker as a surrogate for treatment failure and relapse, depending on factors such as geographic location, baseline disease and cavity status, and concomitantly used medications ^17^. Further evidence against using culture conversion as a surrogate for predicting treatment outcome was recently demonstrated from the phase 3 TB treatment shortening trial REMoxTB ^4^, where subsequent analyses of the culture data collected demonstrated poor correlation with treatment outcome whether analyzed at a single time point (2 months) or over time (time to culture conversion or time to culture positivity) ^18^.

The ability of early radiographic changes to predict subsequent treatment outcomes in TB has been recognized for over 50 years ^19^ and prior studies have identified baseline cavity on chest x-ray as a risk factor for relapse ^20^,^22^. However, chest x-rays are not sensitive for cavities, particularly smaller ones; more recent analyses of radiographic biomarkers have moved beyond chest x-ray to 2-deoxy-2-[ F]-fluoroglucose (FDG) positron emission tomography/computed tomography (PET/CT) as an early marker of treatment response and possibly as a marker for relapse at the end of treatment. CT scans produce more detailed lung morphology than chest x-rays and PET scans provide additional information on inflammatory activity. In macaques, changes on PET/CT scans correlate with TB disease activity and treatment response ^23^,^24^. Our group has analyzed human PET/CT data from a randomized clinical trial using metronidazole in the treatment of pulmonary multi-drug resistant tuberculosis (MDR-TB) participants ^25^. As a substudy within the overall MDR-TB study, we performed PET/CT scans at 0 and 2 months and CT scans at 0, 2, and 6 months of treatment and correlated these changes with final treatment outcomes 30 months after treatment start (6 months after the end of therapy). PET changes at 2 months and CT changes at 6 months appeared to be more sensitive to predict final treatment outcomes than sputum culture conversion at 2 months, although these differences were not statistically significant ^26^. These results support the potential of PET/CT imaging biomarkers as possible surrogate endpoints in clinical trials, and larger cohorts are needed to confirm these results.

We developed the radiographic early treatment completion criteria for the Predict TB trial ( Table 2) (unpublished study; National Institutes of Health, Bethesda, MD, USA) using a cohort of 100 pulmonary DS-TB participants from Cape Town who received PET/CT scans at baseline, 1 month, and 6 months while on standard therapy through the national TB treatment program ^27^. The participants were treated for 6 months, then followed through 18 months for final treatment outcomes. In total, 92 participants had complete PET/CT scan data, Xpert MTB/RIF cycle thresholds, and treatment outcomes. After 18 months of follow-up, 73 were considered cured, 8 failed treatment, and 11 were restarted programmatically on TB treatment, defined as the participant restarting treatment during follow-up for any reason. Our radiographic early treatment completion criteria are divided into baseline disease burden and week 4 reduction in disease burden due to treatment, which is reflective of the Johnson 2009 study which also had a measure of baseline disease burden (baseline chest x-ray without cavity) and treatment response (month 2 sputum culture conversion).

Currently, the only direct measure of TB sputum bacterial load is sputum smear, which is not very sensitive. Alternative surrogate markers evaluated include the time to positivity (TTP) of a positive culture on liquid mycobacterial culture systems, with the shorter TTP indicating a higher bacterial load. Different studies have demonstrated some correlation between Mycobacteria Growth Indicator Tube (MGIT) TTP and sputum bacterial load but with poor specificity in predicting treatment outcomes ^28^,^29^. Thus, although time to culture conversion and MGIT TTP do correlate independently with treatment outcome, these markers do not discriminate well between high and low risk patients and therefore have only a limited role in predicting treatment outcomes of individual patients ^18^. Another marker of sputum bacterial load is the GeneXpert cycle threshold. The GeneXpert assay is an automated rapid molecular diagnostic test for *Mtb* and resistance to rifampin with results provided directly from sputum within 2 hours ^30^. The test is run using a polymerase chain reaction and the number of cycles (cycle threshold) at which the amplification curve crosses the specified threshold is recorded, with a lower cycle threshold suggestive of a higher bacterial load. Three studies have correlated Xpert MTB/RIF cycle threshold with sputum smear status, with varying sensitivity and specificity levels depending on the cycle threshold cut point used ^31^,^34^. A fourth study found that Xpert MTB/RIF results correlated with smear grades, solid culture results, and liquid culture TTP (all p<0.0001) but Xpert MTB/RIF sputum positivity rates declined more slowly during treatment than sputum smear and culture results. Using the combined binary smear and culture results as a reference standard, sensitivity of Xpert MTB/RIF was excellent at 97.0% (95% CI 95.8-97.9) but specificity was poor at 48.6% (95% CI 45.0-52.2) as the assay is unable to differentiate viable, dormant, and non-viable *Mtb* bacteria ^35^.

In the study mentioned previously of 100 pulmonary DS-TB participants in Cape Town, a week 24 Xpert MTB/RIF cycle threshold of **≥**30 predicted treatment failure with higher sensitivity and specificity than earlier time points ^36^. This is in contrast to the week 8 culture, which has lower sensitivity (for cure) than Xpert MTB/RIF cycle threshold at week 24: 61% (week 8 culture) vs 89% (Xpert MTB/RIF cycle threshold week 24, p<0.01). Estimates of specificity (for failure) for Xpert MTB/RIF cycle threshold week 24 was higher than week 8 culture (88% vs 50%), but the improvement was not statistically significant. The poor performance of week 8 culture data as a predictor of treatment outcome is similar to what was observed in the REMoxTB trial ^18^.

The observation that a later test predicts outcomes better than an earlier test may be similar to findings in HIV infection, where baseline CD4 cell count is a strong predictor of mortality over time but current CD4 cell count is even stronger ^37^. This has been seen in TB too, where culture conversion status at month 6 predicts final treatment outcome significantly better than culture conversion status at month 2 ^38^. Taken together, these results suggest that Xpert MTB/RIF cycle thresholds collected later may be able to replace an earlier microbiological culture in predicting treatment outcomes, with the major advantage of Xpert MTB/RIF over culture being the time to test result, with Xpert requiring 2 hours and culture up to 6 weeks. Thus, Xpert cycle threshold may be useful as a point-of-care test whereas culture cannot.

In summary, the Predict TB trial builds upon previous trial results, in particular the Johnson 2009 trial ^6^. Instead of using a chest x-ray to determine baseline disease burden, we will use a PET/CT scan. Instead of using month 2 culture conversion as a measure of treatment response, we will use month 1 change in PET/CT scan disease burden and a month 4 Xpert MTB/RIF cycle threshold. Prior treatment shortening trials that randomized all subjects to shortened vs. standard treatment achieved treatment success rates in the 4-month arms of roughly 80–85%. Using a risk stratification approach, the Johnson study increased this to 93%. By refining their risk stratification parameters, we hypothesize that the treatment success rate in our 4-month arm will be non-inferior to our 6-month arm. If successful, our methodology could be extended to identify participants cured with even shorter regimens. We do not expect that PET/CT scans will become a risk stratification tool for global TB use due to its cost and availability limited to larger cities. Use of PET/CT scans would likely be limited to clinical trials but could be a method to stratify trial participants and possibly become a surrogate endpoint by which to reduce the number of participants needed in a Phase 2b trial, shorten overall trial duration or predict drug sterilizing activity such as in early bactericidal activity studies. These achievements could expand the number of regimens evaluated in Phase 2b trials prior to committing to an expensive and time-consuming Phase 3 study and thereby contribute to the likelihood of identifying optimal regimens. If the Predict TB trial is successful, other immunological biomarkers or transcriptional signatures that correlate with treatment outcome may be identified. These markers or signatures will likely be much cheaper and more widely available than PET/CT scans and more amenable to being scaled up globally.

## Supplementary material

Supplementary File 1: Manual of Operating Procedures (MOP): Clinical and Data Management.

Supplementary File 2: Manual of Operating Procedures (MOP): Laboratory (South Africa).

Supplementary File 3: Manual of Operating Procedures (MOP): Core Laboratory (China).

Supplementary File 4: Manual of Operating Procedures (MOP): Site Laboratory (China).

Supplementary File 5: SOP for Predict TB Trial PET/CT Reading Methodology.
