# Supplementary material for: Using biomarkers to predict TB treatment duration (Predict TB): a prospective, randomized, noninferiority, treatment shortening clinical trial
Source: Gates Open Res. 2017 Nov 6;1:9. [Version 1] doi: 10.12688/gatesopenres.12750.1 (PMC5841574; doi:10.12688/gatesopenres.12750.1)
Supplement: Supplementary file 1 [file gatesopenres-1-13810-s0000.tgz › d4832907-1313-413f-add9-c2685faeeed0.pdf]

Manual of Operating Procedures (MOP): Clinical and Data Management

for

Using Biomarkers to Predict TB Treatment Duration (Predict TB Trial)

NIAID Protocol #16-I-N133

Version Number 2.0

Summary of Changes:

Number	Date	Affected Sections	Summary of Revisions Made

Contents

1 Administration 5
1.1 Administrative and Institutional Review Board (IRB)/Ethics Committee (EC) Requirements 5
1.2 Protocol Amendments 5
1.3 Regulatory Requirements and Site Maintained Binders 6
1.4 Predict Protocol Team and Key Personnel 7

2 Training Plan 15

3 Communications Plan 16

4 Recruitment and Retention Plan 16

5 Drug Management and Adherence Monitoring 17
5.1 Drug Management 17
5.1.1 Overall Study 17
5.1.2 Individual Participants 17
5.2 Drug Regimen and Dosage 17
5.3 Completion of Treatment 18
5.4 Adherence Monitoring 19
5.4.1 Dose Counting 19
5.4.2 Medication Event Reminder-Monitor (MERM) Device 20

6 Study Implementation and Data Collection 22
6.1 Study Design 22
6.2 Informed Consent Process 22
6.3 Eligibility Criteria 24
6.4 Screening 25
6.5 Enrollment 27
6.6 Scheduling 27
6.7 Day 0/Baseline Visit 27
6.8 Study Visits and Randomization 29
6.9 Management of Participants Who Are Non-Adherent to Medications 38
6.10 Management of Participants With Poor Treatment Response 38
6.11 Contraindicated and Concomitant Medications 38
6.12 Logs, Flows, and Worksheets 40
6.12.1 SCREENING VISIT FLOW 40
6.12.2 ENROLLMENT/BASELINE/DAY 0 VISIT FLOW 41
6.12.3 WEEK 1 VISIT FLOW (+/- 3 day window) 42
6.12.4 WEEK 2 VISIT (+/- 3 day window) 43
6.12.5 WEEK 4 VISIT FLOW (+/- 7 day window) 44
6.12.6 WEEK 8 VISIT FLOW (+/- 7 day window) 45
6.12.7 WEEK 12 VISIT FLOW (+/- 7 day window) 46
6.12.8 WEEK 16 VISIT FLOW (+/- 7 day window) 47
6.12.9 WEEK 20 VISIT FLOW (+/- 7 day window) 48
6.12.10 WEEK 24 VISIT FLOW (+/- 7 day window) 49
6.12.11 WEEK 36 VISIT FLOW (+/- 30 day window) 50
6.12.12 WEEK 48 VISIT FLOW (+/- 30 day window) 51
6.12.13 WEEK 60 VISIT FLOW (+/- 30 day window) 52
6.12.14 WEEK 72 VISIT FLOW (+/- 30 day window) 53
6.12.15 Recurrence 54

6.12.16 PET/CT VISIT FLOW 55

7 Study Events 56
7.1 Criteria for Being Randomized at Week 16 (Protocol Table 6, sec 3.1) 56
7.2 Criteria for Achieving a Study Endpoint 56
7.3 Criteria for Stopping the Study 57
7.4 Adverse Events (AE), Unanticipated Problems (UP), Deviations, and Non-compliance 58
7.4.1 Definitions 58
7.4.2 Grading AEs for Severity 59
7.4.3 Assessing Adverse Events for Relationship to Study 60
7.4.4 Recording 61
7.4.5 Reporting Guidelines 61
7.4.6 Communications Flow for Events 64
7.5 Medically Accountable Investigator 67
7.6 Data and Safety Monitoring Board (DSMB) 67

8 Pharmacokinetic Substudy for Sub-Breakpoint Minimum Inhibitory Concentrations (MIC) Comparison 68
8.1 Substudy Design 68
8.2 Eligibility Criteria and Identification of Participants 68
8.2.1 Main Substudy Participants 68
8.2.2 Control Participants 69
8.3 Substudy Study Visits and Implementation 69
8.4 Procedure for Collection of Substudy Blood Samples 70
8.4.1 Schedule of PK Sample Collection 70

9 Data Collection and Correction 72
9.1 Data Collection Process 72
9.1.1 Source Documentation 72
9.1.2 Research Chart 73
9.1.3 CRFs 73
9.1.4 Database: DataFax 79
9.1.5 Data Entry and Submission Timelines 80
9.1.6 General Instructions for Data Entry 81
9.1.7 Data Correction and Tracking Requirements 81
9.1.8 Data Entry and Submission Timelines 83
9.1.9 Retention of Study Documentation 84

10 Site Monitoring 84
10.1 Purpose 84
10.2 Scheduling the Site Monitoring Visit 84
10.3 Components 85
10.3.1 Informed Consent Verification 85
10.3.2 Other Data Reporting 85
10.3.3 Findings 86

11 Laboratory Sample Processing Overview 87
11.1 Overall sample collection schedule 87
11.2 Sputum collection schedule and order of sputum processing 87
11.3 Sputum collection procedures 88
11.4 Safety Bloods 89
11.5 Biomarker Sample Collection 90

11.6 PK SUBSTUDY BLOOD COLLECTION AND HANDLING 92

12 APPENDIX A: MERM DOCUMENTATION 93

12.1 Participant ID/activation date sticker 93
12.2 “Do not remove this cartridge” sticker 96
12.3 Reminder sticker with site address and contact information 99
12.4 Participant instruction sheet 101
12.5 MERM User Guide 104
1. About this User Guide 107
2. MERM Software Installation 107
3. Overview of the MERM 107
4. MERM Software Interface 111
5. Understanding the Menu 112
6. MERM Setup / Laptop (or Desktop) Connection 113
7. Baseline Visit Instructions 114
8. Follow-up Visit Instructions 115
9. Drug Adherence Graph 116
10. Data Transfers 117
11. Upload Log File 117
12. User Support 118

1 Administration

1.1 Administrative and Institutional Review Board (IRB)/Ethics Committee (EC) Requirements

International sites must comply with local regulations and policies as well as the policies set forth by the OHRP, as stated by the site's Federal Wide Assurance.
http://www.hhs.gov/ohrp/assurances/assurances/index.html

All participating sites must obtain local EC/IRB approval for the protocol, informed consent form (s), MERM education sheets, and any advertisements. Sites are required to fulfill their individual institution's review requirements in advance of IRB/EC, including scientific review, radiation safety review, and any other required institutional reviews.

After EC/IRB approval is obtained, a copy of the approval documents, including the approved protocol and ICFs, should be sent to the NIAID Clinical Research Manager Kriti Arora, arorakriti@niaid.nih.gov. In succession or concurrently, Medicines Control Council (MCC) approval shall be sought and obtained before participant Informed Consent and enrollment in South Africa. Triclinium will assist in the application for this approval and NIAID will be the organizing site.

1.2 Protocol Amendments

Proposed changes in a protocol, usually called amendments, must be agreed upon by all sites affected. When changes are made to the protocol, a revised protocol should first be distributed to all participating sites for review. When consensus is made, the sites must submit the new version of the protocol to their local EC/IRB for approval.

- It is critical that the site submit protocol amendments (and revised informed consent forms, if appropriate) to the reviewing EC/IRBs as soon as possible after the decision that a final protocol AM has been made.
- If the amendment states whether expedited or full board review is most appropriate, the site should take this into consideration when presenting the amendment to the local EC/IRB.
- Amendments may not be initiated without both NIAID IRB approval and local EC/IRB approval, except when necessary to safeguard participants from immediate hazards.

  Once local EC/IRB approval is obtained, the following must be submitted to the NIAID study staff (arorakriti@niaid.nih.gov):

- Copy of the local EC/IRB approval letter for the new version of the protocol; and
- EC/IRB-approved copy of the protocol; and
- EC/IRB -approved copy of the informed consent form, if changes to the consent are required (including any translated consent forms and attestation of their accuracy).

1.3 Regulatory Requirements and Site Maintained Binders

All sites are required to maintain a regulatory binder for each protocol. Essential documents required by the ICH/GCP Guidelines are to be maintained in the regulatory binder for the duration of the study and kept for a minimum of 10 years after the completion of the study or per local guidelines. The following documents should be maintained in this binder:

- Copy of the current and previous versions of protocol(s)
- Copy of letter from the Office of Clinical Research Policy and Regulatory Operations (OCRPRO) that the site can begin enrollment
- IRB approval documentation for:
  - Study protocol(s)
  - Protocol amendment(s)
  - Other written (educational) materials provided to participants
  - Annual renewals/Continuing Reviews
  - Study advertising
  - Informed consent(s)
- Copies of EC/IRB -approved original and amended versions (either EC/IRB approval letter or stamped consent from all applicable EC/IRBs, including NIAID IRB)

  (Note: Original signed participant informed consents are usually kept in the participant's research record. Follow institutional requirements.)
- Common study source documents
- Safety reports
- Data and Safety Monitoring Board (DSMB) reports
- Annual summary of study progress (copy of continuing review submissions)
- Study closeout information
- Curricula vitae and documentation of professional licensure of investigators
- Human participants protection training certifications
- Laboratory certifications
- Normal range values from all laboratories used by the site

- Financial disclosure forms
- EC/IRB correspondence
- Delegation of Authority and Signature log (see section attachments)
    - This is a list of signatures and initials of all persons authorized to perform study-related procedures and/or prescribe study medications.

To facilitate organized and accurate maintenance of the Regulatory Binder, it is permissible to:

- Maintain regulatory documents common to all studies in an organized and labeled central binder.
    - Types of documents include CVs and licenses, training certificates, laboratory certifications, laboratory normal range values, EC/IRB rosters, EC/IRB assurances, monitoring reports, Site Visit Log, and nonstudy-specific communications.
- Maintain archived regulatory documents in an organized binder separate from current regulatory documents.
    - Types of documents include expired/no longer applicable: EC/IRB approvals (protocols and consents), laboratory certifications, laboratory normal ranges, CVs, and financial disclosure forms.
    - Do not maintain study-specific archived regulatory documents with a different study's specific archived documents, i.e., each study must have a separate archive binder if used.

1.4 Predict Protocol Team and Key Personnel

Sponsor Principal Investigator

Clifton E Barry, 3rd PhD
Tuberculosis Research Section
Room 2W20D, Building 33
9000 Rockville Pike
Bethesda, MD 20892
Phone: (301) 435-7509
Fax: (301) 480-3506
Email: cbarry@niaid.nih.gov

Sponsor Medically Accountable Investigator

Ray Chen, MD, MSPH
Staff Clinician, Tuberculosis Research Section
Laboratory of Clinical Infectious Diseases
National Institute of Allergy and Infectious Diseases
National Institutes of Health

9000 Rockville Pike, MSC 3206
Bldg 33, Rm 2W20
Bethesda, MD 20892
Phone: 301-443-5816
Fax: 301-451-5492
Email: ray.chen@nih.gov

Sponsor Lead Scientific Investigator

Laura E. Via, PhD
Associate Staff Scientist and Project Officer, Tuberculosis Research Section
Laboratory of Clinical Infectious Diseases
National Institute of Allergy and Infectious Diseases
National Institutes of Health
9000 Rockville Pike, MSC 3206
Bldg 33, Rm 2W20G
Bethesda, MD 20892
Phone: 301- 451-9554
Fax: (301) 480-5713
Email: lvia@niaid.nih.gov

❖ South African Sites and Investigators

Site Name	Country	City	Assurance Type/Number	Role	IRB/IEC
University of Cape Town (UCT) Lung Institute	South Africa	Cape Town	FWA00001637	Enrollment	UCT HREC
Stellenbosch University (SUN)	South Africa	Cape Town	FWA00001372	Enrollment/Sample processing/ Immunology Lab	Stellenbosch HREC
TASK Applied Science, Inc	South Africa	Cape Town	FWA00017533	Enrollment	Stellenbosch HREC
Khayelitsha Site B (CIDRI)	South Africa	Khayelitsha	FWA00001372	Enrollment/Sample processing	UCT HREC
South African Tuberculosis Vaccine Initiative (SATVI)	South Africa	Cape Town	FWA00001372	Enrollment/Sample processing	UCT HREC

Stellenbosch University

South African National and Local PI

Gerhard Walzl, MBChB, Mmed, PhD
Division of Molecular Biology and Human Genetics
Faculty of Medicine and Health Sciences,
Stellenbosch University
South Africa
Phone: +27 21 938 9401

Email : gwalzl@sun.ac.za

TASK Applied Sciences, Inc.
Local PI
Andreas Diacon, MD
Task Applied Sciences;
Medicine and Health Sciences, Stellenbosch University
+27 021 9171044
ahd@sun.ac.za

University of Cape Town (UCT) Lung Institute
Local PI
Rodney Dawson MBChB, FCP(SA), Cert. Pulm.(SA)
Head, Centre for TB Research Innovation,
UCT Lung Institute
+27 (0)21 406 6850

Khayelitsha Site B
Local PI
Friedrich Thienemann, MD
Senior Research Medical Officer
S3.13 WBS & Khayelitsha Clinic
Friedrich.Thienemann@uct.ac.za
+27 21 406 6084

South African Tuberculosis Vaccine Initiative (SATVI)
Local PI
Michele Tameris MBChB
Clinical Researcher
Worcester
Western Cape
6850
South Africa
023 346 5400

Investigators

Name, Degree	Professional Designation	Organization (Institute/Branch)	Phone #	Protocol Role	Medical Decision Maker	Informed Consent
Jacqui Cirota, MD	Physician	UCT Lung Institute	+27 21 406-6815	Investigator	Yes	Yes

Rod Dawson, MD	Head, Centre for TB Research Innovation	University of Cape Town (UCT) Lung Institute	+27 (0)21 406 6850	Local PI	Yes	Yes
Elsa Du Bruyn, MD	Physician	Clinical Infectious Diseases Research Initiative (Khayelitsha site)	+27 21 406 6084	Associate Investigator	Yes	Yes
Andreas Diacon, MD	Professor	Task Applied Sciences	+27 21 917 1044	Local PI	Yes	Yes
Jeannine du Bois, MD	Physician	Task Applied Sciences	+27 21 917 1044	Associate Investigator	Yes	Yes
Hendrik Geldenhuys, MBChB	Clinical Research Officer	SATVI	27233465 400	Associate Investigator	Yes	Yes
Rene Goliath, RN	Project Coordinator	Clinical Infectious Diseases Research Initiative	02165019 38	Associate Investigator	Yes	Yes
Madeleine Hanekom, MD	Physician	Task Applied Sciences	+27 21 917 1044	Associate Investigator	Yes	Yes
Mark Hatherill, MD	Associate Professor	South African Tuberculosis Vaccine Initiative (SATVI), University of Cape Town	27214066 791	Associate Investigator	Yes	Yes
Bronwyn Hendricks, MD	Physician	UCT Lung Institute	+27 21 406-7747	Investigator	Yes	Yes
Rene Kitshoff, B. Pharm	Pharmacist	Task Applied Sciences	+27 21 917 1044	Pharmacist	No	Yes
Andre Loxton, PhD	Senior Scientist	Stellenbosch University	27 21-9389399	Associate Investigator	No	Yes
Angelique Luabeya, MBChB	Clinical Research Officer	SATVI, University of Cape Town	27233465 400	Associate Investigator	Yes	Yes
Elizna Maasdorp, MD	Study Clinician	Stellenbosch University	27 21 938 9399	Associate Investigator	Yes	Yes
Fanie Malherbe, MD	Physician	Stellenbosch University	27 21 938 9399; 27 84 225 3227	Associate Investigator	Yes	Yes
Bronwyn Smith	Project coordinator	Stellenbosch University	+27 21 9389399	Associate Investigator	No	Yes
Sandra Mukasa, MD	Physician	Clinical Infectious Diseases Research Initiative (Khayelitsha site)	+27 21 406 6084	Associate Investigator	Yes	Yes
Mark Nicol, MD	Professor	University of Cape Town	+27 21 4066083	Associate Investigator	Yes	Yes
Kim Narunsky, MD	Physician	UCT Lung Institute	+27 21 406-6605	Investigator	Yes	Yes
Ramonde Patientia, MD	Physician	Task Applied Sciences	+27 21 917 1044	Associate Investigator	Yes	Yes

Justin Shenje, MBChB	Clinical Research Officer	SATVI	27233465 400	Associate Investigator	Yes	Yes
Taeksun Song, PhD	Lab Head	UCT		Associate Investigator	No	No
Michele Tameris MBChB	Clinical Research Officer	SATVI	27233465 400	Local PI	Yes	Yes
Friedrich Thienemann, MD	Physician	Clinical Infectious Diseases Research Initiative (Khayelitsha site)	+27 21 406 6084	Local PI	Yes	Yes
Elana van Brakel, MD	Physician	Task Applied Sciences	+27 21 917 1044	Associate Investigator	Yes	Yes
Gerhard Walzl, MBChB, MMed, PhD	Professor	Stellenbosch University	27 21 938 9401	Local PI	Yes	Yes
Robert Wilkinson, MA, PhD, BM, BCh, DTM&H, FRCP	Professor	Clinical Infectious Diseases Research Initiative (Khayelitsha site)	+27 21 406 6084	Associate Investigator	Yes	Yes
Petri Ahlers, MD	Study Clinician	Stellenbosch University	27 21 938 9639	Associate investigator	Yes	Yes
Mpho Tlali, MD	Study Clinician	Stellenbosch University	27 21 938 9639	Associate investigator	Yes	Yes
Novel Chegou, PhD	Senior Scientist	Stellenbosch University	27 21 938 9953	Associate investigator	No	Yes
Andriette Hiemstra, MD	Study Clinician	Stellenbosch University	27 21 938 9639	Associate investigator	Yes	Yes
Unice Vorster, MD	Study Clinician	CIDRI	27 21 361 4592	Associate investigator	Yes	Yes

❖ China Sites and Investigators

Site Name	Country	City	Assurance Type/Number	Role	IRB/IEC
Henan Provincial Chest Hospital (HPCH)	China	Zhengzhou, Henan Province	FWA00014285	Enrollment/Lab	HPCH IRB
Xinmi County Institute of Tuberculosis Prevention and Control: #492	China	Xinmi, Henan Province, China	FWA00023940	Enrollment/Lab	HPCH IRB
Zhongmu County Epidemic Prevention Station: No.1106	China	Zhongmu District, Zhengzhou, Henan Province, China	FWA00023941	Enrollment/Lab	HPCH IRB
Kaifeng Tuberculosis Prevention and Treatment Institute	China	Kaifeng, Henan Province, China	FWA00024110	Enrollment/Lab	HPCH IRB
HPITPC	China	Zhengzhou, Henan Province	FWA00008609	Lab	HPCH IRB

Investigators

Henan Provincial Chest Hospital (HPCH)
Local PI

Dr. Xin Liu, MD
Director, TB Ward 6, Henan Provincial Chest Hospital
Zhengzhou, Henan Province, China
Phone: +86 138 3717 1629
Email: liuxin1962@hotmail.com

Henan Provincial Institute of Tuberculosis Prevention and Control (HPITPC)

Dr. Xing Jin
Director
Henan Province
China
451450

Xinmi City Center for Disease Control and Prevention
Local Co-PI

Dr. Xiaowei Jin
East Main St,
Xinmi County
Zhengzhou City
Henan Province
China
452370

Zhongmu County Station for Disease Control and Prevention
Local Co-PI

Dr. Dakuan Wang
Western part of Qingnian Rd
Zhongmu County
Zhengzhou City
Henan Province
China
451450

Kaifeng Tuberculosis Prevention and Treatment Institute
Local PI

Dr. Zhenya Ma
W Huancheng Rd North Section,
Kaifeng Henan Province China

Phone:+86 371-23316669
Email: 861852536@qq.com

HPCH AIs in Alphabetical Order

Name, Degree	Professional Designation	Organization (Institute/Branch)	Phone #	Protocol Role	Medical Decision Maker	Informed Consent
Ying Chen, MD	Chief Physician	HPCH	+86 371 6566 2949	Associate Investigator	Yes	Yes
Xia Gao	Direct of research and foreign affairs department	HPCH	+86 371 6566 2791	Associate Investigator	No	No
Bin Li, MD	Chief Physician	HPCH	+86 371 6566 2556	Associate Investigator	Yes	Yes
Lili Liang, MD	Deputy Chief Physician	HPCH	+86 371 6566 2949	Associate Investigator	Yes	Yes
Xin Liu, MD	Director of No.6 TB Department/Senior Physician	HPCH	+86 371 6566 2826	Local PI	Yes	Yes
Xianglin Ruan, MD	Director of No.4 TB Department/Senior Physician	Henan Public Health Clinical Center	+86 371 6566 2556	Co- PI	Yes	Yes
Huimin Shi, MD	Chief Physician	HPCH	+86 371 6566 2556	Associate Investigator	Yes	Yes
Wei Wang, PhD	Director of Laboratory /Deputy Chief Technician	HPCH	+86 371 6566 2785	Co-Local PI	No	No
Rui Yang, MD	Chief physician for imaging diagnosis	HPCH	+86 371 6566 2665	Associate Investigator	No	No
Hongli Zhang	Technician	HPCH	(+86) 371 6566 2948	Associate Investigator	No	No
Yue Zhao	Technician	HPCH	(+86) 371 6566 2948	Associate Investigator	No	No
Shulan Zheng, MD	Chief Physician	HPCH	+86 371 6566 2949	Associate Investigator	Yes	Yes

Xinmi County Institute of Tuberculosis Prevention and Control (XCITPC), Zhongmu County Epidemic Prevention Station (ZCEPS), and Henan Provincial Institute of Tuberculosis Prevention and Control (HPITPC) AIs and other Chinese collaborators in Alphabetical Order

Name, Degree	Professional Designation	Organization (Institute/Branch)	Phone #	Protocol Role	Medical Decision Maker	Informed Consent

Shaoli Cao, MD	Director, TB prevention and Control Department	Gongyi Center for Disease Control and Prevention	(+86) 371 6459 9120	Associate Investigator	No	No
Qian Gao, PhD	Professor	Fudan University	(+86) 136 7164 5425	Associate Investigator	No	No
Yuping Huang	Deputy Director	KCITPC	(+86) 371-23310006	Associate Investigator	No	No
Hongjian Jin	Deputy Chief Physician	Xinmi County Institute of Tuberculosis Prevention and Control (XCITPC)	(+86) 371 6987 2897	Site Co-PI	No	No
Xiaowei Jin	Director of TB Department	XCITPC	(+86) 371 6987 2897	Associate Investigator	No	No
Xing Jin, MD	Director, Senior Physician	Henan Provincial Institute of Tuberculosis Prevention and Control (HPITPC)	(+86) 371 6808 9286	Site Principal Investigator	Yes	Yes
Xiaowei Jin, MD	Director	Xinmi County Institute of Tuberculosis Prevention and Control (XCITPC)	(+86) 371 6987 2897	Site Co-PI	Yes	Yes
Hongliang Li	Director, TB prevention and Control Department	Xingyang County Epidemic Prevention Station	(+86) 371 6462 9220	Associate Investigator	Yes	Yes
Hui Li, MD	Chief Technician	HPITPC	(+86) 371 6808 9284	Associate Investigator	No	No
Liping Ma, MD	Deputy Director	HPITPC	(+86) 371 6808 9284	QA/QC Specialist	No	No
Zhenya Ma, MD	Director	Kaifeng City Institute of Tuberculosis Prevention and Control (KCITPC)	(+86) 371-23316669	Site Co-PI	Yes	Yes
Shouguo Pan, MD	Director	Zhongmu County Epidemic Prevention Station (ZCEPS)	(+86) 371 6218 1458	Site Co-PI	Yes	Yes
Dakuan Wang, MD	Director, TB prevention and Control Department	Zhongmu County Epidemic Prevention Station	(+86) 371 6219 0195	Site Co-PI	Yes	Yes
Jianwei Yang	Director, PET-CT Department	Henan Provincial Tumor Hospital (HPTH),	(+86) 371 6558 8176	Associate Investigator	Yes	No
Guolong Zhang, MD	Deputy Director, HPHCC	Henan Public Health Clinical Center (HPHCC)	(+86) 371 6808 9063	Associate Investigator	No	No

Hong Zhu, MD	Deputy Chief Physician	Sino-US TB Project	(+86) 371 6557 9050	HPCH Site Project Manager/ AI	No	Yes

❖ US and Other Investigators

Name, Degree	Professional Designation	Organization (Institute/Branch)	Phone #	Protocol Role	Medical Decision Maker	Informed Consent
David Alland, MD	Professor	Rutgers New Jersey Medical School	973-972-2179	Associate Investigator	No	No
John Belisle, PhD	Professor	Colorado State University	970-491-5384	Associate Investigator	No	No
Ying (Chrissie) Cai, MS	Data Management Specialist	Kelly Government Solutions (Contractor for NIH/NIAID/LCID/TRS)	301-443-5702	Data Management and support	No	No
Lori Dodd, PhD	Statistician	NIH/NIAID/DCR/BRB	301-451-2485	Statistician	No	No
Kriti Arora, PhD	Clinical Research Manager	NIH/NIAID/LCID/TRS	301-761-6252	Protocol Management and Liaison Coordinator	No	No
Michael Mix, PhD, MSc	Medical Physicist	University of Freiburg, Germany	+49 761 27039650	Associate Investigator	No	No
Jill Winter, PhD	Program Director	Catalysis Foundation	510-289-5766	Associate Investigator	No	No

2 Training Plan

Study staff must have documentation that they have received proper training to be performing their role on study. All study staff, no matter what role, must complete Good Clinical Practices (GCP) training before performing trial related activities. This training can be in-person or can be obtained online at the following links:
https://gcplearningcenter.niaid.nih.gov/Pages/default.aspx
www.citiprogram.org

The following are basic learner instructions to logon to your CITI site for the first time.

- Go to www.citiprogram.org to register for CITI online training.
- Select language from the pull down menu top right if needed.
- Once there, simply click on **"New Users Register Here"** and follow the steps
- Under "Select your institution or organization" page in the **"Participating Institutions"** drop down box select **RCHSPB/DCR/NIAID/NIH**
- Proceed to create their own username and password and select the Learner group.
- Select GCP for Clinical Trials Involving Drugs (ICH international focus) unless you have been instructed otherwise.

- Once logged in you may select the language of choice by clicking on the **SELECT LANGUAGE button** in the upper right of the window.
- Once you have completed the training you will be able to print a certificate of completion for your training files.
- You may log out at any time and come back in to complete the training.

Specific protocol training, including adverse event, informed consent training, laboratory, and database trainings will be provided to the sites.

3 Communications Plan

Open communication is key for a successful study.

Weekly team meetings will occur as the study begins. This will eventually change to monthly team meetings throughout the course of the study. These teleconferences will be planned by the project manager or other study staff and study specific topics and issues will be discussed. Representatives from each study site will be encouraged to join the call. The frequency of meetings will be assessed as the study goes on and adjusted as necessary. Minutes will be recorded and distributed to the team.

For overall study information, a study website is available, PredictTB Space, and is accessible with a password. Study documents, team contact details, study calendar, enrollment statistics, and other study-related updates will be accessible on this site.
Website link: https://www.scienceforum.sc/sites/predicttb/_layouts/15/start.aspx#/

For communication flow due to an adverse event, unanticipated problem, please see section 7, specifically 7.4.6.

Specific in-country regulatory issues that deal with issues regarding country regulatory bodies can be addressed to the in-country regulatory expert or CRO. If the CRO is addressed directly, please inform the NIAID staff of the issue and intended plan of action for the situation.

4 Recruitment and Retention Plan

Each site will recruit participants from their local catchment areas according to their own standard procedures. An emphasis will be made on recruiting first-time TB patients. We will recruit all people who meet the inclusion/exclusion criteria without considering severity of disease because people with more serious disease will be put in Arm A and will therefore still be part of the study.

Every effort will be made to retain participants enrolled onto the trial. If possible, participants should be called the week of or the day before their next study visit appointment to remind them to come. In particular, this should be done before key study visits (week 4 PET/CT and week 16 PET/CT and before each post-treatment follow-up visit). Different strategies may be used to maintain participant retention on study, such as the use of local village doctors to encourage retention. It is important to keep participants on study as the primary endpoint is at

18 months. If participants cannot be retained until 18 months, they will not contribute to the primary endpoint.

5 Drug Management and Adherence Monitoring

5.1 Drug Management

5.1.1 Overall Study

Investigators and study staff should maintain proper drug accountability records for drug management, as is required by their site and local regulatory authorities who provided the drugs. Drugs for this study are not considered study drugs, as they are the standard of care regimen, and therefore accountability is not as strict as it would be for an investigational product. For example, remaining drugs do not need to be collected and destroyed at the end of each follow-up period or at the end of the study. Remaining drugs at the end of each follow-up period may be re-dispensed to the same individual for the next follow-up period. At the end of the study, any remaining, non-dispensed drugs may be used or disposed of according to requirements of the local regulatory authority who provided the drugs.

5.1.2 Individual Participants

The site should maintain proper documentation for the distribution and accountability of all drugs given to a participant on study. Documentation should include:

- Source document: Adherence chart; Pharmacy Accountability Log; Drug Log, Pill Count Cards
- CRF: Adherence Monitoring (ADHERE); MERM lid opening data download (eCRF only; directly uploaded to DataFax)

5.2 Drug Regimen and Dosage

Drugs for the intensive phase and continuation phase of treatment will be taken orally, seven days a week, throughout treatment using locally available fixed dose combination tablets. For the best absorption, tablets should be taken on an empty stomach, either 1 hour before or 2 hours after eating. However, if participants do not tolerate the drugs on an empty stomach, taking with food is permissible. Drug dosing will be per local standard of care guidelines.

South Africa:

According to the South African National Tuberculosis Management Guidelines 2014, new and previously treated adults and children >8 years/>30 kg will receive Regimen 1:

REGIMEN 1: FOR ADULTS AND CHILDREN OLDER THAN 8 YEARS OR WEIGHING MORE THAN 30KG

Pre-treatment body weight	Intensive Phase 7 days a week for 2 months	Continuation phase 7 days a week for 4 months	
	RHZE (150,75, 400,275)	RH (150,75)	RH (300,150)
30-37 kg	2 tabs	2 tabs	
38-54 kg	3 tabs	3 tabs	
55-70 kg	4 tabs		2 tabs
>70kg	5 tabs		2 tabs

Treatment is provided as a fixed dose combination tablet with one intensive phase tablet type incorporating four drugs and two continuation phase tablet types incorporating two drugs of different dosages. It will be important to document which of the two continuation phase tablets is dispensed for each participant.

China:
In China, the 4-drug combination intensive phase tablet is provided in the same strength as in South Africa (RHZE 150/75/400/275 mg) with the same weight-based dosing guideline as the South African guideline in the table above. The 2-drug combination continuation phase tablet, is provided as an RH 300/150 mg tablet only. Those who weigh 50-54 kg will receive 2 tablets, per Chinese dosing guidelines. For those under 50 kg, single drug pills will be provided.

Both countries:
Participants with a weight that falls between the weight bands in the table above should be rounded up to the next weight band as it is expected that their weights will increase as they improve on treatment. In addition, participants should be weighed at each follow-up visit and doses adjusted to the participants' current weight.

In addition to the daily fixed dose combination tablets, vitamin B6 (pyridoxine) may also be dispensed for daily treatment, per local site standards.

Other adverse effects of TB treatment, such as nausea, vomiting, abdominal pain, jaundice, arthralgias, visual changes, or rash, should be managed according to the local standard of care. Drug should be stopped as needed if drug-induced hepatitis is suspected. Note that according to protocol sec. 5.10, if permanent discontinuation of any drug is required due to an adverse event, that participant will be moved to Arm A. If drug treatment is held and subsequently restarted, the participant will be managed according to protocol sec. 5.2 regarding the number of doses missed (see section below).

5.3 Completion of Treatment

Total treatment duration will be determined by total dose counts, determined at every study visit. Participants will receive either 16 weeks of treatment (Arm C) or 24 weeks of treatment

(Arms A and B). Arm A participants may be treated beyond week 24 at the discretion of the treating physician.

5.4 Adherence Monitoring

All possible and available forms of adherence monitoring are encouraged as much as local resources allow. This includes (but does not require) directly observed therapy, whether by a healthcare worker, an outreach worker, or a family member. An electronic pill box, the Medication Event Reminder Monitor (MERM), may also be used.

5.4.1 Dose Counting

When drugs are dispensed into the MERM (if used) at each visit, the blister packs may be cut up and packaged into daily doses to reduce confusion for the participant as to how many pills to take each day and to reduce the complexity of dose counting. This way, if 4 pills remain, it is more clear whether this was a missed 4-pill dose or if the participant took partial doses on multiple days instead. Participants should be instructed to save empty dose packs in the MERM for dose counting purposes.

A dose count determination should be made at every visit during treatment. **This is critically important to do as accurately as possible because there is a precise dose count requirement in the early treatment completion criteria.** However, unless strict DOT is done, determining how many doses a participant actually took is not always straightforward. For example, if a participant is supposed to take 4 pills/day and returns with 4 pills remaining, does this mean s/he missed a single full dose or inadvertently took several partial doses? Taking a partial dose should count as a missed dose for dose counting purposes. **Site staff should utilize all available information to make their best judgment as to the total dose count taken for that follow-up period.** Available information includes participant adherence self-report, manual dose counts, MERM box lid open data, DOT records, etc. When available data are discrepant (e.g. patient self-reports no missed doses but MERM data show no openings on certain days), a discussion with the participant is necessary to resolve the discrepancy. If the discrepancy cannot be resolved even after discussing with the participant, the site staff must make his/her best educated guess as to the actual number of doses taken. The final determined dose count for that follow-up period is recorded on the source documents and study CRF. **A cumulative dose count, including the doses taken at all previous study visits, should be maintained by each site for each participant.** Participants in Arm B/C who miss doses at a rate approaching or exceeding on average 1 dose/week are in danger of missing the 100 dose minimum count at week 16 to be randomized if this low adherence rate continues and thus should receive adherence counseling as soon as possible during follow-up. Participants who do not achieve this minimum dose will be moved to Arm A.

Missed doses during the initial 8 week intensive phase will be added on to the end of the intensive phase, replacing continuation phase dosing. These missed but replaced intensive phase doses will still be counted as missed for dose count purposes.

Arms A and B participants at week 24 should have received a total of 168 doses, with a minimum of 150 doses required to be considered treatment complete. Participants who do not reach 150 doses by week 24 should continue to be dosed until 150 doses are reached.

If the MERM is used, participants should be instructed to bring their MERM with them to every visit during treatment so that the remaining doses and empty blister packs can be counted, MERM open-close data can be downloaded (see next section), and the number of medication doses in the box replenished for the next visit.

1. The study staff should open the MERM and remove the empty blister packs. The number of pills missing from the packs should be calculated and recorded. The pills still left in all blister packs should also be counted and recorded. The number of pills remaining and dispensed should be recorded on the Adherence source document at every visit during treatment.
2. Note that adherence data recorded should be as dose counts rather than pill counts. Incomplete doses taken should be counted as missed doses.
3. Participants should be encouraged to record doses taken on the self-reported adherence log and to keep this log inside the box. Study staff should review this adherence log at each visit. Used logs should be kept in the participant's study binder as source and a new log (if needed) should be placed in the MERM to record adherence through to the next visit.
4. Open the MERM user interface software program and connect the USB cord from your computer to the MERM cartridge. Lid open data will automatically download to your local computer, then automatically upload to the study database. No manual processes are needed. A graph of the MERM lid open data will appear in the MERM user interface.
5. Review all available adherence data (e.g. patient self-report, adherence log, dose counts, and MERM open-close data) and, with the participant, make a decision as to the actual number of doses taken during this follow-up period (as described above).
6. Replenish the MERM (or dispense to the participant if MERM is not used) enough extra days of medication to cover the window period until the next visit in case the participant is late to the next follow-up appointment. The window periods during treatment are:
    a. Weeks 1-2: +/- 3 days
    b. Weeks 4-24: +/- 7 days

   Unused medication from the previous follow-up period may be re-dispensed to the same participant for the next follow-up period.
7. Participants again should be instructed to keep their empty blister packs (kept in the MERM) to count for adherence. Participants should self-record doses taken on the adherence log kept in the MERM.

5.4.2 Medication Event Reminder-Monitor (MERM) Device

5.4.2.1 *MERM Acquisition and Setup*

Each site will be provided MERM devices by the NIAID study team or designee. A designated team member at each site will be responsible for setting up the MERM, according to the MERM Setup Work Instruction as below:

Materials needed for set up:
1. MERM box (cardboard box in Cape Town, plastic box in Henan)
2. MERM cartridge
3. Battery (2 AA alkaline batteries per cartridge); note Energizer E91 AA batteries were tested by Wisepill and lasted 1 year
4. Mini-USB connector cord:

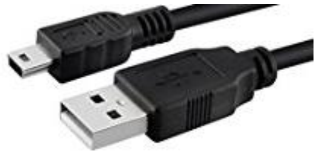

5. Pre-installed software on a windows machine
6. Participant ID/activation date sticker (to be printed on Avery 5160 or equivalent label sheet)
7. “Do not remove this cartridge” sticker (to be printed on Avery 5160 or equivalent label sheet; see MERM appendix A)
8. MERM participant instruction sheet (see MERM appendix A)
9. MERM reminder sticker with site address and contact information (to be printed on Avery 5389 or 5889 or equivalent label sheet; see MERM appendix A).
10. A water-resistant bag to place the MERM in (resealable, plastic zip-top bag or any type of bag the sites choose which protects the MERM from water)

Procedure for preparing MERM box for participant:
- Set up the MERM software and activate the MERM box as outlined in the MERM user guide (see MERM appendix A).
- Complete the “participant ID/activation ID sticker” with the participant ID and activation date. Place this sticker on the back of the MERM cartridge such that it covers the opening for the battery to deter the participant from removing the battery. Tell the participant not to remove the battery.
- Place the “Do not remove this cartridge” sticker over the cartridge such that one end of it is on the front of the box (going over the cartridge) and the other end is at the back side of the cartridge to deter the participant from removing the cartridge. Tell the participant to not remove the cartridge.
- Place the “MERM reminder sticker” on the lid of the box, either on the inside or on the outside (as per the preference of the site/participant). Remind the participant to bring the box with him/her to every visit and inform him/her that s/he can contact the number listed for questions or concerns.
- Place a copy of the MERM participant instruction sheet in the box.
- Place the medications in the box, recording the number of pills/doses dispensed on the source document.
- Place the box in a bag and hand it to the participant.

5.4.2.2 ***Training***

MERM training will be provided by the study team prior to study start. Training will consist of full operations of the MERM device, including but not limited to

downloading data, filling the container, changing the batteries, programming of alarms, and error checks.

5.4.2.3 *Data Collection*

MERM lid open data should be downloaded at each study visit according to the MERM User Guide (see MERM appendix A). Please notify the NIAID IT team (Michael Duvenhage, michael.duvenhage@nih.gov) in the event of a MERM technical failure.

The providers should fill out the MERM questionnaire once after the first 1-3 participants have begun using the MERM.

5.4.2.4 *Forgotten MERM box*

If the MERM is not brought to a study visit, the participant should be re-scheduled to return on another day within the visit window and to bring the MERM. If the MERM is forgotten, adherence can only be measured based on participant self-report, which is not reliable. Note that a sputum should still be collected while the participant is at the clinic in case s/he does not return.

Reminders at each visit should be given to bring the MERM to the next visit.

6 Study Implementation and Data Collection

6.1 Study Design

This is a prospective, randomized, phase 2b noninferiority trial in pulmonary DS-TB participants. Eligible participants will initially receive HRZE for 8 weeks, then switch to HR. Early treatment completion criteria will be evaluated for each participant up through week 16. Those who do not meet the early treatment completion criteria will be put into Arm A (2HRZE/4HR). Those who meet early treatment completion criteria will be randomized at week 16 either to continue therapy to week 24 (Arm B) or to complete therapy early at week 16 (Arm C). Randomization will be stratified by site. All participants will be followed until approximately 18 months from their start of the study, with the primary endpoint evaluated at 18 months.

6.2 Informed Consent Process

Written informed consent, using IRB-approved consent forms (ICF), will be obtained by trained study personnel prior to performing any study-specific procedures. Informed consent is a process that will be initiated prior to the individual's agreeing to participate in the study and will continue throughout the individual's study participation. Potential participants will receive information about risks and possible benefits of study participation, study objectives and procedures, and potential toxicities in the informed consent process. Informed consent requires the legally effective signature or mark of the participant. A copy of the signed and dated informed consent document will be given to each participant for his/her records. The rights and welfare of the participants will be protected by emphasizing to participants that the quality of their medical care will not be adversely affected if they decline to participate in this study, and that they may withdraw consent at any time. Individuals who choose not to participate in the study will be referred to local sources of TB care.

The investigator will ensure that the purpose of the study is explained to the participant and that written consent is obtained prior to participation in the study. The participant, investigator or designee, or others as required by local regulatory guidelines will sign the consent prior to entry into the study. The investigator will retain a copy of the signed consent forms, which may be inspected at the monitor's or auditor's request.

Documenting the consent process will include the issues detailed above. Documentation should be included in the participant's medical/research chart. Following local IRB requirements, other regulatory requirements, and site-specific practices, document that the participant had a chance to ask questions, that their questions were answered, and that the participant verbalized understanding.

Potential participants will be given informed consent for the main study, along with the study leaflet. A Genetic and Stored Samples ICF will also be given at SUN and TASK. At TASK, the consent for HIV testing will be documented on a separate HIV ICF. A PK sub-study ICF will be also be given if the participant is interested.

Key points to highlight

Several key points about the study are important to emphasize during the informed consent process. Some of these key points are highlighted in the informed consent flipbook, which should be reviewed with each participant. Note that this informed consent flipbook does not comprehensively cover the entire informed consent document and therefore cannot replace the informed consent document.

It is very important to remind the study participant during the informed consent that a small proportion of treatment failure or relapse occurs normally with the 6-month SOC treatment. We do not expect the 4-month treatment arm to do better than the 6-month treatment arm. Because a few people normally relapse even with 6 months of treatment, we also expect a few people to relapse with 4 months of treatment. This small proportion of relapses may or may not have anything to do with the 4 vs. 6-month treatment duration and we may not know the relationship until after the study. However, we will monitor this proportion carefully during the study such that if this proportion exceeds the expected non-inferiority margin between Arms B and C, we will notify the study Data and Safety Monitoring Board who will consider whether or not to stop the trial early.

The point to emphasize to the study participant is that a few relapses are expected in the 4-month arm as a part of routine treatment. There are many factors that affect whether or not a person fails or relapses in addition to shorter treatment duration, such as poor adherence, adverse events, disease severity, or individualized treatment responses.

If a study participant does fail or relapse, s/he will be referred for retreatment. The study team will observationally follow all of those who relapse on Arms B and C until the end of retreatment for outcome determination. The study team may also check national/local TB records to evaluate for unreported TB recurrences and for TB retreatment outcomes.

In China, retreatment costs for participants in Arm C will be covered by study trial insurance. In South Africa, retreatment will be covered under the National Health Programme.

Additional key points to highlight include:

- The importance of providing a sputum sample at every single study visit. Sputum induction may be done if the participant is no longer producing sputum spontaneously.
- The importance of TB treatment adherence, both taking the medicine daily when supposed to and stopping the medicine when supposed to.
- The importance of returning to the research clinic anytime TB recurrence is suspected and NOT to the local (non-study) TB clinic.
- The importance of following up all the way through to the week 72 (month 18) final study visit.

6.3 Eligibility Criteria

There will be no exclusion based on gender or ethnicity. Children and pregnant or breast-feeding women will be excluded from this study because of PET/CT scanning. If someone becomes pregnant on study, this event will be reported to the IRB. This participant will continue to be followed on study but will be moved to Arm A. No further PET/CT scans will be performed while the participant is pregnant.

This study will not enroll prisoners. However, it is possible that a participant will be incarcerated after enrollment. If an enrolled individual is incarcerated and the study staff becomes aware of this incarceration, the treatment of TB for this participant will be turned over to the prison and the person will be withdrawn from the study. Incarcerations will be reported to the IRB. If a participant on study is incarcerated and released, and the study staff is not aware of this until after the person’s release from prison, this person can continue on study providing s/he does not meet other criteria for withdrawal.

Inclusion Criteria	Exclusion Criteria
Age 18 to 65 years with body weight from 35 to 90 kg	Extrapulmonary TB, including pleural TB
Has not been treated for active TB within the past 3 years and is not presently on TB treatment	Pregnant or desiring/trying to become pregnant in the next 6 months or breastfeeding
Not yet on TB treatment	HIV infected
Xpert positive for *M.tb*	Unable to take oral medications
Rifampin-sensitive pulmonary tuberculosis as indicated by Xpert	Diabetes as defined by point of care HbA1c above 6.5, random glucose over 200 mg/dL (or 11.1 mmol/L), fasting plasma glucose ≥126 mg/dL (or 7.0 mmol/L), or the presence of any anti-diabetic agent (including traditional medicines) as a concomitant medicine

Laboratory parameters within previous 14 days before enrollment:<br>a. Serum AST and ALT <3x upper limit of normal (ULN)<br>b. Creatinine <2x ULN<br>c. Hemoglobin >7.0 g/dL<br>d. Platelet count >50 x10^9 cells/L	Disease complications or concomitant illnesses that may compromise safety or interpretation of trial endpoints, such as known diagnosis of chronic inflammatory condition (e.g. sarcoidosis, rheumatoid arthritis, connective tissue disorder)
Able and willing to return for follow-up visits	Use of immunosuppressive medications, such as TNF-alpha inhibitors or systemic or inhaled corticosteroids, within the past 2 weeks
Able and willing to provide informed consent to participate in the study	Use of any investigational drug in the previous 3 months
Willing to undergo an HIV test	Substance or alcohol abuse that in the opinion of the investigator may interfere with the participant's adherence to study procedures
Willing to have samples, including DNA, stored	Any person for whom the physician feels this study is not appropriate
Willing to consistently practice a highly reliable, non-hormonal method of pregnancy prevention (e.g., condoms) during treatment if participant is a premenopausal female unless she has had a hysterectomy or bilateral tubal ligation or her male partner has had a vasectomy	

Note: Female participants are allowed to be on hormonal contraception but should be informed that hormonal contraception levels will be reduced by rifampin so should not be relied upon as the sole means of birth control. That is why they must agree to use a non-hormonal method of birth control (in addition) during the trial.

6.4 Screening

Screening will be performed after written informed consent is obtained. *In South Africa, SUN and TASK will also give a Genetic and Stored Samples Informed Consent before screening is performed.* The results of this screening will be recorded in the source documents before reporting the data on the appropriate CRFs.

It is recommended that a screening checklist that enumerates all samples, physiologic measurements, and medical record/clinical documentation, necessary to verify eligibility be utilized. This can be used at the end of the visit as a QC to ensure all study tests have been done. It is also recommended that determination of an available PET/CT slot be made. If there are no PET/CT slots available, it may not be possible to enroll a participant at that time.

The following procedures are to be conducted during screening. If there are documented labs available from within the last 2 weeks, they do not need to be repeated. Source documentation (see Sec. 9.1.1 for description of source documents) of the completed labs should be kept in the

participant binders. Sputum should still be collected even if already collected in the previous 2 weeks. If screening labs/procedures need to be repeated, they may be repeated and documentation of the repeated testing should occur in both the source documentation and the CRFs.

Suggested Screening Order:

1) A complete medical history and physical exam, including vital signs, are to be performed. Participants will be asked questions related to demographics. They will also be asked about their current and past medical history, including past or present illnesses, HIV status, alcohol, TB risk factors, any prior treatment for TB or latent tuberculosis infection. Participants will be asked if they are presently on any medications, if they have diabetes and if they are willing to have their samples stored.

   Note that the race descriptions in the demographics CRF are based on U.S. race descriptions.

   A review of the participant's concomitant medicines and medical history in the medical chart or by participant report will also be done to assess whether this participant meets study eligibility.

   Participants will be asked if they have any known allergies to medications including sensitivity or intolerance to any of the standard TB drugs.

2) Expectorated sputa samples for Xpert, smear and culture, will be collected. A minimum of 3 mL of sputum should be obtained. Xpert should be repeated at screening even if there is already an Xpert result from the local TB program so that we can obtain a baseline Xpert cycle threshold.

   Phenotypic DST is to be conducted from isolates grown using sputa samples collected during screening to confirm the molecular DST results. If there are mixed, conflicting, or inconsistent DST results, additional sputa may be utilized from any culture prior to conversion for additional DST testing.

3) A blood draw for complete blood count, AST, ALT, creatinine, and HbA1c for all participants and a serum pregnancy testing for all females will be done. Other safety blood tests may be done at the discretion of the study physician.

4) An HIV test will be done, unless participant self-reports HIV+, in which case s/he is ineligible.

Note that a CXR is not required for screening or during the study but may be done at any time for clinical care purposes at the discretion of the site physician and if Arm A fills faster than expected.

Maintenance of a screening log is required for all Predict Trial sites.

6.5 Enrollment

If eligibility is confirmed, the participant will only then be considered enrolled. This can occur with the Day 0 visit.

6.6 Scheduling

A scheduling tool provided to the sites may be used that will calculate dates of all study visits and visit windows based on the date of enrollment entered. The week 4 PET/CT scan date will be calculated based on the date of the baseline PET/CT scan date. All other visit dates, including the week 16 or 24 PET/CT scan dates, are scheduled based on the enrollment date.

6.7 Day 0/Baseline Visit

Test results should be discussed with the participants and they should be notified that they are eligible for the study if they have not already been told. Prior to the first dose of medication, tests according to the protocol should be performed and samples should be collected.

The following will be performed/obtained at the Day 0 visit prior to medication:

1) Vital Signs
2) Focused medical history/focused physical exam, if indicated
3) Sputum sample collection (in order of priority)
    a. Expectorated sputum (at least 3.5 mL) for smear and culture (solid and liquid) and for repeat Xpert if longer than 7 days from screening. This sputum may be overnight or home collected sputum brought in by the participant.
    b. Two or more expectorated or induced sputa (at least 4 mL) for biomarkers.
        i. At least 2.5 mL for storage raw. This does not need to be a fresh sputum.
        ii. At least 1.5 mL for storage as 1:1 mix with Trizol for MTB mRNA. This must be a fresh, spot sputum.
4) Blood will be collected for biomarkers
5) Urine will be collected for
    a. Biomarkers
    b. A pregnancy test, if indicated (performed before the scan on the same day of the scan); if within 2 days of the screening pregnancy test, this test may be skipped.
6) Saliva sample will be collected for biomarkers (unless done during PET/CT visit)
7) A baseline FDG-PET/CT scan should be scheduled to be done as soon as possible within 7 days of treatment initiation

GeneXpert does not need to be repeated at this visit if within 7 days of screening.

On the FDG-PET/CT scan day:

1) A finger prick will be done for glucose assessment
2) A urine pregnancy test will be done for all females unless within 2 days of the screening pregnancy test

3) Saliva sample will be collected for biomarkers (unless done during clinic visit)
4) FDG-PET/CT will be performed (if not pregnant or having high glucose)

To maximize sputum collection, the following are allowed at every visit:

- Home overnight sputum collection:
  - Sputum cups may be provided to the participant that day for overnight and morning collection, to be brought back the following day.
  - Sputum cups may be provided to the participant for collection of sputum the night before and morning of the following visit.
- Participant may return the following day for additional sputum collection.

At minimum, a sample of whatever the participant is able to produce should be sent for culture at every study visit. At any time during the study, if the participant is unable to provide sufficient sputa to perform all sputum-related testing, this will not be a protocol deviation.

At this and all subsequent visits, if a sputum culture is found to be contaminated or is otherwise unevaluable, the participant may be called back to provide another sputum sample.

TB treatment will be initiated on this visit. Dosage should be calculated per weight of the participant (as SOC) and the participant should be clearly instructed on how many pills should be taken each day. See section 5.2 for dosing guidelines.

The MERM device should be set and filled with enough drug to reach the next study visit plus the window period. For the baseline visit, this will be 10 days of treatment to reach the week 1 visit plus a 3-day window period. The maximum amount of drug needed at any one study visit will be 5 weeks to account for 4 weeks between visits plus a 1 week visit window. The number of pills to be taken each day should be written on the label inside the MERM box.

The MERM device should then be given to the participant, along with verbal instructions, practical MERM training, and the written MERM participant instruction sheet (see MERM Appendix A).

The PET/CT scan should be scheduled to occur as soon as possible but within 7 days of treatment initiation. On the day of the PET/CT scan, a urine pregnancy test for all females (unless within 2 days of the screening pregnancy test), fingerstick glucose, and a saliva sample will be collected (unless already collected on the day of the clinic visit).

The Week 4 PET/CT scan should be scheduled 4 weeks (-3 to +7 day window) after the baseline PET/CT scan date. Note that this is the only visit that is scheduled NOT according to the baseline enrollment date. All other visits, including the week 16 or 24 PET/CT scan, are scheduled according to the baseline enrollment date.

Arm A PET/CT Randomization: Between the baseline and week 4 visits, the baseline PET/CT scan will be read by designated PET/CT study readers and participants who do not meet the early treatment completion criteria will be identified and moved to Arm A. Site staff will be notified by

email. These Arm A participants will be randomized at week 12 to get their 3rd PET/CT scan at Week 16 or Week 24.

Each PET/CT scan will also be read clinically by local radiologists or nuclear medicine specialists and a clinical report provided to the study team. Unexpected findings should be discussed with the study participant and medical referral for follow-up evaluation should be done as needed.

6.8 Study Visits and Randomization

Weeks 1:

The following will be performed/obtained at the Week 1 visit:

1) Vital Signs
2) Focused medical history/focused physical exam, if indicated
3) One or more expectorated sputa samples (total sputum volume at least 3.5 mL) for smear and culture (liquid and solid)
4) Blood will be collected for biomarkers
5) Urine will be collected for biomarkers
6) Adherence monitoring

The MERM provider and participant questionnaire should be administered at Week 1 at the sites participating in this part of the study.

Week 2:

The following will be performed/obtained at the Week 2 visit:

1) Vital Signs
2) Focused medical history/focused physical exam, if indicated
3) Sputum sample collection (in order of priority)
    a. Expectorated sputum (total sputum volume at least 2 mL) for smear and culture (liquid and solid)
    b. Two or more expectorated or induced sputa (total sputum volume at least 4 mL) for biomarkers
        i. Storage raw
        ii. Storage as 1:1 mix with Trizol for MTB mRNA
4) Blood will be collected for biomarkers
5) Urine will be collected for biomarkers
6) Adherence monitoring

Sputum is collected from all participants at all visits throughout the duration of the study (through month 18). Sites should provide sputum cups to participants to bring home and instruct participants to collect overnight sputum and morning sputum on the day of their next visit. Sites are strongly encouraged (but not required) to collect induced sputa from participants no longer producing sputa spontaneously during the study. A sample of whatever the participant is able to produce should ALWAYS be collected. There should NEVER be a missing sputum sample simply because the participant is not producing sputum.

Notes about these weeks: CBC, chemistries, or LFTs are not routinely scheduled after screening but should be collected per SOC or any time there is any clinical indication of treatment adverse effects at the discretion of the study investigator. If abnormalities are noted, then that lab should continue to be followed to resolution. Adverse events should be reported according to MOP sec. 7.4.

Week 4:
The following will be performed/obtained at the Week 4 visit:

1) Vital Signs
2) Focused medical history/focused physical exam, if indicated
3) Sputum sample collection (in order of priority)
    a. Expectorated sputum (total sputum volume at least 3.5 mL) for smear and culture (liquid and solid) and Xpert
    b. Two or more expectorated or induced sputa (total sputum volume at least 4 mL) for biomarkers
        i. Storage raw
        ii. Storage as 1:1 mix with Trizol for MTB mRNA
4) Blood will be collected for biomarkers
5) Urine will be collected for biomarkers
6) Saliva sample will be collected for biomarkers (unless done during PET/CT visit)
7) Adherence monitoring
8) PET/CT scan (see below)

In addition to the regular study visit, the 2nd PET/CT scan should be done this week for all participants. (The protocol states for participants on Arms B and C and also participants on Arm A if resources allow but we expect sufficient resources for everyone at this time.) The window for the Week 4 PET/CT is 4 weeks after the baseline scan with a -3/+7 day window.
On the day of the PET/CT, prior to the scan, a urine pregnancy test for all females, fingerstick glucose, and saliva (if not done during clinic visit) will be taken.

Arm A PET/CT Randomization: Between weeks 4-8, the 2nd PET/CT scan will be read by designated study readers and participants who do not continue to meet the early treatment completion criteria will be identified and moved to Arm A. Site staff will be notified by email. Participants placed on Arm A at this time point will be randomized at week 12 to receive their 3rd PET/CT scan at Week 16 or Week 24.

Participants placed in Arm A because of the week 4 PET/CT scan are eligible for the PK substudy and should be approached to participate at their week 8 visit. An equal number of control participants who do not move to week 8 should also be approached to enroll. See section 8 for details.

Week 8:
The following will be performed/obtained at the Week 8 visit:

1) Vital Signs

2) Focused medical history/focused physical exam, if indicated
3) Sputum sample collection (in order of priority)
    a. Expectorated sputum (total sputum volume at least 3.5 mL) for smear and culture (liquid and solid) and Xpert
    b. Two or more expectorated or induced sputa (total sputum volume at least 4 mL) for biomarkers
        i. Storage raw
        ii. Storage as 1:1 mix with Trizol for MTB mRNA
    c. Consider ordering sputum DST if clinically indicated by a poor participant response to treatment
4) Blood will be collected for biomarkers
5) Urine will be collected for biomarkers
6) Adherence monitoring

At week 8, PZA and ethambutol will be discontinued for all participants, per SOC.

Protocol section 5.2 states that missed doses during the initial 8-week intensive phase will be added on to the end of the intensive phase, replacing continuation phase dosing. **This needs to be taken into account at this visit when drug tablets are dispensed into the MERM.**

Approach eligible participants to sign informed consent for the PK substudy (see Sec. 8 for details).

Week 12:
The following will be performed/obtained at the Week 12 visit:
1) Vital Signs
2) Focused medical history/focused physical exam, if indicated
3) One or more expectorated sputa samples (total sputum volume at least 2 mL) for smear and culture (liquid and solid)
4) Adherence monitoring
5) Possible PK substudy visit (see Sec. 8)

Arm A PET/CT Randomization: Arm A participants will be randomized at this Week 12 visit to receive a PET/CT scan at Week 16 or Week 24. Randomization will be done automatically within DataFax. Sites will be notified via email of the randomization assignment, after which the scans can be scheduled. Note that if there is a scheduling conflict with not enough available slots 4 weeks later, baseline and week 4 scans have first priority in scheduling, followed by week 16 or 24 scans.

Week 16: Note that depending on site logistics for Xpert testing, this visit may need to occur over more than 1 day during this week. In this case, the initial visit date and sample collection may occur earlier during the visit window. The 2nd visit for randomization may occur later during the visit window, especially for those participants with borderline adherence. Home overnight sputum sampling is allowed as long as it occurs within the visit window. Xpert testing at this visit should be reported IMMEDIATELY so that participants can get randomized.

The following will be performed/obtained on everyone at/during the Week 16 visit:

1) Vital Signs
2) Focused medical history/focused physical exam, if indicated
3) Sputum sample collection (in order of priority)
    a. Expectorated sputum (total sputum volume at least 3.5 mL) for smear and culture (liquid and solid) and Xpert
    b. Two or more expectorated or induced sputa (total sputum volume at least 4 mL) for biomarkers
        i. Storage raw
        ii. Storage as 1:1 mix with Trizol for MTB mRNA

An expectorated sputum may be used for smear and culture. The collection of an induced sputum is strongly encouraged at this point for sputum biomarkers assessment due to the volume needed.

4) Blood will be collected for biomarkers
5) Urine will be collected for biomarkers
6) Saliva sample will be collected for biomarkers
7) Adherence monitoring
8) Possible PK substudy visit (see Sec. 8)

The following will be done AFTER RANDOMIZATION into Arm B or C (and subsequent randomization to Week 16 or 24 PET/CT scan for those in Arm A:

9) FDG-PET/CT scan *for those randomized to Arm B or C and Arm A participants randomized to receive their 3rd PET/CT scan at* Week 16 (scan should be within +14 days, but ideally within +7 days of randomization)

For those receiving a PET/CT scan, the following will be performed/obtained on the day of the scan:

1) A finger stick will be collected for blood glucose levels prior to PET/CT scan
2) Urine for pregnancy test for all females prior to scan
3) Saliva sample (unless done during the clinic visit)

Arms B/C Randomization: Participants who meet all early treatment completion criteria (baseline PET/CT scan, week 4 PET/CT scan, week 16 Xpert MTB/RIF cycle threshold, and week 16 adherence) are randomized to either Arm B (continue treatment to week 24) or Arm C (stop treatment at week 16). Randomization will occur by site. The date of randomization should be scheduled within the visit window of +/- 7 days.

Randomization will occur through DataFax. To perform randomization, study staff will need to enter the results of the Week 16 Xpert MTB/RIF cycle threshold and adherence dose counts directly into DataFax electronically. All other early treatment completion criteria will already be populated in DataFax. After the Xpert and adherence data have been entered, the system will generate a page telling the staff whether or not this person is eligible for randomization. The

arm assignment will be displayed on the screen and the staff should print and file this in the participant's records.

If the internet is inaccessible when randomization is needed, a telephone backup can be used. In Cape Town, Shera Weyers [+27 (0)83 994 6963] from the TASK data management team has been designated as the primary backup contact and can be called. In Henan, Gao Jingcai [+86 15838015837] is the primary backup contact. In this event, the site will need to fax to TASK or Gao Jingcai their Xpert cycle threshold result and dose count number. The backup person will then log in to DataFax to input these data for the site and fax back to the site the randomization result.

Once an arm has been assigned, the participant should be told if s/he will stop drugs or continue on drugs to week 24. Participants who meet the early treatment completion criteria may be randomized within the visit window as long as they meet or exceed the minimum required 100 drug doses. Participants who are close to the minimum 100 doses may be scheduled later during the window period if this allows them to meet the 100 required doses.

Arm C participants:

- Please remind them when they complete treatment that:
  - They should not continue treatment elsewhere because their treatment is now complete.
  - At any sign of illness during follow-up, **they should contact the research site for evaluation and NOT go to their local clinic.** It is imperative that we capture all relapses.
- The end of treatment MERM questionnaire should be administered at the sites participating in this part of the study.

Arm A PET/CT scan Randomization: Participants who do not meet all early treatment completion criteria and are placed on Arm A at Week 16 will be randomized to receive a PET/CT scan at Week 16 or Week 24. This randomization will be stratified by site and will be done within DataFax. When DataFax determines that a participant needs to be moved to Arm A, it will automatically also do the randomization and inform the site as to the result.

Week 20:

The following will be performed/obtained at the Week 20 visit:

1) Vital Signs
2) Focused medical history/focused physical exam, if indicated
3) One or more expectorated sputa samples (total sputum volume at least 2 mL) for smear and culture (liquid and solid)
4) Adherence monitoring (Arms A and B only)
5) Blood will be collected in all participants with plasma batch tested for isoniazid and/or rifampin levels to confirm whether or not participants are still taking TB drugs.

Arm C participants should be questioned to confirm that they truly stopped TB treatment and are not surreptitiously still taking treatment. Even with this questioning, plasma will still be tested for drug levels, as noted above.

PET/CT scan should be scheduled for Arm A participants randomized to receive their 3rd PET/CT scan at Week 24. Note that if there is a scheduling conflict with not enough available slots 4 weeks later, baseline and week 4 scans have first priority in scheduling, followed by week 16 or 24 scans.

Week 24:

The following will be performed/obtained at the Week 24 visit:

1) Vital Signs
2) Focused medical history/focused physical exam, if indicated
3) Sputum sample collection (in order of priority)
    a. Expectorated sputum (total sputum volume at least 3.5 mL) for smear and culture (liquid and solid) and Xpert
    b. Two or more expectorated or induced sputa (total sputum volume at least 4 mL) for biomarkers
        i. Storage raw
        ii. Storage as 1:1 mix with Trizol for MTB mRNA

Please note that the collection of an induced sputum is strongly encouraged at this point to enable sufficient sputum volume for biomarkers analysis.

4) Blood will be collected for biomarkers
5) Urine will be collected for biomarkers
6) Saliva sample will be collected for biomarkers
7) Adherence monitoring (Arms A and B only)

FDG-PET/CT scan will be done *for those on Arm A randomized to receive the scan at this time point* (scan should be within +14 days, but ideally within +7 days from stopping drug) For those receiving a PET/CT scan, the following will be performed/obtained on the day of the scan:

1) A finger stick will be collected for blood glucose levels prior to PET/CT scan
2) Urine for pregnancy test for all females prior to scan
3) Saliva sample (unless done during the clinic visit)

Participants in Arms A and B will complete treatment at Week 24. If an Arm A participant is sputum culture positive at Week 16, s/he may be treated for longer than 24 weeks at the discretion of the treating physician. Note that Arm B participants will be allowed complete at least 150 doses of treatment even if this exceeds the week 24 visit window.

Sites participating in the MERM questionnaire part of the study should administer the 2nd questionnaire to providers and Arms A and B participants.

Weeks 36-72 (General notes, week visits are below):

Participants should be encouraged to return to the study site clinic for an unscheduled sick visit any time they are concerned that they may be developing recurrent TB.

If a participant had previously converted his/her sputa to culture negative, now has new symptoms consistent with recurrent TB, and the sputum Xpert MTB/RIF is positive, then the participant should be considered a TB recurrence suspect. Samples and testing should be followed as per the recurrence visit noted below. Any time that someone is suspected to have recurred, please collect as many sputa samples as possible across more than 1 day to increase the chances of a positive culture. **Two separate positive sputum cultures on separate days are required per protocol (sec. 6.2.3) to confirm TB recurrence.** A PET/CT scan should be scheduled and the participant should be referred to the local TB clinic for consideration to restart TB therapy. This participant will be considered a confirmed TB recurrence if the sputum culture becomes positive for *M.tb* that is confirmed on a 2nd subsequent culture on solid or liquid medium.

Week 36:
The following will be performed/obtained at the Week 36 visit:

1) Vital Signs
2) Focused medical history/focused physical exam, if indicated
3) One or more expectorated sputa samples (total sputum volume at least 2 mL) for smear and culture (liquid and solid)

Week 48:
The following will be performed/obtained at the Week 48 visit:

1) Vital Signs
2) Focused medical history/focused physical exam, if indicated
3) Sputum sample collection (in order of priority)
    a. Expectorated sputum (total sputum volume at least 2 mL) for smear and culture (liquid and solid)
    b. Two or more expectorated or induced sputa (total sputum volume at least 4 mL) for biomarkers
        i. Storage raw
        ii. Storage as 1:1 mix with Trizol for MTB mRNA

Please note that the collection of an induced sputum is strongly encouraged at this point to enable sufficient sputum volume for biomarkers analysis.

4) Blood will be collected for biomarkers
5) Urine will be collected for biomarkers

Week 60 follow-up phone call: Participants should be called and asked the following questions:

Since your last visit, have you experienced any of the following symptoms?

- Persistent cough
- Hemoptysis (coughing up blood)

- Unexplained weight loss
- Unexplained fevers, chills, or night sweats
- Persistent shortness of breath
- Unexplained fatigue
- Chest pain

If the participant answers yes to any of the above, s/he should be asked to come in for a full study visit. At that visit, the participant should be evaluated similar to the week 48 visit. If TB recurrence is suspected based on the history, physical exam, and laboratory results, the participant should be called to return for a TB recurrence study visit, as described below.

Week 72:
The following will be performed/obtained at the Week 72 visit:

1) Vital Signs
2) Focused medical history/focused physical exam, if indicated
3) Sputum sample collection (in order of priority)
    a. Expectorated sputum (total sputum volume at least 3.5 mL) for smear and culture (liquid and solid)
    b. Two or more expectorated or induced sputa (total sputum volume at least 4 mL) for biomarkers
        i. Storage raw
        ii. Storage as 1:1 mix with Trizol for MTB mRNA

Please note that the collection of an induced sputum is strongly encouraged at this point to enable sufficient sputum volume for biomarkers analysis.

4) Blood will be collected for biomarkers
5) Urine will be collected for biomarkers

Participants with a positive culture at this visit may be asked to return for sputum culture confirmation. In the event that the participant is unable to return for follow-up, every effort will be made to contact the participant by telephone to determine his/her clinical status. If possible, health authorities local to where the patient is located may be asked to collect a sputum sample for culture.

TB Recurrence: Note that participants should be reminded at all study visits after stopping treatment that they should return to the study clinic for evaluation any time s/he suspects TB recurrence. **S/he should NOT go to the local TB clinic for evaluation.**

As listed in protocol section 4.17, the following will be performed/obtained in the event that someone on study has a suspected recurrence of TB:

1) Vital Signs
2) Focused medical history/focused physical exam, if indicated
3) One or more sputa samples (at least 3.5 mL) for Xpert testing, smear and culture (liquid and solid)

4) Two or more sputa samples or induced sputum (total sputum volume at least 4 mL) for biomarkers
    a. Storage raw and
    b. Storage as 1:1 mix with Trizol for MTB mRNA
5) Blood draw for
    a. Complete blood count, chemistries (including HbA1c), and liver function tests
    b. HIV test
    c. Biomarkers
6) Urine will be collected for biomarkers
7) FDG-PET/CT scan will be scheduled for as soon as possible

The following will be performed/obtained on the day of the PET/CTscan:

1) A finger stick will be collected for blood glucose levels prior to PET/CT scan
2) Urine for pregnancy test for all females prior to scan
3) Fasting saliva sample

National/local TB records may be reviewed for unreported TB recurrence and for TB retreatment outcomes. Although Xpert MTB/RIF should be done, a positive Xpert MTB/RIF may identify dead bacteria and should not, by itself, be the reason to restart treatment.

As defined in protocol section 6.2.3, participants are not confirmed as treatment recurrences until two sputa cultures on solid medium are positive for *Mtb* on different days. If TB recurrence is confirmed, any remaining TB recurrence study procedures (protocol section 4.17) not already done as part of the TB recurrence evaluation should then be done (e.g. sputum for biomarkers, blood for biomarkers, and urine for biomarkers), including scheduling of the PET/CT scan. Participants should be referred to the local TB clinic for retreatment if not started already.

Please note that the primary endpoint for this study is defined in the protocol as culture results on solid medium. MGIT results are secondary endpoints. Thus site clinical staff should refrain from making treatment decisions based solely on MGIT results and, as much as possible, await LJ culture results for primary decision-making. Of course, participant safety is paramount so if a participant has already culture converted and a MGIT result returns unexpectedly positive, the participant may be clinically assessed (by phone or in person) for clinical symptoms before the LJ results are back. **The decision of whether or not to restart TB treatment is a clinical decision based on all available data to that point.** If TB treatment is restarted for strong clinical suspicion and participant safety before recurrence is confirmed by the study, **every effort should be made to collect multiple sputa samples over more than 1 day before restarting treatment**. Retreatment is not provided as a part of this protocol and participants should be referred to the local TB clinic for retreatment.

Confirmed TB recurrence participants from Arm C will continue to be followed observationally to the end of retreatment for retreatment outcomes even if this extends follow-up beyond 18 months. Data captured should include treatment regimen, treatment duration, and any laboratory and microbiology data that are collected, including sputum smear and cultures. Final retreatment outcome at the end of retreatment should be recorded.

TB recurrence is the only time during the trial (after screening) when HIV testing is repeated. If a participant is identified to be HIV+ during the trial via outside testing and informs the study staff, how to handle this participant will be decided on a case-by-case basis through a discussion with NIH staff and study PIs and will depend in part on whether or not the participant has already been randomized.

6.9 Management of Participants Who Are Non-Adherent to Medications

Adherence counseling must be done at every study visit during the treatment phase. Participants who consistently miss treatment doses by self-report, dose count, or MERM monitoring will need more intense adherence counseling. Clinic staff should take the time to understand the participant's social and economic situation as well as his/her perspective, knowledge, and understanding of TB disease and TB stigma. This will form the basis to understand the reasons why the participant is missing doses. Proper functioning of the MERM container and its daily reminder feature should be confirmed. Suggestions for how to improve adherence should be provided and may include things like reviewing the importance of treatment adherence, assistance in developing a treatment routine, additional options for how to take the medications, or establishing formal or informal directly observed therapy by a family member, friend, or local healthcare/outreach worker. Any adverse effects experienced by the participant should be addressed. A new treatment plan should be developed in conjunction with the participant.

The study status of participants who miss doses will be managed according to protocol sec. 5.2.

6.10 Management of Participants With Poor Treatment Response

Participants who are not improving clinically (decreasing symptoms, increasing weight) by week 4 and especially by week 8 will have their baseline DST information reviewed to confirm sensitivity to first-line drugs and drug adherence records reviewed. Participants who are not fully adherent to treatment should be managed per MOP sec. 6.9. If clinical or microbiological improvement is not noted by week 8 despite good adherence, sputa should be sent for a repeat DST. Any participant identified to have resistance to any first-line drug at any time during treatment will be withdrawn from the study and replaced (protocol sec. 5.9).
Participants in Arms B or C who are subsequently found to be week 16 sputum culture positive for *M.tb* that is confirmed on a subsequent culture will be considered to have treatment failure, will be taken off study as having met a study endpoint, and will be referred for TB re-treatment. Participants in Arm A who are still sputum culture positive for *M.tb* at week 24 will be considered to have treatment failure, will be taken off study as having reached a study endpoint, and will be referred to continue TB treatment (protocol sec. 6.2.2).

6.11 Contraindicated and Concomitant Medications

Rifampin is a strong inducer of the hepatic cytochrome P450 enzyme system, including isoenzymes CYP2B6, CYP2C8, CYP2C9, CYP2C19, CYP3A4, CYP3A5, and CYP3A7. Rifampin will increase the metabolism of drugs metabolized by these enzymes rendering them less effective or ineffective.

Rifampin has been reported to substantially decrease the plasma concentrations of the following antiviral drugs: atazanavir, darunavir, fosamprenavir, saquinavir, and tipranavir. These antiviral drugs must not be co-administered with rifampin.

Rifampin has been reported to accelerate the metabolism of the following drugs: anticonvulsants (e.g., phenytoin), digitoxin, antiarrhythmics (e.g., disopyramide, mexiletine, quinidine, tocainide), oral anticoagulants, antifungals (e.g., fluconazole, itraconazole, ketoconazole), barbiturates, beta-blockers, calcium channel blockers (e.g., diltiazem, nifedipine, verapamil), chloramphenicol, clarithromycin, corticosteroids, cyclosporine, cardiac glycoside preparations, clofibrate, oral or other systemic hormonal contraceptives, dapsone, diazepam, doxycycline, fluoroquinolones (e.g., ciprofloxacin), haloperidol, oral hypoglycemic agents (sulfonylureas), levothyroxine, methadone, narcotic analgesics, progestins, quinine, tacrolimus, theophylline, tricyclic antidepressants (e.g., amitriptyline, nortriptyline) and zidovudine. It may be necessary to adjust the dosages of these drugs if they are given concurrently with rifampin. Patients using oral or other systemic hormonal contraceptives should be advised to change to nonhormonal methods of birth control during rifampin therapy.
Rifampin has been observed to increase the requirements for anticoagulant drugs of the coumarin type. In patients receiving anticoagulants and rifampin concurrently, it is recommended that the prothrombin time be performed daily or as frequently as necessary to establish and maintain the required dose of anticoagulant.
Rifampin levels may also be affected by a concomitantly dosed medication. When the two drugs were taken concomitantly, decreased concentrations of atovaquone and increased concentrations of rifampin were observed. Concurrent use of ketoconazole and rifampin has resulted in decreased serum concentrations of both drugs. Concurrent use of rifampin and enalapril has resulted in decreased concentrations of enalaprilat, the active metabolite of enalapril. Dosage adjustments should be made if indicated by the patient's clinical condition. Concomitant antacid administration may reduce the absorption of rifampin. Daily doses of rifampin should be given at least 1 hour before the ingestion of antacids. Probenecid and cotrimoxazole have been reported to increase the blood level of rifampin. When rifampin is given concomitantly with either halothane or isoniazid, the potential for hepatotoxicity is increased. The concomitant use of rifampin and halothane should be avoided. Patients receiving both rifampin and isoniazid should be monitored close for hepatotoxicity.
Plasma concentrations of sulfapyridine may be reduced following the concomitant administration of sulfasalazine and rifampin. This finding may be the result of alteration in the colonic bacteria responsible for the reduction of sulfasalazine to sulfapyridine and mesalamine. Because rifampin is supplied as a part of a fixed drug combination for this trial, adjusting the dose of rifampin alone is not possible. Either the dose of the concomitant medication will need to be adjusted accordingly or this participant will not be able to take the fixed dose combination pills and therefore is not eligible to enroll in this trial (protocol sec. 3.2.2 exclusion criterion #6).

6.12 Logs, Flows, and Worksheets

6.12.1 SCREENING VISIT FLOW

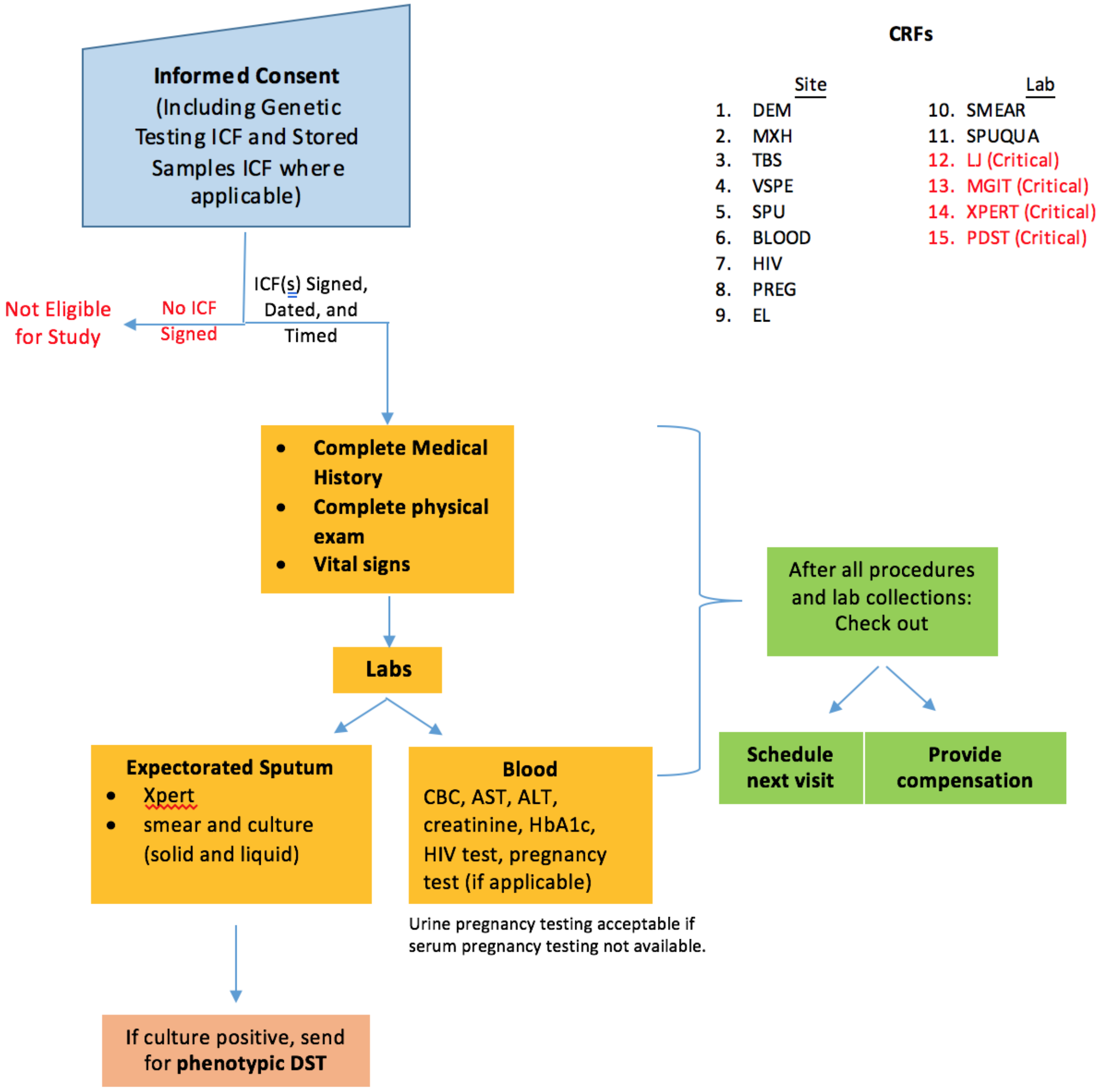

6.12.2 **ENROLLMENT/BASELINE/DAY 0 VISIT FLOW**

- Review Lab Results with Participant
- Review Incl/Excl with participant

If participant meets all criteria, consider the participant enrolled.

Perform

- Focused interval medical history (if indicated)
- Focused physical exam (if indicated)
- Vital signs

CRFs

Site	Lab
1. ADHERE	8. SMEAR
2. PETSAL	9. SPUQUA
3. SPU	10. LJ (Critical)
4. BLDSTR	11. MGIT (Critical)
5. AE	12. XPERT (Critical)
6. CONMED	13. SPUSTR 1
View	14. SPUSTR 2
7. EARLY0	15. SALSTR (SUN)

Labs

Sputum (in order of priority)

- Expectorated sputum for smear and culture (solid and liquid)
- Expectorated or induced sputum for biomarkers
- If >7 days since screening labs: Xpert

Blood

- Biomarkers

Additional clinical labs CBC, chemistries, LFTs, etc ***are not study required*** *but may be done as clinically indicated*

Urine

- Biomarkers
- Pregnancy test (with PET/CT)

Note that sputum samples are required to be collected at every visit throughout the study. Every effort should be made to obtain the required number of sputum samples. Sites should provide sputum cups to participants to bring home and instruct participants to collect overnight sputum and morning sputum on the day of their next visit. If the participant is unable to produce sputum (especially during the later months of the study), an induced sputum is encouraged if possible but not required. If induction is not done, the participant should be instructed to cough vigorously and clear the back of his/her throat to produce an adequate volume of saliva for testing. A sample of sputum or saliva should always be collected at every visit.

If culture positive, send for **phenotypic DST**

- **Initiate HRZE treatment by weight**
- **Adherence education and counseling**
- **MERM setup and education (if used)**

Schedule baseline PET/CT as soon as possible within 7 days, including finger stick for glucose and urine pregnancy test

Saliva sample at end of visit or at PET/CT center

Schedule week 1 visit (+/- 3 day window)

Schedule week 4 PET/CT scan (-3/+7 day window from baseline PET/CT scan date)

Provide compensation

6.12.3 WEEK 1 VISIT FLOW (+/- 3 day window)

CRFs

Site
1. ADHERE (Critical)
2. SPU
3. BLDSTR
4. AE
5. CONMED

Lab
6. SMEAR
7. SPUQUA
8. LJ (Critical)
9. MGIT (Critical)
10. XPERT (Critical)
11. SPUSTR 1
12. SPUSTR 2

Perform
- Focused interval medical history (if indicated)
- Focused physical exam (if indicated)
- Vital signs

Labs

Sputum
- Expectorated sputum for smear and culture (solid and liquid)

Blood
- Biomarkers

Additional clinical labs CBC, chemistries, LFTs, etc ***are not study required*** *but may be done as clinically indicated*

Urine
- Biomarkers

Note that sputum samples are required to be collected at every visit throughout the study. Every effort should be made to obtain the required number of sputum samples. Sites should provide sputum cups to participants to bring home and instruct participants to collect overnight sputum and morning sputum on the day of their next visit. If the participant is unable to produce sputum (especially during the later months of the study), an induced sputum is encouraged if possible but not required. If induction is not done, the participant should be instructed to cough vigorously and clear the back of his/her throat to produce an adequate volume of saliva for testing. A sample of sputum or saliva should always be collected at every visit.

- **Pill Count**
- **Adherence education and counseling**
- **MERM**
  - **Download data and upload to study database**
  - **Refill**
  - **Complete provider and participant questionnaires (some sites)**

Schedule week 2 visit (+/- 3 day window)

Provide compensation

6.12.4 **WEEK 2 VISIT (+/- 3 day window)**

Perform
- Focused interval medical history (if indicated)
- Focused physical exam (if indicated)
- Vital signs

CRFs

Site
1. ADHERE (Critical)
2. SPU
3. BLDSTR
4. AE
5. CONMED

Lab
6. SMEAR
7. SPUQUA
8. LJ (Critical)
9. MGIT (Critical)
10. SPUSTR 1
11. SPUSTR 2

Labs

Sputum (in order of priority)
- Expectorated sputum for smear and culture (solid and liquid)
- Expectorated or induced sputum for biomarkers

Blood
- Biomarkers

Additional clinical labs CBC, chemistries, LFTs, etc ***are not study required*** *but may be done as clinically indicated*

Urine
- Biomarkers

Note that sputum samples are required to be collected at every visit throughout the study. Every effort should be made to obtain the required number of sputum samples. Sites should provide sputum cups to participants to bring home and instruct participants to collect overnight sputum and morning sputum on the day of their next visit. If the participant is unable to produce sputum (especially during the later months of the study), an induced sputum is encouraged if possible but not required. If induction is not done, the participant should be instructed to cough vigorously and clear the back of his/her throat to produce an adequate volume of saliva for testing. A sample of sputum or saliva should always be collected at every visit.

- **Pill Count**
- **Adherence education and counseling**
- **MERM**
  - **Download data and upload to study database**
  - **Refill**

Schedule week 4 visit (+/- 7 day window)

Provide compensation

6.12.5 WEEK 4 VISIT FLOW (+/- 7 day window)

CRFs

Site
1. ADHERE (Critical)
2. PETSAL
3. SPU
4. BLDSTR
5. AE
6. CONMED

View
7. EARLY4

Lab
8. SMEAR
9. SPUQUA
10. LI (Critical)
11. MGIT (Critical)
12. XPERT (Critical)
13. SPUSTR 1
14. SPUSTR 2
15. SALSTR (SUN)

Perform
- Focused interval medical history (if indicated)
- Focused physical exam (if indicated)
- Vital signs

Labs

Sputum (in order of priority)
- Expectorated sputum for smear and culture (solid and liquid)
- Expectorated sputum for Xpert
- Expectorated or induced sputum for biomarkers

Blood
- Biomarkers

*Additional clinical labs CBC, chemistries, LFTs, etc **are not study required** but may be done as clinically indicated*

Urine
- Biomarkers
- Pregnancy test (BEFORE PET/CT scan), if applicable

Note that sputum samples are required to be collected at every visit throughout the study. Every effort should be made to obtain the required number of sputum samples. Sites should provide sputum cups to participants to bring home and instruct participants to collect overnight sputum and morning sputum on the day of their next visit. If the participant is unable to produce sputum (especially during the later months of the study), an induced sputum is encouraged if possible but not required. If induction is not done, the participant should be instructed to cough vigorously and clear the back of his/her throat to produce an adequate volume of saliva for testing. A sample of sputum or saliva should always be collected at every visit.

- **Pill Count**
- **Adherence education and counseling**
- **MERM**
  - **Download data and upload to study database**
  - **Refill**

Ensure participant has 2nd PET/CT scan (4 weeks after baseline scan with -3/+7 day window), including finger stick glucose and urine pregnancy test

Saliva sample at end of visit or at PET/CT center

Schedule week 8 visit (+/- 7 day window)

Provide compensation

6.12.6 WEEK 8 VISIT FLOW (+/- 7 day window)

CRFs

Site		Lab	
1.	VSPE	8.	SMEAR
2.	TBS	9.	SPUQUA
3.	ADHERE (Critical)	10.	LI (Critical)
4.	SPU	11.	MGIT (Critical)
5.	BLDSTR	12.	XPERT (Critical)
6.	AE	13.	SPUSTR 1
7.	CONMED	14.	SPUSTR 2

Perform
- Focused interval medical history (if indicated)
- Focused physical exam (if indicated)
- Vital signs

Labs

Sputum (in order of priority)
- Expectorated sputum for smear and culture (solid and liquid)
- Expectorated sputum for Xpert
- Expectorated or induced sputum for biomarkers
- Consider ordering sputum DST if clinically indicated by poor response to treatment

Blood
- Biomarkers

Additional clinical labs CBC, chemistries, LFTs, etc ***are not study required*** *but may be done as clinically indicated*

Urine
- Biomarkers

Note that sputum samples are required to be collected at every visit throughout the study. Every effort should be made to obtain the required number of sputum samples. Sites should provide sputum cups to participants to bring home and instruct participants to collect overnight sputum and morning sputum on the day of their next visit. If the participant is unable to produce sputum (especially during the later months of the study), an induced sputum is encouraged if possible but not required. If induction is not done, the participant should be instructed to cough vigorously and clear the back of his/her throat to produce an adequate volume of saliva for testing. A sample of sputum or saliva should always be collected at every visit.

- **Pill Count**
- **Adherence education and counseling**
- **MERM**
  - **Download data and upload to study database**
  - **Refill**

Make up any HRZE doses missed during intensive phase, then discontinue PZA and ETH per SOC

Schedule week 12 visit (+/- 7 day window)

Informed consent for eligible PK substudy participants

Provide compensation

6.12.7 **WEEK 12 VISIT FLOW (+/- 7 day window)**

Perform
- Focused interval medical history (if indicated)
- Focused physical exam (if indicated)
- Vital signs

CRFs

Site
1. ADHERE (Critical)
2. SPU
3. SUB (PK)
4. AE
5. CONMED

View
6. Arm A Randomization at Week 12

Lab
7. SMEAR
8. SPUQUA
9. LJ (Critical)
10. MGIT (Critical)

Labs

Sputum
- Expectorated sputum for smear and culture (solid and liquid)

Note that sputum samples are required to be collected at every visit throughout the study. Every effort should be made to obtain the required number of sputum samples. Sites should provide sputum cups to participants to bring home and instruct participants to collect overnight sputum and morning sputum on the day of their next visit. If the participant is unable to produce sputum (especially during the later months of the study), an induced sputum is encouraged if possible but not required. If induction is not done, the participant should be instructed to cough vigorously and clear the back of his/her throat to produce an adequate volume of saliva for testing. A sample of sputum or saliva should always be collected at every visit.

- **Pill Count**
- **Adherence education and counseling**
- **MERM**
  - **Download data and upload to study database**
  - **Refill**

Arm A randomization
- **3rd PET/CT scan at Week 16**
  - **Schedule Week 16 PET/CT scan (+14d window)**
- **3rd PET/CT scan at Week 24**

Schedule week 16 visit; may occur over >1 day within +/- 7 day window

Possible PK substudy visit

Provide compensation

6.12.8 WEEK 16 VISIT FLOW (+/- 7 day window)

CRFs

Site

1. VSPE
2. TBS
3. ADHERE (Critical)
4. PETSAL
5. SPU
6. BLDSTR
7. SUB (PK)
8. AE
9. CONMED
10. EARLY16DOSE (eDC)
11. EARLY16XPERT (eDC)
12. RAND16 (eDC)

Lab

13. SMEAR
14. SPUQUA
15. LJ (Critical)
16. MGIT (Critical)
17. SPUSTR 1
18. SPUSTR 2
19. SALSTR (SUN)

Perform

- Focused interval medical history (if indicated)
- Focused physical exam (if indicated)
- Vital signs

Labs

Sputum (in order of priority)

- Expectorated sputum for Xpert
- Expectorated sputum for smear and culture (solid and liquid)
- Induced sputum for biomarkers

Blood

- Biomarkers

Additional clinical labs CBC, chemistries, LFTs, etc ***are not study required*** *but may be done as clinically indicated*

Urine

- Biomarkers
- Pregnancy testing, if applicable, for participants receiving PET/CT scans only

Note that sputum samples are required to be collected at every visit throughout the study. Every effort should be made to obtain the required number of sputum samples. Sites should provide sputum cups to participants to bring home and instruct participants to collect overnight sputum and morning sputum on the day of their next visit. If the participant is unable to produce sputum (especially during the later months of the study), an induced sputum is encouraged if possible but not required. If induction is not done, the participant should be instructed to cough vigorously and clear the back of his/her throat to produce an adequate volume of saliva for testing. A sample of sputum or saliva should always be collected at every visit. **At this visit, expectorated sputum should be collected for culture. Induced sputum should be collected for biomarkers.**

- **Pill Count**
- **Adherence education and counseling**
- **MERM**
  - **Download data and upload to study database**
  - **Refill if continuing treatment**
  - **Arm C: complete MERM questionnaire**

Schedule week 20 visit (+/- 7 day window)

Provide compensation

Possible PK substudy visit

Arm B/C randomization

Arm B: Continue treatment to Week 24

Arm C: Stop treatment now at Week 16

Arm A randomization

3rd PET/CT scan at Week 16

3rd PET/CT scan at Week 24

Week 16 PET/CT scan; window is +14d but ideally schedule within 7d, including **finger stick glucose and pregnancy test**

Saliva sample at end of visit or at PET/CT center

6.12.9 **WEEK 20 VISIT FLOW (+/- 7 day window)**

Perform

- Focused interval medical history (if indicated)
- Focused physical exam (if indicated)
- Vital signs

CRFs

Site

1. ADHERE for A and B (Critical)
2. SPU
3. AE
4. CONMED

Lab

5. SMEAR
6. SPUQUA
7. LI (Critical)
8. MGIT (Critical)

Data Import

9. Drug level (will be imported in batch)

Labs

Sputum

- Expectorated sputum for smear and culture (solid and liquid)

Blood

- DRUG MONITORING

*Additional clinical labs CBC, chemistries, LFTs, etc **are not study required** but may be done as clinically indicated*

Note that sputum samples are required to be collected at every visit throughout the study. Every effort should be made to obtain the required number of sputum samples. Sites should provide sputum cups to participants to bring home and instruct participants to collect overnight sputum and morning sputum on the day of their next visit. If the participant is unable to produce sputum (especially during the later months of the study), an induced sputum is encouraged if possible but not required. If induction is not done, the participant should be instructed to cough vigorously and clear the back of his/her throat to produce an adequate volume of saliva for testing. A sample of sputum or saliva should always be collected at every visit.

FOR ARMS A AND B ONLY:

- **Pill Count**
- **Adherence education and counseling**
- **MERM**
  - **Download data and upload to study database**
  - **Refill**

Schedule week 24 visit (+/- 7 day window)

Schedule week 24 PET/CT scan for Arm A participants randomized to this time point

Provide compensation

6.12.10 WEEK 24 VISIT FLOW (+/- 7 day window)

CRFs

Site	Lab
1. VSPE	9. SMEAR
2. TBS	10. SPUQUA
3. ADHERE Arm A and B (Critical)	11. LI (Critical)
4. PETSAL Arm A	12. MGIT (Critical)
5. SPU	13. XPERT (Critical)
6. BLDSTR	14. SPUSTR 1
7. AE	15. SPUSTR 2
8. CONMED	16. SALSTR (SUN)

Perform

- Focused interval medical history (if indicated)
- Focused physical exam (if indicated)
- Vital signs

Labs

Sputum (in order of priority)

- Expectorated sputum for smear and culture (solid and liquid)
- Expectorated sputum for Xpert
- Induced sputum for biomarkers

Blood

- Biomarkers

Additional clinical labs CBC, chemistries, LFTs, etc ***are not study required*** *but may be done as clinically indicated*

Urine

- Biomarkers
- Pregnancy testing, if applicable, for Arm A only

Note that sputum samples are required to be collected at every visit throughout the study. Every effort should be made to obtain the required number of sputum samples. Sites should provide sputum cups to participants to bring home and instruct participants to collect overnight sputum and morning sputum on the day of their next visit. If the participant is unable to produce sputum (especially during the later months of the study), an induced sputum is encouraged if possible but not required. If induction is not done, the participant should be instructed to cough vigorously and clear the back of his/her throat to produce an adequate volume of saliva for testing. A sample of sputum or saliva should always be collected at every visit. **At this visit, expectorated sputum should be collected for culture. Induced sputum should be collected for biomarkers.**

ARMs A AND B ONLY

- **Pill Count**
- **Adherence education and counseling**
- **MERM**
    - **Download data and upload to study database**
    - **Refill only for those in Arm A still culture positive at Week 16 and whose treating physician wants to continue treating**
- **Arm B: stop treatment**

PET/CT for those in ARM A RANDOMIZED to receive scan (+14d window), including finger stick glucose and pregnancy test

Saliva sample at end of visit or at PET/CT center

Schedule week 36 visit (+/- 30 day window, but as close to actual date as possible)

Provide compensation

6.12.11 WEEK 36 VISIT FLOW (+/- 30 day window)

CRFs

Site		Lab	
1.	VSPE	6.	SMEAR
2.	TBS	7.	SPUQUA
3.	SPU	8.	LJ (Critical)
4.	AE	9.	MGIT (Critical)
5.	CONMED		

Perform

- Focused interval medical history (if indicated)
- Focused physical exam (if indicated)
- Vital signs

Labs

Sputum

- Expectorated sputum for smear and culture (solid and liquid)

Note that sputum samples are required to be collected at every visit throughout the study. Every effort should be made to obtain the required number of sputum samples. Sites should provide sputum cups to participants to bring home and instruct participants to collect overnight sputum and morning sputum on the day of their next visit. If the participant is unable to produce sputum (especially during the later months of the study), an induced sputum is encouraged if possible but not required. If induction is not done, the participant should be instructed to cough vigorously and clear the back of his/her throat to produce an adequate volume of saliva for testing. A sample of sputum or saliva should always be collected at every visit.

Schedule week 48 visit (+/- 30 day window, but as close to actual date as possible)

Provide compensation

6.12.12 WEEK 48 VISIT FLOW (+/- 30 day window)

Perform
- Focused interval medical history (if indicated)
- Focused physical exam (if indicated)
- Vital signs

CRFs

Site		Lab	
1.	VSPE	7.	SMEAR
2.	TBS	8.	SPUQUA
3.	SPU	9.	LJ (Critical)
4.	BLDSTR	10.	MGIT (Critical)
5.	AE	11.	SPUSTR 1
6.	CONMED	12.	SPUSTR 2

Labs

Sputum (in order of priority)
- Expectorated sputum for smear and culture (solid and liquid)
- Induced sputum for biomarkers

Blood
- Biomarkers

Additional clinical labs CBC, chemistries, LFTs, etc ***are not study required*** *but may be done as clinically indicated*

Urine
- Biomarkers

Note that sputum samples are required to be collected at every visit throughout the study. Every effort should be made to obtain the required number of sputum samples. Sites should provide sputum cups to participants to bring home and instruct participants to collect overnight sputum and morning sputum on the day of their next visit. If the participant is unable to produce sputum (especially during the later months of the study), an induced sputum is encouraged if possible but not required. If induction is not done, the participant should be instructed to cough vigorously and clear the back of his/her throat to produce an adequate volume of saliva for testing. A sample of sputum or saliva should always be collected at every visit. **At this visit, expectorated sputum should be collected for culture. Induced sputum should be collected for biomarkers.**

Schedule week 60 phone call (+/- 30 day window, but as close to actual date as possible)

Provide compensation

6.12.13 WEEK 60 VISIT FLOW (+/- 30 day window)

CRF

1. TEL

Call Participant

ADMINISTER QUESTIONS AND FILL OUT TELEPHONE CALL SOURCE DOCUMENT

If participant answers no to all questions, schedule Week 72 study visit (+/- 30 day window).

If participant answers yes to any question, ask him/her to come to study clinic for full study visit and evaluation for recurrent TB.

At site visit, follow requirements for unscheduled visit/recurrence evaluation.

If recurrence is suspected based on history, physical exam, and laboratory results (visit may last >1 day), participant should complete procedures (labs, PET/CT) for a recurrence visit. (See recurrence flow diagram.)

6.12.14 WEEK 72 VISIT FLOW (+/- 30 day window)

Perform
- Focused interval medical history (if indicated)
- Focused physical exam (if indicated)
- Vital signs

CRFs

Site		Lab	
1.	VSPE	8.	SMEAR
2.	TBS	9.	SPUQUA
3.	SPU	10.	LJ (Critical)
4.	BLDSTR	11.	MGIT (Critical)
5.	AE	12.	SPUSTR 1
6.	CONMED	13.	SPUSTR 2

Labs

Sputum (in order of priority)
- Expectorated sputum for smear and culture (solid and liquid)
- Expectorated sputum for XPERT MTB/RIF and XPERT ULTRA
- Induced sputum for biomarkers

Blood
- Biomarkers

Additional clinical labs CBC, chemistries, LFTs, etc ***are not study required*** *but may be done as clinically indicated*

Urine
- Biomarkers

Note that sputum samples are required to be collected at every visit throughout the study. Every effort should be made to obtain the required number of sputum samples. Sites should provide sputum cups to participants to bring home and instruct participants to collect overnight sputum and morning sputum on the day of their next visit. If the participant is unable to produce sputum (especially during the later months of the study), an induced sputum is encouraged if possible but not required. If induction is not done, the participant should be instructed to cough vigorously and clear the back of his/her throat to produce an adequate volume of saliva for testing. A sample of sputum or saliva should always be collected at every visit. **At this visit, expectorated sputum should be collected for culture. Induced sputum should be collected for biomarkers.**

Provide compensation and information about who to contact with questions in the future.

Note the visit window may be extended for up to 11 more months if participant is unable to make +/- 30d window

6.12.15 Recurrence

CRFs

Site		Lab	
1.	VSPE	10.	SMEAR
2.	TBS	11.	SPUQUA
3.	BLOOD	12.	LJ (Critical)
4.	PETSAL	13.	MGIT (Critical)
5.	SPU	14.	XPERT
6.	BLDSTR	15.	SPUSTR 1
7.	AE	16.	SPUSTR 2
8.	CONMED	17.	SALSTR (SUN)
9.	RTROUT (re-treatment outcome)	18.	RLPISO (relapse isolate)

Perform

- Focused interval medical history (if indicated)
- Focused physical exam (if indicated)
- Vital signs

Labs

Sputum (in order of priority)

- Expectorated sputum for smear and culture (solid and liquid)
- Expectorated sputum for Xpert
- Expectorated or induced sputum for biomarkers

It is VERY important to collect multiple sputa samples for culture over >1 day BEFORE re-starting treatment

Blood

- CBC/Chemistries/LFTs
- HbA1C
- HIV
- Biomarkers

Urine

- Biomarkers
- Pregnancy Testing, if applicable

Note that sputum samples are required to be collected at every visit throughout the study. Every effort should be made to obtain the required number of sputum samples. Sites should provide sputum cups to participants to bring home and instruct participants to collect overnight sputum and morning sputum on the day of their next visit. If the participant is unable to produce sputum (especially during the later months of the study), an induced sputum is encouraged if possible but not required. If induction is not done, the participant should be instructed to cough vigorously and clear the back of his/her throat to produce an adequate volume of saliva for testing. A sample of sputum or saliva should always be collected at every visit. **At this visit, expectorated sputum should be collected for culture. Induced sputum should be collected for biomarkers.**

Ensure they will have their PET/CT (scan should be as soon as possible but within 2 weeks of recurrence), including finger stick glucose and pregnancy

Saliva sample at end of visit or at PET/CT center

Refer to national program for re-treatment and follow observationally for final re-treatment outcome

Provide compensation

6.12.16 PET/CT VISIT FLOW

WINDOWS:

- **Baseline scan within 7 days after treatment initiation;**
- **Week 4 scan at least 4 weeks after baseline scan with a -3/+7 day window;**
- **Weeks 16 and 24 scan within 14 days of visit;**
- **Recurrence scan should be as soon as possible, but within 2 weeks of recurrence**

Call to schedule PET/CT scan

Call Participant 1-3 days before the scan to confirm. Remind him/her not to eat within 6 hrs of scan.

Arrange transportation to the scan

DAY OF SCAN

1. **COLLECT SALIVA (IF APPLICABLE)**
2. **DO URINE PREGNANCY TEST (IF APPLICABLE)**
3. **COLLECT FINGERSTICK GLUCOSE**

Provide compensation

Transport participant back home or to clinic

REMEMBER TO…..

- **Save PET/CT dicom and raw files.**
- **Upload dicom and raw files to Aspera.**

All PET/CT scans will be read clinically by nuclear medicine physicians and a clinical report will be provided back to the participant's study physician for clinical follow-up as needed. For research purposes, the PET/CT scans will be read centrally by 2-3 readers with the research result (what arm the participant is in) emailed to the site staff about 4 weeks following the baseline and week 4 PET/CT scans.

7 Study Events

7.1 Criteria for Being Randomized at Week 16 (Protocol Table 6, sec 3.1)

Participants who meet the following criteria will be randomized to Arm B or C at week 16:

Baseline PET/CT scan:

- No total lung collapse of a single side, AND
- No pleural effusion, AND
- No single cavity air volume on CT scan >30 mL, AND
- CT scan hard volume (-100 to +100 HU density) <200 mL, AND
- PET total activity <1500 units

Participants who do not meet any one of these criteria will be placed into Arm A and will subsequently be randomized to receive his/her 3rd PET/CT scan at Week 16 or Week 24.

Week 4 PET/CT scan:

- All individual cavities decrease by >20% (unless cavity <2 mL), AND
- CT scan hard volume does not increase by >10% unless the increase is <5 mL, AND
- PET total activity does not increase by >30% unless the increase is <50 units

Participants who do not meet any one of these criteria will be placed into Arm A and will subsequently be randomized to receive his/her 3rd PET/CT scan at Week 16 or Week 24.

Week 16:

- Xpert MTB/RIF cycle threshold ≥30. Note that if the week 16 solid or liquid medium sputum culture is subsequently found to be positive for *Mtb* in a participant randomized to Arm B or C, this participant will be called in for evaluation and to provide sputum for a repeat culture. If the initial positive culture is confirmed by a second positive culture, this participant will be considered to have met the study endpoint as a treatment failure and will be referred for continued treatment.
- Minimum of 100 treatment doses received

Participants who do not meet all of these criteria will be placed into Arm A. Randomization to receive a PET/CT scan at Week 16 or Week 24 will be performed.

7.2 Criteria for Achieving a Study Endpoint

Study endpoints are defined in protocol sec. 6.2 as follows:

- Treatment success: Participants with at least 2 consecutive negative cultures on solid medium over a span of at least 4 weeks, achieved before the end of therapy, with no subsequent confirmed positive cultures during treatment or follow-up.
- Treatment failure:
    - Participants who remain culture positive on solid medium at Week 24 in Arm A will be considered treatment failures and will be withdrawn from the study and referred to continue treatment per the local SOC. Participants who convert to solid culture negative who have a single solid culture positive for *Mtb* at week 24 need to have a subsequent culture positive for *Mtb* to be confirmed as treatment failures.

  - Note that solid culture results will be used for all primary endpoint analyses with the exception that the liquid culture result may be used at the final week 72 study visit if the solid culture result is contaminated or missing and the participant cannot be brought back to repeat the sputum sample. In this instance, if the liquid culture result is negative, the sputum sample will be considered negative. For other study visits, only solid culture results will be used for the primary analysis. Solid culture results that are missing or contaminated will be classified as unavailable. Liquid culture results may be used for secondary analyses.
  - Participants randomized to Arms B or C who are subsequently found to have a positive culture for *M.tb* on solid medium at week 16 that is confirmed on a subsequent culture will be considered treatment failures. These participants will be discontinued from the study as having met a study endpoint and referred to continue treatment per local SOC.
  - Note that single positive cultures that are not confirmed on a subsequent sputum sample are not considered failures as these may have arisen from clerical error or laboratory contamination
- Treatment recurrence:
  - Participants who convert their sputum to culture negative (2 consecutive negatives over ≥4 weeks) and who subsequently become culture positive for *M.tb* again on solid medium during follow-up after treatment completion, confirmed by a second sputum culture positive for *M.tb* collected on a different day, will be considered recurrences. Single positive cultures that are negative on follow-up culture will not be considered recurrences. Participants with a positive, contaminated, or unevaluable culture on the final month 18 (week 72) follow-up visit may be asked to return for sputum culture confirmation.
  - Relapses will be distinguished from re-infections by DNA strain typing (protocol sec. 5.5.3) and only relapses will be considered a study endpoint.
  - National records may be looked at to determine if someone has been diagnosed with TB again, in the event that someone doesn't come back for their study visits.

7.3 Criteria for Stopping the Study

As specified in protocol sec. 6.7, the protocol may be stopped early during interim analyses for two different reasons. The first reason is inferiority of the treatment shortened Arm C compared to Arm B. A Fisher's Exact test will be performed after 1/3 and 2/3 of participants have been followed for 72 weeks from study entry, using a Pocock boundary. The stopping boundary is derived from a test of inferiority (of the treatment shortening arm) that corresponds to a z-score of 2.178 (i.e., a two-sided p-value of 0.029).

The second reason for stopping early is for study futility, which may occur if the success rate in Arm B is lower than the estimated 97% used to calculate the sample size such that the study would have insufficient power to demonstrate non-inferiority with the current sample size. When half of the participants have completed their week 72 follow-up, the treatment success rate will be evaluated and presented to the DSMB. If more than 16 (of 75) patients in the standard treatment arm have a relapse, a recommendation to stop the trial will be considered. Under this scenario the highest achievable success rate in the standard treatment arm is 90%,

which would correspond with low power to prove non-inferiority of the treatment-shortened arm. Note that, in the event of a lower than anticipated treatment success rate, the bounds on the confidence interval of the treatment success rate between treatment arms will still remain informative. Therefore, the criteria to stop a study for futility are expected to be met only in a relatively extreme setting.

7.4 Adverse Events (AE), Unanticipated Problems (UP), Deviations, and Non-compliance

7.4.1 Definitions

Adverse Event: Any untoward medical occurrence in a human participant, including any abnormal sign, symptom, or disease, temporally associated with the participant's participation in research, whether or not considered related to the participant's participation in the research.

Serious adverse event: Any adverse event that

- results in death;
- is life-threatening (places the participant at immediate risk of death from the event as it occurred);
- results in inpatient hospitalization or prolongation of existing hospitalization;
- results in a persistent or significant disability/incapacity;
- results in a congenital anomaly/birth defect; or
- based upon appropriate medical judgment, may jeopardize the participant's health and may require medical or surgical intervention to prevent one of the other outcomes listed in this definition *(examples of such events include allergic bronchospasm requiring intensive treatment in the emergency room or at home, blood dyscrasias or convulsions that do not result in inpatient hospitalization, or the development of drug dependency or drug abuse).*

Protocol Deviation: Any change, divergence, or departure from the IRB approved study procedures in a research protocol. Protocol deviations are designated as serious or non-serious and are further characterized as the following:

1) Those that occur because a member of the research team deviates from the protocol
2) Those that are identified before they occur, but cannot be prevented
3) Those that are discovered after they occur

Serious Protocol Deviation: A deviation that meets the definition of a Serious Adverse Event or compromises the safety, welfare or rights of participants or others.

Non-compliance: The failure to comply with applicable NIH HRPP policies, IRB requirements, or regulatory requirements for the protection of human participants. Non-compliance is further characterized as the following:

1) Serious: Non-compliance that
    a. increases risks, or causes harm, to participants
    b. decreases potential benefits to participants
    c. compromises the integrity of the NIH-HRPP
    d. invalidates the study data
2) Continuing: Non-compliance that is recurring
3) Minor: Non-compliance that, is neither serious nor continuing

Unanticipated Problem (UP): Any incident, experience, or outcome that meets all three of the following criteria would be considered a serious UP:

1) unexpected in terms of nature, severity, or frequency in relation to
    a. the research risks that are described in the IRB-approved research protocol and informed consent document; Investigator's Brochure or other study documents; and
    b. the characteristics of the participant population being studied
2) related, possibly or probably related to participation in the research
3) suggests that the research places participants or others at a greater risk of harm (including physical, psychological, economic, or social harm) than was previously known or recognized

Unanticipated problem that is not an Adverse Event (UPnonAE): An unanticipated problem that does not fit the definition of an adverse event, but which may, in the opinion of the investigator, involve risk to the participant, affect others in the research study, or significantly impact the integrity of research data. These events may involve a greater risk of social or economic harm to participants or others rather than physical/psychological harm. Such events would be considered a non-serious UP. Examples of an UPnonAE include a breach of confidentiality, accidental destruction of study records, or unaccounted-for study drug.

7.4.2 Grading AEs for Severity

The severity of each AE will be determined using the DAIDS Table for Grading the Severity of Adult and Pediatric Adverse Events, version 2.0, November 2014. This table can be found using the following link: DAIDS Toxicity Table. Any event not listed in this toxicity table will be graded by the local investigator as follows:

Grade 1 -	Mild	Transient or mild discomfort; no limitation in activity; no medical intervention/therapy required
Grade 2 -	Moderate	Moderate limitation in activity – some assistance may be needed; no or minimal medical intervention/ therapy required

Grade 3 -	Severe	Marked limitation in activity; some assistance usually required; medical intervention/therapy required, hospitalizations possible
Grade 4 -	Life-threatening	Extreme limitation in activity, significant assistance required; significant medical intervention/therapy required, hospitalization or hospice care probable

Grade 5 – Death

7.4.3 Assessing Adverse Events for Relationship to Study

Any AE that occurs in a participant will be assessed for its relationship to the study. A causal relationship means an intervention caused (or is reasonably likely to have caused) the AE. This usually implies a relationship in time between one or more interventions and the AE—for example, the AE occurred shortly after the participant received the drugs/study agents/intervention.

For all AEs, the clinician who examines and evaluates the participant will determine the AE's causality based upon the temporal relationship to administration of the intervention, the pharmacology of any applicable study agents, and his/her clinical judgment.

The following scale will be used to reflect the PI's judgment as to the relationship between the intervention and the AE:

Definitely Related: The AE is clearly related to one or more of the interventions – follows a reasonable temporal sequence from administration of one or more of the interventions, follows a known or expected response pattern to the one or more of the interventions that is confirmed by improvement on stopping and reappearance of the event in repeated exposure and that could not be reasonably explained by the known characteristics of the participant's clinical state.

Probably Related: The AE and administration of the interventions are reasonably related in time and/or follows a known pattern of response, and the AE is more likely explained by one or more of the interventions than other causes.

Possibly Related: AE follows a reasonable temporal sequence from administration of the interventions, follows a known or expected response pattern to the suspected intervention or interventions, but that could readily have been produced by a number of other factors.

Unlikely Related: A potential relationship between one or more of the interventions and the AE could exist (i.e., the possibility cannot be excluded), but the AE is most likely explained by causes other than one or more of the interventions (e.g., could readily have been produced by the participant's clinical state or could have been due to environmental or other interventions)

Unrelated: AE is clearly not related to one or more of the interventions– another cause of the event is most plausible and/or a clinically plausible, temporal sequence is consistent with the onset of the event and the intervention administration and/or event is biologically implausible.

7.4.4 Recording

Per protocol section 8.3, all adverse events that occur during the study will be recorded in each study participant's source documents, for example their research study chart. All recorded adverse events should be assessed for:

1) Seriousness
2) Severity using the DAIDS toxicity table 2014 version 2.0 (2014) at http://rsc.tech-res.com/safetyandpharmacovigilance/gradingtables.aspx
3) Expectedness: Expected adverse events are listed in protocol section 8.5. Any treatment-related adverse events are also considered expected.
4) Relatedness to the study (protocol section 8.2.2) as definitely related, probably related, possibly related, unlikely related, and unrelated.

All adverse events should be managed promptly and appropriately with actions taken recorded in the participant's research study chart. Adverse events should be followed to resolution in subsequent study visits. Unresolved/ongoing adverse events at the end of the study should be recorded as such.

After the initial screening labs, routine safety labs (CBC, LFTs) are not included as a part of study visits but should be ordered as clinically indicated by the study physician.

In South Africa: Participants who are withdrawn from the study due to an AE (or any other reason) while they are still on treatment will be referred back to the National Programme's clinic managers. The clinic managers will be presented with a report and treatment plan for the person and this transfer will be documented.

In China: Participants who are withdrawn from the study due to an AE (or any other reason) while they are still on treatment will continue on standard of care treatment off study through the local TB treatment program, which likely is the same clinic site as the research clinic.

7.4.5 Reporting Guidelines

Although all adverse events occurring during the study should be *recorded* in your source documents, not all need to be *reported* to NIH or to your IRB/ethics committee. Because the TB drugs used in this study are not considered study drugs, all treatment-related AEs are considered expected and do not need to be reported *unless it meets the definition of a SAE or UP*.

Expected AEs:

Intervention or potential cause of AE	Adverse effects
**Blood drawing**	**Common:** Discomfort
	**Significant but rare:** Hematoma, Infection, nerve damage, syncope
**FDG-PET/CT scan**	**Significant but rare:** Hematoma, thrombophlebitis, infection, nerve damage, syncope
**Induced sputum**	Can have coughing, wheezing, and or bronchospasms

7.4.5.1 *Investigator Reporting Responsibilities to the NIAID Institutional Review Board (IRB)*

7.4.5.1.1 *Expedited Reporting to the NIAID IRB*

Serious and non-serious unanticipated problems, deaths (except for those related to the natural history of TB), serious deviations, and serious or continuing non-compliance will be reported within 7 calendar days of investigator awareness. Serious Adverse Events that are possibly, probably, or definitely related to the research will be reported to the NIAID IRB within 7 calendar days of investigator's awareness, regardless of expectedness.

7.4.5.1.2 *Waiver of Reporting Anticipated Protocol Deviations, Expected UPnonAEs and Deaths to the NIAID IRB*

Anticipated deviations in the conduct of the protocol will not be reported to the IRB unless they occur at a rate greater than anticipated by the study team. Expected adverse events will not be reported to the IRB unless they occur at a rate greater than that known to occur in those with tuberculosis. If the rate of these events exceeds the rate expected by the study team, the events will be classified and reported as though they are unanticipated problems. Deaths related to the natural history of tuberculosis will be reported at the time of continuing review.

7.4.5.1.3 *Annual Reporting to the NIAID IRB*

The following items will be reported to the NIAID IRB in summary at the time of Continuing Review. To note, we will not report on adverse events related to TB drugs.

- Serious and non-serious unanticipated problems
- Expected serious adverse events that are possibly, probably, or definitely related to the research
- Serious adverse events that are not related to the research
- All adverse events, except expected AEs and deaths granted a waiver of reporting
- Serious and Non-Serious Protocol deviations
- Serious, continuing, and minor non-compliance
- Any trends or events which in the opinion of the investigator should be reported

The NIAID PI is responsible for reporting to the NIAID IRB. All reportable events will be reported by email, telephone, or fax by the participating sites to the following:

	Phone	Fax	Email	Address
**Clifton E. Barry, 3rd, Ph.D.**	+1-301-693-4665	+1-301-402-0993	**cbarry@niaid.nih.gov**	Building 33, Room 2w10D, Bethesda, MD
**Ray Chen, MD (NIAID Medically Accountable Investigator)**	+1-301-443-5816	+1-301-451-5492	**rchen@niaid.nih.gov**	Building 33, Room 2w10B, Bethesda, MD

7.4.5.2 ***Local Study Site Reporting To Local IRB***

The Local PIs have the responsibility to report AEs to their local IRB. The Local PIs also have the responsibility to report to the NIAID team.

7.4.5.2.1 ***China Team to Local IRB***

In the event of an unanticipated problem (UP)

1. The local principal investigator must initially report the UP and summary of the problem to the local IRB as soon as possible (usually within 24 ~ 48 hours) after awareness.

2. After the initial report, the local PI must provide a formal report to the IRB within 7 days of awareness including the investigator’s judgment of harmfulness to the participant(s) and others.

7.4.5.2.2 ***South African Team to the ECs***

In the event of an unanticipated problem (UP)

1. The Local principal investigator must initially report the UP and summary of the problem to the local IRB according to the local IRB SOPs as soon as possible after awareness.

2. After the initial report, the Local PI must provide a formal report including any relevant information to the IRB as necessary, including the investigator’s judgment of harmfulness to the subject(s) and others.

7.4.5.3 ***South African Team to the MCC***

The local study teams are responsible for reporting reportable events to Triclinium who will in turn report to the MCC.

7.4.5.4 ***Local Site Reporting To NIAID Study Team***

The Local PI has the responsibility to report serious and non-serious unanticipated problems, deaths, serious deviations, and serious or continuing non-compliance to the NIAID PI within 3 calendar days of investigator awareness. Serious Adverse Events that are possibly, probably, or definitely related to the research will be reported to the NIAID PI within 3 calendar days of investigator's awareness, regardless of expectedness.

Deaths which do not fit into these categories above (i.e. are due to the natural history of tuberculosis disease itself) should be reported to the NIAID PI within 7 days.

Anticipated deviations in the conduct of the protocol will be recorded and if they occur at a rate greater than anticipated by the study team, they should be reported to the NIAID PI. Expected adverse events that occur at a rate greater than that known to occur in those with tuberculosis will be reported to the NIAID PI. If the rate of these events exceeds the rate expected by the study team, the events will be classified and reported as though they are unanticipated problems.

7.4.6 **Communications Flow for Events**

Flow of China Site Reporting of SAEs and UPs to the NIAID Team:

1. Chinese investigators will fill out the SAE forms and Problem Reports in Mandarin.
2. CRFs will be entered into DataFax in real time (same day as awareness of the event) and DataFax will send an email flag to the AE/SAE team (Gao Jingcai, Zhu Hong, Kriti Arora, Ray Chen, Chrissie Cai)
3. Gao Jingcai will translate the narrative into English and enter that narrative into DataFax. DataFax will again notify the AE/SAE team that an SAE and narrative is ready for review.
4. NIAID side of the AE/SAE team (Ray Chen/Kriti Arora) will review the SAE. If there are questions, Gao Jingcai/Zhu Hong will be notified and they will ask the investigator who reported the SAE.
5. NIAID team (Kriti Arora) will report the SAE to the NIAID IRB and ensure that the Chinese team has reported the SAE to the HPCH IRB.

Flow of South African Site Reporting of SAEs and UPs to the NIAID Team

1. South African investigators will fill out the SAE forms and Problem Reports.
2. Forms will be scanned into DataFax in real time (same day as awareness of the event) and DataFax will send an email flag to the AE/SAE team.
3. NIAID side of the AE/SAE team (Ray Chen/Kriti Arora) will review the SAE. If there are questions, the site will be notified.
4. NIAID team will report the SAE to the NIAID IRB and ensure that the site team has reported the SAE to their EC.

EVENT REPORTING TABLE

Reportable Event	Definitions	Group/Site to be Notified	Timeline
**Serious Adverse Event (SAE)**<br>**• SAE Reporting Form**<br>**• SAE Line Listing**	SAEs are any adverse events / experiences occurring the course of the study that result in any of the following outcomes:<br>• Death<br>• Life threatening (subject at immediate risk of death)<br>• Requires inpatient hospitalization or prolongation of existing hospitalization<br>• Results in congenital anomaly/birth defect<br>• Results in persistent or significant disability or incapacity	1. Local EC/IRB, if reportable in real-time<br>2. NIAID Site Team (ray.chen@nih.gov; arorakriti@niaid.nih.gov)	**To the NIAID Team:** Serious Adverse Events that are possibly, probably, or definitely related to the research will be reported to the NIAID PI within 3 calendar days of investigator's awareness, regardless of expectedness. Deaths that do not fit into the category above should be reported to the NIAID team within 7 days of awareness.<br>**To the HPCH IRB**: per local requirements<br>**To the UCT EC and SUN EC**: per local requirements<br>**MCC:** per local requirements
**Unanticipated Problem (UP)**<br>**• NIAID Problem Report**	Unanticipated problems involving risks to participants or others is defined as a problem that:<br>1. is unexpected in terms of nature, severity or frequency; and<br>2. is related or possibly related to participation in the research; and<br>3. suggests that participants or others are at greater risk of harm than was previously known or recognized.	1. Local EC/IRB<br>2. NIAID Site Team (ray.chen@nih.gov; arorakriti@niaid.nih.gov)	**To the NIAID Team:** Serious and non-serious unanticipated problems will be reported to the NIAID PI within 3 calendar days of investigator's awareness.<br><br>**To the HPCH IRB:** The local principal investigator must initially report the UP and summary of the problem to the local IRB as soon as possible (usually within 24 ~ 48 hours) after awareness.<br>After the initial report, the local PI must provide a formal report to the IRB within 7 days of awareness including the investigator's judgment of harmfulness to the participant(s) and others.<br><br>**To the UCT EC and SUN EC:** The Local principal investigator must initially report the UP and summary of the problem to the local IRB according to the local IRB SOPs as soon as possible after awareness.<br>After the initial report, the Local PI must provide a formal report including any relevant information to the IRB as necessary, including the investigator's judgment of harmfulness to the subject(s) and others.<br><br>**MCC**: per local requirements
**Adverse Event (AE)**<br>**• Adverse Event Source Document**<br>**• Adverse Event CRF**	AEs are any untoward medical occurrence in a participant administered a pharmaceutical product regardless of its causal relationship to the study treatment.	1. Local EC/IRB<br>2. NIAID Site Team (ray.chen@nih.gov; arorakriti@niaid.nih.gov)<br>3. MCC	Per protocol, any AE related to TB treatment is considered expected and will not be reported. All other AEs will be reported at time of CR and any biyearly review (MCC). Expected AEs that occur at a rate greater than that known to occur in those with TB will be reported to the NIAID PI.

**Protocol Deviation (PD)**<br>• **NIAID Problem Report**<br>• **Deviation Log**	Deviations from the most recent version of the approved study protocol; these PDs involve risks to subjects or others, or affect the scientific soundness of the research plan.	1. Local EC/IRB<br>2. NIAID Site Team (ray.chen@nih.gov; arorakriti@niaid.nih.gov)<br>3. MCC	**To the NIAID Team:** Serious deviations and non-compliance will be reported to the NIAID PI within 3 calendar days of investigator's awareness. Anticipated deviations in the conduct of the protocol will be recorded and if they occur at a rate greater than anticipated by the study team, they should be reported to the NIAID PI.<br>**To the HPCH IRB:** per local requirements<br>**To the UCT EC and SUN EC**: per local requirements<br>**MCC:** per local requirements

7.5 Medically Accountable Investigator

NIAID policy requires principal investigators who are not medical doctors to appoint a medically accountable investigator (MAI) who is a medical doctor to oversee their studies. Dr. Ray Chen serves as the MAI on the Predict Trial. He is responsible for reviewing all reportable events on the study and liaising with the sites regarding questions of a medical nature. In the event of an emergency, Dr. Chen can be reached at +1-240-786-8167.

7.6 Data and Safety Monitoring Board (DSMB)

Per protocol sec. 10.2, the standing NIAID DSMB with the addition of 3 ad hoc members who are TB experts will evaluate safety, study conduct, and scientific validity and integrity of the trial. The three ad hoc members are W. Henry Boom, USA; Patrick Phillips, UK; and Nick Paton, Singapore. The DSMB will meet by telephone or in person at least twice per year with the first meeting convened before the study starts. DSMB members must be satisfied that the timeliness, completeness, and accuracy of the data submitted to them for review are sufficient to evaluate the safety and welfare of study participants. The DSMB will also assess the performance of overall study operations and any other relevant issues, as necessary. The DSMB will conduct interim analyses for:

1) Safety (inferiority of Arm C compared to Arm B) when ⅓ and ⅔ of participants have completed the study (18 months follow-up) and
2) Futility (treatment success rate in Arm B) when ½ of participants have completed the study.

Serious adverse event reports will be sent by the PI electronically to the DSMB members and the DSMB Executive Secretary before each meeting and within 7 days after knowledge of a relapse or per the DSMB guidelines. Enrollment will continue unless the DSMB requests stopping enrollment to perform a more in-depth review. The DSMB will also be available for other clinical advice as requested.

Items reviewed by the DSMB include but are not limited to:

1) SAE and AE line listings and SAE narratives
2) Demographic information on study participants
3) TB disease recurrences
4) Interim analysis of treatment success rates
5) Factors that might affect the study outcome or compromise the confidentiality of the trial data (protocol violations, unmasking, etc.)
6) Factors external to the study, such as scientific or therapeutic developments, that may adversely affect participant safety or the ethics of the study

In addition, the DSMB recommends:

- That data from Arms B and C be presented without identification of treatment group, with unblinding codes readily available to the DSMB.
- That efficacy and safety outcomes stratified by country.

8 Pharmacokinetic Substudy for Sub-Breakpoint Minimum Inhibitory Concentrations (MIC) Comparison

8.1 Substudy Design

The purpose of this substudy is to test the hypothesis that one reason why some patients fail treatment may be because their Mtb is slightly more resistant to the standard medicines than the Mtb in other patients who are successfully treated. Because serum drug concentrations can vary widely in TB patients, we also need to test drug pharmacokinetic (PK) levels in these patients.

Two types of participants are eligible for this substudy. The first are participants at the highest risk of relapse of TB. Based on preliminary data, we believe that participants who are placed in Arm A due to a poor treatment response on the week 4 PET/CT scan are at higher risk of relapse. These participants will be targeted for recruitment into the substudy. The second type of participant recruited for this substudy are control participants, those not believed to be at higher risk of relapse. For every participant who moves to Arm A after the week 4 PET/CT scan and is recruited for this study, a participant who remains in Arms B or C after the week 4 PET/CT scan will be recruited as a control.

Participants enrolled onto this substudy agree to provide 0, 1, 2, and 6-hour post-treatment dose blood samples (approximately 2 mL) on 2 different study visits while still on treatment for INH and RIF PK. Participants must arrive on that study visit day without having taken their TB treatment dose. A baseline trough blood sample will be drawn, after which participants will take their TB treatment dose. Three more blood samples (approximately 2 mL) will then be collected at 1, 2, and 6 hours after the TB treatment dose.

Two days of PK sampling per participant are necessary to differentiate inter-patient variability from within patient inter-occasion variability. Inter-occasion variability is “random” variability due to factors such as food intake, concomitant medicines, co-morbidities, etc., that may affect absorption and that change over time. With two sets of data points at different times for each patient, the PK modelers will be able to differentiate signal from noise much more effectively.

8.2 Eligibility Criteria and Identification of Participants

8.2.1 Main Substudy Participants

Inclusion Criteria	Exclusion criteria
Participant moved to Arm A due to Week 4 PET/CT result	None
Willing to come for two study visits between weeks 4-24 (including Week 24) without having taken that day’s TB drug dose, then stay for at least 6 hours for blood draws at 0,	

1, 2, and 6 hours after taking the TB drug dose	
Willing to have samples stored	
Willing to sign substudy informed consent	

8.2.2 Control Participants

For each participant from Arm A invited to participate in this substudy, a similar number of participants from Arms B or C will be invited to participate as controls. Control participants from Arm C must complete PK sampling no later than the week 16 study visit as they will conclude treatment at that time.

Inclusion Criteria	Exclusion criteria
In Arm B or C after the week 4 PET/CT scan	None

8.3 Substudy Study Visits and Implementation

After the week 4 PET/CT scan for each participant, the PET/CT scans will be read centrally and those participants who do not meet the week 4 PET/CT early treatment completion criteria will be identified for placement into Arm A. Site staff will be notified of these participants and these participants should be approached at their next study visit (week 8) about their interest in participating in the substudy. They can be approached before this if they come to the clinic and are interested in hearing about the substudy. Those interested in participating should sign the substudy informed consent. For each Arm A participant recruited onto the substudy, site staff should identify and recruit an Arm B/C participant (likely at his/her week 8 study visit) as the control. All substudy participants should be instructed to come to their next study visit (potentially week 12) without having taken their TB drug dose for that day.

At the week 12 study visit (or earlier if they were consented before week 8), substudy participants should arrive without having taken their TB drug dose for that day. A trough blood sample should be drawn, then the participant should take his/her TB treatment dose. Blood samples (approximately 2 mL) should then be drawn 1, 2, and 6 hours later. MERM container opening times for the previous 2 days should also be recorded. Participants should be instructed to come to their next study visit again without having taken their TB treatment dose for that day.

Note that PK blood samples should not be drawn if the participant arrives to the clinic already having taken his/her TB treatment dose for that day and the blood PK sampling should be deferred until the next visit.

At the second substudy visit, the same procedure as at the first substudy visit should be followed. If this is the 2nd day of PK sampling for the participant, then that concludes the participant's participation in the substudy. If one or both previous PK sampling visits was missed, then PK sampling should be scheduled for the next visit(s) as needed until two days of PK sampling are completed.

Note that PK sampling must be done while the participant is still on INH and RIF treatment. **Thus, control participants (those still on Arm B/C) need to conclude substudy participation by week 16 because half will be randomized to complete treatment at that time.** If an earlier substudy visit was missed for any reason (e.g. participant took drug dose that morning), a visit just for the substudy may need to be scheduled to complete the 2 substudy visits before week 16. Arm A substudy participants will conclude substudy participation by week 24 (or later if treatment is extended for participants in Arm A). A substudy-only visit may be scheduled as needed to complete both substudy visits before the end of treatment.

8.4 Procedure for Collection of Substudy Blood Samples

Placement of a peripheral venous access device (IV catheter for a saline or heparin lock) is permissible for PK sampling since venous access is required for multiple blood draws. The decision to use a venous access device versus performing a venipuncture for each sampling is at the discretion of the site. Each site should follow the respective institution's procedures for IV site selection, cannulation procedures, and flushing and discarding blood from a venous access device. In general, at least 1-2 mL of blood should be discarded prior to collection of the PK sample if a venous access device is used.

- Follow universal (standard) safety precautions.
- Blood draws should be performed by trained medical personnel only.
- Verify the participant's name and PID before initiating any procedures.

8.4.1 Schedule of PK Sample Collection

PK data will only be collected for INH and RIF. The study timeline is as follows. (Note, PK visits may occur between main study visits.)

- Week 4: PET/CT scan (from main study)

- Weeks 5-7: Identification of those participants who are moved to Arm A and are therefore eligible for this substudy. These participants will be identified once the Week 4 scans are read. After these participants are identified, they may be scheduled for PK visit dates at any time, even if not at the dates recommended below. The timeline below may be used as a guide.

- Week 8: Site staff introduce substudy to eligible participants during regular study visit. Those willing to participate will provide informed consent and will be instructed to come to their week 12 study visit without having taken their TB drug dose for that day.

- Week 12: Substudy participants come for their week 12 study visit without having taken their TB drug dose for that day. More detailed methods for the blood draws and processing of blood into plasma are found in the laboratory MOP. The time of TB medication intake (e.g. pill box opening times) for the two previous days are recorded. A trough blood (approximately 2 mL) is drawn into a lavender top [EDTA] tube, then the TB drug dose is taken. Blood (approximately 2 mL) is then drawn again at approximately 1, 2, and 6 hours post-dose. Note that PK sampling cannot be done if the participant

takes his/her TB drug dose before coming to the clinic and sampling will be deferred to the next study visit.

Windows for the blood draws are +/- 10 min for the 1 and 2 hour draws and +/- 20 min for the 6 hour draw.

Participants are instructed to come to their week 16 study visit without having taken their TB drug dose for that day.

- Week 16: Substudy participants come for their week 16 study visit without having taken their TB drug dose for that day. A trough blood (approximately 2 mL) is drawn into a lavender top [EDTA] tube, then the TB drug dose is taken. Blood (approximately 2 mL) is then drawn at approximately 1, 2, and 6 hours post-dose. Note that the week 16 PK blood draws may be deferred to the week 20 visit or later as needed for Arm A participants only.

  Windows for the blood draws are +/- 10 min for the 1 and 2 hour draws and +/- 20 min for the 6 hour draw.

The substudy concludes after two days of PK sampling are completed but no later than the week 24 study visit.

9 Data Collection and Correction

Data for this study is managed using DataFax, a commercial software product designed for clinical trial data collection and management. Throughout the study site staff will record data on paper CRFs and then transmit the CRFs electronically to DataFax. The original paper copy of each CRF will remain in the participant's record, while the designated data management personnel will update the study database based on the received electronic copy.

Once a CRF image is received by the DataFax system, the following occurs:

1) DataFax determines the study and form type using the barcode at the top of the form. An image of each CRF is stored, and some fields are read by the system.
2) Next, each CRF is reviewed by a Data Reviewer (DR). Problems such as missing or potentially incorrect data are identified and marked with quality control (QC) notes through automated edit check programming.
3) QC notes are compiled into QC reports that are sent via e-mail to the Site Data Managers (SDMs) on a regular basis. The SDM is responsible for working with the site team and ensuring that all QC notes are resolved or to clarify any problems identified on the QC reports and re-transmitting the corrected CRFs to DataFax.
4) When the re-transmitted pages are received, DR staff will review the corrected pages and resolve the QC notes in the database.
5) It is important that the QC notes are resolved within <u>one week</u> to avoid repetition of the same query in subsequent QC reports.

9.1 Data Collection Process

9.1.1 **Source Documentation**

A source document is defined as the first document on which study-related information is recorded. Documentation of source data is necessary for the reconstruction, evaluation, and validation of clinical findings, observations, and other activities during a clinical trial. All data captured on the CRFs should be captured first on source documents, allowing comparison of data from source to CRF by monitors to ensure accurate transcription.

Source documents include but are not limited to

- Clinical progress notes
- Lab reports and results of diagnostic testing
- X-rays and PET/CT records
- Study worksheets where the data aren't recorded elsewhere first
- Signed informed consent forms
- Lab notebooks
- Prescription records

Source documents also include any written and/or documented verbal communication between the clinical investigator and participant or clinical investigator and nonstudy health provider regarding the present and/or past medical history of the participant. Source documentation also includes tools developed for capturing study-specific data.

For this trial, a common source document based on the CRFs is provided to each site to use. The sites are free to adapt this common source document to suit the needs of their own site. All sites should maintain a set of source documents in addition to the CRFs.

9.1.2 Research Chart

A research chart should be kept for each participant. The research chart should include but not be limited to:

- Source documents for the participant, including clinical progress notes, lab reports, x-rays, etc.
- Copies of participant's medical record, if applicable (such as an outpatient in China)
- Event reporting documentation
- Any other participant related documentation relevant to the study

9.1.3 CRFs

Case Report Forms are the main tools used to collect research relevant data from each participant. The CRFs have been designed by the NIAID study team and reviewed by the sites. These CRFs are designed to be compatible with DataFax. The CRFs in this study are in general not source documents and therefore all study data should be recorded somewhere other than the CRFs before being entered onto the CRF form itself.

9.1.3.1 *CRF Printing*

The final electronic set of CRFs is called the Master Forms. Each site should keep an electronic version of the approved Master Forms. Each site should print their CRFs on an as needed basis. Use only the printed Master Form—never photocopy the CRFs. Study coordinators or delegated study staff should be responsible for printing and storing blank CRFs for each visit. Blank CRFs for consecutive visits should be placed in the participant's binder once a participant is enrolled.

Note: The Study Coordinator should estimate the number ***of CRFs needed for a week, then*** *print and file accordingly.*

When printing Master Forms:

- Chose 'A4' or 'A3' paper
- "Auto-Rotate" and "Auto-Rotate and Center" are ok to use as these do not displace or resize graphics;
- Print double sided, as there is a signature date stamp on the back of the form
- "Scaling" should not be used;
- "Scale to Fit" should not be used;
- "Fit to printable area" should not be used;
- "Shrink to printable area" should not be used;
- "Shrink to Fit" should not be used….or anything similar to these that implies graphics are resized.

It is important to know the following when printing CRFs. DataFax uses four key fields to identify a submitted CRF page:

- Study number: located in the left section of the barcode
- Plate number: located in the middle section of the barcode
- Visit/page number: located in the right upper corner where it says Visit #. This field is pre-filled with each visit's visit number. If a visit is unscheduled visit or recurrent visit the visit number can be written in.
- Participant ID number: DataFax ID is country code-site number-sequential number with no dashes, as below (note FreezerPro label ID contains prefix PD with dashes, e.g. PD-1-1-001)

Site Name	Country Code	Site Code	Example
Khayelitsha (CIDRI)	1	1	11001, 11002, 11009
SATVI	1	2	12001, 12002, 12009
SUN	1	3	13001, 13002, 13009
TASK	1	4	14001, 14002, 14009
UCT Lung Institute	1	5	15001, 15002, 15009

Site Name	Country Code	Site Code	Example
Kaifeng	2	1	21001, 21002, 21009
Xinmi	2	2	22001, 22002, 22009
Zhongmu	2	3	23001, 23002, 23009
HPCH	2	4	24001, 24002, 24009

If the barcode has been distorted (shrunken or expanded), then DataFax will not be able to read it and therefore will not be able to identify the page.

Unidentified pages are automatically sent to the "unidentified fax router". This creates additional work for data entry staff or data managers who must identify and reroute the pages.

If checkboxes or number boxes have been distorted, DataFax's intelligent character recognition (ICR) function might read them incorrectly or not at all. Data values might be stored in the wrong fields in the database. This creates additional work for data entry staff or data managers who must find and correct misread data values.

9.1.3.2 *General CRF Completion*

Please refer to Completion Guidelines for DataFax CRFs document to complete CRFs.

9.1.3.3 *CRF Completion Timeline*

It is important that the CRFs are completed in a timely manner. Each site should complete transcribing data from source document, source data verification within 5 business days from the date of study visit or from the date lab results are available.

List of CRFs – Site packet

Plate Number	CRF Code	CRF Name	Responsibility	Critical Data Elements	Timeline (days + results days)
1	DEM	Demographics	Site		5
5	MHX1	Medical history	Site		5
6	MHX2	Medical history	Site		5
7	TBS	TB systems	Site		5
8	VSPE	Vital signs and physical exam	Site		5
9	SPU	Sputum Collection	Site	Yes	5
13	XPERT (Screening and Week 16 in China)	Xpert results	Site	Yes	5
15	BLOOD1	Blood tests (CBC)	Site		5+3
16	BLOOD2	Blood tests (CHM and LFT)	Site		5+3
17	HIV	HIV	Site		5
18	PREG	Pregnancy test	Site		5
19	EL1	Eligibility 1	Site		5
20	EL2	Eligibility 2	Site		5
21	EL3	Eligibility 3	Site		5
22	PETSAL	PETCT and Saliva	Site	Yes	5
23	ADHERE	Adherence monitoring	Site	Yes	5
24	BLDSTR	Blood and urine biomarker storage	Site		5
26	SUBEND	Sub study end of study	Site		5
27	PHONE	Telephone	Site		5
31	SALSTR*	Saliva sample storage	Site in RSA		5
32	SUB	Sub study blood collection	Site		5

33	SPUQUA*	Sputum Quality	Site in RSA		5
38	RETREAT	Re-treament Outcome	Site		5
39	PK-CONS	Sub study consent	Site		5
40	ADHERE2	Adherence monitoring 2	Site	Yes	5
300	CONMED	Concomitant medications	Site		5
350	AE	Adverse events	Site		5
498	MISS	Missed visit	Site		5
499	END	End of study	Site		5

List of CRFs – Lab packet

Plate Number	CRF Code	CRF Name	Responsibility	Critical Data Elements	Timeline (days + results days)
10	SMEAR	Smear Microscopy	UCT Barry /Core lab		5+7
11	LJ	LJ Culture	UCT Barry /Core lab	Yes	5+75
12	MGIT	MGIT Culture	UCT Barry/ Core lab	Yes	5+75
13	XPERT	Xpert	UCT Barry/ Core lab	Yes	5
14	PDST	Phenotypic DST results	UCT Barry/ Core lab		5+90
25	SPSTR1	Sputum biomarker sample storage 1	UCT Barry/ Core lab		5
30	SPSTR2	Sputum biomarker sample storage 2	UCT Barry/ Core lab		5
31	SALSTR*	Saliva sample storage	Core lab in China		5
33	SPUQUA*	Sputum Quality	Core lab in China		5
34	STRTYP	Strain Typing	UCT Barry/Core lab		5

List of CRFs – eDC CRFs

Plate Number	CRF Code	CRF Name	Responsibility	Critical Data Elements	Timeline

					(days + results days)
2	EARLY0	Early completion criteria (Baseline)	Auto completed		0
3	EARLY4	Early completion criteria (Week 4)	Auto completed		0
29	RAND12	Week 12 arm A randomization	Auto completed		0
28	WK16ADHERE	Week 16 early completion Adherence	Site		0
4	RAND16	Week 16 randomization	Site		0
400	DLEVEL	Drug level	Direct data import		batch

Image Reviewer CRFs

These are eDC CRFs completed by image reviewers via iDataFax. Only designated image reviewers have access to the review form that is assigned to him/her. The results (data) of their reviews will be auto filled to week 0, 4 early completion criteria CRFs. These CRFs will not be shown to sites.

MERM sub-study CRFs

This sub-study is only being done in certain sites in South Africa. The participant will fill out a participant questionnaire and a healthcare provider will fill out a provider's questionnaire. These CRFs are being placed in a separate study database and data will follow the same timeline as the main study paper CRF timeline.

Plate Number	CRF Code	CRF Name	Responsibility	Timeline (days)
37	PART	Participant questionnaire	Site	5
35,36	PROV	Provider's questionnaire	Site	5

9.1.3.4 *Visit and Page Numbering*

Every study visit has a visit number associated with it. Visit number is one of the 4 keys on the CRF located on the right upper corner of each page and is pre-printed. More than one sputum sample is likely to be collected at each visit, therefore, additional visit numbers have been allocated.

Week	Visit #	Visit # for Sputum Collection CRF

Screening	090	090, 091, 092, 093
Day0 / Baseline	000	000, 001, 002, 003
Week 1	010	010, 011, 012, 013
Week 2	020	020, 021, 022, 023
Week 4	040	040, 041, 042, 043
Week 8	080	080, 081, 082, 083
Week 12	120	120, 121, 122, 123
Week 16	160	160, 161, 162, 163
Week 24	240	240, 241, 242, 243
Week 36	360	360, 361, 362, 363
Week 48	480	480, 481, 482, 483
Week 60	600	600, 601, 602, 603
Week 72	720	720, 721, 722, 723
End of Study	499	499
Recurrent visit	8XX	

9.1.3.5 *Form Review Before Submission*

- Sites must review every page of every DataFax form to verify it is complete and accurate.
- Each site's data management SOP should specify review responsibilities.
- Review checklist:
    - Forms **must not** contain any information that identifies the participant (other than the subject ID). Examples of this type of personal information include: the participant's or family members' names, phone numbers, home address, national identification numbers, *etc*.
    - A response should be recorded for every item unless the item has been skipped *as instructed*.
    - Handwritten responses must be legible and appropriate to the question.
- **Repeated errors in CRF completion should be addressed** by additional training for the responsible individuals.
- **Why is it important to complete CRFs correctly and completely?**
    - It reduces the burden of finding and correcting missing or incorrectly stored data values and pages.
    - It reduces the number of QC queries that must be researched and resolved.
    - It helps to ensure that the data stored in the database is correct!

9.1.3.6 *CRF Scanning*

1. Set your scanner to:
    - Scan to A4 format
    - Scan to "actual size" (no "shrink to fit" or "expand to fit")
    - Scan at 150 / 200 dpi (dots per inch)
    - Scan to "black & white"

2. Feed pages into the Scanner at Right Angles
   REMEMBER: Set the feeder tray guides to A4 and feed the CRF pages into the Scanner at right angles – from the head of the page to the foot of the page.

3. Skewed / Obstructed PDF Images also go into the *Unidentified Fax Router*

Bad image:

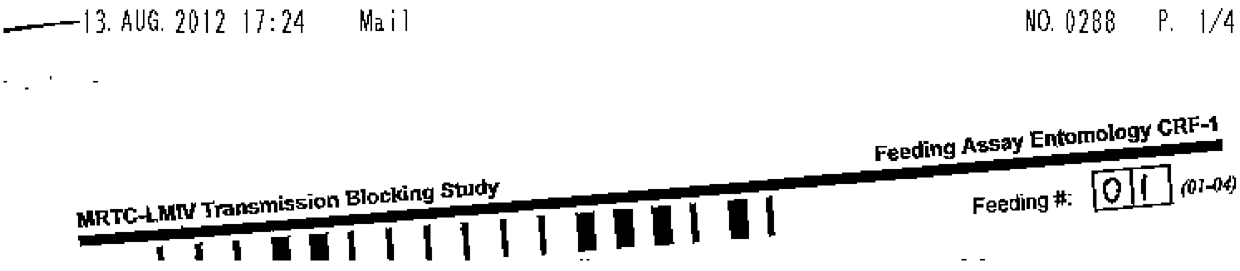
13. AUG. 2012 17:24 Mail NO. 0288 P. 1/4
MRTC-LMIV Transmission Blocking Study
Feeding Assay Entomology CRF-1
Feeding #: 01 (01-04)

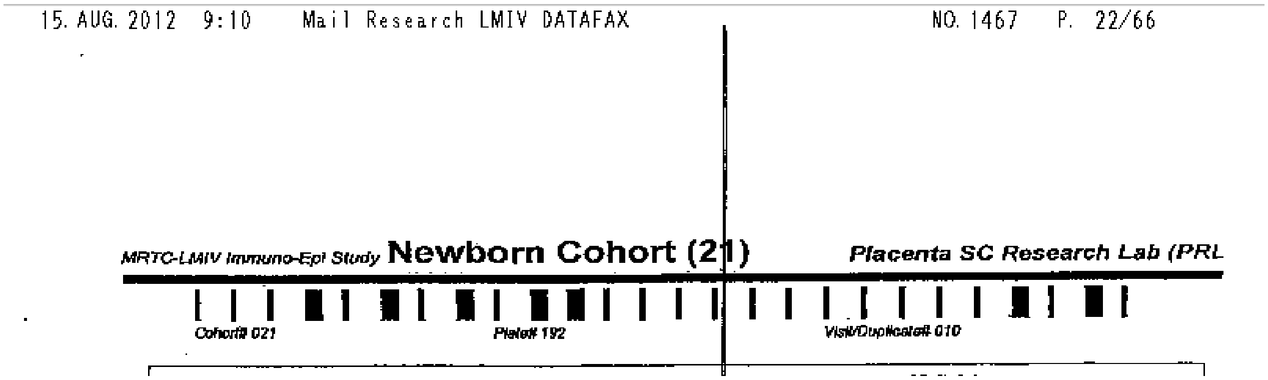
15. AUG. 2012 9:10 Mail Research LMIV DATAFAX NO. 1467 P. 22/66
MRTC-LMIV Immuno-Epi Study Newborn Cohort (21) Placenta SC Research Lab (PRL
Cohort# 021 Plate# 192 Visit/Duplicate# 010

Good image:

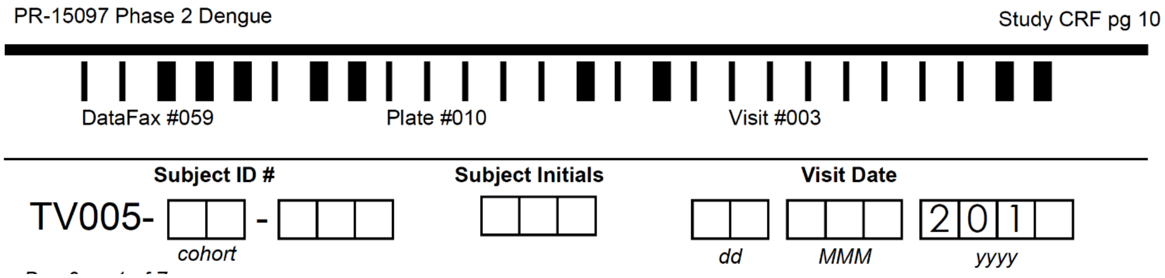
PR-15097 Phase 2 Dengue Study CRF pg 10
DataFax #059 Plate #010 Visit #003
Subject ID # Subject Initials Visit Date
TV005- - 201
cohort dd MMM yyyy

9.1.4 Database: DataFax

DataFax is a 21 CFR part 11 compliant clinical trial data management system. iDataFax is the user client and part of DataFax system. It is a standalone computer program that does not rely on a specific web browser. It can be downloaded and run on your local PC. There are two types of data entry methods:

- Paper based clinical research environment – the study will primarily be using this method. Scanned CRFs will be recognized by ICR first, and designated data entry/personnel will review data.
- Electronic Data Capture or EDC—users can enter, review and modify patient data, and to submit it over the internet to a DataFax server at NIH.

DataFax has built in quality control features. You can compare an electronic record to a scanned image of a paper CRF. It also enables real time edit checking to check missing, N/A, out of range values.

NIH DataFax support group is responsible for creating and maintaining user accounts for study users. If you need a user account, please contact TRS Data Manager Chrissie Cai caiy2@niaid.nih.gov first. She will authorize you and contact the DF support team to assign a user account for you. It is your responsibility to change your password every 90 days. If you have any question, you may contact NIAID OCICB DATAFAX SUPPORT datafax@niaid.nih.gov.

9.1.5 Data Entry and Submission Timelines

A centralized Data Management Center is responsible to do entry/review. It is crucial that data is being reviewed in a timely manner. The double data entry process will begin after CRFs are scanned and submitted to DataFax.

9.1.5.1 *Data Entry Level 1*

- Data must be entered within 2 days after scanned CRFs are received
- During first data entry records go from level 0 to level 1
- Select View, Fax View
- Newly scanned CRFs are listed
- Values that are not recognized by the Datafax's ICR program must be entered into the 'entry plate'
- Values recognized by datafax, need to be checked for accuracy
- When the data is checked it can be saved as final, incomplete or pending

9.1.5.2 *Data Entry Level 2*

- Second data entry must be done 24 hours after 1st entry is completed
- This is the main iDataFax Window, data is moved from level 1 to level 2
- Select View, Data
- Select "Select". Change Mode & Level
- Change mode to "Validate", change level to level 2
- Tab through the document and check for any discrepancies.
- The document can now be saved as Final, Incomplete or Pending
- Different icons and colors indicate the status of the patient data
- Green – No Problems
- Red – One or more pages are incomplete
- Yellow – One or more pages are pending

9.1.6 General Instructions for Data Entry

9.1.6.1 *Data Capture*

- The data capture should strictly follow a 'Type what you see' policy. All data should be captured in the database. All corrections should be initialed and dated by the investigator (or delegate).
- All data recorded in the Case Report Form (CRF) has to be available in predefined source data locations (e.g. patient's medical records, laboratory reports) or have to be the source data themselves.
- All the entries should be written in English and captured in English.

9.1.6.2 *Participant ID*

For DataFax, a participant will be uniquely identified by a 5-digit numeric number as described in sec. 9.1.3.1 above.

9.1.6.3 *Capital Letters*

All alpha-numeric data will be entered as capital letters.

9.1.6.4 *Date Formats*

The date will be captured in the order "Day/Month/Year", e.g. Apr. 01st must be: 01/APR/2016

- If the day value is unknown, this section should be captured as 00/APR/2016
- If the month value is unknown, "UNK", this section will be captured in the corresponding box: 01/000/2016
- If the day and month is unknown "UNK" this will be captured as 00/000/2016

9.1.6.5 *Numbers*

Numeric fields must be completed with numerals and decimal points, if applicable.

9.1.6.6 *Leading Zero's*

If any numeric field is recorded in the CRF without the appropriate leading zero, the data capturer will capture the zero in the database.

9.1.6.7 *Missing Value Codes*

Use these Missing Value Codes for fields that have missing reasons.

* Missing Value
.NA Not Applicable
.ND Not Done
.NR No Result
.R Refused
.UNK Unknown

9.1.7 Data Correction and Tracking Requirements

9.1.7.1 *QC Report*

Once a CRF image is received by DataFax, the DMC reviews the data, identifies problems (such as missing or inconsistent data, invalid data values, and/or illegible

data), and applies QC notes as necessary. The QC report may also show missing pages and overdue visits, based on protocol expectations. QC notes are compiled into study-specific QC reports that are sent via e-mail to the study site on a regular basis. The QC reports will also be stored on study SharePoint web portal. The QC reports are sent in Adobe Acrobat PDF format.

QC reports are sorted by subject ID. Each QC note includes the following:

- Participant ID
- Visit code (or page number, if CRF is a log form)
- CRF acronym
- Query text (if needed)
- Value that was written on the CRF by the site (if present)
- QC Problem Type

9.1.7.2 ***Correcting data and Resolving QC Notes***

Upon receipt of the QC report from the DMC, the Site Data Manager (SDM) reviews the report and contacts the appropriate staff to make the necessary corrections and/or clarify data. The SDM is responsible for responding and addressing/resolving all specific questions and re-transmitting the corrected CRF, indicating at the back of each re-transmitted CRFs that the page has been scanned and adding an initial and date. Thereafter, SDM/designee is responsible for replacing the CRFs in their appropriate folders organized by Participant IDs. If a correction is made to a portion of a multi-page form, only the pages with changes need to be sent to the DMC.

All CRF updates should be clearly updated (you should be able to still see the old value)

- Line through the old/previous value
- Provide the new/updated value
- Initial and date the change next to the new/updated value

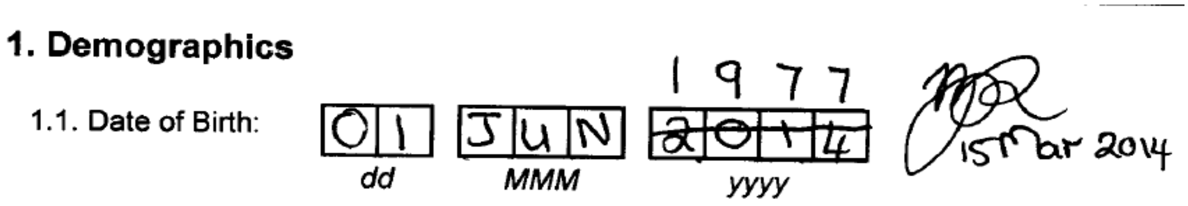

For the study database to accurately reflect the data captured on CRFs, CRFs must be re-transmitted immediately whenever data are added, changed, or removed from a CRF, regardless of whether the change was made in response to a QC report.

The updated CRF's will be uploaded by the SDM and identified as the "Primary Image" by DMC data manager. Some of the queries may resolve "automatically", if not, the following statuses may be used to resolve the query:

- Resolved NA – The query has been resolved – the requested data or correction is Not Available
- Resolved Irrelevant – The query has been resolved – the query was deemed to have been unnecessary
- Resolved Corrected – The query has been resolved – the requested correction of information has been received

Note: If corrected CRFs are not received and entered by the DMC before the next QC report is generated, the QC note(s) may appear again on subsequent QC reports.

9.1.7.3 *CRF Tracking and Resubmission*

While CRFs are being reviewed, it is important that they are stored and tracked systematically. It is also necessary to have a system to identify whether a CRF has been transmitted or not. For this study all CRFs should be printed double-sided and the back of the CRF includes sections to complete when reviewing and transmitting the CRF. Each time the CRF is transmitted, the back of the CRF should be updated to reflect this.

CRFs should be transmitted from the site to the Data Management Center as quickly as possible after completion, within 24 hours whenever possible. Any time a form is modified, it must be retransmitted to the data management center. Changes made to CRFs which are not retransmitted will not be reflected in the study database. Remember to remove paper clips and staples before transmitting. Transmit/re-transmit CRFs as soon as possible after they have been completed and reviewed.

Completed CRFs, responses to QC reports, or other requested data should be submitted by email to the DataFax server. The DMC will work with site staff to ensure that records can be transmitted.

The site should designate which staff members are responsible for transmitting CRFs to the DMC, and responding to QC reports. Rapid response to QC reports is essential for resolving training issues and consistent errors that affect data quality.

9.1.8 Data Entry and Submission Timelines

The DMC will review data twice upon receiving scanned CRFs from each site. DMC will run QC reports on a weekly basis. Queries will be sent to each site to resolve. The suggested timeline in days is below:

	**CRF Submission**	**CRF 1st Review**	**CRF 2nd Review**	**Resolving Queries**	**CRF resubmission**
Critical	1	1	1	3	1
Non-critical	1	2	2	5	1

9.1.9 Retention of Study Documentation

Federal regulation and International Conference on Harmonization (ICH) Good Clinical Practice (E-6) requires investigators and sponsors to retain specific study records associated with the conduct of clinical research. These documents are often referred to as Essential Documents. The general principles apply to all clinical research.

Investigators should be aware that they may also have local government or institutional policies for record retention. The most stringent requirement would apply.

10 Site Monitoring

10.1 Purpose

Site monitoring is the mechanism by which a clinical research program is formally evaluated to assess compliance with Good Clinical Practices (GCP) and the International Conference on Harmonisation (ICH) guidelines, as well as the Predict protocol itself. This process also determines the degree of accuracy and completeness of the data collected.

Site monitors, or clinical research associates (CRAs), are responsible for assessing the regulatory, clinical, laboratory, and pharmacy activities as well as documentation associated with the research protocol at each of the clinical sites. The monitor will schedule visits to the sites to review research records and to meet with the project research team.

The monitor is responsible for assisting clinical sites with continuous quality improvement and will offer objective and constructive feedback to the clinical site staff. The monitor will provide verbal and written site monitoring findings to the site monitored, the Office of Clinical Research Policy and Regulatory Operations (OCRPRO) at the NIH, and to the NIAID Study staff.

10.2 Scheduling the Site Monitoring Visit

Site monitoring visits are scheduled to occur according to the monitoring plan established by the NIAID Office of Clinical Research Policy and Regulatory Operations (OCRPRO) in conjunction with the contract research organization (CRO) that will monitor the study.

The monitor will arrange with the project study coordinator a mutually agreeable date(s) for the site visit. The study coordinator will ascertain that the time is also convenient for the Principal Investigator (PI) and other key staff. The monitor will request that the study coordinator to be available during the visit to provide clarification and to assist the monitor, as needed; and the PI to be available toward the conclusion of the visit to discuss the monitor’s summary of findings and recommendations. The Site Pharmacist will be asked to be available for pharmacy audits; as well as availability of laboratory personnel for microbiological laboratory reviews.

The monitor will confirm the site visit in a letter or email to the PI at least two weeks before the visit date. To ensure adequate preparation at the site, the letter will provide the site monitoring agenda and will include the monitoring objectives and an outline of all documents required for the monitor’s review. The confirmation letter will include a list of participant records that the monitor plans to review.

10.3 Components

10.3.1 Informed Consent Verification

During the site monitoring visit, the following aspects of the informed consent process will be verified for 100% of enrolled participants at all sites:

- The consent form version most recently approved by the Institutional Review Board (IRB) or Independent Ethics Committee (IEC) was used.

- The informed consent form (ICF) and other related information were provided in a language understandable to the participant. Note: Ensure sufficient documentation regarding how the study and consent information was communicated, e.g. for non-English speakers, via the use of an in-person interpreter, etc.

- The ICF was signed prior to implementing study-related activities. It is recommended that the time the participant signed the ICF be included in documentation of the consent process. This will support confirmation that no study-related activities were completed prior to consent. This is particularly important for cases where the ICF is signed on the same date as the start of study activities. The time is also documented on the ICF.

- The ICF was signed and dated by the participant.

- The ICF was signed and dated by the person obtaining consent and the PI (if required by the local IRB/IEC).

- The ICF was properly witnessed, if applicable, as for short form consents and/or per local requirements.

  Signatures and dates of each individual were written at the same time by the particular signer and that each individual dated his/her own signature. If the participant forgets to date his/her signature, it is recommended that the omission be documented in a progress note or other notation mechanism (not on the ICF), describing the circumstances. According to GCP, no one else should date in lieu of the participant, nor correct a date written by the participant.

- The source documentation briefly describes the informed consent process and confirms the participant was offered a copy of the signed ICF. If the participant refuses a copy of the signed ICF, include source documentation indicating that the participant refused a copy.

10.3.2 Other Data Reporting

At each visit, the following activities will occur:

- A predetermined percentage of randomized records will be reviewed for maintenance of confidential records, adverse event (AE) and serious adverse event (SAE) monitoring and reporting, and adherence to the protocol’s schedule of evaluations.

- A predetermined percentage of data points critical to the outcome of the study will be reviewed for completion and accuracy.
- A predetermined percentage of stored samples collected will be reviewed to determine if collected and stored properly.
- The site's current missing forms, forms requiring verification, data query reports and other requests for corrections, and missing sample reports will be discussed with the site staff and a plan to address and complete these tasks will be established and implemented.

10.3.3 Findings

Findings will be discussed with the team at the monitoring visit summary meeting. A summary letter will be drafted and sent to the site and the Sponsor. A corrective action plan should be put in place for needed items and these items will be reviewed at the next monitoring visit.

11 Laboratory Sample Processing Overview

See Laboratory MOP for full details.

11.1 Overall sample collection schedule

	Screen	D0	W1	W2	W4	W8	W12	W16	W20	W24	W36	W48	W72	RECUR
**Sputum 1 Culture, Xpert (X)**	X	X[1]			X	X		X		X				X
**Sputum 2 TRIzol**														
**Sputum 3 Biomarkers LAM (L)**		L			L	L		L		L			L	L
**Safety Blood**														
**Pregnancy test**	Serum	Urine			Urine			Urine[2]		Urine[2]				Urine
**Blood Biomarkers**														
**Urine Biomarkers**														
**Saliva Biomarkers**								2		2				
**Blood PK**							3	3	3	3				
**Blood Drug monitoring**														
**Blood Host DNA**														

[1] *Only performed if screening GeneXpert was >7 days prior*
[2] *Performed at W16 or W24 based on randomization*
[3] *Two PK visits at any time between W12 and W24*

11.2 Sputum collection schedule and order of sputum processing

Up to 3 sputum samples will be collected following the collection schedule below.

- Sputum 1, for culture and Xpert test

  - **Sputum 1 must be collected at every visit**.

  If there is volume remaining, then it can be further used for XpertUltra and LAM (in that order).

  - Expectorated

  - Overnight or spot collection

  - More than 3 or 4 ml, depending on the visit

- Sputum 2, for mRNA (+ TRIzol)
  - Expectorated or induced
  - *Spot collection*
  - 1 - 1.5 ml
- Sputum 3, for biomarkers
  - Expectorated or induced
  - 3 ml

	Screen	D0	W1	W2	W4	W8	W12	W16	W20	W24	W36	W48	W72	RECUR
**Sputum 1 Culture, Xpert**	4 ml	4 ml	3 ml	3 ml	4 ml	4 ml	3 ml	4 ml	3 ml	4 ml	3 ml	3 ml	4 ml	4 ml
**Sputum 2 TRIzol**		1.5 ml		1.5 ml	1.5 ml	1.5 ml		1.5 ml		1.5 ml				1.5 ml
**Sputum 3 Biomarkers**		3 ml		3 ml	3 ml	3 ml		3 ml		3 ml		3 ml	3 ml	3 ml

11.3 Sputum collection procedures

Obtaining high quality sputum without saliva contamination is the goal. Explain to the participant to rinse his/her mouth with water to remove the saliva. If he/she cannot do this, then ask him/her to spit out the saliva in his/her mouth as much as possible before expectorating. Follow site-specific procedures for where the patient expectorates the sputum.

Label the sputum collection container with identifying label including PID, specimen type, and date and blank for time of collection. Provide the container to the participant and instruct him in sputum collection as described below. A bar coded label may also be added to the container.

Instruct the participant not to expectorate saliva or postnasal discharge into the container but rather specimen resulting from a deep cough into a sterile screw-cap cup or sterile sputum collection container. The participant should continue trying to expectorate until the required volume of sputum has been collected (there is not a time requirement other than before the visit is over). Show the mark on the container(s) to the participant and instruct the participant to place the sputum specimen container in the biohazard specimen bag.

The container for Sputum 1 is given to the participant at the previous visit and he/she is asked to collect the sputum during the night before the next visit and bring the collected sputum on the day of the visit. He/she must be reminded the day before the visit. Instruct the patient to follow precautions to avoid exposure of other household members. Preferably, the specimen should be collected outside of the dwelling. The participant should fill the cup to the intended volume even if this requires multiple collections into the same cup.

If the participant fails to bring Sputum 1, it must be collected during the visit. Sputum 1 must be collected at every visit.

Sputum induction, according to the site-specific SOP, should be strongly considered for participants who are no longer able to expectorate sputum spontaneously, particularly at weeks 16, 24, 48, and 72, for Sputum 2 and Sputum 3.

If for any reason, during sputum collection at the site, the staff member needs to enter the room to assist the patient, the staff member should put on personal protective equipment (PPE) properly, including a N95 mask, disposable gloves, and a protective labcoat. The staff member should not remove the mask until he/she has left the space where the participant is coughing.

The staff member must put on gloves to receive specimens from participant. Place the sputum collection container(s) 1 and 3 in the designated refrigerator or cold box, until it is delivered to the laboratory (do not freeze).

Sputum 2 is stored at room temperature before mixing with Trizol, until it is delivered to the laboratory. After mixing with Trizol, the specimen should be maintained on ice until frozen.

11.4 Safety Bloods

Safety bloods will be processed by an accredited lab of choice for each individual site. Tests will be done per standard of care for each site.

For results that are out of range, staff should confirm whether the out of range results indicate the participant is not eligible for the study. For safety bloods during the study, test results should be reported to the study doctor who should follow standard site procedure for further testing or monitoring.

The following tests will be requested as Safety Bloods:

Safety Screening Blood

<table>
<tr><th></th><th>Tube</th><th>Volume</th></tr>
<tr><td>AST</td><td rowspan="4">1 x Serum tube: Red top
<u>Or</u>
1 x Serum tube: Gold top
(preferred by NHLS)</td><td rowspan="5">5mL</td></tr>
<tr><td>ALT</td></tr>
<tr><td>HIV</td></tr>
<tr><td>Creatinine</td></tr>
<tr><td>ß-hCG (Pregnancy test)</td><td>Serum (same tube as above) or urine</td></tr>
<tr><td>Full Blood Count</td><td rowspan="2">EDTA: Purple top</td><td rowspan="2">4mL</td></tr>
<tr><td>HbA1c</td></tr>
</table>

Pregnancy testing on serum is preferred at Screening however; urine testing can be performed if serum testing is not available. All required timepoints after Screening will be done on urine. Any urine pregnancy test kit that is approved by the local regulatory authority may be used.

11.5 Biomarker Sample Collection

Biomarker sample collection

	Tube	Volume	Number of tubes
**Serum**	Red top Serum tube - no gel	4mL	2
**Host mRNA**	PAXGene tube	2.5 mL	2
**Pharmacokinetics**	EDTA	1-2mL	4 (1 each timepoint)
**Host DNA**	EDTA	4mL	1
*** Whole blood (PBMC's optional)**	Sodium Heparin	10mL	4
**Urine**	Urine specimen container	5 - 20mL	1
**Saliva**	Salivettes	1mL	3
**Drug Monitoring**	EDTA	1-2mL	1

*This is an optional assay for sites.

ORDER OF SAMPLE COLLECTION

Sample collection should occur in the following order:

1. Collected in any order:
    a. Spontaneous sputum
    b. Urine
    c. Blood
    d. Saliva – Must be fasting for a minimum of 1 hour prior to collection
2. Induced sputum – Always collected last and never before spontaneous sputum sample collection.

The following order of blood draw should be considered when performing phlebotomy on participants:

1. Serum (Safety blood) (*)
2. Serum (Biomarker)
3. Whole blood (Na Hep – optional PBMC's, not included in MOP)
4. EDTA 4mL (Safety bloods) (*)
5. EDTA 2mL/4mL (Biomarker – Host DNA, Drug Monitoring)
6. PAX tubes

** Please note: The safety blood samples are only performed routinely during the Screening visit and Recurrence and therefore should not normally coincide with the biomarker blood collection. However; if clinically indicated, the study doctor may request a repeat of safety blood samples at any timepoint which would result in phlebotomy of both safety blood and biomarker blood samples simultaneously.*

CONSUMABLES AND REAGENTS:

- Blood collection equipment
- Sputum/urine jars
- 4mL EDTA tubes (BD cat # 367861) – Host DNA and Safety blood
- 2mL EDTA tubes (BD cat # 367842) – PK Samples
- PAXgene tubes (BD cat # 762165)
- Red top serum tubes, 5mL/6mL (BD cat # 367814/367815) – Safety blood and biomarker serum
- Sodium Heparin tubes, 10mL (BD cat # 367874) – PBMC's (Optional)
- Sarstedt Salivettes (Biodex cat # 51.1534) - Saliva
- Standard personal protective equipment

Please note: Catalogue numbers may vary depending on the country they are being ordered from. Please ensure the description of the tube is correct before ordering tubes with varying catalogue numbers. Please consult the BD website for more information.

SERUM COLLECTION PROCEDURES:

- Only red top, non-gel serum tubes should be used for biomarker sample collection. Gold top, SST tubes may be used for ONLY safety bloods.
- Blood tubes should be inverted after blood draw to mix the blood with the clot activator.

PAXGene COLLECTION PROCEDURES:

- PaxGene tubes contain a stabilization buffer thus the tube will not be empty before the blood draw. Please draw blood directly into the tube and do not discard the buffer before blood draw.
- The volume of blood collected is 2.5mL.

URINE COLLECTION PROCEDURES

Urine collection provides samples for pregnancy testing and aliquots for biomarker assay groups. Clean catch is not essential for assay groups, but encouraged.

- Provide participant with ID-labeled urine container.
- Explain the clean-catch method:
  Participant goes to clinic cloakroom/toilet. When in the stall, participant cleans the area of the urethral opening (where urine passes) with paper towel(s) and water. Female participants are to wipe from front to back at least twice with two separate wet towels. Subsequently, participant starts to pass urine, stops midway, and then catches the "mid stream sample" in the container supplied (aim for 5 to 20 mL). Midstream collection is not absolutely necessary, but encouraged. participant must replace lid of container and take it back to the study staff.
- Ask participant to void at a convenient point during the visit.

- Store all the labeled sample in a refrigerator or in a laboratory cooler at 2-8°C until processing for freezing (within the work day).
- Pregnancy testing should follow the test's instructions or clinical SOP.

SALIVA COLLECTION PROCEDURES

Collect saliva at the scheduled visit (Enrolment, Week 4, Week 16/24 and Recurrence).

Saliva is collected from 3 salivettes for each patient at each visit.

These do not need to be sequential but should be completed before the participant receives food.

- Label the salivette tubes with patient ID, name, date and time of collection (temporary labels- no bar code required)
- Record when the participant last ate or drank
- Remove the swab from the first salivette
- Place the swab in the mouth of the participant with tweezers
- The participant chews the swab for 45 seconds to stimulate salivation
- Collect the swab with tweezers
- Place the swab back into the salivette tube
- Store the salivettes on wet ice until taken to the lab

Repeat collection of saliva twice more, timing according to the comfort of the patient

11.6 PK SUBSTUDY BLOOD COLLECTION AND HANDLING

Notes on timing of blood collection and TB drug administration

- Participants must arrive for the study visit without having taken their TB drug dose for that day or the study must be delayed to another day.
- Windows for the blood draws are +/- 10 min for the 1 and 2 hour draws and +/- 20 min for the 6 hour draw.
- It is best to process blood into plasma within 2 hours but preferably 30 minutes, so the processing will have to take place at the site during the time when the samples are being collected.
- Normal procedures and precautions for drawing blood should be followed

Procedure for timing of blood collection and TB drug administration

- Record the time TB medication was taken (by patient report or check the pill box opening times) for the two previous days
- Using a Lavender top [EDTA] tube, collect 2 mL of blood, record the time
- Have the subject take their INH/RIF dose and record the time
- Place the tube on wet ice, in cooler box, or refrigerate (2-8°C) immediately.
- Process or pass on the blood for processing
- Draw 3 additional blood samples (approximately 1-2 mL each) into Lavender top tubes at approximately 1, 2, and 6 hours post-dose, recording the time and holding the tubes on ice or refrigerate (2-8°C) immediately.

12 APPENDIX A: MERM DOCUMENTATION

12.1 Participant ID/activation date sticker

Sticker template formatted for Avery 5160 label sheet

**Participant ID:** ________________<br>**Activation Date:** _____________	**Participant ID:** ________________<br>**Activation Date:** _____________	**Participant ID:** ________________<br>**Activation Date:** _____________
**Participant ID:** ________________<br>**Activation Date:** _____________	**Participant ID:** ________________<br>**Activation Date:** _____________	**Participant ID:** ________________<br>**Activation Date:** _____________
**Participant ID:** ________________<br>**Activation Date:** _____________	**Participant ID:** ________________<br>**Activation Date:** _____________	**Participant ID:** ________________<br>**Activation Date:** _____________
**Participant ID:** ________________<br>**Activation Date:** _____________	**Participant ID:** ________________<br>**Activation Date:** _____________	**Participant ID:** ________________<br>**Activation Date:** _____________
**Participant ID:** ________________<br>**Activation Date:** _____________	**Participant ID:** ________________<br>**Activation Date:** _____________	**Participant ID:** ________________<br>**Activation Date:** _____________
**Participant ID:** ________________<br>**Activation Date:** _____________	**Participant ID:** ________________<br>**Activation Date:** _____________	**Participant ID:** ________________<br>**Activation Date:** _____________
**Participant ID:** ________________<br>**Activation Date:** _____________	**Participant ID:** ________________<br>**Activation Date:** _____________	**Participant ID:** ________________<br>**Activation Date:** _____________
**Participant ID:** ________________<br>**Activation Date:** _____________	**Participant ID:** ________________<br>**Activation Date:** _____________	**Participant ID:** ________________<br>**Activation Date:** _____________
**Participant ID:** ________________<br>**Activation Date:** _____________	**Participant ID:** ________________<br>**Activation Date:** _____________	**Participant ID:** ________________<br>**Activation Date:** _____________

**Participant ID: ________________**<br><br>**Activation Date: _____________**	**Participant ID: ________________**<br><br>**Activation Date: _____________**	**Participant ID: ________________**<br><br>**Activation Date: _____________**

12.2 "Do not remove this cartridge" sticker

Sticker template formatted for Avery 5160 label sheet

**DO NOT REMOVE THIS CARTRIDGE**		**DO NOT REMOVE THIS CARTRIDGE**		**DO NOT REMOVE THIS CARTRIDGE**
**DO NOT REMOVE THIS CARTRIDGE**		**DO NOT REMOVE THIS CARTRIDGE**		**DO NOT REMOVE THIS CARTRIDGE**
**DO NOT REMOVE THIS CARTRIDGE**		**DO NOT REMOVE THIS CARTRIDGE**		**DO NOT REMOVE THIS CARTRIDGE**
**DO NOT REMOVE THIS CARTRIDGE**		**DO NOT REMOVE THIS CARTRIDGE**		**DO NOT REMOVE THIS CARTRIDGE**
**DO NOT REMOVE THIS CARTRIDGE**		**DO NOT REMOVE THIS CARTRIDGE**		**DO NOT REMOVE THIS CARTRIDGE**
**DO NOT REMOVE THIS CARTRIDGE**		**DO NOT REMOVE THIS CARTRIDGE**		**DO NOT REMOVE THIS CARTRIDGE**
**DO NOT REMOVE THIS CARTRIDGE**		**DO NOT REMOVE THIS CARTRIDGE**		**DO NOT REMOVE THIS CARTRIDGE**
**DO NOT REMOVE THIS CARTRIDGE**		**DO NOT REMOVE THIS CARTRIDGE**		**DO NOT REMOVE THIS CARTRIDGE**

**DO NOT REMOVE THIS CARTRIDGE**		**DO NOT REMOVE THIS CARTRIDGE**		**DO NOT REMOVE THIS CARTRIDGE**
**DO NOT REMOVE THIS CARTRIDGE**		**DO NOT REMOVE THIS CARTRIDGE**		**DO NOT REMOVE THIS CARTRIDGE**

12.3 Reminder sticker with site address and contact information

Sticker template formatted for Avery 5389 or 5889 label sheet

MEDICATION EVENT REMINDER MONITOR (MERM) BOX

Participant ID:

PLEASE REMEMBER TO **BRING THIS BOX WITH YOU FOR YOUR APPOINTMENTS!**

PLEASE REMEMBER TO TAKE YOUR MEDICATION ON TIME DAILY!

FOR ANY QUESTIONS/CONCERNS PLEASE CONTACT:

Address of the site:

Name of contact:
Contact's phone number:
Contact's email:
After hours phone number (if available):
This box remains the property of the study team at (Name of site)

MEDICATION EVENT REMINDER MONITOR (MERM) BOX

Participant ID:

PLEASE REMEMBER TO **BRING THIS BOX WITH YOU FOR YOUR APPOINTMENTS!**

PLEASE REMEMBER TO TAKE YOUR MEDICATION ON TIME DAILY!

FOR ANY QUESTIONS/CONCERNS PLEASE CONTACT:

Address of the site:

Name of contact:
Contact's phone number:
Contact's email:
After hours phone number (if available):
This box remains the property of the study team at (Name of site)

12.4 Participant instruction sheet

This is NIH version 1.0, dated May 26, 2017. Individual sites may have a different version number or date.

Medication Event Reminder Monitor (MERM) Instructions

1. Your study doctor will tell you when to start taking your medication. It is best if you take your medication around the same time every day. The MERM will beep automatically each day at the set time and the green light will flash. This reminds you to take your medication.

Green reminder light

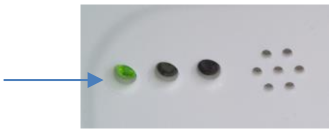

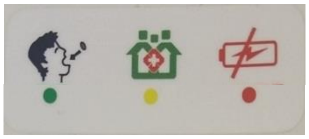

2. Open the MERM (you will hear a beep) and look at the medication amount label on the inside of the box. Remove the medication, take out the amount to be taken, **swallow the medication,** and put the rest of the medication back into the MERM box. Then close the box completely. You will hear a beep telling you the box is closed.

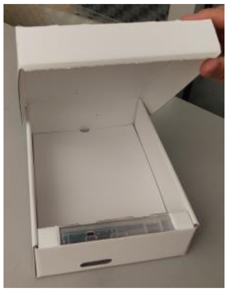

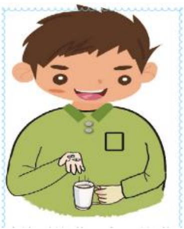

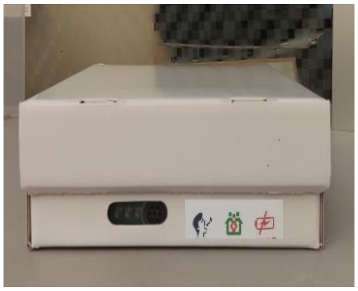

3. You do not have to wait for the box to remind you to take the medication! You can take your medication before the box reminds you, as long as it is around the time you take your medication every day. If you take your medication before it beeps that day, it will not beep until the next day.
4. Keep all empty medicine packets in the box to bring with you to your next visit.
5. A yellow light will flash on the day of your next clinic study visit. Please remember to bring your box with you to every visit.
6. A red light will appear whenever the box lid is opened. The red light will also flash if your battery is getting low.
7. If you have any questions about the MERM, please ask a member of the study team. We are here to help you!

We hope you enjoy using the MERM and that it helps you remember to take your medication every day!

12.5 MERM User Guide

This is version 1.0, dated 31 May 2017.

PredictTB MERM User Guide

Release 1.0.0.5

Created by

Office of Cyber Infrastructure and Computational Biology

for the

Tuberculosis Research Section
Laboratory of Clinical Infectious Diseases

National Institute of Allergy and Infectious Diseases
NIH

31 May 2017

Table of Contents

1. About this User Guide 107
2. MERM Software Installation 107
3. Overview of the MERM 107
4. MERM Software Interface 111
5. Understanding the Menu 112
6. MERM Setup / Laptop (or Desktop) Connection 113
7. Baseline Visit Instructions 114
8. Follow-up Visit Instructions 115
9. Drug Adherence Graph 116
10. Data Transfers 117
11. Upload Log File 117
12. User Support 118

1. About this User Guide

The user guide is intended for Predict study team members using the Medication Event Reminder Monitor (MERM) system for drug adherence monitoring. This user guide is an instructional document covering some of the technical aspects regarding the MERM as well as the MERM software interface.

This user guide is not intended for study participants taking part in the Predict trial.

This user guide has been developed for the MERM release 1.0.0.5.

2. MERM Software Installation

It is important that the MERM system is installed on a Windows laptop (or desktop). The software will not be operational in the Mac operating system.

- Unzip the file named NIAID MERM.exe
- The following three folders will be unzipped:
  - MERM
  - PreInstallation
  - Original_MERM
- Refer to the PreInstallation folder – Pre-installation instruction documents are available (depending if MERM installation is to be done on a Windows 7 or Windows 8 operating system).
- After successful completion of all steps in the Pre-installation document, the MERM system can be opened from the MERM folder. Run the Merm file (Application) to open the MERM system.

3. Overview of the MERM

The MERM is used to (i) store medication, (ii) issue medication reminders, (iii) record dosing history, and (iv) issue refill medication reminders (next visit date).

The following visual and audible alerts are applicable:

- Daily medication reminder: green light-emitting diode (LED) & buzzer
- Medication refill reminder: yellow LED
- Low battery alert: red LED

MERM adherence data will be automatically sent to the Data Management Centre, however visual cues are provided in the MERM system and will be discussed in later sections (Section 10).

Daily Medication Reminder: Green LED and Buzzer

- The medication reminding period is initiated at the pre-programmed time as set by the health care provider using the MERM system front-end.
- If the MERM container is opened at any point during this medication reminding period, the alarms are cancelled until the next day.
- If the MERM container is opened within four hours before the medication reminding period, the alarms are cancelled until the next day.
- During alert periods, the green LED will flash as described.

Daily Medication Reminder Alert Steps

The medication reminding period lasts for a total of 30 minutes as follows:

1. First 5 minutes: the green LED will flash + the buzzer will sound
2. Next 5 minutes: all alerts will stay OFF (no flash or sound)
3. Next 5 minutes: the green LED will flash + the buzzer will sound
4. Next 5 minutes: all alerts will stay OFF (no flash or sound)
5. Next 5 minutes: the green LED will flash + the buzzer will sound
6. Next 5 minutes: all alerts will stay OFF (no flash or sound)

After 30 minutes, the alarm will end and no further alerts will occur until the next day

Dosing Reminder: Green LED and Buzzer – Participant Use Steps

1. At the pre-programmed time, the green LED lights and the buzzer sounds to alert the participant of the dosing time.	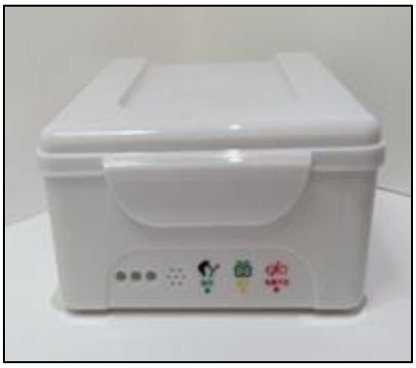

2. The participant opens the MERM container by opening the box to access the stored medication.	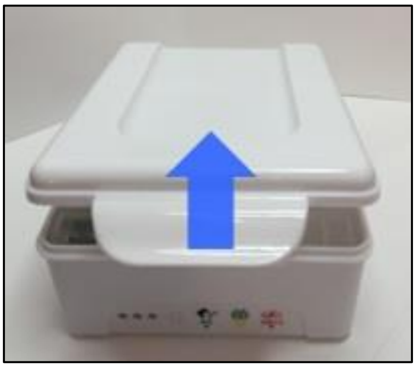
3. The participant follows the medication-taking instructions on the label inside the MERM container lid.	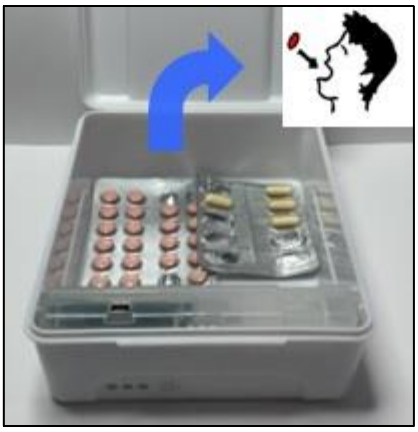
4. After taking the medication, the participant closes the box and stores the MERM to be used again the next day.	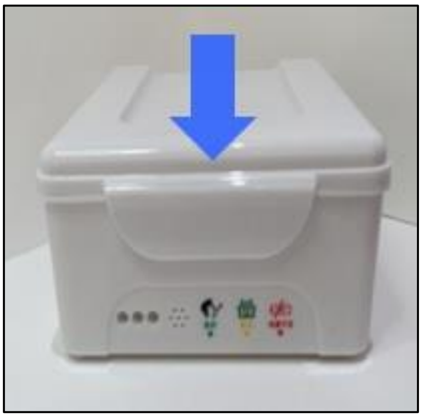

Medication Refill Reminder: Yellow LED

The medication refill alert is initiated on the pre-programmed date in the MERM software front-end (as set by the health center) and every day following until participant goes to the clinic. The health center will then refill the MERM container and set the alarm to the next visit date. Alert steps occur as outlined.

Refill Reminder Alert Steps:

If the medication refill date has been reached, the refill reminder will be activated and alerts will occur in the following instances:

1. The yellow LED will flash continuously for 30 minutes during the Medication Reminding Period. Once the medication reminding period ends, the yellow LED will turn OFF.
2. The yellow LED will turn ON in any instance that the MERM container lid is opened, regardless of whether or not it is during the medication reminding period. The yellow LED will turn OFF once the MERM container lid is closed.

NOTE: If the MERM container is accessed within four hours before the medication reminding period – even though the dosing reminders are turned off for the day – the refill reminder stays active and will alert as outlined above.

Low Battery Alert: Red LED

The MERM device measures the combined voltage of the batteries and will alert via the red LED when a battery change is required. When the combined voltage is 2000 mV or less, the low battery alert is activated and occurs as outlined.

Low Battery Alert Steps:

1. The red LED will flash continuously for 30 minutes during the medication reminding period. Once the medication reminding period ends, the red LED light will turn OFF.
2. The red LED will turn ON in any instance that the MERM container lid is opened, regardless of whether or not it is the medication reminding period. The red LED will turn OFF once the MERM container lid is closed.

NOTE: If the MERM container is accessed less than four hours before the medication reminding period – even though the dosing reminders are turned off for the day – the low battery alert stays active and will occur as outlined above.

4. MERM Software Interface

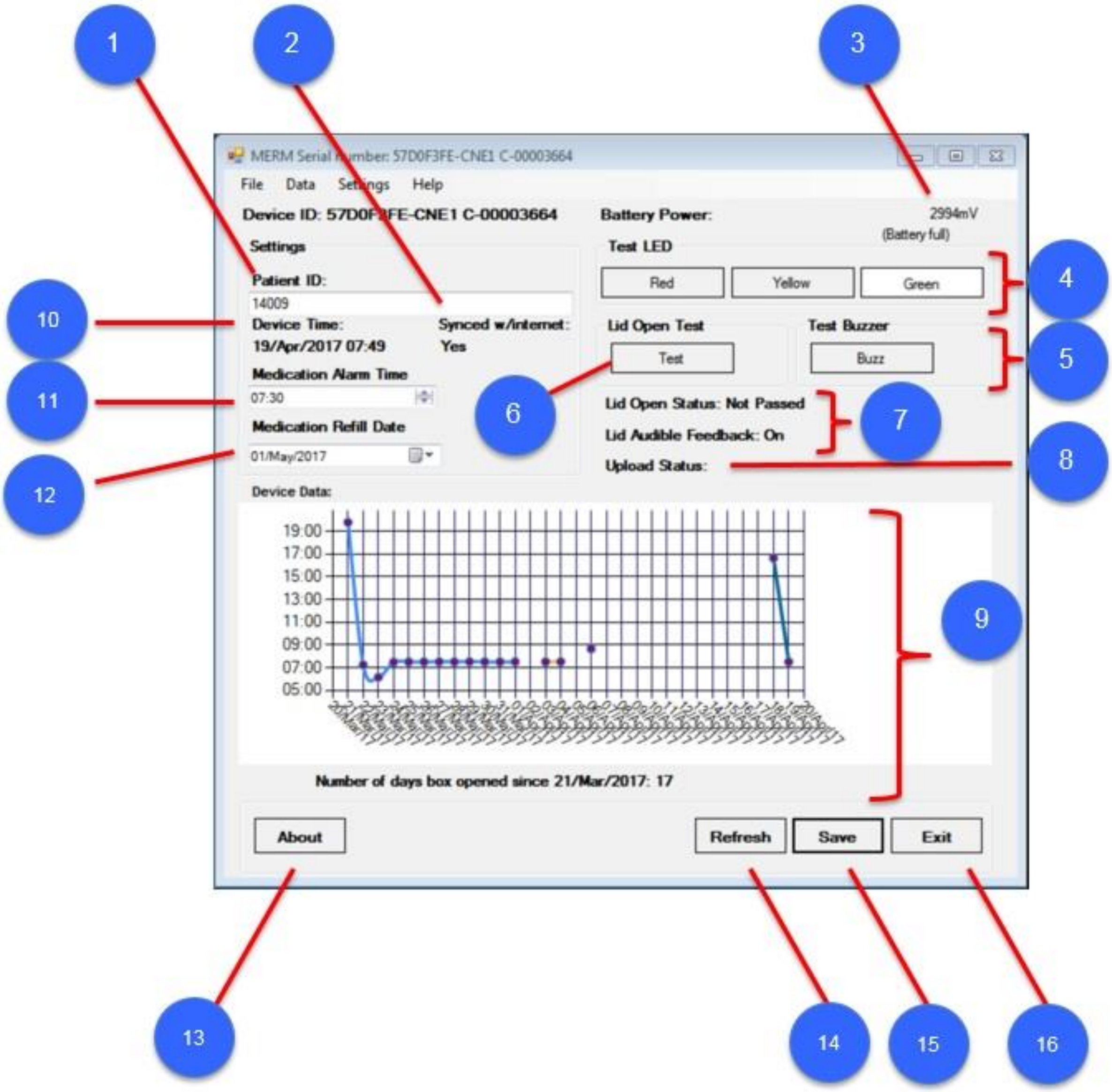

1. Participant ID	Entered by Predict Study team member. (Field is blank at Baseline.)
2. Synced w/internet	Indication if the MERM device time has been synched with the Internet (ensuring the Internet time). Ensure that laptop (or desktop) is connected to the Internet when using the MERM.
3. Battery Power	Battery power indicator in mV. Battery will either be indicated as “Battery (full)” or “Replace battery”.
4. LED Test buttons	Testing for red, yellow, and green LED lights
5. Test Buzzer	Testing the buzz audio
6. Lid Open Test	Testing that when the MERM Lid is opened the needed data recordings are performed

7. Lid Open Status	Indicator if test passed. Lid Audible Feedback will always be "On".
8. Upload Status	Indicates if any data uploads are pending or successfully completed (more information in section 10)
9. Device Data	Drug Adherence graph. Section will be blank at Baseline; however, at follow-up visits the graph will be populated. Note indicator (counter) at bottom of section showing how many days the box was opened since a specific date. (The counter only indicates unique days, not times, the MERM box was opened.)
10. Device Time	Shows the device time; this should be the same as the current time in your region.
11. Medication Alarm Time	Entered by Predict study team member at Baseline and updated at follow-up visits (if required). The entered time is when the MERM will alert the participant to take the required study medication (green LED + buzzer).
12. Medication Refill Date	Entered by Predict study team member at Baseline and updated at follow-up visits. The entered date is when the participant should return to the clinic for the next Predict study visit (yellow LED).
13. About button	
14. Refresh button	Performing a refresh of the data on the MERM to the software
15. Save button	
16. Exit button	

5. Understanding the Menu

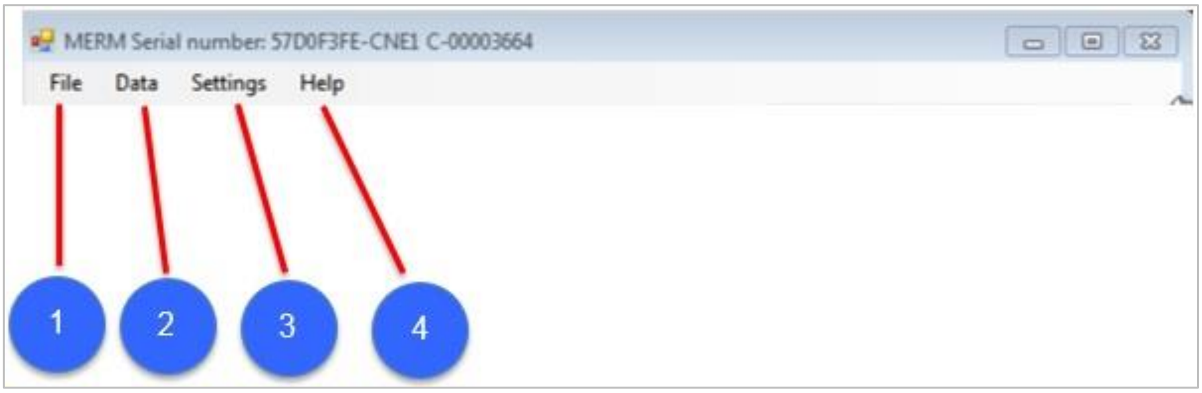

1. File	Allows "Refresh", "Save", or "Exit" the MERM

2. Data	Allows for the manual transfer of data in case there are problems with the automatic data transfers. This function is not expected to be part of the day-to-day operations. More information in section 10.
3. Settings	Currently, MERM software is available only in English. Language will be changeable between English and Chinese in a future version.
4. Help	Ability to view the logs, as well as the MERM software “About” screen

6. MERM Setup / Laptop (or Desktop) Connection

- Ensure two AA alkaline batteries (preferred make Energizer E91) are inserted into the MERM cartridge
- The MERM box needs to be constructed – for detailed guidance please refer to the following video instruction guidelines:

  Step 1: https://www.youtube.com/watch?v=sZbvvwAZXKg
  Step 2: https://www.youtube.com/watch?v=BC0BYa4PrM0
  Step 3: https://www.youtube.com/watch?v=KtwV9G0SW0w
  Step 4: https://www.youtube.com/watch?v=LzEIGV-xoIl

- Ensure that the various MERM labels/stickers are attached to the MERM box. They include:
  - MERM General label: Place on the flip-top (either outside or inside the flip-top) with the site specific details ompleted for your site. Make sure that all relevant site details are completed on the label.
  - Cartridge label: Sticked onto the MERM plastic cardige indicating the Participant number and the MERM cartridge activation/setup date (to ensure that the cartridge is assigned to a specific participant).
  - Do Not Remove label: Sticked securely across the MERM cartridge to clearly indicate that the MERM cartridge is not to be removed from the MERM housing.
  - Site specific DOT Card should be placed inside the MERM box (if applicable)
  - Short User instructions should be provided with the MERM box to the participant.
- Ensure the MERM cartridge is inserted the correct way into the cardboard box (*i.e.,* LED lights facing out). If not correct, it will not register the box opening/closing.
- Connect the MERM cartridge to the desktop or laptop with the provided USB cable. Only use the desktop or laptop on which the MERM software has been installed; otherwise, the application will not function.
- Ensure that the desktop or laptop being used is connected to the Internet.

- Run the Merm.exe application to open the MERM front-end.

7. Baseline Visit Instructions

Please note the first time connecting a new MERM cartridge to the Windows laptop or desktop a message will be displayed indicating that no records currently exist for the participant.

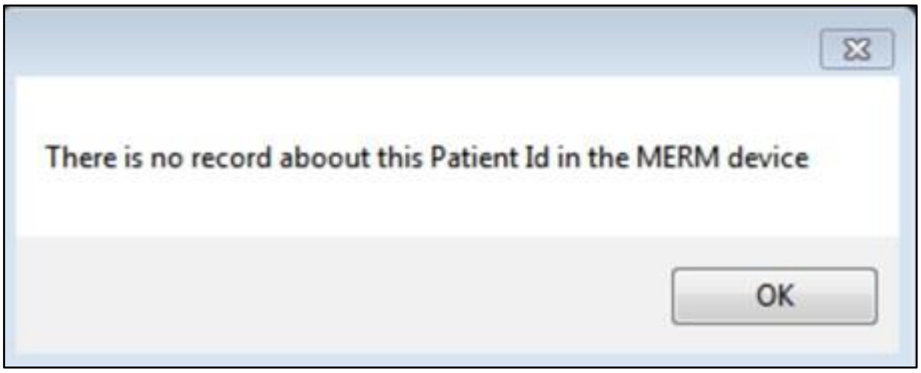

MERM Baseline Setup

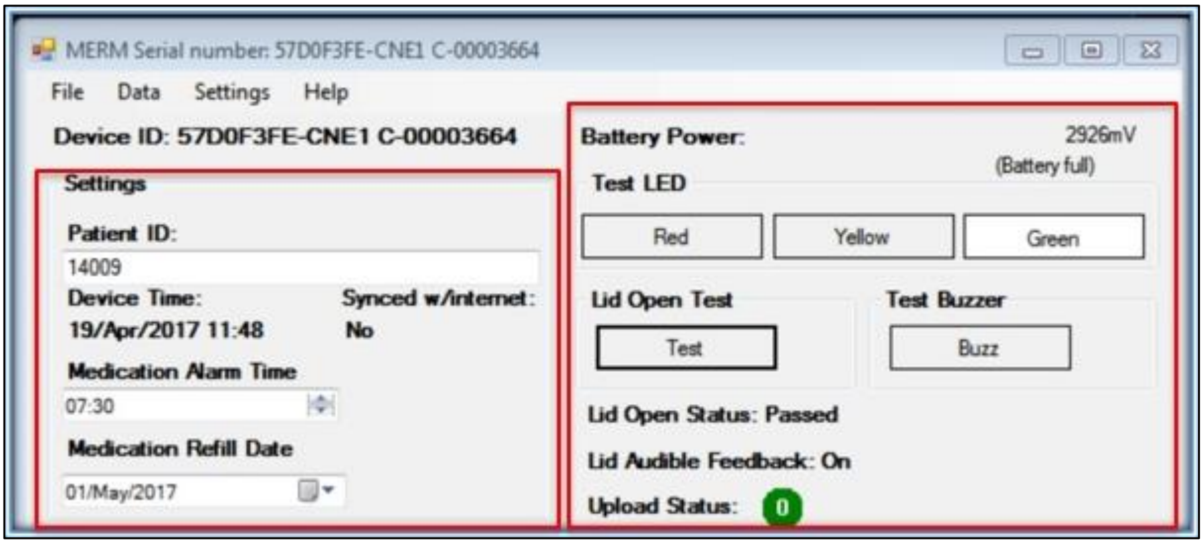

Various actions are to be completed at Baseline with regard to the MERM setup:

- Enter the Participant ID
- Device Time will automatically set to Internet time (the computer should be connected to the Internet)
- Set Medication Alarm Time – after discussion with participant and asking when the daily medication alarm is to be triggered
- Set the Medication Refill Date to the next visit date.
- Save

MERM Testing

- Ensure there is sufficient battery power. When the battery has less than 2500mV the MERM system will indicate to replace the battery. Please do so immediately at the current visit.
- Click the Red, Yellow, and Green buttons to test that the LED lights are functioning. (Please note for some new versions of the MERM cartridges the Green LED lights up automatically when the MERM cartridge is connected.)
- Click the Buzzer button to test the sound
- Perform the Lid Open Test
    - Disconnect the MERM box
    - Close the lid
    - Open the lid
    - Reconnect the MERM box via the USB cable
    - Click the Lid Open Test button
    - If successful, the “Lid Open Status” will indicate “Pass”
    - If unsuccessful, redo the test
- Click Save

8. Follow-up Visit Instructions

The following actions are to be completed at each Follow-up visit:

- Set Medication Alarm Time – Medication Alarm should be updated if required
- Set the Medication Refill Date to the next visit date
- Save

MERM testing

- Ensure there is sufficient battery power. When the battery has less than 2500mV the MERM system will indicate to replace the battery. Please do so immediately at the current visit.
- Click the Red, Yellow, and Green buttons to test that the LED lights are functioning. (Please note for some new versions of the MERM cartridges the Green LED lights up automatically when the MERM cartridge is connected.)
- Click the Buzzer button to test the sound
- Perform the Lid Open Test
    - Disconnect the MERM box
    - Close the lid
    - Open the lid
    - Reconnect the MERM box via the USB cable

- Click the Lid Open Test button
- If successful, the "Lid Open Status" will indicate "Pass"
- If unsuccessful, redo the test

- Click Save

Additionally the Drug Adherence graph will be populated showing the dates / times when the MERM box was opened (first opening of the day)

9. Drug Adherence Graph

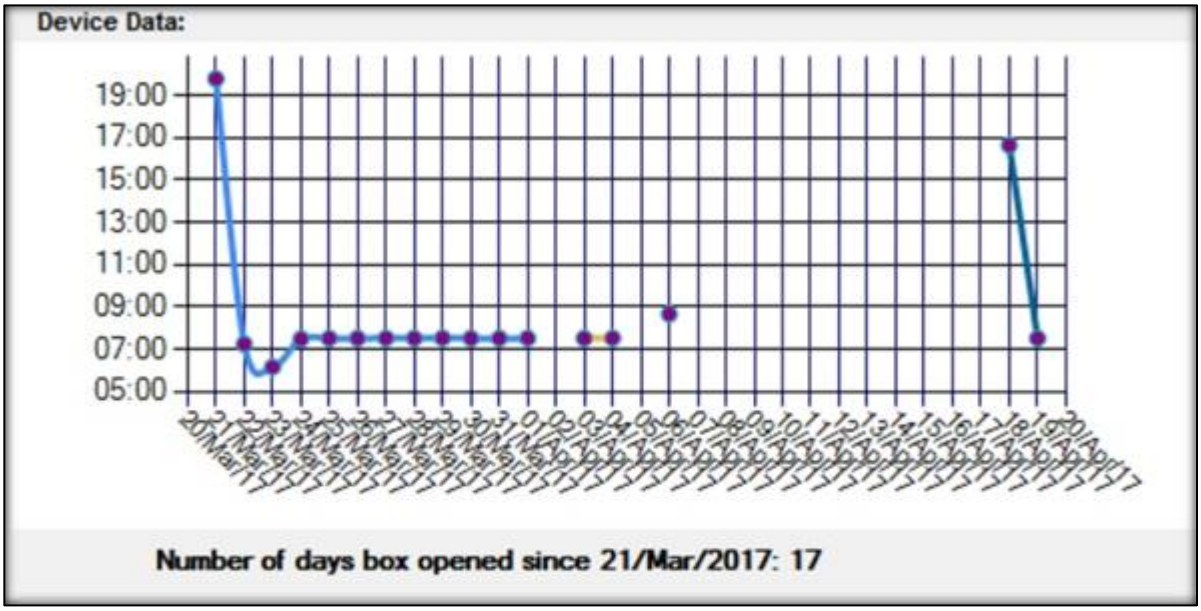

The Drug Adherence Graph will be displayed and will be populated starting from the first follow-up visit (please note the Device Data area will be blank at Baseline).

- The graph will show all days when the pill box was opened
- The line will be drawn in different colors if there are gaps in adherence (*i.e.,* when one or more days were missed)
- "Number of days box opened since ddMMMyyyy" will indicate how many days the box was successfully opened. The gaps must be counted to assess how many days the box was not opened.

It is important to understand that the drug adherence graph only shows the first time the container was opened on the day. Thus if the container was open (for any reason) in the morning, but usually the participant take his/her medicine in the evening, the drug adherence graph will show the first opening on that specific day.

10. Data Transfers

The MERM system will start the data transfer process automatically after the MERM system is started, but with a 60-second delay. During this time, data will be transferred automatically to the NIAID data management center. Please note that an active Internet connection is required for the process to work. Participants' adherence data files cannot be transferred if the Internet is not available and will remain on the desktop or laptop until the Internet becomes available again.

If there are any problems with the data transfer process, the user may perform a manual data transfer. Please note, we do not expect this function to be part of the day-to-day operations.

If the **Export Pending** function is invoked, the MERM system will immediately start uploading all the participant's adherence data files that were not uploaded before, and move the successfully uploaded files to an archive status.

If **Export Archive** is invoked, the system will immediately start uploading all of the participant's adherence data files -- new files as well as old files (archived files) that were previously uploaded. Please note, there should be no reason to perform this function unless there is a direct request from the NIAID data management center.

An **Upload Status** icon is provided on the MERM front-end to indicate data transfer upload status. The icon can be in the following colours - red, yellow, or green, with a number inside.

Red	There was an error during the last upload. The number indicates the number of files still not uploaded. Corrective action: Ensure the Internet connection is active, and restart and reconnect the MERM. If data transfer automation still does not work, run the manual data transfer "Export Pending".
Yellow	There is an ongoing upload. The number indicates the number of files remaining to be uploaded.
Green	The last upload was successful. The number indicates the number of files remaining to be uploaded (see the upload log for details). Most of the time, users will see "0" – indicating there are no more files pending upload.

11. Upload Log File

The Upload Log File lists all MERM data transfers and whether or not they were successful. The file may assist problem-solving efforts if drug adherence data transfers fail.

Source file name to upload	Target file name to upload	Timestamp file upload started	Timestamp file upload completed	Upload status	Error message
C:\.....\MERM\MERMData\MERMEXPORT _57D0F3FE-CNE1 C-00003664_14009.txt.pending	C:\...\MERM 1.0.0.5\NIAID MERM\MERM\MERMData\MERMEXPORT _57D0F3FE-CNE1 C-00003664_14009.txt	19/Apr/2017 11:50:07	19/Apr/2017 11:50:47	Success	
C:\.....\MERM\MERMData\MERMEXPORT _57D0F3FE-CNE1 C-00003664_14009.txt.pending	C:\...\MERM 1.0.0.5\NIAID MERM\MERM\MERMData\MERMEXPORT _57D0F3FE-CNE1 C-00003664_14009.txt	19/Apr/2017 08:29:27	19/Apr/2017 08:29:43	Success	

The Upload Log File can be accessed by clicking on the Help Menu and then selecting View Log.

12. User Support

The following Predict project team will provide MERM user support.

Name	Region (Time Zone)	Phone / Email
Shera Weyers	South Africa	+27 (0)83 994 6963<br>shera@task.org.za
Michael Duvenhage	South Africa	+27 (0)83 379 5499<br>michael.duvenhage@nih.gov
Gao Jingcai	China	+86 15838015837<br>gaojingcai818@163.com
Chrissie Cai	US (EST / EDST)	+1 301 761-6273<br>caiy2@niaid.nih.gov
