# Supplementary material for: Using biomarkers to predict TB treatment duration (Predict TB): a prospective, randomized, noninferiority, treatment shortening clinical trial
Source: Gates Open Res. 2017 Nov 6;1:9. [Version 1] doi: 10.12688/gatesopenres.12750.1 (PMC5841574; doi:10.12688/gatesopenres.12750.1)
Supplement: Supplementary file 2 [file gatesopenres-1-13810-s0001.tgz › 90b435b8-6627-48f0-88dd-2c47030eec10.pdf]

Manual of Operating Procedures (MOP)-Laboratory (South Africa)

for

Using Biomarkers to Predict TB Treatment Duration (Predict TB Trial)

NIAID Protocol #16-I-N133

Version Number 2.0

Table of Contents

1 Overview 3
1.1 Sample collection schedule 3
1.2 Sample processing, transport and storage 4
1.3 Storage records 4
1.3.1 Labeling 4
1.3.2 Sample storage records 5
1.3.3 Required labeling fields: 5
1.3.4 Periodic Reconciliation of Freezer Inventory with DataFax logs 5

2 Sputum 5
2.1 Sputum collection schedule and order of sputum processing 5
2.2 Sputum collection procedures 6
2.2.1 Destination 7
2.2.2 Procedures 7
2.3 Sputum processing and testing 8
2.3.1 Sputum quality assessment 8
2.3.2 Precaution 8
2.3.3 Smear and culture 9
2.3.4 GeneXpert MTB/RIF test 9
2.3.5 Sputum for Bacterial mRNA analysis 11
2.3.6 Raw sputum for LAM analysis 13
2.3.7 Raw sputum for other biomarkers 13
2.3.8 Raw sputum for detecting persisters 13

3 Biomarkers 13
3.1 Blood Biomarkers 13
3.1.1 Sample collection schedule 13
3.1.2 Consumables and Equipment 14
3.2 SAFETY BLOODS—CBC/Chemistries/Liver Function Tests 14
3.3 Blood Biomarker Processing 15
3.4 Serum Processing 16
3.5 PAXGene Processing 16
3.6 Urine Collection, Processing, and Storage 17
3.7 Saliva 18
3.7.1 Timing and responsibility 18
3.7.2 Summary of each visit 18
3.7.3 Preparation 18
3.8 Host DNA 19
3.9 Drug Monitoring 19
3.10.3 Processing of samples 20
3.10.4 Storage and Specimen Shipping 21
3.10.5 Reporting of results 21

4 Appendices 22
4.1 Appendix 1: NHLS test request form 22
4.2 Appendix 2: Sputum Packaging Instructions from UCT Barry Lab 23
4.3 Appendix 3: Sputum Transport Log Sputum Transport Log [Site Name] 26
4.4 Appendix 4: Sputum-TRIzol Source Document 27
4.6 Appendix 6: Summary of changes from version 1.0 to version 2.0 30

1 Overview

This laboratory manual highlights the operating procedures for collection, processing, and transport of samples with focus on the study sites in South Africa.

For each visit, samples will be collected, then either processed and stored at the sites or transported to UCT-Barry laboratory for tests and storage on the same day. Extra transport will be arranged for some samples that are stored temporarily at the sites for long-term storage at UCT-Barry laboratory, depending on the storage capacity of the sites.

1.1 Sample collection schedule

	Screen	D0	W1	W2	W4	W8	W12	W16	W20	W24	W36	W48	W72	RECUR
**Sputum 1 Culture, Xpert (X)**	X	X[1]			X	X		X		X				X
**Sputum 2 TRIzol**														
**Sputum 3 Biomarkers LAM (L)**		L			L	L		L		L			L	L
**Safety Blood**														
**Pregnancy test**	Serum	Urine			Urine			Urine[2]		Urine[2]				Urine
**Blood Biomarkers**														
**Urine Biomarkers**														
**Saliva Biomarkers**														
**Blood PK**							[3]	[3]	[3]	[3]				
**Blood Drug monitoring**														
**Blood Host DNA**														

[1] *Only performed if screening GeneXpert was >7 days prior*
[2] *Performed at W16 or W24 based on randomization*
[3] *Two PK visits at any time between W12 and W24*

1.2 Sample processing, transport and storage

	Processing at the sites	Storage at the sites	Transport to UCT-Barry Lab
**Sputum 1** **Culture, Xpert**	- Refrigerate[1] immediately	None	Same day (cooler box, 2-8°C)
**Sputum 2** **+ TRIzol**	- Keep at RT[2]<br>- Mix with TRIzol immediately (within 5 mins)	-80°C or the coldest	Bulk transport/dried ice
**Sputum 3** **Biomarker**	- Refrigerate[1] immediately	None	Same day (cooler box, 2-8°C)
**Safety Blood**	None	None	None
**Pregnancy test**	None	None	None
**Blood** **Biomarkers: serum**	- >30 min at RT[2]<br>- Separation of serum/storage within 2 hr	-80°C	None
**Blood** **Biomarkers: mRNA**	- >2 hr at RT[2]<br>- Freeze[3] overnight	-80°C	None
**Urine** **Biomarkers**	- Aliquot/storage within 6 hr	-80°C	None
**Saliva** **Biomarkers**	- Refrigerate[1] immediately (Salivette tube)<br>- Aliquot/storage within 6 hr	-80°C	None
**Blood** **PK**	- Refrigerate or place on ice immediately<br>- Separation of plasma/storage within 2 hr (preferably 30 min)	Below -20°C	Bulk transport (dried ice)
**Blood** **Drug monitoring**	- Refrigerate or place on ice immediately<br>- Separation of plasma/storage within 2 hr (preferably 30 min)	Below -20°C	Bulk transport (dried ice)
**Blood** **Host DNA**	- Refrigerate[1] immediately<br>- Aliquot/storage on the same day	-80°C	None

[1] Refrigerate at 2-8℃
[2] RT: 18-25℃
[3] Freeze at -20℃

1.3 Storage records

1.3.1 Labeling

All samples should be labeled properly. At least, participant IDs, visits, sample types and aliquot numbers (if there are more than two aliquots for the same sample type), as appeared in the corresponding CRFs are required on the labels.

Use of a barcode system is recommended.

1.3.2 Sample storage records

A record of all samples should be maintained in an electronic form, either a sample database or an Excel spreadsheet. Fields in the form should be the same as appeared in the labels. Use of FreezerPro is recommended.

1.3.3 Required labeling fields:

Labeling number: Participant IDs will be generated based on a naming convention. Each ID will begin with a study identifier, followed by a country code, site code, participant code e.g. PD-1-1-001

Site	Country Code	Site code	Participant ID
Khayelitsha (CIDRI)	1	1	PD-1-1-001
SATVI	1	2	PD-1-2-001
SUN	1	3	PD-1-3-001
TASK	1	4	PD-1-4-001
UCTLI	1	5	PD-1-5-001

1.3.4 Periodic Reconciliation of Freezer Inventory with DataFax logs

On a regular basis, a list of all samples that have been recorded on CRFs will be submitted to each site and an assigned person at each site will generate a list of freezer storage addresses for each of these samples and return it to the requestor. The list generated from the CRFs will include all samples with a cut-off date of 2 weeks before the request is sent to the sites. Every 6 weeks a query will be sent for confirmation of a month of freezer positions for all samples. The freezer addresses for these samples should be returned within 1 week. If the system used by each site does not automatically define which CRF samples are absent from the freezer database when the two lists (CRF list and freezer list) are compared, then this comparison will be done by the requestor and sent back to the site with a query generated for any ambiguities; this query should be addressed with explanations within one week. If the site freezer storage program automatically generates a list of which CRF samples are absent from the freezer, the sites can investigate this list at once and send back explanations with the report.

2 Sputum

2.1 Sputum collection schedule and order of sputum processing

Up to 3 sputum samples will be collected following the collection schedule below.

- Sputum 1, for culture and Xpert test

  - **Sputum 1 must be collected at every visit**.

  If there is volume remaining, then it can be further used for XpertUltra and LAM (in that order).

- Expectorated

- Overnight or spot collection

- More than 3 or 4 ml, depending on the visit

- Sputum 2, for mRNA (+ TRIzol)

  - Expectorated or induced

  - *Spot collection*

  - 1.5 ml

- Sputum 3, for biomarkers

  - Expectorated or induced

  - 3 ml

	Screen	D0	W1	W2	W4	W8	W12	W16	W20	W24	W36	W48	W72	RECUR
**Sputum 1 Culture, Xpert**	4 ml	4 ml	3 ml	3 ml	4 ml	4 ml	3 ml	4 ml	3 ml	4 ml	3 ml	3 ml	4 ml	4 ml
**Sputum 2 TRIzol**		1.5 ml		1.5 ml	1.5 ml	1.5 ml		1.5 ml		1.5 ml		1.5 ml	1.5 ml	1.5 ml
**Sputum 3 Biomarkers**		3 ml		3 ml	3 ml	3 ml		3 ml		3 ml		3 ml	3 ml	3 ml

2.2 Sputum collection procedures

Obtaining high quality sputum without saliva contamination is the goal. Explain to the participant to rinse his/her mouth with water to remove the saliva. If he/she cannot do this, then ask him/her to spit out the saliva in his/her mouth as much as possible before expectorating. Follow site-specific procedures for where the patient expectorates the sputum.

Only use the blue-capped sputum collection containers provided to the sites. Label the sputum collection container with identifying label including PID, specimen type, and date and blank for time of collection. Provide the container to the participant and instruct him in sputum collection as described below.

Instruct the participant not to expectorate saliva or postnasal discharge into the container but rather specimen resulting from a deep cough into a blue-capped sterile sputum collection container (as shown in appendix 2). The participant should continue trying to expectorate until the required volume of sputum (which should be no more than half the volume of the container) has been collected (there is not a time requirement other than before the visit is over). Show the mark on the container(s) to the participant and instruct the participant to place the sputum specimen container in the biohazard specimen bag.

The container for Sputum 1 is given to the participant at the previous visit and he/she is asked to collect the sputum during the night before the next visit and bring the collected sputum on the day of the visit. He/she must be reminded the day before the visit. Instruct the patient to follow precautions to avoid exposure of other household members. Preferably, the specimen should be collected outside of the dwelling. The participant should fill the cup to the intended volume even if this requires multiple collections into the same cup.

If the participant fails to bring Sputum 1, it must be collected during the visit. Sputum 1 must be collected at every visit.

Assisted sputum production (whether by percussion of the chest or by use of a saline nebulizer), according to the site-specific SOP, should be implemented for participants who are no longer able to expectorate sputum spontaneously, particularly at weeks 16, 24, 48, and 72, for Sputum 2 and Sputum 3.

If for any reason, during sputum collection at the site, the staff member needs to enter the room to assist the patient, the staff member should put on personal protective equipment (PPE) properly, including a N95 mask, disposable gloves, and a protective labcoat. The staff member should not remove the mask until he/she has left the space where the participant is coughing.

The staff member must put on gloves to receive specimens from participant. Place the sputum collection container(s) 1 and 3 in the designated refrigerator or cold box, until it is delivered to the laboratory (do not freeze). Please refer to appendix 2 for detailed packaging instructions.

Sputum 2 can be stored at room temperature while preparing for its immediate mixing with TRIzol. After mixing with TRIzol, the specimen should be maintained on ice until it is delivered to the laboratory where it will be aliquoted and stored in a -80C freezer.

Sputum transport

2.2.1 Destination

Sputum 1 and 3 should be transported to UCT-Barry laboratory as soon as possible after collection in a cooler box (2-8°C).

UCT-Barry laboratory
S2.09 Wernher and Beit Building North
UCT Faculty of Health Sciences
Anzio Road
Observatory, 7925

2.2.2 Procedures

- Prior to packaging the sputum containers for transport, confirm that the specimen lids are tightly sealed though the specimen bag. If not, while wearing PPE (a NIOSH-approved mask, disposable gloves and a labcoat), transfer the bag and container to a cleaned class 2 hood with operating air flow and open the zippered bag. Reposition the lid onto the container and tighten the lid, and reseal the bag.

- As a standard procedure for packaging of infectious agents for transport, sputum samples have to be triple-packed. Place at most 2 sputum containers (from the same participant) in a larger plastic container (the secondary container) in the transport box. Please be sure to fill the plastic container with cushioning material to keep the sputum containers upright position during the transport.
- For detailed sputum packing instructions please refer to Appendix 2.
- Sputum 1: Fill out the NHLS form (Appendix 1) for each sample and put the form into the bag so as the header section of the form is visible from outside.
- List all the samples to be transported in the sputum transport log (Appendix 3) and place the logged samples into a cooler box with cold packs and seal the cooler box. Deliver the box to the driver, collect his/her signature and record the time in the transport log.

The NHLS form and the transport log will be provided as electronic forms and should be printed on A4 paper (green for the NHLS form, and white for the transport log).

- **The temperature inside the box should be kept at 2-8°C during the transit. Place enough cold packs in the box so that the temperature does not rise above 8°C. Place the temperature probe of the thermometer INSIDE the transport box to ensure accurate measurement. Place the thermometer OUTSIDE the box to enable reading without opening the box.**
- Email the scanned copies of the NHLS form and the transport log to UCT-Barry laboratory (p663predict@gmail.com). Queries or concerns may be directed to:

  Dr Gail Louw, gail.louw@uct.ac.za

  Bevika Sewgoolam, bevika.sewgoolam@uct.ac.za

It is important to know what samples are being delivered and how much. With the information the lab staff can arrange necessary steps before the samples arrive to process them as quickly as possible.

- Once the box arrives at the destination, the driver and receiver should record the time and sign the transport log.

2.3 Sputum processing and testing

2.3.1 Sputum quality assessment

The sites will assess the quality of the sputum. If the sputum is salivary, the sites should also make a note in the source documents and this information should be maintained in the FreezerPro log.

2.3.2 Precaution

The unopened bottle of beads will be brought into a clean biosafety cabinet/room where TB culture and TB DNA, and TB PCR reactions have not been processed. The bottle will then be opened and aliquoted into secondary "single use" bottles. This procedure will be performed at the start of the day before the person performing this task has entered any "dirty" biosafety cabinet/rooms where TB cultures, DNA or analysis of open PCR reactions has been performed. The person will have showered and changed all clothes from the prior day. Sterile technique will be used at all times in this procedure.

2.3.3 Smear and culture

Smear and culture will be done at the NHLS laboratory at the Groot Schuur Hospital following the NHLS laboratory SOPs.

	Test						Report		
	Flagged	Visible inspection	AFB (ZN stain)	Contamination (2% BAP, 48 h)	Mtb complex (MPT64, BD Tbc ID)	Speciation (HAIN Myco. CM)	Mycobacterial Culture	Mycobacterial ID -Ag	PCR/Line probe assay
MGIT	Positive	-	Positive	Growth positive or negative	Positive	-	Culture positive. AFB observed. + TTD (days)	Mtb complex	-
					Negative	NTM species		Negative	Species name
						other NTM			NTM (MOTT)
						Mtb complex			Mtb complex*
			Negative	Growth positive	-	-	Culture contaminated. No furthur result.	-	-
							Culture contaminated. To be re-decontaminated.*	-	-
				Growth negative	-	-	-	-	-
	Negative	Mtb suspected	Positive	Same as above	Same as above	Same as above	Same as above	Same as above	Same as above
			Negative	-	-	-	Culture negative	-	-
		No growth	-	-	-	-	Culture negative	-	-

	Test				Report	
	Visible inspection		AFB (ZN stain)	Speciation (HAIN Myco. CM)	Mycobacterial Culture	PCR/Line probe asssay
LJ	Positive	(Pure Mtb)	Positive	Mtb complex	Culture positive. AFB observed. + colony count	Mtb complex
				NTM species		Species name
				other NTM		NTM (MOTT)
			Negative	-	Culture contaminated (?)	-
	Contaminated	Mtb suspected	Positive	Same as above	Same as above	Same as above
			Negative	-	Culture contaminated.	-
		No signs of Mtb	-	-	Culture contaminated.	-
	Negative		-	-	Culture negative	-

2.3.4 GeneXpert MTB/RIF test

If the volume of sputum collected is enough for both culture and GeneXpert MTB/RIF test, a portion of Sputum 1 will be taken for the test.
If the volume of Sputum 1 is less than 3 ml there will be no GeneXpert test, except for Week 16 for which the GeneXpert test is the first priority.

Materials:

- GeneXpert kits (contains cartridges, sample reagent buffer and sterile disposable transfer pipettes)
- Extra disposable transfer pipettes (the ones that come with the kits might not be enough).
- Vortex Mixer
- Conical screw-capped tube and test tube rack
- Additional transfer pipettes
- Timer
- Laboratory Worksheet/CRF form
- Labels and indelible labeling marker
- Miscellaneous (Disposable gloves, etc)

Precautions, Use and Storage:

Safety Precautions: Universal precautions should be used when handling all biological specimens. Wear appropriate PPE (Personal Protective Equipment) including protective disposable gloves, N-95 masks, laboratory coats and eye

protection when handling specimens and reagents. After handling specimens and reagents, wash hands thoroughly. When in doubt, refer to the safety procedures set forth by your institution for working with chemical and biological samples.

Specimen Use: Specimens utilized for this assay will be freshly collected per standard laboratory protocols for specimen collection. Sputa collected will be held at 2-8°C prior to processing if the delay in processing is more than 6h.

Equipment and Material Use and Storage: Store the GeneXpert cartridges and reagents at 2-28°C. Do not open a cartridge until testing will be performed. Use the cartridge within 30 minutes of opening the lid. The cartridge is stable up to 7 days after opening the package. Follow manufacturer's instructions and standard laboratory protocols for usage and storage of other routine equipment. Once completed, dispose of used equipment into proper biohazard containers.

Test Procedures

Sample Preparation (Perform in BSL3 laboratory):

1) Label each GeneXpert assay cartridge with the sample ID or affix ID label. Do not place the label on the lid of the cartridge or obstruct the 2D barcode on the cartridge.
2) The minimum initial volume for testing is 0.75mL of raw sputum. In the biosafety cabinet, carefully open the sputum container, measure the initial volume of sputum with a pipettor set to 0.75 mL, and if there is not enough sputum to perform the test do not continue further with this specimen, review the other specimens and choose one with a larger volume, saving the first one for other uses.
3) If using only part of the specimen, mix the sputum with the pipettor and transfer 0.75 mL to a 5 mL tube and add Sample Reagent 1.5 mL, (2:1 (v/v) directly to sample).
4) Re-cap the 5mL tube, and shake vigorously 10-20 times, avoiding the creation of bubbles. (Note: One back and forth motion is a single shake)
5) Incubate the sample for 15 minutes at room temperature in the hood. After 5-10 minutes of this incubation, mix the sample again either by shaking or snap vortexing for 5 seconds, avoiding the creation of bubbles. (Note: Samples should be liquefied with no visible clumps of material; if there are remaining clumps shake again as above and incubate for another 5 to 10 minutes.)

Cartridge Preparation (Perform in BSL3 laboratory):

1) Using a sterile transfer pipette, measure the liquefied sample. If there is less than 2 mL volume, do not process the sample further.
2) Open the cartridge lid, and use a graduated transfer pipette to transfer 2 mL of sample into the open port of the GeneXpert cartridge. Dispense slowly to

avoid aerosol formation. If adding sample to multiple GeneXpert cartridges, use separate transfer pipettes for each sample.

3) Close the cartridge lid, making sure it snaps firmly into place.

Running the test (Perform in BSL2 laboratory)

(Note: Start testing within 30 minutes of sample addition to the cartridge)

1) Scan the barcode on the Assay Cartridge, and scan/type in the sample ID into the software.
2) Once instructed by the G4, load cartridge into place, and start test.

Report:

Once the testing is complete, copy the assay result file and report to the sites.

Repeat Testing:

If the results indicate ERROR, INVALID TEST or NO RESULT, the test will be repeated only for Week 16.

2.3.5 Sputum for Bacterial mRNA analysis

Consumables and equipment:

- 11ml collection tube (cat#364915) with no additive (pre-marked to indicate 1.5ml mark)
- BD vacutainer luer-lok access device (cat# 364902)-Optional
- 3ml BD Luer-lok syringe (cat# 309657)
- BD leur-lok sterile syringe tip cap (cat# 305819)-Optional
- TRIzol (Invitrogen cat# 15596026)
- BD PrecisionGlide 18G X 1 ½ (1.2mm X 40mm) (Cat# 305196)
- Corning 2ml sterile RNase-free storage cryo-vials (cat.# 430659)-Suggested
- Permanent marker
- Disinfectant wipes
- Vortex mixer-optional

Sputum processing for bacterial mRNA profiling:

Sputum 2 will be collected for mRNA processing.

The goal is to try to collect approximately 1.5ml of sputum 2 in the 11ml collection tube. The participant should be instructed to cough into the provided tube such that s/he fills the tube to roughly the indicated mark.:

1. Provide the participant with a pre-marked 11ml collection tube (The mark should be made at 1.5ml from the bottom with a permanent marker). (Also, provide the participant with a sputum cup to dispense extra volume of sputum if required as described below in 3).
2. Instruct the participant to close mouth around the opening of the tube such that the lips provide a seal when collecting sputum in the tube.
3. Then the participant should try to cough sputum into the tube avoiding spilling the sputum outside the tube or along its outside walls. The participant must attempt to

collect the sputum to the indicated mark. However, if more sputum is collected, it may be reduced by transferring into the provided sputum cup. If less is dispensed, an attempt should be made to collect more by repeating the process. If no more is produced, the sputum in the tube should still be provided to the study staff for use in other applications.

4. After coughing the desired volume into the tube, the participant must cap the tube, and clean the outside of the tube with a disinfectant wipe. The participant may now hand over the sputum tube to the study staff along with any other sputum collected.
5. The study staff must prepare 3ml BD leur-lok syringes pre-loaded with 1.5ml TRIzol and capped with luer-lok syringe tip caps to avoid spillage of TRIzol. The TRIzol-containing syringes may be prepared up to 1 week before the day of sputum 2 collection (please read note below).
   NOTE: **TRIzol is light-sensitive and should be stored in a cool, dark place. Therefore, it is suggested that the TRIzol-containing syringes also be stored in the dark.**
6. Once the sputum tube is received from the participant, TRIzol should be immediately added (within 5 mins) to and mixed with the sputum.
7. Wearing appropriate protective gear (mask, gloves, goggles and labcoat) the study staff should attach a leur-lok access device to the sputum tube such that the rubber seal is punctured and needle inside the access device pierces all the way through the rubber cap of the tube.
8. On the other end of the leur-lok access device the TRIzol-containing syringe should be attached (after removal of the tip cap). The syringe should be locked in place.
   NOTE: The use of the luer-lok accesss device is optional. Instead of a leur-lok access device a TRIzol containing syringe with an 18G needle may be used to inject TRIzol.
9. Then the plunger of the syringe should be pulled out to allow an equal volume of air to be aspirated out as the volume of TRIzol to be dispensed in.
   NOTE: This is a crucial step. Not removing an equal amount of air from the tube before pushing in the TRIzol might result in back-pressure which could cause splashing of the TRIzol or break the integrity of the tube/assembly.
   A video of the procedure can be found on PredictTB Space in the MOP folder under study documents (https://www.scienceforum.sc/sites/predicttb/Shared%20Documents/MOP/Mock%20addition%20of%20trizol%20to%20glycerol_with%20luer-lok%20device_060917.mov).
10. After aspirating air in to the syringe, the TRIzol should be gently dispensed into the tube ensuring no leaks or damage to the integrity of the set-up.
11. After dispensing TRIzol (do not force the air back in to the tube) the syringe and leur-lok access device should be detached and discarded appropriately.
12. The tube, now containing sputum and TRIzol, must be immediately mixed. This may be achieved by gently inverting the tube 15-20 times or by using a vortex mixer (if available). In either case, the TRIzol should mix with sputum as best as possible. The sputum-TRIzol mix should now be placed and transported on ice.
13. The homogenized sputum-TRIzol mix can then be aliquoted into sterile **RNase-free** cryo-vials of choice. The recommended volume for aliquoting is 1.5-1.8ml per cryo-vial for

2.0ml cryo-vials (and accordingly less for smaller ones) ensuring there is head-space left at the top of the tubes.

14. Document all steps as indicated in the source document (Appendix 4).

2.3.6 Raw sputum for LAM analysis

For each visit where a Xpert test will be conducted, a sample for LAM analysis will be saved from Sputum 3. If there is enough sputum for Sputum 1 this LAM sample will be taken preferably from Sputum 1. LAM analysis will be conducted in batches later in the study with reagents supplied by Otsuka Corp. following the instructions from their kit and a ELISA reader.

1) Using a positive displacement pipet and matching tip, transfer 0.2 mL of sputum into a sterile cryotube with screw cap lid and O-ring.
2) Snap freeze in liquid nitrogen or on an ethanol + dry ice slurry & then store frozen. Transfer on dry ice if necessary and maintain at -80°C for long term storage.
3) Once frozen, ship on dry ice in batches if the sample must be moved to another facility for analysis.

2.3.7 Raw sputum for other biomarkers

This sample can be taken from either Sputum 1 or Sputum 3.

1) Once the sputum for culture and other specimens are stored, pipet the remaining sputum in 1-ml aliquots into sterile cyrotubes with screw cap lids and O-rings.
2) Snap freeze in liquid nitrogen or on an ethanol + dry ice slurry if possible. Transfer to -80°C for long term storage.
3) Once frozen, ship on dry ice in batches if it must be moved to another facility for analysis.

2.3.8 Raw sputum for detecting persisters

At the end of treatment (16 or 24 weeks), when sputum has been induced, three 1-ml aliquots of sputum will be processed for plating for persister populations.

3 Biomarkers

3.1 Blood Biomarkers

3.1.1 Sample collection schedule

	Screen	D0	W1	W2	W4	W8	W12	W16	W20	W24	W36	W48	W72	RECUR
Safety Bloods														
Pregnancy test	Serum	Urine			Urine			Urine *		Urine *				Urine
Blood Biomarkers														

**Urine Biomarkers**														
**Saliva Biomarkers**														
**Drug Monitoring**									(all arms, same as PK)					
**Host DNA**														

** Performed at W16 and W24 based on randomization*

3.1.2 Consumables and Equipment

- 2ml screw cap tubes (Sarstedt or SSI)
- Stick on barcodes/labels suitable for storage at -80˚C
- P1000 pipette and filter tips
- P200 pipette and filter tips
- Storage boxes:
  - 10X10 for storage of 2ml screw cap tubes
  - 7X7 for storage of PAXgene tubes
- Centrifuge compatible with the primary blood tubes
- Sputum/urine jars
- 4mL EDTA tubes (BD cat # 367861) – Host DNA and Safety blood
- 2mL EDTA tubes (BD cat # 367842) – PK Samples
- PAXgene tubes (BD cat # 762165)
- Red top serum tubes, 5mL/6mL (BD cat # 367814/367815) – Safety blood and biomarker serum
- Sodium Heparin tubes, 10mL (BD cat # 367874) – PBMC's (Optional)
- Sarstedt Salivettes (Biodex cat # 51.1534) - Saliva
- Custom made freezer racks to accommodate 7x7 box for PAXGene tubes (optional)

3.2 SAFETY BLOODS—CBC/Chemistries/Liver Function Tests

Safety bloods will be processed by an accredited lab of choice for each individual site. Tests will be done per standard of care for each site.

For results that are out of range, staff should confirm whether the out of range results indicate the participant is not eligible for the study. For safety bloods during the study, test results should be reported to the study doctor who should follow standard site procedure for further testing or monitoring.

The following tests will be requested as Safety Bloods:

Safety Screening Blood

	Tube	Volume
**AST**	1 x Serum tube: Red top<br>Or<br>1 x Serum tube: Gold top (preferred by NHLS)	5mL
**ALT**		
**HIV**		
**Creatinine**		
**ß-hCG (Pregnancy test)**	Serum (same tube as above) or urine	
**Full Blood Count**	EDTA: Purple top	4mL
**HbA1c**		

Pregnancy testing on serum is preferred at Screening however; urine testing can be performed if serum testing is not available. All required timepoints after Screening will be done on urine. Any urine pregnancy test kit that is approved by the local regulatory authority may be used.

3.3 Blood Biomarker Processing

Biomarker sample collection

	Tube	Volume	Number of tubes
**Serum**	Red top Serum tube - no gel	4mL	2
**Host mRNA**	PAXGene tube	2.5 mL	2
**Pharmacokinetics**	EDTA	1-2mL	4 (1 each timepoint)
**Host DNA**	EDTA	4mL	1
*** Whole blood (PBMC's optional)**	Sodium Heparin	10mL	4
**Urine**	Urine specimen container	5 - 20mL	1
**Saliva**	Salivettes	1mL	3
**Drug Monitoring**	EDTA	1-2mL	1

*This is an optional assay for sites.

Aliquot preparation

Sample type	Volume received	# aliquots for storage
Serum	8ml whole blood	5 x 500µl, 1 x 1mL
PAXgene	2 x 2.5ml whole blood	None
Urine	5 - 20ml urine	5 x 1ml<br>1 x 1.8ml
Saliva	2.5 - 3 ml in salivettes	5 x 500µl
PK Plasma	2ml per timepoint (0,1,2,6 hour)	2 x 300µl per timepoint
Drug Monitoring	2ml whole blood	2 x 300µl

Host DNA	4ml whole blood	2 x 2mL

All aliquots should be made using 2mL Screw Cap tubes (Sarstedt or SSI equivalent)

3.4 Serum Processing

1) After phlebotomy, serum blood tubes should stand upright for a minimum of 30 minutes, at room temperature (18 - 25ºC), to ensure complete clot formation and processing of the serum should preferably occur within 2 hours but no longer than 4 hours after phlebotomy.

2) Centrifuge the serum tubes at 1200 x g for 10 -15 minutes. As there is no gel plug present in the red top serum tubes, care should be taken when removing the serum so as not to draw up cells.

3) Aliquot the serum into each of the 6 labelled aliquot tubes as follows:

   Tube 1 - 2: 500µl each for storage
   Tube 3: 1mL for LUMC
   Tube 4: 500µl for CSU (John Belisle)
   Tube 5 - 6: 500µl or remainder for storage for antibody or other marker analysis.

   NOTE: *If insufficient serum to fill all tubes, prepare as many tubes as possible with the remainder in the final tube. Record the volume on the side of the last tube for capture into the database. If extra serum is available after aliquoting, please add it to the last tube up to a maximum of 2ml.*

4) Store the tubes in a 10x10 cryobox (cardboard or plastic) at -80˚C according to the storage report that is generated in the database.

3.5 PAXGene Processing

The PAXgene tube contains an additive that stabilizes the *in vivo* gene transcription profile by reducing *in vitro* RNA degradation and minimizing gene induction.

1) The PAXgene tube should be kept at room temperature prior to use.

2) After phlebotomy, the tube should remain upright at room temperature for a minimum of 2 hours and a maximum of 72 hours before freezing.

3) Upon receipt in the laboratory, ensure sufficient time (2 hours) has passed before placing the tube in the freezer.
4) Transfer the tube to -20°C overnight and into -8°C the following day. Do not freeze the tubes in a Styrofoam tray as this may cause the tubes to crack. Frozen

tubes are subject to breakage on impact so tubes should be treated as glass tubes to avoid this.

3.6 Urine Collection, Processing, and Storage

Collection:

Urine collection provides samples for pregnancy testing and aliquots for biomarker assay groups. Clean catch is not essential for assay groups, but encouraged.

- Provide participant with ID-labeled urine container.
- Explain the clean-catch method:
  Participant goes to clinic cloakroom/toilet. When in the stall, participant cleans the area of the urethral opening (where urine passes) with paper towel(s) and water. Female participants are to wipe from front to back at least twice with two separate wet towels. Subsequently, participant starts to pass urine, stops midway, and then catches the "mid stream sample" in the container supplied (aim for 5 to 20 mL). Midstream collection is not absolutely necessary, but encouraged. participant must replace lid of container and take it back to the study staff.
- Ask participant to void at a convenient point during the visit.
- Store all the labeled sample in a refrigerator or in a laboratory cooler at 2-8°C until processing for freezing (within the work day).
- Pregnancy testing should follow the test's instructions or clinical SOP.

Processing:

Urine processing must be completed within 6 hours or by the end of the day of collection.

1) Aliquot 1ml of urine into each of 6 screw cap tubes as follows:
   - Tube 1-5: 1mL for storage
   - Tube 6: 1.8mL for CSU (John Belisle)

2) The urine should not be centrifuged prior to aliquoting.

3) Store the tubes at -80 °C according to the storage report generated in the database.

Urine Storage for Biomarkers:

- Label 4 freezer storage vials (1.6 to 2 mL) with laboratory label containing Subject ID, specimen type and visit ID
- Wearing gloves and a lab coat, arrange the specimen, sterile transfer pipettes and the storage vials in a biological safety cabinet
- open the urine container in the cabinet, pipet 1.8 mL of urine into each vial and close the caps tightly

- Record the number of vials prepared onto the laboratory log
- Store the vials upright in a rack or storage box in -20 to 80°C freezer if not immediately transfer to freezer farm or core lab
- Using the laboratory log, enter the storage information onto the storage CRF
- Transfer to other lab sites must be on dry ice to prevent the samples from thawing before analysis.
- Analysis methods will not be addressed in the MOP

3.7 Saliva

3.7.1 Timing and responsibility

Saliva will be collected at the day 0, week 4, week 16, week 24, and suspected recurrence visits at the clinical site.

Materials

- Salivette tubes per patient
- Supplier: Sarstedt
- Catalogue number: 51.1534 “Cotton swab without preparation”
- Ice chest and wet ice
- Centrifuge
- 1ml pipettor
- Sterile Pipette tips
- Ice and Ice chest
- Sterile Barcoded 1.6 to 2 mL Cryo vials
- Scanner and computer for entry into database
- Barcoded Freezer Box, final location entered into database
- -80°C for storage

3.7.2 Summary of each visit

- Saliva is collected from 3 chews for each patient
- Does not have to be sequential, but should be done before patient is fed
- Coordinate timing of collection prior to administering [18F] FDG so that the samples do not become radioactive.

3.7.3 Preparation

- Label the salivette tubes with patient ID, name, date and time of collection (temporary labels- no bar code required)
- Record when the participant last ate or drank
- Remove the swab from the first salivette
- Place the swab in the mouth of the participant with tweezers
- The participant chews the swab for 45 seconds to stimulate salivation
- Collect the swab with tweezers
- Place the swab back into the salivette tube

- Store the salivettes on wet ice until taken to the lab
- Repeat collection of saliva twice more, timing according to the comfort of the patient

3.7.4 Saliva Processing

Previous literature show that saliva may contain MTB thus it is recommended that the saliva processing occur within a BSL 3 facility. Salivettes should be kept at 2 - 8°C until processing can occur and processing should be completed within 6 hours.

1) Centrifuge each of the 3 salivette tubes at 1000 x g for 2 minutes to collect the saliva in the bottom of the tube. This should occur at 2 - 8 °C wherever possible.

2) The yield of saliva should be approximately 1ml from each salivette tube (approximately 2.5 to 3mL in total).

3) Aliquot 500ul of saliva into each of 5 screw cap tubes as follows:

   Tube 1-3: 500µl for storage
   Tube 4: 500µl for CSU (John Belisle)
   Tube 5: 500µl or remainder for storage

4) Store the tubes at -80°C according to the storage report generated in the database.

3.8 Host DNA

All sites will collect whole blood for host DNA at the Day 0 visit only.

- At the day 0 visit only (baseline) draw whole blood using a 4 mL EDTA vacutainer tube.
- Invert the tube to mix in the EDTA and keep at 2 - 8 °C or on wet ice until the blood can be processed for freezing. Blood should be frozen on the same work day.
- Label two 2 mL screw cap tubes per blood sample with labels reflecting the subject ID, timepoint and sample type.
- Aliquot 1.8mL of whole blood into each of the two tubes. If there is insufficient blood to fill both tubes, divide the blood equally between the tubes.
- Store the tubes in a 10x10 cryobox (cardboard or plastic) at -80˚C according to the storage report that is generated in the database as soon as possible after processing.

3.9 Drug Monitoring

Drug Monitoring samples are only received for the Week 20 timepoint and will not coincide with the PK sample processing. Sample processing should occur within 2 hours from phlebotomy and the samples should be frozen immediately after processing with minimal delay at room temperature.

Samples should be processed using the same procedure as PK Substudy samples below (Section 3.10).

3.10 PK Substudy blood collection

3.10.1 Notes on timing of blood collection and TB drug administration

- Participants must arrive for the study visit without having taken their TB drug dose for that day or the study must be delayed to another day.
- Windows for the blood draws are +/- 10 min for the 1 and 2 hour draws and +/- 20 min for the 6 hour draw.
- It is best to process blood into plasma within 2 hours but preferably 30 minutes, so the processing will have to take place at the site during the time when the samples are being collected.
- Normal procedures and precautions for drawing blood should be followed

3.10.2 Procedure for timing of blood collection and TB drug administration

- Record the time TB medication was taken (by patient report or check the pill box opening times) for the two previous days
- Using a Lavender top [EDTA] tube, collect 2 mL of blood, record the time
- Have the subject take their INH/RIF dose and record the time
- Place the tube on wet ice, in cooler box, or refrigerate (2-8°C) immediately.
- Process or pass on the blood for processing
- Draw 3 additional blood samples (approximately 1-2 mL each) into Lavender top tubes at approximately 1, 2, and 6 hours post-dose, recording the time and holding the tubes on ice or refrigerate (2-8°C) immediately.

3.10.3 Processing of samples

The LC-MS assay for identifying and quantifying RIF and INH requires 50 to 100 µL of plasma per assay, so storing at least 200 µL (but preferably 300 µL) will allow for repeated measures of each time point if desired. Keep plasma at 4°C between collection and processing.

1) Keep the samples on ice or cold whenever possible

2) Centrifuge the plasma samples at 1200 – 1300 x g for 10 – 15 minutes at 4°C if possible.

3) Aliquot 300µl of plasma into each of 2 labelled tubes. If there is insufficient volume (<600µl) to complete both aliquots, aliquot 200µl into the first tube and the remainder into the second tube.

4) Store the tubes in a 10x10 cryobox (cardboard or plastic) at -80˚C according to the storage report that is generated in the database as soon as possible after processing.

5) Tubes will be shipped every 1 – 2 months with prior arrangement with Taeksun at Barry Lab, UCT.

3.10.4 Storage and Specimen Shipping

- The plasma samples will be transported to the UCT-Barry laboratory in a box containing dried ice.
- The UCT Barry lab will then transfer the samples to the analysis lab without thawing and maintain frozen until the samples will be analyzed by liquid chromatography mass spectrometry.

3.10.5 Reporting of results

Results of the analysis – concentrations of INH and RIF in the blood samples – will be reported in CSV files that can be imported into datafax.

4 Appendices

4.1 Appendix 1: NHLS test request form

TRIALS ONLY

GROOTE SCHUUR HOSPITAL
DEPARTMENT OF CLINICAL LABORATORIES

LAB TRIAL NO. SA833	PREDICT TB Study
Location S A 0 8 3 3	PID [ ] - [ ] - [ ] [ ] [ ] Sex [ ]
Ward: Co-ordinator -tick appropriate box<br>[ ] *CIDRI* [ ] *TASK*<br>[ ] *SATVI* [ ] *UCT LUNG INS*<br>[ ] *SUN*<br>*Note to capturer: enter ward*	Initials [ ] [ ] [ ] (first middle last) DOB [ ] [ ] [ ] [ ] [ ] [ ] (d d m m y y)
Specimen: SPUTUM<br>Date taken: [ ] [ ] [ ] [ ] [ ] [ ]<br>Time taken: [ ] [ ] H [ ] [ ]<br>Taken by: [ ]	VISIT: [ ]

Investigator:	Dr Clifton Barry	Tel: 021 650 1572	Account:
Co-ordinator:	Dr Taeksun Song	Tel: 021 650 1572 Cell: 083 980 5621	12ZSTU000855

MICROBIOLOGY
Tel: (021) 404-5137/5340

X	TB Microscopy<br>TB culture and senstivity<br>LJ Culture	AUR<br>TBCUL<br>MDUM<br>TBSM	TM833

LABORATORY USE ONLY

Auramine	**Yes**
GeneXpert on sample	No
LJ Slope	**Yes**
Hain MTBDRplus on isolate	**No (Perform Antigen Test)**
Susceptibility testing (MGIT)	**Yes**
2nd line DST for MDR isolates	No
Routine identification MOTTS	No
Storage of isolates (RT)	**Yes (To be collected daily)**

SA833 M1 vers 1.1 08-May-17

In the event of any problems with the specimen(s), please contact the trial co-ordinator

NB. Fill in non-shaded areas only. No amendments allowed - specifically, no additional tests may be added

4.2 Appendix 2: Sputum Packaging Instructions from UCT Barry Lab

Sputum packaging

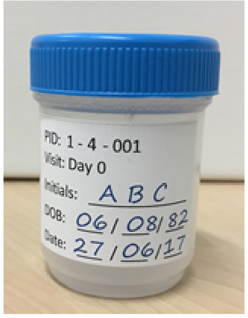

Only use the sputum containers with blue lid provided to the sites.

- Do not use other containers as this makes sample processing by the Barry Lab and the NHLS difficult.

The maximum volume: NOT more than a half of the container

- Adding glass beads and vortexing sputum volumes greater than 15 ml can lead to spills.
- Larger volumes also increases the risk of sputum leakage from the container.

Please ensure that the lid of the sputum container is properly closed to prevent spills.

The Sputum 1 label and the NHLS form

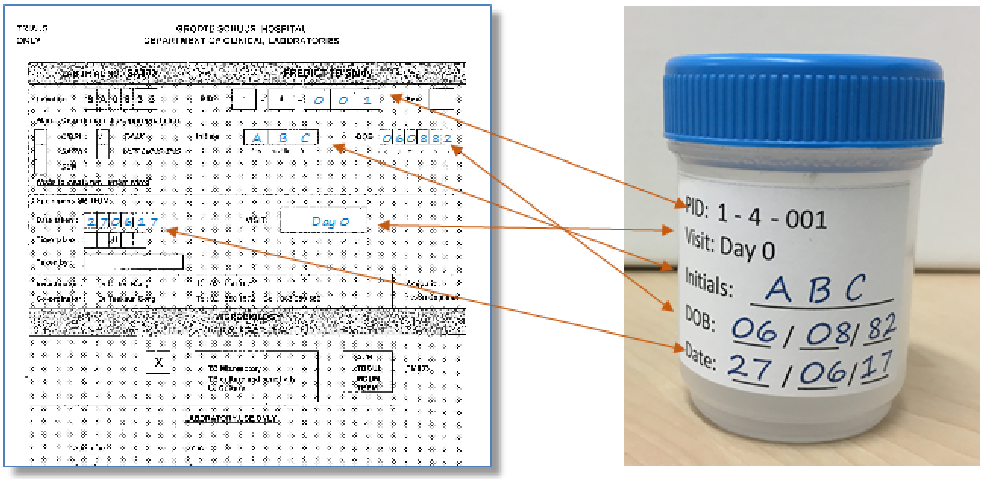

- Ensure that the correct label is on on the sputum container.
- Ensure that the information on the sputum container is the same as on the NHLS form.
- Fields such as PID, Visit number, Initials, DOB and Date should match.

Ice packs, the thermometer, and the transport log

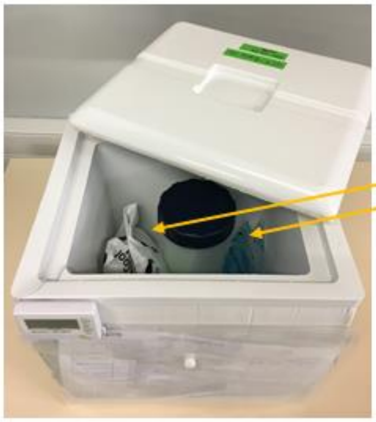

Place ice packs around the secondary container inside the transport box to keep the sputum sample cool.

Place a temperature probe of the thermometer INSIDE the transport box to ensure accurate measurement.

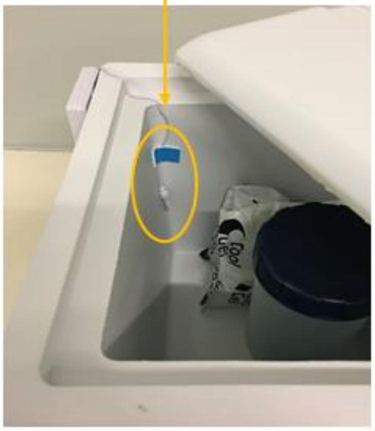

Place the thermometer OUTSIDE the box to enable reading without opening the box

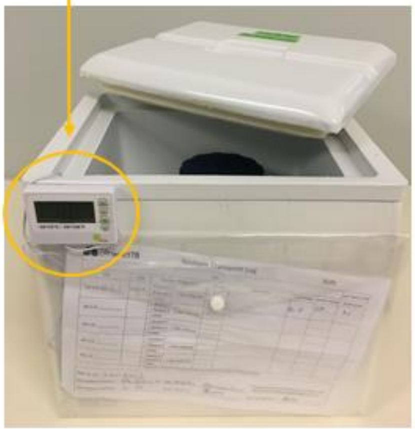

Place the sputum transport log on the OUTSIDE of the transport box.

Do not place the sputum transport log inside the transport box because opening the box is not allowed in the BSL2 laboratory.

Ensure that the transport box is securely closed during transport of the sputum sample.

Sputum transport log

- Please complete the form in the blue boxes and ***send a scanned copy to UCT-Barry lab by email (p663predict@gmail.com)*** while the driver is on the way. Driver's initial is optional.
- The bottom part will be completed when UCT-Barry Lab receives the samples.

Version 1.0, 8 June 2017

PredictTB Sputum Transport Log CIDRI

Site							UCT-Barry Lab
PID	Visit	Sputum designation	Volume	Comments	Staff initial	Driver initial	Staff initial
PD-1-1-__ __ __		Sputum 1*				@Site	
		Sputum 3 Not collected					
		other				@UCT-Barry	
PD-1-1-__ __ __		Sputum 1*				@Site	
		Sputum 3 Not collected					
		other				@UCT-Barry	
PD-1-1-__ __ __		Sputum 1*				@Site	
		Sputum 3 Not collected					
		other				@UCT-Barry	
PD-1-1-__ __ __		Sputum 1*				@Site	
		Sputum 3 Not collected					
		other				@UCT-Barry	
PD-1-1-__ __ __		Sputum 1*				@Site	
		Sputum 3 Not collected					
		other				@UCT-Barry	

To be completed at the sites

** Sputum 1 must be accompanied by the NHLS form in the vinyl bag provided.*

Date: __ / ___ / To be completed at the sites

Time delivery started: __ : __ Site staff signature __________

** Please send a scanned copy to UCT-Barry Lab by email at bevika.sewgoolam@uct.ac.za.*

Time delivery finished: __ : __ Driver signature __________ Lab staff signature __________

To be completed at UCT-Barry Lab

Sputum volume

The volume of sputum can be estimated by comparison with known volumes of liquid in the same sputum container.

When noting the sputum volume, if the volume is a fraction then round off the volume to the previous whole number. For example:

- If the estimated volume is less than 3 ml it should be indicted as 2 ml.
- If the estimated volume is equal to or more than 3 ml it should be indicated as 3 ml.
- If it is equal to or more than 4 ml it should be indicated as 4 ml.

UCT Barry lab requests

- Please inform UCT Barry lab the day before OR the morning (by 10 am) of possible patient visit
- Please ensure continuous communication with regards to expected sample delivery time etc.
- *The daily cut off times for sample delivery to the UCT Barry lab is preferably 11:00 am and no later than 2:00 pm.*

PredictTB

4.3 Appendix 3: Sputum Transport Log

Sputum Transport Log [Site Name]

Site							UCT-Barry Lab
PID	Visit	Sputum designation	Volume	Comments	Staff initial	Driver initial	Staff initial
PD-1-___- ___ ___ ___		☐ Sputum 1*				@Site	
		☐ Sputum 3 ☐ Not collected				------------------------	
		☐ other				@UCT-Barry	
PD-1-___- ___ ___ ___		☐ Sputum 1*				@Site	
		☐ Sputum 3 ☐ Not collected				------------------------	
		☐ other				@UCT-Barry	
PD-1-___- ___ ___ ___		☐ Sputum 1*				@Site	
		☐ Sputum 3 ☐ Not collected				------------------------	
		☐ other				@UCT-Barry	
PD-1-___- ___ ___ ___		☐ Sputum 1*				@Site	
		☐ Sputum 3 ☐ Not collected				------------------------	
		☐ other				@UCT-Barry	
PD-1-___- ___ ___ ___		☐ Sputum 1*				@Site	
		☐ Sputum 3 ☐ Not collected				------------------------	
		☐ other				@UCT-Barry	

** Sputum 1 must be accompanied by the NHLS form in the vinyl bag provided.*

Date: __ __ / __ __ __ / __ __ __ __

Time delivery started: __ __ : __ __

Site staff signature ________________________

** Please send a scanned copy to UCT-Barry Lab by email at bevika.sewgoolam@uct.ac.za.*

Time delivery finished: __ __ : __ __

Driver signature ________________________

Lab staff signature ________________________

4.4 Appendix 4: Sputum-TRIzol Source Document

Sputum processing for mRNA transcriptomics

Participant ID ____________________________

Visit D0 W2 W4 W8 W16 W24 W48 W72 Recurrence

1 If the mRNA sample was not collected, please specify a reason ☐

2 Date sputum was produced ☐

NOTE: All subsequent steps must be able to occur on the date of production, or the sputum should be dedicated to another purpose

3 Time sputum was produced ☐

NOTE: Sputum must be maintained at room temperature (18-25ºC) before adding TRIzol

4 Volume of sputum collected (ml) ☐

NOTE: Volume of TRIzol added should be roughly equal to the volume of sputum collected

5 Time TRIzol was added ☐

6 Was the sputum-TRIzol mix agitated immediately (within 5 minutes)? Y/N ☐ If no, go to 7. If yes, go to 8

7 How was the sample mixed after adding TRIzol
- Mixed using a vortex mixer
- Mixed in the hood using a pipet
- Mixed by inverting tube at least 15-20 times

NOTE: Vortexing is recommended for sputum collected in tubes while pipetting is recommended for sputum collected in a cup

8 Time sputum-TRIzol mix was mixed by pipetting, vortexing, or inverting ☐

9 Time sputum-TRIzol mix was placed on ice/cooled and maintained cold (2-8ºC) ☐

10 Time sputum-TRIzol mix was aliquoted into storage tubes and frozen ☐

11 Number of aliquoted tubes ☐

PredictTB

4.5 Appendix 5: Predict TB Trial Protocol 16-I-N133 PET/CT Scan booking form

Enrolment date: ________________________

Name	Study ID	PET visit # (W0/ W4/ W16/W24)	Folder #	Address	DOB	ID number

Is this person's weight greater than 120 Kg? **Y** **N** **If yes, Actual weight:** __________________

Have you included a copy of the participants ID? **Y** **N** **N/A**
(Baseline scan only)

Email to: bronwynt@sun.ac.za
nomvuyos@sun.ac.za

Comments: ______________________________________
____
____
____

Date emailed ______________________________________
Emailed by ______________________________________
(print name)
Signature ______________________________________

DOCUMENT HISTORY

Version No.	Date Approved/ Reviewed	Location of Change History	Author	Approving Official	Next Review Date
1.0	20 June 2017	*New form in use*	B Smith	L Muller	June 2018
2.0	05 October 2017	*Table: Removed column for GeneXpert and weight. Moved the PET visit number to take place of visit number. Removed 2 rows. Changes approved by Valencia at PET Centre.*<br>*Removed e-mail for Nashreen and replaced with e-mail for Nomvuyo.*<br>*Added 2 questions about weight and attaching ID for baseline bookings.*<br>*Added a section for comments*	B Smith	L Muller	As needed

4.6 Appendix 6: Summary of changes from version 1.0 to version 2.0

S. No.	Pg no.	Section	Section description	Description
1	throughout	header		version number updated to version 2.0, date updated to 08-08-17
2	throughout	various		formatting changes
3	1	MOP face page		version no. updated to version 2.0 and removed changes table because inserted separately as appendix item
4	throughout	various	various	trizol changed to TRIzol
5	3, 7	1.1 Table, Table for sputum volumes	sample collection schedule	Table updated to indicate that:<br>i. sputum for mRNA will also be collected on wk 48.<br>ii. saliva will be collected on both week 16 and 24 visits<br>Sputum volume table updated to indicate sputum 2 is to be collected on wk48 and 72 as well.
6	4	1.2 Table	Sample processing, transport and storage	Updated information that TRIzol should be added to sputum 2 within 5 mins if possible
7	5	1.3.4	Periodic Reconciliation of Freezer Inventory with DataFax logs	A new section has been added defining the strategy for ensuring missing freezer samples are caught before too late
8	6	2.2	Sputum collection procedures	Clarified that only blue-capped sputum cups are to be used which should be no more than half full.
9	7	2.2	Sputum collection procedures	Clarified that sputum must be collected even when participants are no longer able to expectorate
10	6 and 7	2.2	Sputum collection procedures	included reference to appendix 2 for detailed packaging and transport instructions
11	8	2.2	Sputum collection procedures	Clarified that at most 2 sputum containers may be placed in one bag
12	8	2.2.2	Sputum collection procedures-procedures	Clarified the placement of the probe and the thermometer with respect to the transport box
13	8	2.2.2	Sputum collection procedures-procedures	Included email address to which scanned transport logs are to be sent: p663predict@gmail.com

14	8	2.3.1	Sputum quality assessment	Included new section on sputum quality assessment
15	11	2.3.5	Sputum processing for mRNA	Included a suggestion for RNase-free cryo-vials which can be used for aliquoting
16	12	2.3.5	Sputum processing for mRNA	Updated information that TRIzol should be added to sputum 2 within 5 mins
17	12	2.3.5	Sputum processing for mRNA	Included link to sputum-trizol movie on PredictTB Space
18	13	2.3.5	Sputum processing for mRNA	included instructions for mRNA aliquot preparation
19	13	2.3.5	Sputum processing for mRNA	included instructions for documentation of mRNA procedure steps in source document
20	15	3.3-aliquot preparation Table	Blood biomarker processing	Updated PAXgene tube volume and added host DNA aliquot volume and vial number information
21	17	3.6	Urine Collection, Processing and Storage	Corrected volume for tube 6 from 1.5ml to 1.8ml in 2 places
22	17	3.7 and all subsequent sections	Saliva	Corrected sectioning numbering from 3.5. to 3.7 and all subsequent numbers.
23	18	3.7.1	Saliva- Timing and responsibility	Updated the sample collection times to clarify collection at both weeks 16 and 24
24	19	3.8	Host DNA	aliquot volume updated to 1.8ml
25	20	3.9	Drug monitoring	Updated section number for PK substudy
26	22-26	4.2	Appendix 2	Inserted sputum packaging and transport instructions from UCT Barry Lab
27	27	4.3	Appendix 3	Sputum transport log
28	28	4.4	Appendix 4	Sputum-TRIzol source document (please note additional time points W48 and W72 indicated on this version).
29	29-30	4.5	Appendix 5	PET/CT scan booking form
30	31-32	4.6	Appendix 6	Summary of changes since the previous version
