# Supplementary material for: Using biomarkers to predict TB treatment duration (Predict TB): a prospective, randomized, noninferiority, treatment shortening clinical trial
Source: Gates Open Res. 2017 Nov 6;1:9. [Version 1] doi: 10.12688/gatesopenres.12750.1 (PMC5841574; doi:10.12688/gatesopenres.12750.1)
Supplement: Supplementary file 3 [file gatesopenres-1-13810-s0002.tgz › 45fc70d0-b05f-43f5-9e8f-8366d00896fd.pdf]

Core Laboratory MOP
Protocol Name: *Using Biomarkers to Predict TB Treatment Duration*

Site: Henan CDC, Zhengzhou, China

Title: Routine procedures for mycobacterium culture and testing and biomarker sample storage

**Version #** 2.0	**Effective Date:** 20 SEP 17

Prepared by:
Name: Laura E. Via
Title: Assoc. Staff Scientist, NIH

1. Labeling, Barcoding, and Storing of Specimens

a. Labels- making and using

- Input all the information into spreadsheet provided
- Import spreadsheet into FreezerPro
- A barcode will be generated automatically in FreezerPro for each sample that has been imported
- Highlight the samples of which the labels have to be printed

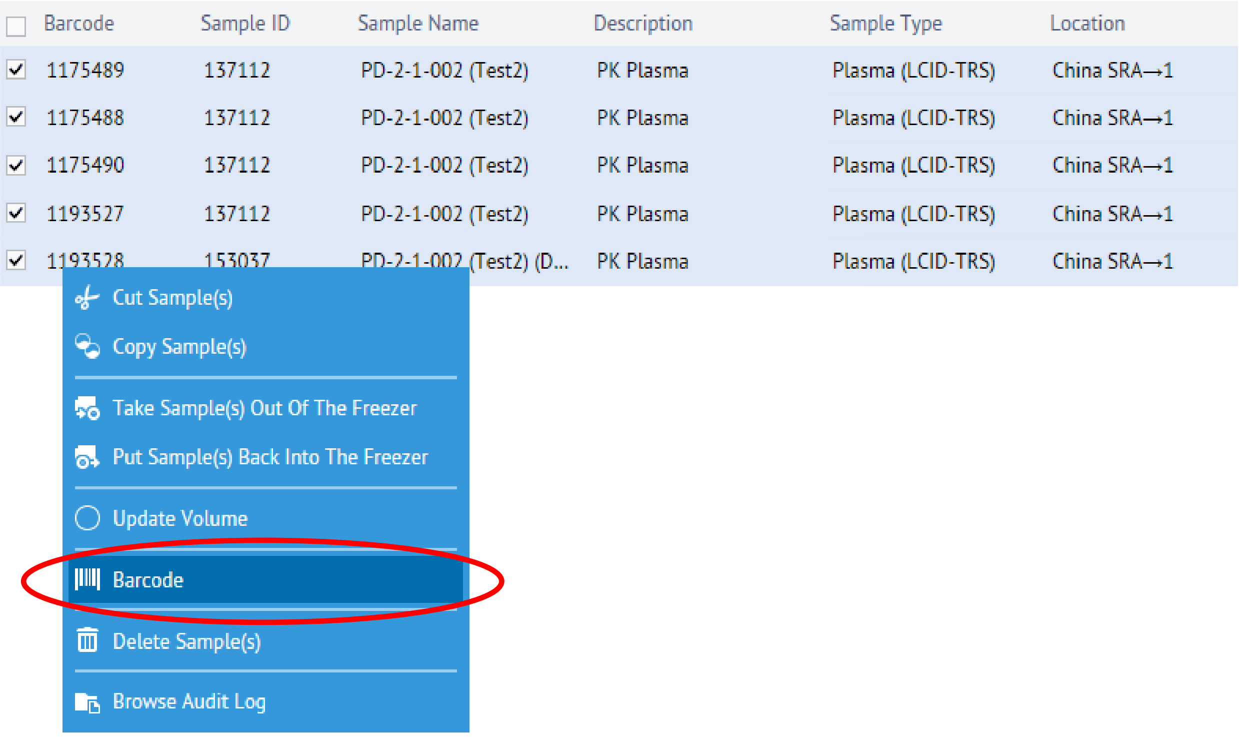

- Press the icon for Print Barcode and select the appropriate label template and click OK to print

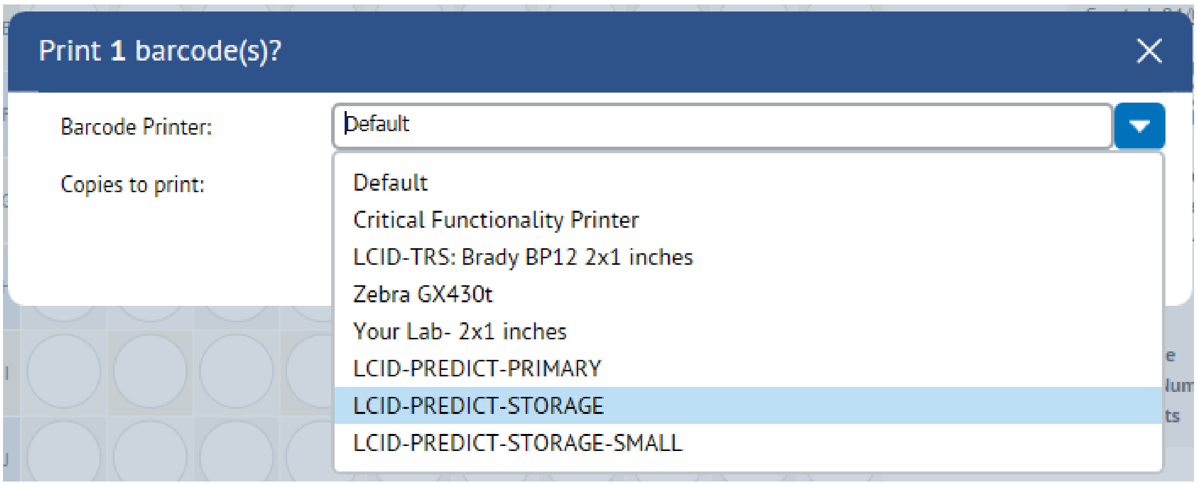

b. Use of bar code reader

- Go to Staging and Receiving area, the virtual freezer that was created for generating barcodes in Step a.
- Click on the barcode icon and click on Search:Vials

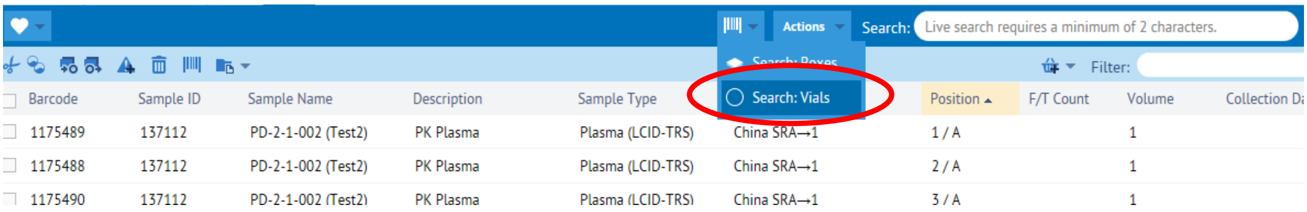

- The Search Vials window pops up. Scan barcode on each tube and the barcode will show up in the window. You can scan multiple samples, separate barcodes by return, comma, or semicolon.

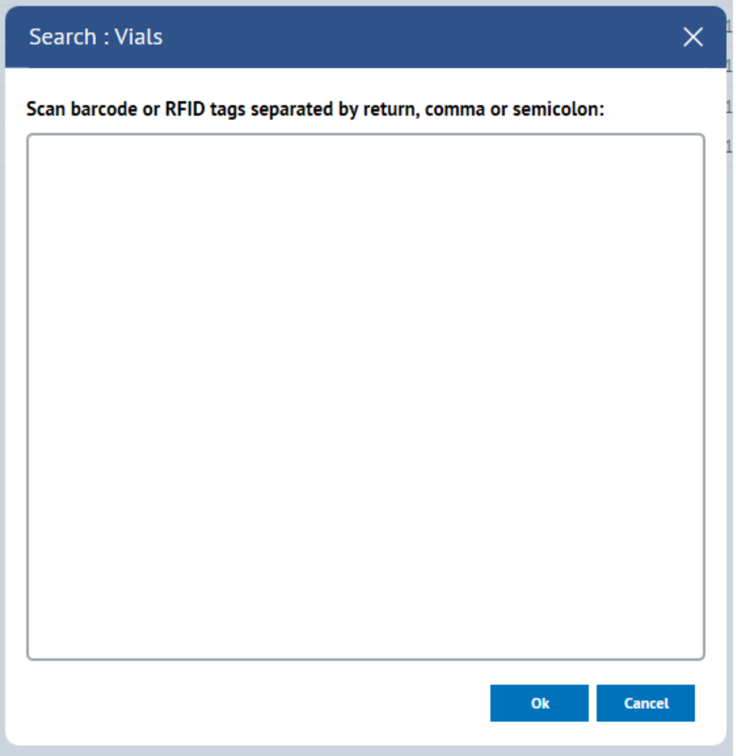

- For storage of a sample in the freezer, scan the barcode on the label with a barcode reader
  in the search position within FreezerPro
- Highlight the sample and drag it to the assigned location in the freezer within FreezerPro
- Note the location and physically put the sample in the assigned location in the freezer

c. Use of Freezer Pro to track study samples (core lab)

- Scan barcodes on the tubes into the appropriate study database or FreezerPro [Refer to study-specific sample storage SOP].
- A position in a sample-type specific box within a -80ºC freezer should be allocated to each tube. [Refer to sample type description for instructions for specific sample freezing.]
- Place the barcoded tubes in the corresponding allocated positions in one of these clearly labelled 9 x 9 storage boxes or 7x7 PAXgene samples storage boxes (Study Name, sample type, freezer name, rack letter, box number)
- Where possible, two aliquots will be allocated to one freezer and the remaining two to another freezer.
- Position 1 is always in the top left hand corner, and positions 81or 49 in the bottom most right hand corner of the storage boxes.
- Once 81 or 49 positions have been allocated in a certain box, move onto the next consecutively labelled box in that rack, starting again at position 1.

2. Sputum Requirements and Handling Procedure Overview:

- Sputa will arrive from the sites in a cooler box at 2 to 8 C and each subject's sample set needs to be logged into the lab on sputum receipt log and the temperature of the box recorded on the log.
- The sputa must be refrigerated or cooled 2 to 8 C until they are processed. All samples should be processed as soon as practical, with a target of 48h from receipt at the clinical site and within 80h of production to preserve quality.
- Each subject should have 2- 3 sputum samples from each visit. Refer to the visit flow charts or Table 1 below for the specifics of each visit.
- The sputum cups will be numbered and a packing list for specimens arriving from the site will include a copy of the sample source document made at the site with information like time/date of production and method of production for each sample.
- Confirm receipt of the sputum sample source document, if it has not been sent, contact the site immediately to get a scanned copy of the document
- Culture on LJ is the primary end point of the study so this is always the most important test if there is limited sputum available.
- If any of the sputum samples (or all) appear to be only saliva, the culture, Xpert MTB/RIF, Xpert Ultra, and LAM tests will still prepared. In FreezerPro, add a note that sample appears to contain saliva only.
- INDUCED SPUTUM is not to be used for culture or Xpert tests, it is only to be used for biomarker raw sputum storage.

Table 1 Core lab tests – Tests in order of priority and recommended volume (mL)						
Time Point	Culture	Xpert MTB	LAM	Raw Sputum	Xpert Ultra*	Comments
Screen	2-3	sites[a]	0.2		0.75[b]	
Day 0	2-3	0.75[c]	0.2	1.0[d]	0.75[c]	
Wk 1	2-3					
Wk 2	2-3					
Wk 4	2-3	0.75	0.2	1.0[d]	0.75[b]	
Wk 8	2-3	0.75	0.2	1.0[d]	0.75[b]	
Wk 12	2-3					
Wk 16	2-3	sites[a]	0.2	1.0[d]	0.75[b]	
Wk 20	2-3					
Wk 24	2-3	0.75	0.2	1.0[d]	0.75[b]	
Wk 36	2-3					
Wk 48	2-3					
Wk 72	2-3					
Relapse	2-3	0.75	0.2	1.0[d]		
*Ultra storage is done from raw sputum if available, otherwise use stored sputum sediment later						
[a] Clinical sites will conduct this test with 0.75 mL raw sputum						
[b] If 3 mL of raw sputum volume is available for storage, otherwise use sediment						
[c] If more than 7 days since screening test was done, Xpert is repeated						
[d] make as many vials as necessary to store all sputum. All induced sputum will be stored as raw sputum if it is sent to the core lab. Write in the FreezerPro record, that a specific sample is induced sputum if that sample is reported by the study site to be induced.						

3. Sputum Processing, Storage and Smear Preparation

a. Background

The majority of clinical specimens sent to the mycobacteriology laboratory for cultural confirmation of suspected mycobacterial infection are contaminated by rapidly growing normal repertory flora. To maximize the mycobacterial yield, contaminated specimens require treatment with a digestion and decontamination procedure. NALC-NaOH-Citrate-Solution–or NALC---NaOH– is a gentle but effective decontaminating agent. NaOH (Sodium Hydroxide) can be used both as a digesting and decontaminating agent. As mucolytic agent, it is most effective at a final concentration of 4%. However, this concentration is not only toxic to contaminants, but also to some mycobacteria. NALC (N-Acetyl-L-cysteine) is a mucolytic agent. NALC loses its mucolytic activity on standing. Therefore, the reagents should be gently mixed before use and used within 4h. Sodium citrate (Tri-sodium-citrate-dihydrate) is included in the NALC-NaOH-Citrate solution to bind heavy metal ions that could inactivate NALC. A phosphate buffer with a pH 6.8 decreases the pH and caustic nature of NALC-NaOH-Citrate solution and lowers the specific gravity of the specimen to assist in mycobacteria recovery by centrifugation. The resuspended pellets are used for semiquantitative sputum smear and semiquantitative culture onto solid media plus liquid culture.

b. Precautions

NaOH is a caustic chemical that can cause burns on exposed skin. Wear proper protective

equipment (laboratory coat, mask, eye protection and gloves) when handling the product. Open inside a chemical fume hood when preparing this solution.

Specimens should be processed in a BSL3 or BSL2(+) environment inside a biosafety cabinet (BSC) Class II. Use appropriate personal protective equipment (N95 mask, laboratory gown and two pairs of disposable gloves) according to procedures in the biosafety SOP.

c. Materials

N-Acetyl-L-cysteine, 0.2 g measured in sterile 50 ml Conical centrifuge tubes
8% Sodium hydroxide solution (NaOH), autoclaved, 20 ml
2.9 % Tri-sodium-citrate-dihydrate solution, autoclaved, 20 ml
Phosphate buffer solution (Potassium-Di-hydrogen-phosphate) pH 6.8
Microman pipetter and sterile capillary tips with piston
Pipetter, aerosol-barrier (filter) pipet tips, timer, racks
Sterile centrifuge tubes (50 ml)
Sterile transfer pipettes (5 ml),
Camping gas or Bunsen burner, microscopy slides, pencil, disinfectant, autoclave bag
Sterile 3-4mm glass beads
Sterile gauze squares (small packages of 2 to 5)

d. Equipment

Biological Safety cabinet (class II),
Vortex,
Shaker,
Centrifuge with buckets for 50 ml centrifuge tubes and aerosol protection caps
Refrigerator

e. **Sputum Sample Handling Procedures:**

1. Dress in protective clothing, N95 mask, and two pairs of gloves.
2. Clean the mycobacteria sputum hood where the work will be done and be sure it has been running for at least 15 minutes before using it. This must be done for each ½ days work or more often.
3. Review the received sputum sample log, the sputum source document and the bags of sputum samples to be processed and sort according to ascending sequence of subject and visit numbers.
4. Fill a lab notebook entry for each patient's specimens to be processed
5. SET ANY INDUCED SPUTUM SAMPLES ASIDE FOR USE IN RAW SPUTUM STORAGE ONLY – after recording them on the lab notebook, return them to the refrigerator for later handling. Refer to section 8 for storage of induced sputum. NOTE: if only induced sputum is submitted, proceed to culture it, but all logs and notebooks must indicated that induced sputum was used so that the data team will know this.
6. Load the storage vials, 50 mL tubes and other materials into the hood in the order that they will be used
7. Working with one subject's spontaneously produced sputa at a time, estimate the volume of each sputa received comparing this with the source document from the site, and record these observations for each sputum in the lab note book

8. Based on the visit requirements, determine if there is enough sputum volume for all the tests/samples. If there is, determine which spontaneously produced sample will be cultured using the maximum recommended volume of sputum for culture.
9. If sputum is less than that recommended, determine which spontaneously produced sample will be cultured using the minimum volume for each sample type.
10. If the sputum selected for culture is > 3 ml and viscous or not homogeneous, either 1) pippete the specimen up and down vigorously with the microman pipptor and piston tips, or 2) transfer that specimen to a 50 mL centrifuge tube and add a vial of sterile 8-12 glass beads to the tube
11. Vortex specimen vigorously in 10-second intervals, 3 or more times to homogenize the specimen enough to mix the specimen well for aliquoting the material
12. Remove the 2 mL (minimum) or 3 mL (maximum) for culture to a new labeled 50 mL tube and cap tightly. Set it aside in a 50 mL tube rack for culture samples. Continue to aliquot this chosen sputum until it is all transferred to a test or storage vial as described below
    a. If sufficient sputum volume remains and <u>if the test is required</u>, remove 0.75 mL for Xpert MTB/Rif using a positive displacement pipetter into a 50 mL tube labeled with Subject ID, visit, and Xpert MTB/Rif and set aside in a 50 mL tube rack for Xpert samples (see Section 3 for processing)
    b. If sufficient sputum volume remains and if the test is required, remove 0.2 mL sample for LAM measurement with a positive displacement pipetter into the labeled LAM cryovial (see section 6 for more information)
    c. If sufficient sputum volume remains, aliquot ~ 0.75 to 1 mL of sputum into labeled sterile cryovials until the chosen sputum is empty
    d. Raw sputum can be stored at 4 C for up to 3 days to use for repeating assays if contamination or errors occur. If the sputum is not needed for these assays, transfer it to the -80 freezer by the 4th day.
13. Continue with the next spontaneously produced sputum sample, pipetting the sputum into test or storage vials as listed in Table 1 (and a, b, c, and d above) until all the sputum is used.
14. Empty the hood of all storage vials and trash, change to clean outside gloves, wipe down the hood and pipetters with disinfectant to kill any contaminants or escaped MTB.
15. With clean gloves, repeat these steps (#1-14) with next subject's specimens until all the specimens for culture are aliquoted and ready for decontamination

f. Reagent Preparation for Sputum Processing:

1. Clean the media hood (if available) otherwise completely empty and clean the mycobacteria hood fully so no possible contaminates remain
2. Prepare NALC-NaOH-solution adding 20 mL of the NaOH and 20 mL of TriSodium Citrate solutions to the 0.2 g of NaLC in a 50 mL conical tube (use this reagent within 4 hours).
3. Aliquot the PBS if necessary since any left of PBS that has been in the culture hood must be discarded after this days' work
4. Label any processing tubes, smear slides, and sediment storage vials with preprinted subject ID, visit number, sample type labels

g. Sputum decontamination and concentration

For specimens requiring digestion and concentration (i.e. being cultured), perform the following

1. Load the reagents and other materials into the hood in the order that they will be used for no more than the number of specimens that will fit in the centrifuge at one time
2. Add 4% NALC-NaOH-Citrate solution to the first centrifuge tube containing sputum for culture in a volume that is equal to the specimen volume (2 to 3 ml).
3. Vortex the alkaline suspension gently for 10-15 seconds and hold at room temperature for 15 minutes. Start timer when NaOH is added to the first specimen. Continue with the next specimen.
4. Vortex lightly or invert every 5-to-10 minutes during the 15 minute standing time.
5. When 15 minutes have passed, add phosphate buffered saline to the 45 mL mark on the centrifuge tube. Do not touch the centrifuge tube with the buffer container when dispensing buffer. It will contaminate the buffer and the next specimen.
6. Mix the sputum-buffer suspension by inverting the tubes 5-6 times or by vortexing the tubes for 10 seconds.
7. Place these 50 mL tubes in centrifuge in a balanced arrangement and centrifuge at least 3000 x g for 15 minutes using aerosol-free sealed centrifuge buckets. If possible, use a refrigerated centrifuge to maintain centrifugation temperature at 4-10°C.
8. Remove tubes from centrifuge and smoothly decant the supernatant into splash-proof container watching the pellet closely (container containing 20% tuberculocidal disinfectant) in the biosafety cabinet, leaving <u>only the sample pellet in the tube</u>. If the pellet is loose, either recentrifuge the sample for 5 to 10 minutes more, or use a sterile transfer pipette to remove the remaining NALC solution. Fresh Sterile gauze pads may also be used to absorb the final drops of NALC solution so that all the NaOH is removed. If necessary, swab lip of tube with disinfectant-soaked gauze.
9. Add phosphate buffer to the centrifuged pellet to adjust volume to 1.5 mL. Recap and vortex briefly to re-suspend the pellet.
10. Spread 1 drop of the resuspended sediment (~50µl) onto a labeled glass microscope slide. Place slides on slide rack to dry for about 20-30 min. Flame or heat on slide fixing heat block to kill the bacteria before removing the slides from the hood. Process as described in Section 4
11. Additional pellet will be utilized in the following ways: See Section 5. Culture Inoculations for instructions
    a) 2 X 0.15 mL inoculated into 2 LJ medium (to standardized volume with RSA sites)
    b) 0.5 mL inoculated into MGIT tube
    c) 0.5 mL aliquot and store for later Xpert Ultra test (if required at visit)
    d) Any remaining pellet/sediment after the above tests will be transferred to a 2mL cryovial and refrigerated for 5 days in case there is a need for culture re-digesting/re-inoculation due to contamination. After 5 days, vial will be frozen at -80°C.

4. GeneXpert Processing

The GeneXpert system is used to detect the presence of MTB DNA and rifampicin (RIF) drug resistance in expectorated sputum specimens. On weeks were GeneXpert MTB/RIF and/or Xpert Ultra is specified, each Xpert test will be performed with 0.75 mL of sputum. Ultra tests will be processed based on NIH requirements later when the cartridges are available.

Notes

- The screening Xpert is done at the clinical site, do not repeat unless the day 0 visit is more than 7 days from the screening visit of the subject
- After the Xpert MTB/RIF test is completed, the Xpert Ultra test is carried out under the NIH requirement if sputum is limited.
- Either test can be done from raw sputum or from 0.5 mL of sediment if raw sputum is limited and using samples that have been stored refrigerated or frozen is valid
- Xpert Ultra tests will be run in batches when time permits from frozen samples
- Add sample reagent 2:1 (v/v) to raw sputum and 3:1 to sediment so that the final volume of either type of sample is 2 mL or slightly more
- IMPORTANT -record which sample type is used in each assay in the lab notebook and on the CRF

Equipment:

- GeneXpert System
- Biosafety cabinet

Materials:

- GeneXpert kits (contains Cartridges, Sample reagent buffer and sterile disposable transfer pipettes)
- Microman pipetter and sterile capillary tips with pistons
- Vortex Mixer
- Conical screw-capped tube and test tube rack
- Additional transfer pipettes
- Timer
- Laboratory Worksheet/CRF form
- Labels and indelible labeling marker
- Miscellaneous (Disposable gloves, etc)

Precautions, Use and Storage:

Safety Precautions: Universal precautions should be used when handling all biological specimens. Wear appropriate PPE (Personal Protective Equipment) including protective disposable gloves, N-95 masks, laboratory coats and eye protection when handling specimens and reagents. After handling specimens and reagents, wash hands thoroughly. When in doubt, refer to the safety procedures set forth by your institution for working with chemical and biological samples.

Specimen Use: Specimens utilized for this assay will be collected per standard laboratory protocols for specimen collection. Sputa collected will be held at 2-8°C prior to processing.

Equipment and Material Use and Storage: Store the GeneXpert cartridges and reagents at 2-28°C. Do not open a cartridge until testing will be performed. Use the cartridge within 30 minutes of opening the lid. The cartridge is stable up to 7 days after opening the package. Follow manufacturing instructions and standard laboratory protocols for usage and storage of other routine equipment. Once completed, dispose of used equipment into proper biohazard containers.

Test Procedures:

Sample Preparation (Perform in Biosafety Cabinet):

1) Determine which Xpert tests are required for the visit
2) Label each GeneXpert assay cartridge with the sample ID or affix ID label. Do not place the label on the lid of the cartridge or obstruct the 2D barcode on the cartridge.
3) For Xpert MTB/RIF, the minimum initial volume for testing is 0.75 mL of raw sputum (prepared in Section 2e). For each sample of raw sputum add 1.5 mL Sample Reagent 2:1 (v/v) directly to the 50 ml tube and precede to step 6 below.
4) If there was not enough sputum the MTB/RIF aliquot in Section 2e, the test can be performed from 0.5 mL of sediment from storage. Record the low volume in the lab notebook and that sediment was used.
5) If there is sputum or sediment available for the Xpert Ultra test, transfer 0.75 mL of sputum or 0.5 mL of sediment to a 5 to 50 mL sterile tube and add 1.5 mL Sample Reagent to the tube and continue to step 6
6) Re-cap, and rotate vigorously 10-20 times, avoiding the creation of bubbles. (Note: One back and forth motion is a single rotation)
7) Incubate the sample for 15 minutes at room temperature. After 5-10 minutes of this incubation, mix the sample again either by shaking or snap vortexing for 5 seconds, avoiding the creation of bubbles. (Note: Samples should be liquefied with no visible clumps of material- if clumps remain, shake again and wait an additional 2-3 minutes)

Cartridge Preparation and Test Initiation:

(Note: Start testing within 30 minutes of sample addition to the cartridge)

1) Using a sterile transfer pipette, aspirate the liquefied sample. If there is insufficient volume (2mL), do not process the sample further.
2) Open the cartridge lid, and use a marked transfer pipette to transfer 2mL of sample into the open port of the GeneXpert cartridge. Dispense slowly to avoid aerosol formation. If adding sample to multiple GeneXpert cartridges, use separate transfer pipettes.
3) Close the cartridge lid, making sure it snaps firmly into place. If additional liquefied sample remains, it may be kept for up to 12 hours at 2-8°C if repeat testing is required. (See section below entitled “Repeat testing”).
4) Scan the barcode on the Assay Cartridge, and scan/type the sample ID.
5) Once instructed, load cartridge into place, and start test.

Results:

1) Results Interpretation and Recording: Conventional MTB/RIF Xpert:
   Once testing is complete, print out the test results and file them with the patient specific records and record results on the corresponding CRF, indicating whether MTB positive or negative, the CT values for MTB detection and RIF resistance information.

Print out the Xpert Ultra test results (PDF) and file these with the patient records. a method for upload in the database will be provided later.

2) Repeat Testing:
The decision to repeat testing with either test will be made on-site. Repeat testing will occur one time, and will be based on the following test outputs:

MTB/RIF Xpert: Results indicate ERROR, INVALID TEST or NO RESULT, only screening and 16 weeks of testing required repeated testing. Repeats will occur from sample reagent (SR) treated sample and must occur within 12 hours of SR addition. The retest information will need to be recorded on a laboratory worksheet for quality management purposes.

MTB Ultra: Results indicate ERROR, INVALID TEST or NO RESULT
Repeats will occur from sample reagent (SR) treated sample and must occur within 12 hours of SR addition. This information will need to be recorded on a laboratory worksheet for quality management purposes. If both Xpert tests fail on a given sample and sufficient volume remains for only one repeat test, priority will be given to the Conventional MTB/RIF Xpert test.

Quality Control:
Sample Processing Control (SPC)—Ensures the sample was correctly processed. The SPC contains non-infectious spores in the form of a dry spore cake that is included in each cartridge to verify adequate processing of MTB. The SPC verifies that lysis of MTB has occurred if the organisms are present and verifies that specimen processing is adequate. Additionally, this control detects specimen-associated inhibition of the real-time PCR assay. The SPC should be positive in a negative sample and can be negative or positive in a positive sample. The SPC passes if it meets the validated acceptance criteria. The test result will be “Invalid” if the SPC is not detected in a negative test.

Probe Check Control (PCC)—Before the start of the PCR reaction, the GeneXpert Dx System measures the fluorescence signal from the probes to monitor bead rehydration, reaction-tube filling, probe integrity and dye stability. Probe Check passes if it meets the assigned acceptance criteria.

Refer to the Cepheid System Operator Manual for additional information on Diagnostic Testing and Troubleshooting

5. Sputum Smear Microscopy for the Core Lab

a. Background

The smears are made to detect mycobacteria though auramine fluorescent microscopy to give a semi-quantitative estimation of their number (conducted on sputum or sputum sediment

only), which serves as a rough guide to the bacterial burden of disease and the infectiousness of the diseased patient. Mycobacteria and related organisms retain carbol-fuchsin and other dyes like auramine despite washing with acid/alcohol. This feature is exploited to differentiate mycobacteria from the background of cellular material and other organisms in clinical specimens.

b. Precautions:

Chemicals used in the AFB staining process can be irritating to skin. Utilize laboratory gowns and disposable gloves when handling. If preparing stains in--house, use a face-shield and/or goggles for eye protection. Perform work in the appropriate biosafety environment.

Materials and Equipment:

- Sink and running water
- AFB Fluorescent Staining reagents
- Microscope slide box
- Forceps
- Quality control slides (to be run per batch)
- Laboratory worksheet
- Microscope slides with heat-killed sediment smears

Smear Preparation Procedure Review:

1. ARRANGE slides on staining rack, leaving space between each slide to prevent transfer of material and/or stain from one slide to another
2. APPLY Auramine-O stain, cover surface, and leave for **15 MINUTES** (Do not heat!)
3. RINSE slide gently with water, being careful to not wash off specimen.
4. DECOLORIZE the smear with Acid-Alcohol, let sit for a maximum of **2 MINUTES.**
5. RINSE slide gently with water. The auramine solution should be washed off, but if some stain remains add a little more acid-alcohol until it is completely colorless.
6. COUNTERSTAIN slide with Potassium permanganate solution for MAXIMUM **2 MINUTES.**
7. RINSE slide gently with water.
8. If any stain still remains on bottom of slide, gently wipe and remove with alcohol.
9. AIR-DRY slide prior to reading under the microscope. If slides are not read immediately, place in slide box.

REMEMBER!

-FM slides can fade after staining, therefore protect the stained slides from light (keep in a dark place) and read slides within 24 hours of preparation

-Prepare QC slides (negative and positive AFB) with each batch of slides stained.

-Record results on laboratory worksheet.

**Grading of FM Smears**		
**IUATLD/WHO Scale**	**FM (20x)**	**FM (40x)**
**Negative**	0 AFB/1 length	0 AFB/1 length
**Scanty**	1-29 AFB/ 1 length	1-19 AFB/ 1 length
**1+**	30-299 AFB/ 1 length	20-299 AFB/ 1 length
**2+**	10-100 AFB/field	5-50 AFB/field
**3+**	>100 AFB/field	>50 AFB/field

6. Culture Inoculations and MTB Identification

a. Background

The goal of mycobacterial culture is to detect viable mycobacteria in clinical samples. Mycobacteria grow on specific media after processing. Factors such as the rate of growth, the colony morphology, and the microscopic appearance of culture isolates help distinguish mycobacteria from contaminating flora. Liquid culture provides increased sensitivity and speed, while solid culture yields information about morphology and purity of culture. The higher the bacterial load in a sample, the greater the number of colonies in solid culture and the faster growth detection in liquid culture.

b. Precautions:

All culture work should be performed under a Biosafety Cabinet Class 2 in a BSL3 environment, utilizing Personal Protective Equipment (N95 mask, two pairs of disposable gloves and laboratory gowns). Follow procedures in Biosafety SOP for work with infectious substances.

c. Materials:

- Commercial MGIT media (7 ml)
- LJ-medium (Löwenstein Jensen medium)
- PANTA and other supplements
- Sterile plastic pipettes (5 ml)
- Pipettor (100 to 1000uL) and sterile aerosol-barrier (filter) pipet tips
- Clean biological Safety cabinet
- Incubator 37 ± 1 °C,
- MGIT 960 machine
- Materials for microscopic culture confirmation and speciation of culture isolates

d. Procedures:

- Prepare MGIT media by adding PANTA and other supplements in a freshly cleaned, empty BSC. This may be the Mycobacteria culture BSC if no bacterial work has been done for at least 30 minutes and the BSC fan has been running for at least 15 min but it is better to use a "clean" hood used for setting up clean media only.
- Another well-cleaned BSC that has been operating for at least 15 minutes with no bacterial samples inside may also be used to prepare the MGIT tubes and they may be transferred to the mycobacterial work BSC when sputum samples will be processed.
- Once the sediment is prepared, inoculate 2 LJ slants with 0.15 mL of sediment each (to align with NHLS) and one MGIT tube with 0.5ml of re-suspended pellet.
- Wait 30 min to insert tubes into the MGIT instrument.
- Incubate liquid media vials at 37°C for 6 weeks and LJ slants 37°C for 8 weeks checking as described in the experimental flow listed below.
- See Appendix A: FIND MGIT Manual for additional detailed information regarding inoculation and use of the MGIT platform for AFB culture.

e. Experiment flow and reporting of results:

<table>
<tr><th>Evaluation</th><th colspan="5">Procedure Flow and Reporting</th></tr>
<tr><td>1. MGIT growth report</td><td colspan="3">Positive:
Report: Culture Positive and Days: hours
If less than 5 days, return tube to MGIT logging it into system, when next flags positive continue to step 2</td><td colspan="2">No positive (at 42 days):
Review tube for possible bacterial button at bottom, if so continue tests, if not, report Culture negative, STOP</td></tr>
<tr><td>2. Visual inspection</td><td>Continue to 3 and 4</td><td colspan="2">Continue to 3 and 4</td><td colspan="2">Turbidity observed: Continue to 3 and 4</td></tr>
<tr><td>3. ZN Staining: observe for red bacilli (positive)</td><td>Positive: report AFB (+) and if blue bacteria or fungal hyphae observed, should report contaminated and AFB (+)</td><td colspan="2">Negative: report AFB (-) or if blue bacteria or fungal hyphae observed contaminated, should notify the site immediately for asking the patient to come back and leave one additional sputum if contaminated
If negative on 2nd test, report AFB (-) STOP</td><td>Positive: report AFB (+)</td><td>Negative: report AFB (-) STOP</td></tr>
<tr><td>4. Blood Plate incubated 48h</td><td>Growth (+):
Report Contaminated

Growth (-)
Record no growth in laboratory notebook

Continue to 5</td><td>Growth (+):
Report Contaminated, should notify the site immediately for asking the patient to come back and leave one additional sputum if contaminated

Decontaminate culture or sediment and resume testing at step 1 once

If (-) 2nd time report contaminated, STOP</td><td>Growth (-):
Return to Incubator for 7 days and resume testing at step 2 once.

If (-) 2nd time report no growth, STOP</td><td>Growth (+):
Report Contaminated.

Growth (-)
Record no growth in laboratory notebook

Continue to 5</td><td></td></tr>
<tr><td colspan="6"></td></tr>
<tr><td>5. MPT64 antigen detection</td><td>Positive: report MTB complex detected, STOP</td><td>Negative, continue to 6</td><td></td><td></td><td></td></tr>
<tr><td colspan="6"></td></tr>
<tr><td>6. Speciation tests real time PCR or PCR/sequence</td><td>Results can be NTM,</td><td>MTB complex</td><td>Or both MTB complex and NTM,</td><td colspan="2">or no mycobacteria detected depending on kit</td></tr>
</table>

At screening and/or Visit 0, the MGIT culture needs to be pure so that DST can be performed. DST is preformed from first positive pure culture. If MGIT is contaminated, then LJ can be used for DST evaluation.

If the MGIT culture is contaminated, the core laboratory staff will be responsible for informing the site data management person by telephone and WeChat. The person will notify study doctor and the doctor will contact the participant return to the site for an another sputum for culture. Telephone and WeChat contacts will be recorded in the core laboratory and site's telephone recording log. Researchers and lab technician are required to complete unexpected visit CRFs.

In later visits it is important to detect MTB if it is there, original culture isolates are saved for resistance generation detection

LJ MTB growth or no growth is a primary end point of the study so this is a **critical test**
Results are reported semi-quantitatively for LJ in the lab notebook:

# of AFB colonies	Result	
0	Negative	The length of incubation (in days) until<br>positive result is recorded (date positive <56 – date inoculated).
<10	Record number	
10-100	+	
>100	++	
Growth on entire LJ tube	+++	

**Evaluation**	**Procedure Flow and Reporting**				
1. LJ growth report	**Growth Positive:**<br>Review at 2-3 days and 1 week for colonies, if observed report **contaminated**, request another sputum sample immediately .<br>Continue to incubate slants and observe once weekly, once growth clearly visible with colony shape visible (3 weeks to 6 weeks typical), continue to step 2				**No growth (at 8 weeks):** if no colonies observed report Culture negative, **STOP**
2. Visual inspection	Single colony type< possibly mycobacteria<br><br>Report colony scale from WI		Possibly mixed mycobacteria and other colonies, pick dry corded colonies to streak on new slant and to test as below		
3. ZN Staining: observe for red bacilli (positive)	**Positive:** report AFB (+) and if blue bacteria or fungal hyphae observed report contaminated and AFB (+)<br><br>**Continue to 4**	**Negative:** report AFB (-)<br><br>**STOP**	**Positive:** report AFB (+) and if blue bacteria or fungal hyphae observed report contaminated AFB (+)<br>**Continue to 4**	**Negative:** report AFB (-)<br><br>**STOP**	
4. Check MGIT result	**MTB complex detected:** report MTB complex detected<br><br>**STOP**	**MTB complex not detected:**<br><br>**Continue to 5**			
5. MPT64 antigen detection	**Positive:** report MTB complex detected,<br><br>**STOP**	**Negative:**<br>Report MTB complex not detected.<br><br>Only if Doctor requests NTM Speciation tests, **Continue to 6**			
6. Speciation tests: Real	Results can be NTM,	MTB complex	Or both MTB complex and	or no mycobacteria detected depending on kit	

time PCR in China or PCR sequence			NTM,	

7. STORING RAW SPUTUM FOR LAM ANALYSIS

For each visit where a Xpert test will be conducted, a sample for LAM analysis will be saved as described in Section 2e. LAM analysis will be conducted in batches later in the study with reagents supplied by Otsuka Corp. following the instructions from their kit and a ELISA reader.

- Any sputum sample (overnight, fresh, or induced) may be used for this sample. If possible use excess from the culture sample for LAM.
- Record which sputum sample has been used for this sample in the sputum notebook log. Use this log to record the sample storage on the sample CRF.
- Using a positive displacement pipetter and matching tip, transfer 0.2 mL of sputum into a barcode-labeled 2 mL sterile cryotube with screw cap lid and O-ring (also described in section 2e).
- Freeze at -20 to -80 for short term storage. Transfer on dry ice if necessary and maintain at -80°C for long term storage.
- Once frozen, ship on dry ice in batches if the sample must be moved to another facility for analysis.

8. STORING RAW SPUTUM FOR OTHER BIOMARKERS

- Once the sputum for culture and other specimens are stored, pipet the remaining sputum in 1 or 0.5 mL aliquots into barcode labeled 2 mL sterile cryotubes with screw cap lids and O-rings.
- Pipet any induced sputum in 1 mL aliquots into barcode labeled 2 mL sterile cryotubes with screw cap lids and O-rings and record the aliquot numbers that are from induced sputum
- Record the subject ID, sample type (raw sputum or induced sputum), which sputum sample it came from originally, and number of aliquots on the sample log and use this information to complete the sample CRF.
- Snap freeze in liquid N2 or on an ethanol + dry ice slurry if possible. Freezing in ethanol + dry ice slurry at -20°C is also an option, transfer to -80°C for long term storage.
- Once frozen, ship on dry ice in batches if it must be moved to another facility for analysis.

9. Drug Susceptibility Testing

General Approach

Determine the number of specimens to be set up for drug susceptibility test (DST) assays that day and in a cleaned, empty BSC that has had the fan blowing for at least 30 minutes, put on fresh gloves and label and prepare all the MGIT tubes adding the PANTA and drug dilutions that will be needed for the procedures before beginning to handle the bacterial cultures.

Once the bacteria culture work has begun, work with one culture specimen at a time, making the culture dilutions and inoculating the set of MGIT tubes for that culture before beginning work with the second or subsequent cultures. Change outer gloves frequently but <u>at a minimum between each specimen</u> ID. If in doubt that the gloves are still clean, change the outer gloves.

a. Primary Drug Susceptibility Testing (SIRE)

Guidelines on the use of the primary MGIT culture for DST testing.
Take out the MGIT960 liquid culture tubes that were reported to be positive between 1 to 5 da y (1) The day a MGIT tube is positive by the instrument is considered Day 0). Hold (+) MGIT tubes in a 35-37 C incubator while waiting set up of DST tests. If growth in a tube is on Day 1 or Day 2 after positive, mix well (vortex) to break up clumps. Leave the tube undisturbed for about 5-10 minutes to let big clumps settle on the bottom. Use the supernatant undiluted for inoculation of the drug susceptibility test (DST) tube set.

(2) If growth is on Day 3, 4, or 5 after positive, mix well to break up the clumps or use sonication disrupter (confirm the time needed). Let the large clumps settle for 5-10 minutes and then dilute 1.0 ml of the positive broth with 4.0 ml of sterile saline. This will be a 1:5 dilution. Use this well mixed diluted culture for inoculation of the DST tube set. If Growth Day 6 or after, inoculate new MGIT tube and incubate until reported to be positive (then follow steps above).

(3) If necessary scratch off some colonies from the LJ culture into 2 mL of PBS for the DST. Use the disrupter (confirm time needed) until the turbidity is equal to 1 McFarland standard. Dilute this liquid by 100 and 10,000.

(4) Calculations of the dilution factor for MGIT medium: 7.0 ml of medium + 0.8 ml of SIRE Supplement + 0.5 ml of inoculum = 8.3 ml. Addition of 0.1 ml of the drug solution in 8.3 ml of the medium = 1:83 dilution. Add the calculated amount of each drug to all of the supplemented MGIT tubes prior to beginning to work with the bacterial cultures.

(5) Prepare the cultures: For the control, first dilute the test culture suspension 1:100 by adding 0.1 ml of the test culture suspension to 10.0 ml of sterile saline. Mix well by inverting the tube 5-6 times. Use this diluted suspension to add 0.5 ml into the growth control tube. Prepare the inoculum for the drug-containing MGIT tubes as described above based on their age and add 0.5 ml into the drug-containing tubes.

(6) Tighten the caps and mix the inoculated broth well by gently inverting the tube several times.

(7) Enter the susceptibility set carrier into the BACTEC MGIT 960 instrument using the susceptibility test set entry feature.

(8) Once the test is complete (within 4 to 13 days), the instrument will indicate that the results are ready. Scan the susceptibility Set Carrier and print the report. The instrument printout indicates susceptibility results for each drug. Results are qualitative: Susceptible (S), Resistant (R) or indeterminate (X). If there is an error (E400) on any of the drugs tested, repeat testing once for that drug only, following the above procedures. Record the error type or contamination on the lab log records, and repeat the tests, recording also in the lab log record.

10. Specimen and Isolate Storage

a. Background

It is the role of the site laboratories to ensure that there is a system to collect and store specimens properly, monitor the proper functioning of equipment including freezers, and maintain accurate records of what samples are in storage and sample history. Records will include any removal of the partial contents of a vial, and any freeze/thaw cycles.
The initial strains of each participant must be shared with the CDC. Since LJ's are made, if two is positive, one of them can be transferred to CDC, if only one is positive, inoculate a new LJ from the positive one so as to give them a positive culture.

b. Precautions

Universal safety precautions and Personal Protective Equipment (PPE) appropriate to the Biological Safety Level must be followed by laboratory personnel. For all the steps performed below, appropriate PPE (two pairs of disposable gloves, protective clothing, and N95 respiratory mask (when applicable)) is required. Utilize a face shield or goggles when handling non-infectious specimens outside of a safety cabinet where there is risk of splashing/aerosol creation. When in doubt refer to your standard laboratory guidelines for biosafety policies and procedures.

c. Procedures

Biological specimens stored under this SOP will include processed sputum sediment and *M. tuberculosis* isolates. Samples will be de-identified in regards to participant information, with only laboratory and study ID numbers used as references. The specimen database will include the following fields with necessary information:

- Specimen type
- Specimen collection date
- Participant Identification number
- Vial number
- Vial volume
- Date of storage at -20°C and -80°C

• Final diagnosis with respect to TB

I. Processing of Sputum Sediments for Storage

After the inoculation of MGIT and LJ media (and Xpert test as required) with the processed sputum sediment, along with any remaining aliquot of the sputum sample should be frozen for biomarker use.

(1) Seal the cryovial with a thin layer of parafilm.
(2) Freeze at -80°C. Prior to freezing at -80°C, the specimen may remain at approximately 2-8°C (refrigerator) for up to 24 hours, and/or at -20°C for up to 5 days.
(3) The specimen should not be allowed to thaw after it is frozen.

II. Processing of Cultures for Storage

Cryopreservation of MGIT cultures

(1) Bacteria are preserved so that they may be cultured again if necessary and for use to prepare DNA for sequencing. Bacteria for later culture are preserved in M7H9 medium with OADC and 20% glycerol. Bacteria for later sequencing are preserved in Tris EDTA.
(2) Transfer the liquid in a positively-cultured MGIT tube to 2 sterile centrifuge tubes; with 2ml culture in one tube and 1 to 2 ml in the second tube; centrifuge 10 min at 4000 rpm; remove supernatant.
(3) In the tube with the pellet from 2 mLs of culture, add 2 ml of 7H9+OADC+ 20% glycerol medium, cap the tube, and resuspend the pellet by vortexing or pipetting up and down with a filtered pipette tip (this is critical that the tip be filtered) and divide the bacteria solution into to 3 cryopreservation tubes.
(4) In the tube with the pellet from 1 ml of culture, blot dry, and add 500µl of TE buffer and resuspend the pellet as above, to be used for later heat killing and extraction of DNA.

Cryopreservation of LJ-grown cultures

(1) Bacteria are preserved so that they may be cultured again if necessary and for use to prepare DNA for sequencing. Bacteria for later culture are preserved in M7H9 medium with OADC and 20% glycerol. Bacteria for later sequencing are preserved in Tris EDTA.
(2) All of the colonies on the slant of LJ medium (if containing only 1 type of colony that is also acid-fast) should be scraped with transferring loop and placed in sterile grinding tube with 0.5 ml of sterile normal saline. The colonies are then pulverized.
(3) A sterile pipette with a filter-tip is used to aspirated 0.5 ml of bacterial suspension and is added the centrifuge tube with 500µL of TE buffer for preservation at -80 °C

(4) The remained bacteria solution is added to 2 ml of 7H9+OADC+20% glycerol medium and is then divided into three centrifuge tubes for preservation

11. Bacterial DNA Sequencing (check translations – text changed)

DNA sequencing will occur on isolates from participants that are thought to have recurrent TB. When a sample from recurrence visit is identified as culture positive for MTB, the isolate from their screening or 0 visit freezer stock will be cultured on LJ media until colonies are 1 to 3 mm in diameter. Bacteria used for DNA isolation is prepared by first killing the bacteria by placing several colonies into 0.5mL Tris-EDTA buffer and heating for just 30 minutes at 80 °C. The sample is then frozen at -80 °C until shipping. The final DNA isolation work will be undertaken at Fudan University, and sequencing will follow standardized operating procedures found in the Gao laboratory.

This work instruction has been adapted from those prepared by CDRC's Dereck Armstrong.

12. SALIVA for BIOMARKERs

1.1.1. Timing and responsibility

Saliva will be collected while the patients are fasting at the 3 scheduled PET/CT visits and at any recurrence investigation.

The technical staff accompanying the subject for the PET/CT will be responsible for collecting the salivettes and completing the storage of the saliva at HPCH

M. tuberculosis has been found in saliva, full personal protective equipment (gloves, masks, etc) should be worn when handling and pipetting saliva samples

1.1.2. Materials

- 3 Salivette tubes per patient
- Supplier: Sarstedt
- Catalogue number: 51.1534 "Cotton swab without preparation"
- Ice chest and wet ice or cold packs in transport box
- Centrifuge
- 1ml pipettor
- Sterile Pipette tips
- Ice and Ice chest
- Sterile Barcoded 1.6 to 2 mL Cryo vials
- Scanner and computer for entry into database
- Barcoded Freezer Box, final location entered into database
- -80 for storage

1.1.3. Summary of each visit

- Saliva is collected from 3 chews for each patient
- Does not have to be sequential, but should be done before patient is fed,
- Coordinate timing of collection prior to administering [18F] FDG if on the same day as the PET scan so that the samples do not become radioactive.

1.1.4. Preparation

- Label the salivette tubes with patient ID, name, date and time of collection (temporary labels- no bar code required)
- Record when the participant last ate or drank (at least 1 hour earlier)
- Remove the swab from the first salivette
- Place the swab in the mouth of the participant with tweezers
- The participant chews the swab for at least 45 seconds to stimulate salivation
- Collect the swab with tweezers or have the subject place the swab back into the salivette tube without touching it.
- Store the chewed salivettes on cold packs in a transport container or in a refrigerator until the samples are taken to the lab
- *Repeat collection* of saliva twice, timing according to the comfort of the patient and before administration of FDG
- Process the samples within 2 hours

1.1.5. Saliva Processing and Aliquoting

- Centrifuge salivettes in a centrifuge for 2 minutes at 1,000xg (2900 rpm in HPCH machine)
- Keep all materials on ice between processing steps
- Working in a biological safety cabinet, pipette the saliva from the bottom of the conical tubes
- Yield expected is approximately 1.1ml ± 0.3ml per salivette
- Aliquot the saliva into 4 barcoded plastic 1.6 to 2 mL cryotubes (~ 0.5 mL each) and record onto sample log for later entry into sample CRF
- Scan into database according to scanning protocol and store in -80 ℃ freezer
- when samples are shipped for analysis they must be shipped on dry ice to prevent thawing in transit
