# Supplementary material for: Using biomarkers to predict TB treatment duration (Predict TB): a prospective, randomized, noninferiority, treatment shortening clinical trial
Source: Gates Open Res. 2017 Nov 6;1:9. [Version 1] doi: 10.12688/gatesopenres.12750.1 (PMC5841574; doi:10.12688/gatesopenres.12750.1)
Supplement: Supplementary file 4 [file gatesopenres-1-13810-s0003.tgz › 3e68301b-f1a3-4840-93f5-6eca40568fcb.pdf]

9 CLINICAL SITE LABORATORY MANUAL (CONTINUATION OF MOP)

9.1 SUMMARY OF SPECIMENS AND TESTS

For each visit, specimens will be collected, processed and temporary storaged (if necessary) at the sites, and be transported to the core laboratory of Henan CDC for tests or long-term storage. Refer to the Predict Sample FLIPBOOK for China for pictures of what samples and supplies are needed at each visit. Sputum for culture and biomarkers will be transported cold but not frozen unless specified.

Table 1 Lists which samples are collected in China at different time points and indicates the responsibility as either clinical site labs or core lab. Recommended sputum volumes are included below.

China	Screen	D0	W1	W2	W4	W8	W12	W16	W20	W24	W36	W48	W72	RECUR
	Clinical sites[a]													
Safety Bloods	√													√
Pregnancy test	Serum / Urine	Urine			Urine			Urine *		Urine *				Urine
Blood biomarkers		√	√	√	√	√		√		√		√	√	√
Urine biomarkers		√	√	√	√	√		√		√		√	√	√
Blood Drug Monitoring									√					
Sputum collection	≥3.5 mL	≥7.5 mL	≥3.5 mL	≥6 mL	≥7.5 mL	≥7.5 mL	~3 mL	≥7.5 mL	~3mL	≥7.5 mL	~3 mL	≥6 mL	≥7.5 mL	≥7.5 mL
Sputum + Trizol		√		√	√	√		√		√			√	√
Sputum GeneXpert	√[a]	**b			√[b]	√[b]		√[a]		√[b]				√[b]
	Core lab staff[b]													
Sputum Culture (spontaneous production)	√	√	√	√	√	√	√	√	√	√	√	√	√	√
Sputum LAM		√			√	√		√		√			√	√
Sputum Biomarkers		√		√	√	√		√		√		√	√	√
Saliva biomarkers		√			√			√ *		√ *				√

* Performed at W16 or W24 based on PET/CT randomization
** Only performed if screening GeneXpert was >7 days prior
[a]: performed by clinical sites (blue)
[b]: performed by core lab (gray

Samples should be collected in the following order for each visit to control for stress hormone generation in the samples during the visit. Specifically, all other samples should be collected before sputum induction is undertaken.

1. Spontaneous sputum
2. Blood: red top- Serum
3. Blood: lavender top- EDTA (host DNA/PK samples)
4. Blood: PAXGene tube
5. Urine
6. Induced sputum

9.2 LABELING, BARCODING, AND STORING OF SPECIMENS

9.2.1 REQUIRED LABELING FIELDS (INCLUDE AN EXAMPLE)

There will be two types of labels primary and secondary. The primary label are the ones that will be placed on the blood collection tubes/sputum collection cups/urine cups that will not be stored in freezers. The secondary labels are usually the ones derived from the primary labels and will be stored in -80°C freezers.

Primary Label

1) Fields on the label
    - Participant ID: PD-2-1-001 (Study identifier, country code, site code, participant sequential number), printed
    - Sample type: printed
    - Visit: printed
    - Date: sample collection date, written
    - Time: sample collection time, written
    - 2D barcode
    - Sputum sample numbers 1, 2, 3, 4 can be written under Sample Type area
2) Size: 2x1 label

2x1 label

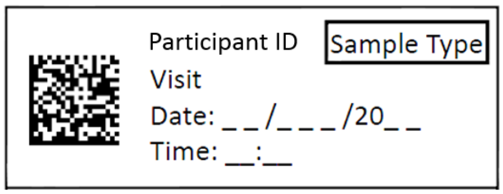

Secondary Label

1) Fields on the label
    - Participant ID: PD-2-1-001 (Study identifier, country code, site code, participant sequential number), printed
    - Sample description: printed
    - Aliquot number: 1, 2, 3, 4, printed
    - 2D barcode
2) Sizes:
    - 2x1 label for big storage tubes

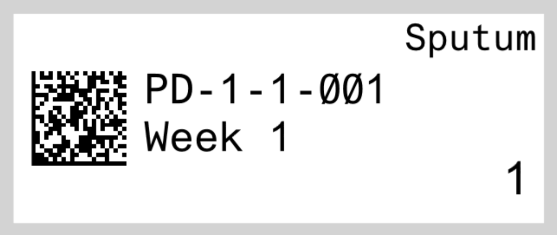

- Small label for Frozen (2mL) tubes. The round label with barcode will be placed on the top of the Frozen tube. The rectangle label will be placed on the side of the tube.

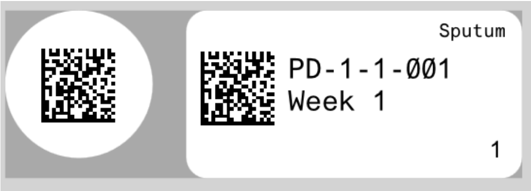

9.3 LABORATORY QUALITY MANAGEMENT

9.3.1 TEMPERATURE MONITORING

- Any refrigerators, freezers, or other equipment meant to operate at a specific temperature range and used for study work must be monitored for that temperature on working days.
- Acquire calibrated thermometers for the temperature range to be monitored from a reputable vendor and place one into each piece of equipment to be monitored.
- Make a log (paper is easiest) on which to record the temperature with a title of the piece of equipment and recommended range, along with columns for the temperature, date, and initials or name of the person observing and recording the temperature.
- These logs should be filled out on each working day and stored in an organized fashion (a binder or other folder) for review by the protocol monitoring team during monitoring visits.
- Logs can be discarded 3 years after the trial is complete or after instructed in writing by the protocol monitoring team.

9.3.2 RECORD KEEPING

- All documents created while doing study work should be signed and dated by the persons doing the work and must be stored (if a scrap of paper is used as a primary document – that must be signed and stored)
- All data collected (and entered on a CRF) must have a source document to support the data
- All records must be kept until 3 years after the trial is complete or after instructed in writing by the protocol monitoring team.

9.4 SPUTUM

China Overview

1) All culture (MGIT and LJ) will be done in the core lab at the H-CDC (the county sites do not do concentration method culture or liquid culture generally so all culture will be done at the H-CDC).
2) For screening, the individual sites must smear 3 separate sputum (country TB program rules) first and then if the doctors still suspect TB each site has a G4 Xpert system with which to do Xpert MTB/RIF on raw sputum.
3) The purpose of the sputum mRNA sample (sputum + TRIzol) is to get a snapshot of the mycobacterium tuberculosis transcription as it is in the patient's body. The more rapidly the sample is preserved with TRIzol the more realistic this picture is- TRIzol should be added as soon as possible and within 10 minutes of production.
4) The sites will submit all remaining sputum and any additional sputum cups to the core lab for culture
5) At visits other than week 16, the sputum will be transported to the core lab for both culture and Xpert tests.
6) Because all the sites have a G4 and transport may be delayed up to 48h, the individual sites will do Xpert MTB/RIF on raw sputum of the week 16 sputum sample so that the visit can be one day and submit the remaining sputum to the core lab for culture (transport is about every 48hrs to the core lab).
7) Summary of sputum handling

	**Processing at the sites**	**Storage at the sites**	**Transport to Core lab**
**Sputum for Culture, Xpert**	- Refrigerate 2-8°C immediately	None	Within 48h (cooler box, 2-8°C)
**Sputum + TRIzol for mRNA**	- Mix with TRIzol as soon as possible (<10 min)<br>- Hold on ice until dispensed and frozen	-80°C or the coldest available	Bulk transport/dry ice roughly monthly
**Sputum for Biomarkers**	- Refrigerate 2-8°C immediately	None	Within 48h (cooler box, 2-8°C)

9.4.1 SPUTUM COLLECTION PROCEDURES

Label the sputum collection container with identifying label including PID, specimen type, and blanks for date and time of collection. Provide the container(s) to the participant and instruct him in sputum collection as described below. A bar coded label may also be added to the container if desired.

Follow site-specific procedures for where the patient expectorates the sputum. If specimen is collected at home rather than at a clinic, instruct the patient to follow precautions to avoid other household members. Preferably, specimen should be collected outside of the dwelling.

Obtaining high quality sputum without saliva contamination is the goal. Explain to the participant to rinse his/her mouth with water to remove the saliva. If he/she cannot do this, then ask him/her to spit out the saliva in his/her mouth as much as possible before expectorating.

Instruct the participant not to expectorate saliva or postnasal discharge into the container but rather a specimen resulting from a deep cough into a sterile screw-cap cup or sterile sputum collection container. The participant should continue trying to expectorate until the required volume of sputum has been collected (there is not a time requirement other than before the visit is over). Show

the mark on the container(s) to the participant and instruct the participant to place the sputum specimen container in the biohazard specimen bag.

Sputum induction according to the sputum induction SOP should be strongly considered for participants who are no longer able to expectorate sputum spontaneously, particularly at weeks 16, 24, 48, and 72.

If for any reason the staff member needs to enter the room to assist the patient, the staff member should put on a properly fitted, NIOSH-approved HEPA particulate respirator and disposable gloves. The staff member should not remove the respirator until he or she has left the space where the participant is coughing.

The staff member must put on gloves to receive specimens from participant. Place specimen(s) in the designated area or refrigerator, until it is delivered to the laboratory (do not freeze).

Complete lab testing order sheet and place the sheet in the pocket of the biohazard specimen bag to identify each specimen.

9.4.2 **SPUTUM TRANSPORT (See 9.8 section)**

9.4.3 GENE XPERT USING RAW SPUTUM **(screening and week 16 China clinical sites only)**

The GeneXpert system is used to detect the presence of MTB DNA and rifampicin (RIF) drug resistance in expectorated sputum specimens.

Equipment:

- GeneXpert System
- Biosafety cabinet

Materials:

- GeneXpert kits (contains Cartridges, Sample reagent buffer and sterile disposable transfer pipettes)
- Vortex Mixer
- Conical screw-capped tube and test tube rack
- Positive displacement pipettor and sterile tips
- Timer
- Laboratory Worksheet/CRF form
- Labels and indelible labeling marker
- Miscellaneous (Disposable gloves, N95 mask, lab coat etc.)

Precautions, Use and Storage:

Safety Precautions: Universal precautions should be used when handling all biological specimens. Wear appropriate PPE (Personal Protective Equipment) including 2 pairs of protective disposable gloves, N-95 masks, laboratory coats and eye protection when handling specimens and reagents. After handling specimens and reagents, wash hands thoroughly. When in doubt, refer to the safety procedures set forth by your institution for working with chemical and biological samples.

Specimen Use: Specimens utilized for this assay will be collected per standard laboratory protocols for specimen collection. Sputa collected will be held at 2-8°C prior to processing if the delay in processing is more than 6h.

Equipment and Material Use and Storage:

- Store the GeneXpert cartridges and reagents at 2-28°C.
- Keep a log of the temperature in the place where cartridges are stored
- Keep a log of the temperature in the room where the test is performed. The test **can not** performed in a room above 30 °C. Turn on the cooling in the room if necessary until the room is within the temperature range for valid test performance.
- Do not open a cartridge until testing will be performed. Use the cartridge within 30 minutes of opening the lid. The cartridge is stable up to 7 days after opening the package.
- Follow manufacturing instructions and standard laboratory protocols for usage and storage of other routine equipment. Once completed, dispose of used equipment into proper biohazard containers.

Test Procedures:

Sample Preparation (Perform in Biosafety Cabinet):

1) Label each GeneXpert assay cartridge with the sample ID or affix ID label. Do not place the label on the lid of the cartridge or obstruct the 2D barcode on the cartridge.
2) The minimum initial volume for testing is 0.75mL of raw sputum. In the biosafety cabinet, carefully open the oldest sputum container, measure the initial volume of sputum with a positive-displacement pipettor set to 0.75 mL, and if there is not enough sputum to perform the test do not continue further with this specimen, review the other specimens and choose one with a larger volume, saving the first one for other uses.
3) If using only part of the specimen, mix the sputum with the pipettor or glass beads
4) Transfer 0.75 mL of sputum into a 15 mL tube and add 1.5 mL Sample Reagent, (2:1 (v/v) directly to sample).
5) Re-cap the tube, and invert the tube vigorously 10-20 times, avoiding the creation of bubbles. (Note: One back and forth motion is a single inversion)
6) Incubate the sample for 15 minutes at room temperature in the hood.
7) After 5-10 minutes of this incubation, mix the sample again either by inverting or snap vortexing for 5 seconds, avoiding the creation of bubbles.
8) (Note: Samples should be liquefied with no visible clumps of material; if there are remaining clumps shake again as above and incubate for another 5 to 10 minutes.)

Cartridge Preparation and Test Initiation:

(Note: Start testing within 30 minutes of sample addition to the cartridge)

1) Using a sterile transfer pipette, measure the liquefied sample. If there is less than 2 mL volume, do not process the sample further.
2) Open the cartridge lid, and use a graduated transfer pipette to transfer 2mL of sample into the open port of the GeneXpert cartridge. Dispense slowly to avoid aerosol formation. If adding sample to multiple GeneXpert cartridges, use separate transfer pipettes for each sample.

3) Close the cartridge lid, making sure it snaps firmly into place. If additional liquefied sample remains, it may be kept for up to 12 hours at 2-8°C if repeat testing is required. (See section below entitled "Repeat testing").
4) Scan the barcode on the Assay Cartridge, and scan/type in the sample ID into the software.
5) Once instructed by the G4, load cartridge into place, and start test.

Results:

1) Results Interpretation and Recording: Conventional MTB/RIF Xpert:
   Once testing is complete, print out the assay results PDF for each sample to file in the subject binder and record results on the corresponding CRF, indicating whether MTB positive or negative, the CT values for MTB detection and RIF resistance information.

2) Repeat Testing:
   The decision to repeat testing on either platform will be made on-site. Repeat testing will occur one time, and will be based on the following test outputs:

   MTB/RIF Xpert: Results indicate ERROR, INVALID TEST or NO RESULT, if this occurs at screening or week 16, the assay will need to be repeated. This information will need to be recorded on a laboratory worksheet and the CRF for quality management purposes. If repeat will occur from sample reagent (SR) treated sample, it must occur within 12 hours of SR addition.

Quality Control:

Sample Processing Control (SPC)—Ensures the sample was correctly processed. The SPC contains non-infectious spores in the form of a dry spore cake that is included in each cartridge to verify adequate processing of MTB. The SPC verifies that lysis of MTB has occurred if the organisms are present and verifies that specimen processing is adequate. Additionally, this control detects specimen-associated inhibition of the real-time PCR assay. The SPC should be positive in a negative sample and can be negative or positive in a positive sample. The SPC passes if it meets the validated acceptance criteria. The test result will be "Invalid" if the SPC is not detected in a negative test.

Probe Check Control (PCC)—Before the start of the PCR reaction, the GeneXpert Dx System measures the fluorescence signal from the probes to monitor bead rehydration, reaction-tube filling, probe integrity and dye stability. Probe Check passes if it meets the assigned acceptance criteria.

Refer to the Cepheid System Operator Manual for additional information on Diagnostic Testing and Troubleshooting

9.4.4 SPUTUM FOR BACTERIAL mRNA ANALYSIS

The purpose of this sample is to get a snapshot of the mycobacterium tuberculosis transcription as it is in the patient's body. The more rapidly the sample is preserved with TRIzol the more realistic this picture is. If the patient has a freshly produced sputum) and at least 6 mL of total sputum samples (induced or

spontaneously produced), follow the procedure below to save a sample for mRNA analysis at the visits indicated in Table 1. **The lab should try to process this sample immediately (less than 10 minutes!).**

1) Record the time the subject produces the sputum on the source document, along with time of TRIzol addition and other information about processing for each specimen.
2) Sputum should be not be chilled (hold approximately 18-25°C) until after it is mixed with TRIzol. Mix sputum immediately into TRIzol (less than 10 minutes). If it cannot be processed within 30 minutes, do not store it for mRNA. Submit the sample to the core lab/ UCT lab for other uses.

3) Place the fresh sputum sample in the biosafety hood and wear an N95 mask, two pairs of gloves, and a lab coat for the following work.

4) If the sputum volume is 2mL or less, just add the TRIzol to the sample and add an equal volume of TRIzol (1:1 ratio), pipette up and down 20 times or more to mix, recap, place into a zipper plastic bag, and vortex 1 minute to mix well. Pipette up and down again if the sample is still not mixed well. If the sputum volume is more than 2mL, will transfer 1.5 mL of sputum using a positive-displacement pipettor to a new > 5 mL tube and add an equal volume of TRIzol (1:1 ratio), pipette up and down several times to mix, recap and vortex 30 seconds to mix well.

5) If the sample is more than 2 mL, homogenize the sputum by pipetting up and down 20 times or more to break up any clumps of sputum with the positive displacement pipettor or shred the sputum with 8 to 12 sterile 3-4 mm glass beads for 30 seconds on a full speed vortex.

6) Transfer 1.5 mL of sputum using a positive-displacement pipettor to a new bigger tube and add an equal volume of TRIzol (1:1 ratio), pipette up and down 20 times or more to mix, recap and vortex 30 seconds to mix well.

7) Dispense the sputum-TRIzol mixture into 2 or more preprinted bar-code labeled 2 mL cryotubes (1.8 mL or less per tube) with screw-on lids and O-rings.

8) Record the information and number of samples on to the sample log and enter the samples onto the sample CRF.

9) Snap freeze in liquid N2 or on an ethanol + dry ice slurry (preferred) or place in the snow on a freezer shelf until frozen solid.

10) Transfer the cyrotubes to freezer boxes and freeze at -80 C until transportation to long term storage. If a -80 freezer is not available at the clinical site, hold in the coldest freezer available and transfer the samples to long term storage within one to two weeks.

9.4.5 CORE LAB CHINA CULTURE PROCEDURES OVERVIEW

This information is provided for the site's reference, all cultures will be done at the core lab. LJ and MGIT culture procedures in the core lab in China will follow the following tables. For detailed procedures, please refer to the relevant Core lab SOP. Results will be returned to the site in a report for the physicians reference.

**Evaluation**	**Procedure Flow and Reporting**				
1. LJ growth report	**Growth Positive:**<br>Review at 2-3 days and 1 week for colonies, if observed report **contaminated**, <u>request another sputum sample</u> immediately. Continue to incubate slants and observe once weekly, once growth clearly visible with colony shape visible (3 weeks to 6 weeks typical), continue to step 2				**No growth (at 8 weeks):** if no colonies observed report <u>Culture negative</u>, **STOP**
2. Visual inspection	Single colony type< possibly mycobacteria<br><br>Report colony scale from WI		Possibly mixed mycobacteria and other colonies, pick dry corded colonies to streak on new slant and to test as below		
3. ZN Staining: observe for red bacilli (positive)	**Positive:** report <u>AFB (+)</u> and if blue bacteria or fungal hyphae observed report <u>contaminated and AFB(+)</u><br><br>**Continue to 4**	**Negative:** report <u>AFB (-)</u><br><br>**STOP**	**Positive:** report <u>AFB (+)</u> and if blue bacteria or fungal hyphae observed report <u>contaminated and AFB(+)</u><br><br>**Continue to 4**	**Negative:** report <u>AFB (-)</u><br><br>**STOP**	
ONLY IF NO MTB RESULT on MGIT CULTURE					
4. MPT64 antigen detection	**Positive:** report <u>MTB complex detected,</u> **STOP**	Negative, continue to 5			
ONLY IF NO MTB RESULT on MGIT CULTURE and requested by physician					
5. Speciation tests: Real time PCR in China or PCR sequence	Results can be <u>NTM,</u>	<u>MTB complex</u>	Or both <u>MTB complex and NTM,</u>	or <u>no mycobacteria</u> detected depending on kit	

MGIT procedure:

**Evaluation**	**Procedure Flow and Reporting**			
1. MGIT growth report	**Positive:**<br>Report: <u>Culture Positive</u> and <u>Days: hours</u><br>If less than 5 days, return tube to MGIT logging it into system, when next flags positive continue to step 2		**No positive (at 42 days):**<br>Review tube for possible bacterial button at bottom, if so continue tests, if not, report <u>Culture negative</u>, STOP	
2. Visual inspection	Continue to 3 and 4	Continue to 3 and 4	Turbidity observed: Continue to 3 and 4<br>No turbidity, STOP	
3. ZN Staining: observe for red bacilli (positive)	**Positive:** report <u>AFB (+)</u> and if blue bacteria or fungal hyphae observed and <u>contaminated and AFB(+)</u>	**Negative:** report <u>AFB (-)</u> or if blue bacteria or fungal hyphae observed <u>contaminated</u><br><br>**<u>REQUEST another specimen from site if contaminated</u>**<br><br>If negative on 2nd test, report <u>AFB (-)</u> **STOP**	**Positive:** report <u>AFB (+)</u>	**Negative:** report <u>AFB (-)</u> **STOP**

4. Blood Plate incubated 48h	Growth (+): Report Contaminated<br><br>Growth (-) Record no growth in laboratory notebook<br><br>**Continue to 5**	Growth (+): Report Contaminated **REQUEST another specimen from site**<br><br>Decontaminate culture or sediment and resume testing at step 1 once<br><br>If (-) 2nd time report contaminated, **STOP**	Growth (-): Return to Incubator for 7 days and resume testing at step 2 once.<br><br>If (-) 2nd time report no growth, **STOP**	Growth (+): Report Contaminated.<br><br>Growth (-) Record no growth in laboratory notebook<br><br>**Continue to 5**	
5. MPT64 antigen detection	**Positive:** report MTB complex detected, **STOP**	Negative, continue to 6 if requested by physician			
6. Speciation tests real time PCR or PCR/sequence	Results can be NTM,	MTB complex	Or both MTB complex and NTM,	or no mycobacteria detected depending on kit	

9.5 BLOOD

9.5.1 BLOOD COLLECTION PROCEDURES

Blood should be collected per local standard operating procedure.
Blood should be collected into the following tubes for the following tests:

SAFETY TEST	Tube	Notes
Liver function tests (AST/ALT)	Red, red/gray marble, or gold top vacutainer tubes	Collect up to 5 mL at the screening visit and at recurrence. After blood collection, tube should be gently inverted about 5 times to ensure mixing of clot activator with blood. Blood should be allowed to clot, then centrifuged and processed per local SOP.
HIV		
CRP, creatinine		
Qualitative serum hCG (if applicable)		
Full blood count	Lavender top vacutainer EDTA	Collect up to 5 mL per visit. After blood collection, tube should be gently inverted about 8 times to ensure mixing of EDTA anticoagulant with blood to prevent clotting.
Hemoglobin A1c		
BIOMARKER tests	Tube	Notes: collect blood tubes in this order
Serum	red top, no additive	Collect approximately 4-5 mL X 2 tubes

Host DNA	Lavender top vacutainers EDTA	Collect approximately 1-2 mL of blood for each sample Gently invert tube 6 to 8 times to mix.
Whole Blood cells or PBMC	Green top tube (sodium heparin)	Collect approximately 10 mL X 4 tubes. Gently invert tube 6 to 8 times to mix.
Host mRNA	PAXgene tube (brown top)	Collect approximately 2.5 mL in one tube. When PAXgene tubes are being drawn, the PAXgene tube should be the last tube drawn. Ensure the blood has stopped flowing into the tube prior to removing the tube from the holder. Gently invert the PAXgene tube 8 to 10 times to mix.
PK ANALYSIS	Tube	Notes
Plasma for PK analysis	Lavender top vacutainers EDTA	Collect approximately 1-2 mL for each sample Gently invert tube 6 to 8 times to mix.

9.5.2 BLOOD PROCESSING AND TESTING

- ***SAFETY BLOODS—CBC/Chemistries/Liver Function Tests***

Safety lab will be processed by each individual site. Tests will be done per standard of care for each site. The table above contains the recommended tests.

For results that are out of range, staff should confirm whether the out of range results indicate the participant is not eligible for the study. For safety bloods during the study, test results should be reported to the study doctor who should follow standard site procedure for further testing or monitoring.

- ***PREGNANCY TESTING***

The screening pregnancy test is done on serum or urine for all females. Subsequent pregnancy tests are done on urine.

- ***HIV TEST***

An HIV test will be performed as part of screening, and if HIV negative and subsequently enrolled, will be only be performed again if the participant has TB recurrence.

9.5.3 BLOOD BIOMARKERS

Summary table for reference

Specimen	Processing at the sites	Storage at the sites	Transport to Core Lab
**Blood Biomarkers: serum**	- >30 min at RT[2]<br>- Separation of serum/storage within 2 hr preferred	-80°C preferred	Bulk transport (dry ice)
**Blood Biomarkers: mRNA**	- at least 2 hr at RT[2]<br>- Freeze at -20°C overnight	-80°C preferred	Bulk transport (dry ice)

**Urine Biomarkers**	- Refrigerate[1] immediately<br>- Aliquot/store within 6 hr	-80°C preferred	Bulk transport (dry ice)
**Saliva Biomarkers**	- Collected during PET/CT procedure<br>- Refrigerate or keep on ice, freeze ASAP	None	None
**Blood PK**	- Refrigerate or place on ice immediately<br>-Separation of plasma/storage within 2 hr (preferably 30 min)	Below -20°C	Bulk transport (dry ice)
**Blood Drug monitoring**	- Refrigerate or place on ice immediately –<br>-Separation of plasma/storage within 2 hr (preferably 30 min)	Below -20°C	Bulk transport (dry ice)
**Blood Host DNA**	- Refrigerate[1] immediately<br>- Aliquot/storage on the same day (within 6 hr)	-80°C preferred	Bulk transport (dry ice)

[1] Refrigerate at 2-8 ℃
[2] RT: 18-25 ℃

- ***SERUM PROCESSING***

Reagents and Hardware

- 2 x 4 mL Red top (BD, cat.# 369032 or similar) no gel
- Centrifuge (Eppendorf)
- 2 mL screwtop cryo tubes
- Barcoded labels
- P1000 pipette
- Filter tips
- Storage boxes

METHOD

1) This procedure is routinely performed in a BSL-2 hood in the laboratory. If a BSL-2 hood is not available, this procedure can be performed on a benchtop, provided that appropriate PPE is used (gloves, lab coat, and safety goggles).
2) Blood samples for serum collection are collected in a RED top vacutainer tube. Two 4-5 mL vacutainers are collected per study participant.
3) Hold the vacutainers at room temperature (18 - 25ºC), for ~ 30 minutes but no more than 2 hours. Freezing with in 4 hours is required.
4) Centrifuge vacutainer tubes for 10-15 min at 1200-1500 x g. Determine these settings for your centrifuge and record here
   1200-1500 x g - ____________rpm
5) Prepare 5 to 6 2 mL sterile cyro tubes with the barcodeded labels with subject ID and visit ID.
6) Pipette off appropriate volume of serum avoiding the red blood cell pellet and dispense into labelled micro tubes. Generally, ~500 µl is aliquoted per tube.
7) If there is insufficient serum to fill all tubes, prepare as many tubes as possible at 0.5 mL with the remainder in the final tube. Record the volume on the side of the last tube for capture into the database. If extra serum is available after aliquoting, add the extra to the last tube up to a maximum of 2ml.

- ***PAXgene tubes for human mRNA***

Transport to the Lab

Store the PAXgene tube upright at 18 to 25 C or at 4 C during transport to the laboratory from the site.

Receipt of PAXGENE RNA Blood Collection Tubes

1) After collection of the blood sample, it is important to hold the PAXGene RNA Blood collection tube for at least 2 hours at room temperature (15–25°C). Incubation of the PAXGene RNA Blood collection tube overnight may increase RNA yields in some cases.
2) The PAXGene Blood RNA tubes should be frozen standing upright at –20°C overnight in a wire rack, NOT in a polystyrene foam tray.
3) The tubes may stay at -20 to -80 C as convenient, but should be held in freezer storage boxes once frozen to protect the tubes from impact and cracking until transfer to the core lab
4) Transfer the PAXGene tubes frozen packed safely in freezer boxes on dry ice to the core for long term storage at –80ºC
5) Currently data suggest viability of RNA following storage of PAX RNA Tubes at –20°C and –80°C for up to 50 months. Long term archiving studies are ongoing.

- ***Host DNA***

  All sites will collect whole blood for host DNA at the Day 0 visit only.
  - At the day 0 visit (baseline) only draw whole blood using a 2 to 4 mL EDTA vacutainer tube.
  - Shake the tube to mix in the EDTA and hold at 4 -8 C or on wet ice until the blood can be processed for freezing. Blood should be frozen on the same work day.
  - Label two 2 mL cryo tubes per blood sample with preprinted labels reflecting the subject ID, and sample type,
  - Wearing protective clothing and gloves, divide the blood into the two cryo tubes (~1 mL each).
  - Store at -20 to -80°C for short term storage until the samples can be transferred to the core lab for long term storage at -80 C.

- ***Plasma for drug PK (Week 20 only)***
  - For processing ETDA blood into plasma, see section 9.5.3

9.6 PK SUBSTUDY BLOOD COLLECTION AND HANDLING

9.6.1 Notes on Timing of blood collection and TB drug administration

- Participants must arrive for the study visit without having taken their TB drug dose for that day or the study must be delayed to another day.
- Windows for the blood draws are +/- 10 min for the 1 and 2 hour draws and +/- 20 min for the 6 hour draw.
- It is best to process blood into plasma within 2 hours but preferably by 30 minutes, so the processing will have to take place at the collection site during the time when the samples are being collected.
- Normal procedures and precautions for drawing blood should be followed

9.6.2 **Procedure for timing of blood collection and TB drug administration**

- Record the time TB medication was taken (by patient report or check the pill box opening times) for the two previous days
- Using a Lavender top [EDTA] tube, collect 1-2 mL of blood, record the time
- Have the subject take their INH/RIF dose and record the time
- Place the tube on wet ice, in cooler box, or refrigerate (2-8°C) immediately.
- Process or pass on the blood to the site lab for processing
- Draw 3 additional blood samples (approximately 1-2 mL each) into Lavender top tubes at approximately 1, 2, and 6 hours post-dose, recording the time and holding the tubes on ice or refrigerate (2-8°C) immediately.

9.6.3 **Processing blood into plasma**

- The blood should be kept cold (on wet ice or in the refrigerator) until centrifugation
- Prepare two 1.6 – 2 mL storage tubes per blood sample with preprinted labels reflecting the subject ID, PK sample type, sample time point, and date and time collected
- The blood can be centrifuged in the collection tube or transferred to 2 labeled microcentrifuge tubes depending on the centrifuge available. Cells are removed by centrifugation for 10 -15 minutes at 1,200 -1,500 xg.
- Determine these settings for your centrifuge and record here
  1200-1500 x g - ____________rpm
- Using a plastic Pasteur pipette or a pipettor and clean tips, transfer the clear fluid from the tube (avoiding disturbing or transferring the red pellet) aliquoting about 0.2 to 0.5 mL into the 2 labeled storage tubes.
- Store the plasma cold (2-8°C) for no more than 4 hours until it can be frozen at -20°C or below.
- transfer to the samples frozen on dry ice to the long term storage site, where they will be stored at -80 C.

9.6.4 **Storage and Specimen Shipping**

- The plasma samples will be transferred to the core lab at the Henan Provincial CDC for storage until shipping for analysis.
- Transfer the samples to the analysis lab without thawing and maintain frozen until the samples will be analyzed by liquid chromatography mass spectrometry.
- The samples will be analyzed in the Shanghai Public Health Clinical Center (Lab of Lijun Zhang) using well established methods in batches of 30 or more subjects or more per group.
- Dr. Zhang is the contact for the shipping company and documents necessary for shipping the samples on dry ice overnight to her lab.

9.6.5 **Reporting of results**

Results of the analysis- concentrations INH and RIF in the blood samples- will be report in CSV files that can be imported into datafax.

9.7 URINE

9.7.1 **URINE COLLECTION PROCEDURES**

Urine collection provides samples for pregnancy testing and aliquots for biomarker assay groups. Clean catch is not essential for assay groups, but preferred.

- Provide participant with ID-and visit labeled urine container.
- Explain the clean-catch method:
  Participant goes to clinic cloakroom/toilet. When in the stall, participant cleans the area of the urethral opening (where urine passes) with paper towel(s) and water. Female participants are to wipe from front to back at least twice with two separate wet towels. Subsequently, participant starts to pass urine, stops midway, and then catches the "mid stream sample" in the container supplied (aim for 5 to 10 mL). Midstream collection is not absolutely necessary, but encouraged. participant must replace lid of container and take it back to the study staff.
- Ask participant to void at a convenient point during the visit.
- Store all the labeled sample in a refrigerator or in a laboratory cooler at 2-8°C until processing for freezing (within the work day or a maximum of 6 hours).
- Pregnancy testing should follow the test's instructions or clinical SOP.

9.7.2 Urine Storage for Biomarkers

- Label 4 freezer storage vials (1.6 to 2 mL) with laboratory label containing Subject ID, specimen type and visit ID (in china these will be provided by the core lab)
- Wearing gloves and a lab coat, arrange the specimen, sterile transfer pipettes and the storage vials in a biological safety cabinet
- open the urine container in the cabinet, pipet 1- 2 mL of urine into each vial and close the caps tightly
- Record the number of vials prepared onto the laboratory log
- Store the vials upright in a rack or storage box in -20 to -80°C freezer if not immediately transfer to freezer farm or core lab
- Using the laboratory log, enter the storage information onto the storage CRF
- Transfer to other lab sites must be on dry ice to prevent the samples from thawing before analysis.
- Analysis methods will not be addressed in the MOP

9.8 Shipping of Samples

The site lab should contact the core lab contact for shipping to notify them of the intention to transfer frozen samples at least 3 days in advance so that a dry-ice containing shipper will be available for the transfer.

1. Transportation requirements: by no less than 2 person special car escort, and the transport personnel must hold the Provincial Health Planning Commission issued laboratory biological safety training certificate, and take corresponding biological safety protection measures.

2. Sample transport external package requirements: containers of transport samples should be three layers of packaging system. The first layer is used for placing samples, and will be putting samples in a waterproof, leak proof biological bag, and wrapping is enough absorbent material to absorb rapidly when leaks occur. The second layer should be firm, waterproof, anti leakage for the protection of the first layer. This layer can be cans, plastic cans, plastic bags or plastic foam, and the layer can accommodate a couple of specimens. Between the one and two layers, to fill the absorbent material sufficient, and a detailed list of specimens will be placed between the second and the third layer. In order to reduce the temperature

inside of the package, some of dry or wet ice should be placed in between the second and third layers. If the use of ice, the packaging must be waterproof, if the use of dry ice, the packaging must be able to let the emission of carbon dioxide gas, so that can prevent the pressure from increasing and caused the container rupture. The third layer is used to protect the inner packing. It can be made of cardboard boxes, wooden cases, solid plastic boxes, etc.. The name, delivery place and time of the carrier and the receiver's name will be marked on the out site of the packager box, and "infectious substances", indicating the degree of biological damage are labeled also.

3. Shipping records requirements: each specimen transportation must fill in the laboratory application form, enclose copies of the specimen collection source document and the shipping log.

4. Samples packaging and records: on-site personnel need to complete the sample labeling and packaging, ready to copy the samples source files, and completed the patient and sample information in the shipping log for the shipping samples before carrier arrived site for a single.

5. Sample inspection: when the carrier receives the sample at the site, the sample type and quantity shall be checked according to the record of the transport log. Meanwhile, should make sure the specimen cap has been tightened. If not, should wear N95 masks and disposable gloves, and transfer the specimen bags and containers to the biological safety cabinet, maintain ventilation function, open the seal of the specimen bag, the container cover is covered, then tighten, reseal specimen bag. The sample is loaded into the sample container as required. The purpose is to ensure that all the samples are correct and the transportation is safe.

6. Complete log: transport log will be completed by three people, including site researchers, transport personnel and the central laboratory samples receiving personnel, and they will be signed, indicating the transfer time. See the schedule sample transport log for details. A signed form will eventually be returned to the research site and a copy will be kept at central laboratory.

LOGS AND DOCUMENTS WE NEED IN CONJUNCTION WITH THIS MOP
MERM Education Sheet
MERM Set up Work Instruction
Screening and Enrollment Log
Delegation of Authority and Site Signature Log
Site Visit Log
Participant Visit Log
Shipping Log
Communication Plans—Data management, Clinical, and Lab
Drug Adherence Card/Pill counting log
Pregnancy injection monitoring log
Smear and Culture SOP
Freezer Logs (or this will be FreezerPro?)

Form 11-1 Laboratory Request Form (South Africa)
Form 11-2 Transport Log (South Africa)

SOP 11-1 Transport (South Africa)
SOP 11-2 Laboratory SOPs: smear, culture, DST
