# Supplementary material for: Using biomarkers to predict TB treatment duration (Predict TB): a prospective, randomized, noninferiority, treatment shortening clinical trial
Source: Gates Open Res. 2017 Nov 6;1:9. [Version 1] doi: 10.12688/gatesopenres.12750.1 (PMC5841574; doi:10.12688/gatesopenres.12750.1)
Supplement: Supplementary file 5 [file gatesopenres-1-13810-s0004.tgz › eba77868-e1aa-4a8d-9cf8-754f1ed0c93b.pdf]

SOP for
Predict TB Trial PET/CT
Reading Methodology

Ray Chen

Version 1.2

8-September-2017

Download PET/CT Scans

- If you have Aspera Drive set up on your computer, PET/CT scans randomly assigned to you will automatically download into that folder when you are connected to the internet.
  – On my computer, this is in the Aspera Drive folder Predict PET-CT scans > rchen > LCID-TRS_Predict_PET-CT-Scans_Chen
  – This is a one-way download folder. Items you place in this folder will not be uploaded back to Aspera.
- If you do not have Aspera Drive set up or it is not working, you can download the scans manually from the LCID-TRS_Predict_PET-CT-Scans folder in Aspera.

Not Available to Read

- If you will be on vacation or otherwise will not be available to read scans for a specific period of time, the reader randomization algorithm will be adjusted to exclude you for those days. Please email the following people at least 7 days in advance:
  - Michael Duvenhage:
  - Kanwal Bajwa:
  - Lori Dodd:
  - Jing Wang:
  - Ray Chen:

Accessing MIM

- There is a single MIM license located on a cloud server available for the 2 China readers and 2 South African readers to share.
- Every time you log onto the MIM license, you have a 2 week/30 use grace period when you can continue to use MIM without touching the license.
- To allow 4 readers to share 1 license:
  - Only log onto MIM with your computer wifi connection on occasionally (every 1-2 weeks), then close MIM.
  - Turn off your wifi and then open MIM again to do any measuring work.
  - Keep your wifi off while you work in MIM.
  - This will allow 4 readers to share 1 license. If you work in MIM while connected to the license, others will not be able to access the license.
- Our MIM license server address is *xxxxx*. This is just FYI as this should already be pre-set in your MIM.

MIM Date Format

- Please set your date format to MM-DD-YYYY to allow data comparison:
  - Click on the settings gear at the very top of MIM
  - Select General Preferences
  - Select Application
  - Scroll to the bottom to “Date Format”
  - Select MM-DD-YYYY option
  - Apply the changes and restart MIM for this to take effect

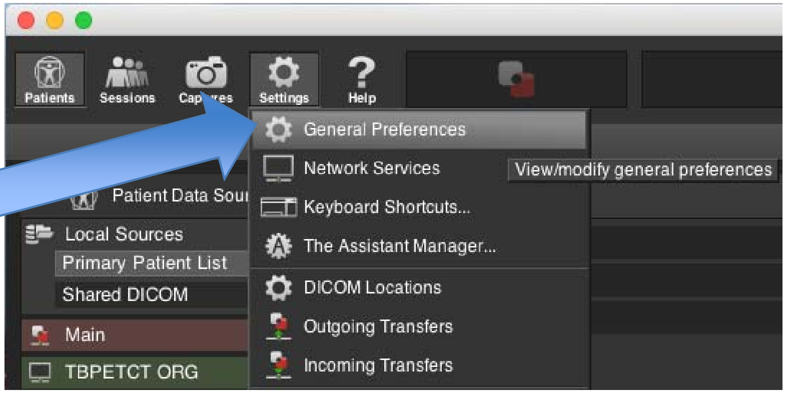

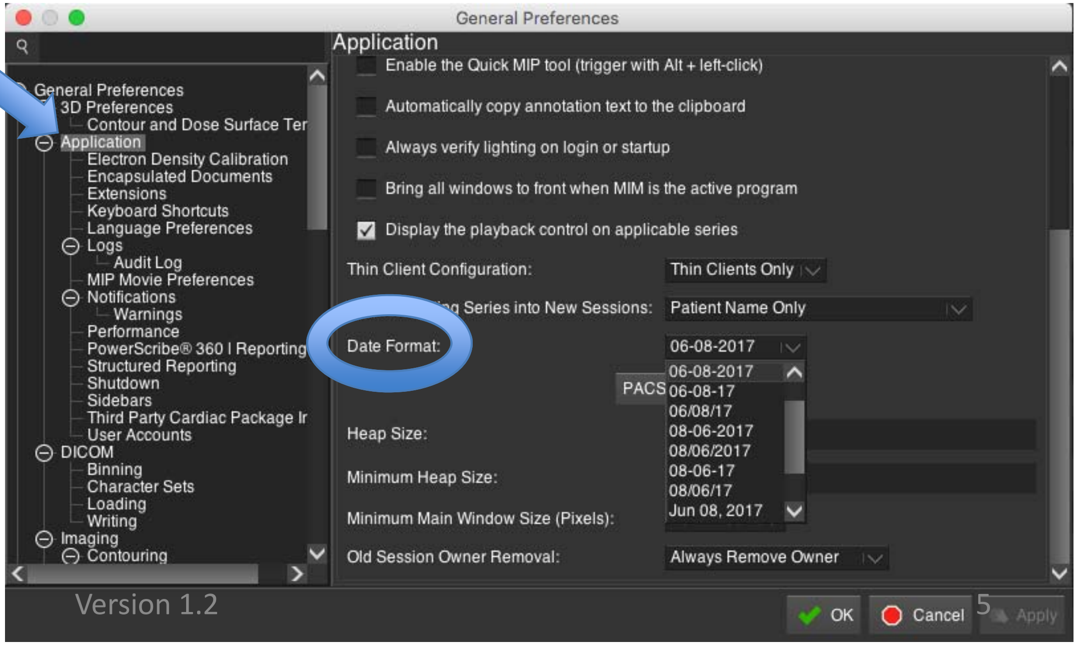

Reading Methodology

- Goal is to develop a Predict study reading methodology that:
  - Captures required information to stratify patients without being too laborious thus taking too much time
  - Is simple enough to be reproducible and consistent across readers for patients with both minimal and severe disease
- Required information:
  - Assessment of:
    - Pleural thickening to rule out pleural (extrapulmonary) TB
    - Total lung collapse of a single side
    - Pleural effusion
  - Cavity air with baseline to week 4 comparison by cavity to determine % change
  - Total hard volume with baseline to week 4 comparison
  - Total lesion glycolysis (TLG) with baseline to week 4 comparison

Early Treatment Completion Criteria

Early Completion criteria:	Determined at Week 16 – unless known to have failed a radiographic criterion at baseline or week 4.
Radiographic criteria	Baseline PET/CT:<br>• No total lung collapse of a single side, AND<br>• No pleural effusion, AND<br>• No single cavity air volume on CT scan >30 mL, AND<br>• CT scan hard volume (-100 to +100 HU density) <200 mL, AND<br>• PET total activity <1500 units<br>Week 4 PET/CT:<br>• All individual cavities decrease by >20% (unless cavity <2 mL), AND<br>• CT scan hard volume does not increase by >10% unless the increase is <5 mL, AND<br>• PET total activity does not increase by >30% unless the increase is <50 units
Bacterial load criterion	Week 16 Xpert cycle threshold ≥30*
Adherence criterion	Minimum of 100 doses received by week 16

Measurement Elements

- Pleural thickening
- Total lung collapse
- Pleural effusion
- Cavity air
- Hard volume
- Total lesion glycolysis (TLG)

Baseline Scan

Open Baseline PET/CT Scan

- Load and open the baseline PET/CT scan. Be sure to select the “Lungs” CT scan as it has the best resolution and the WB_CTAC PET scan.
- In the “Display” tab on the left side of the screen, create a new window with just the fused PET/CT scan and the CT scan alone.
- It is easier to view cavities on the CT scan without the PET activity. If the PET activity scale is set too high for that scan, air pockets on the CT scan may be washed out.
- Adjust the size of the scans and change the CT to lung window view.

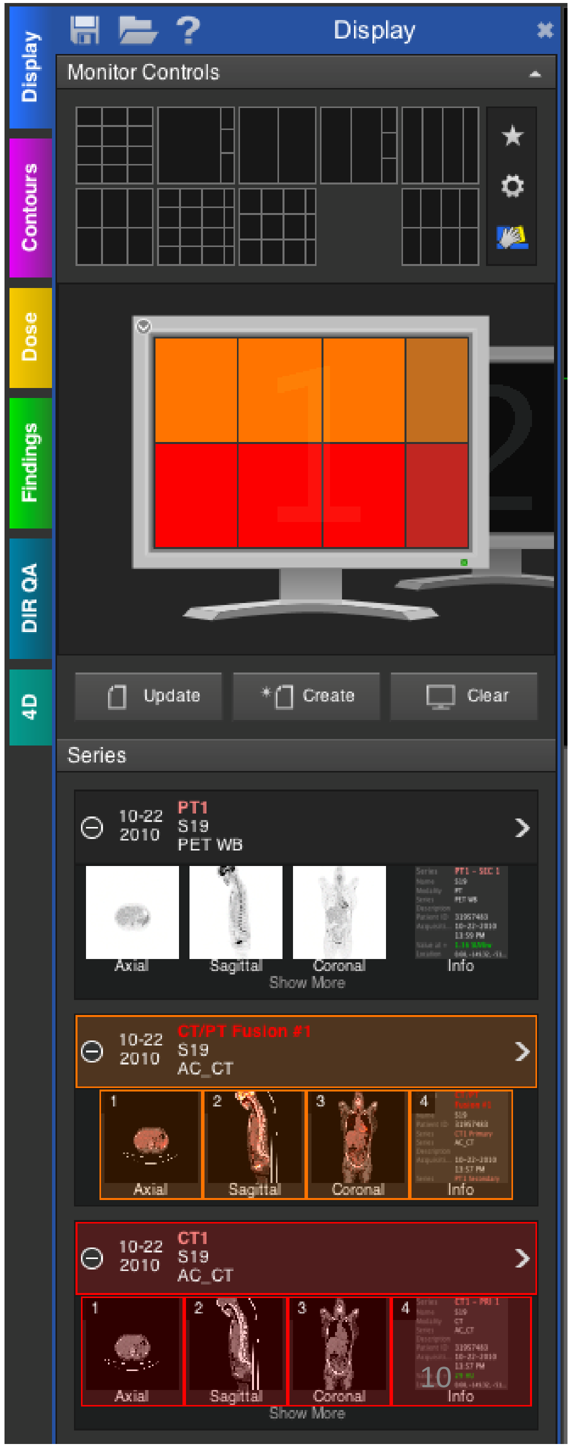

Pleural Thickening

- Look for obviously thickened, diffusely inflamed pleura.
- Do not count if pleural thickening is very focal or limited.
- Because extrapulmonary TB is an exclusion criterion, patients like this should be withdrawn and replaced.

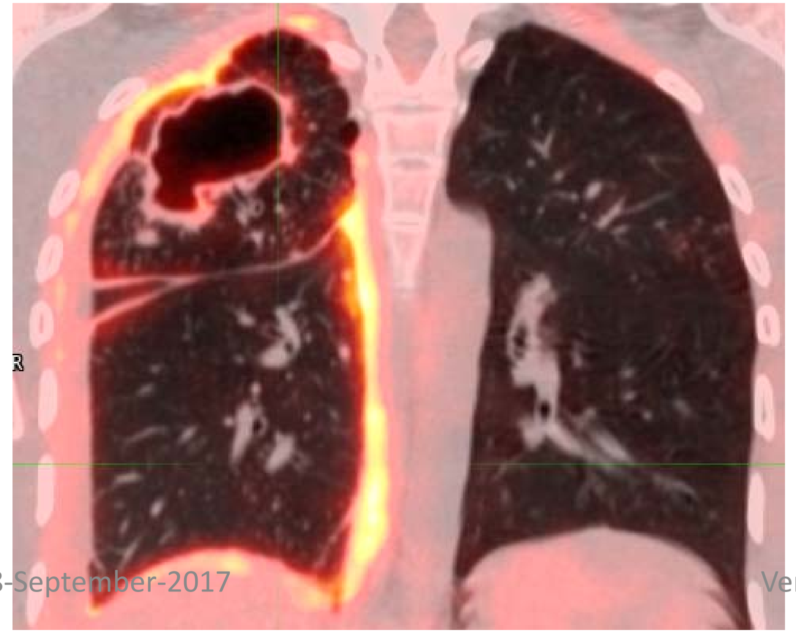

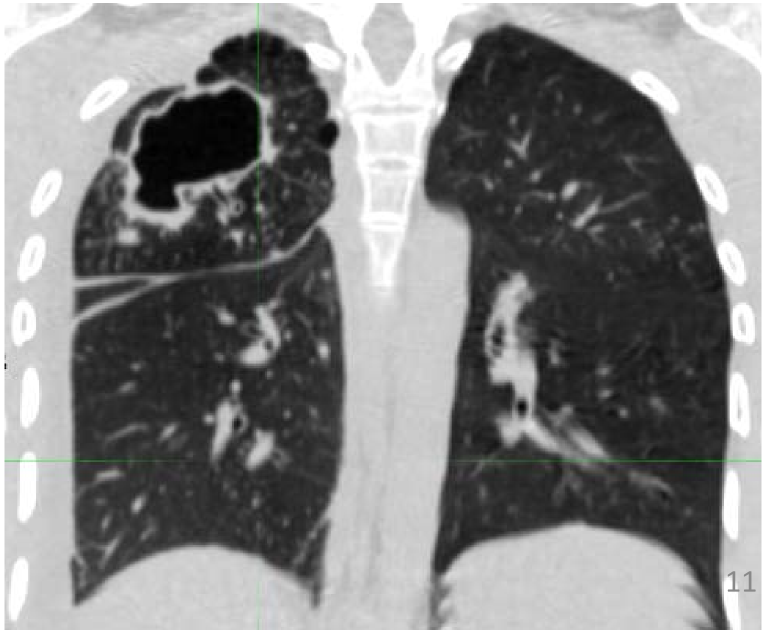

Total Lung Collapse

- Does the participant have a total lung collapse on the left or right side?
- This participant has a large cavity in the left upper lobe with total collapse of the rest of the left lung.
- Although this participant will be moved to Arm A, **all other measurements should still be completed** for future analyses.
- The reader does not need to track Arm A requirements. The system will automatically move participants who meet those requirements.

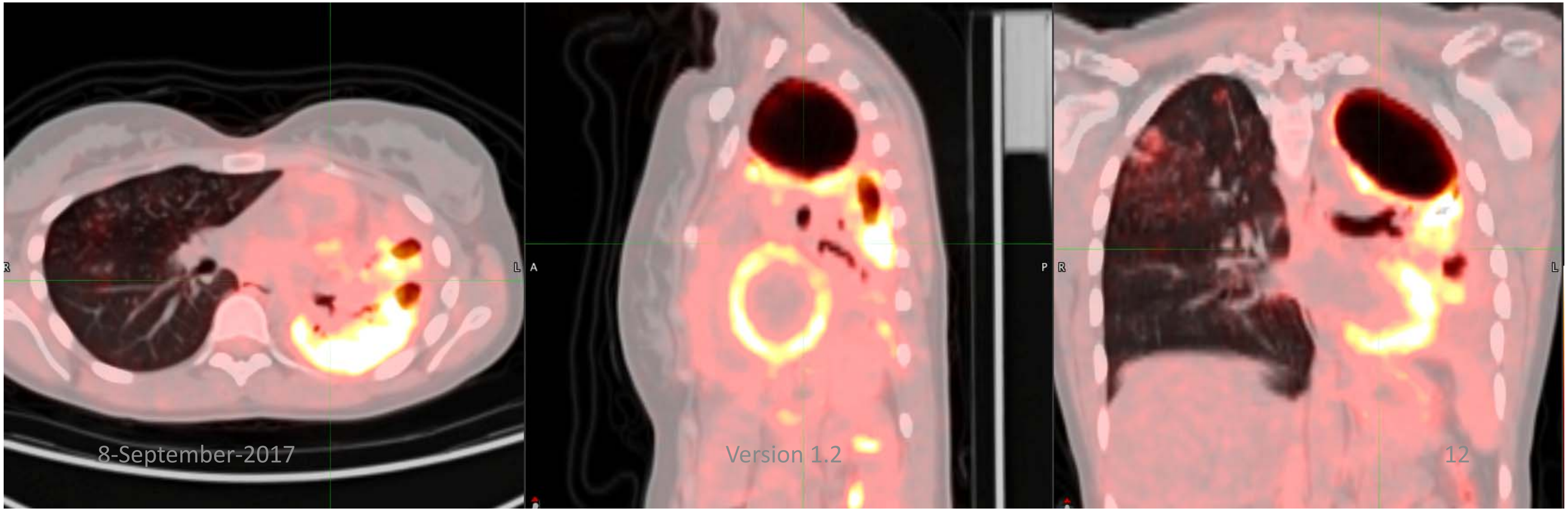

Pleural Effusion

- Participants with large pleural effusions on baseline PET/CT scan should be withdrawn and replaced.
  - Large pleural effusion is defined as an effusion that exceeds the first anteroposterior (AP) quartile (25%) of the hemithorax or measures ≥3 cm [illegible]n the axial view just superior to the hemidiaphragm.

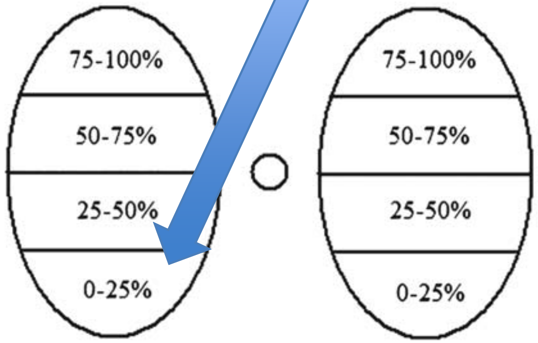

FIGURE 1. Line drawing of the assessment of the anteroposterior (AP) quartile.

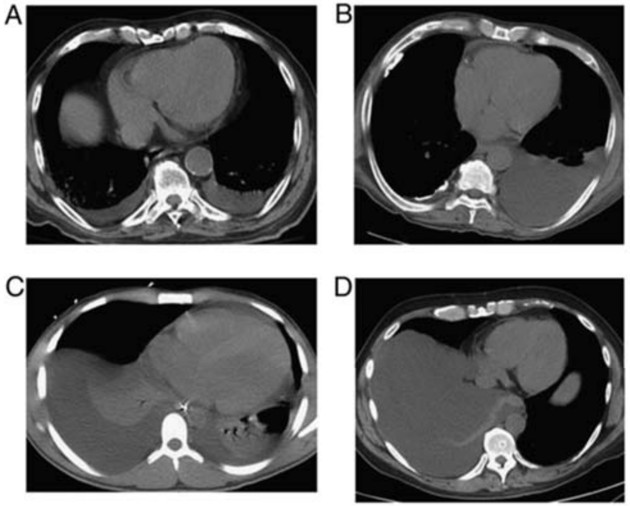

FIGURE 2. CT images of the thorax. A, A left effusion measuring 17% of the hemithorax and reaching the first AP quartile (0%-25%). B, A left effusion measuring 38% of the hemithorax and reaching the second AP quartile (25%-50%). C, A right effusion measuring 40% of the hemithorax and reaching the third AP quartile (50%-75%).D, A right effusion measuring 82% of the hemithorax and reaching the fourth AP quartile (75%-100%). See Figure 1 legend for expansion of abbreviation.

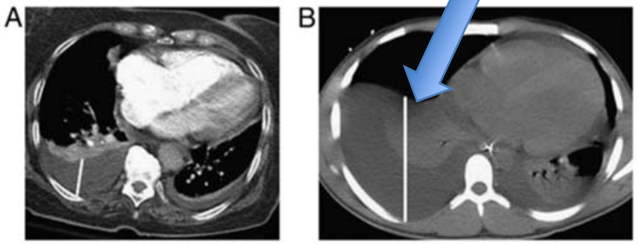

FIGURE 3. CT images of the thorax. A, The measurement of AP depth does not include atelectatic lung that is anterior to the effusion. Note that the measurement is not perpendicular to the plane of the figure because the patient is slightly rotated. B, The measurement does include atelectatic lung that is completely surrounded by pleural fluid. See Figure 1 legend for expansion of abbreviation.

Table 3—*Criteria for Three-Point Prediction Rule*

	Criterion	
Effusion Size	AP Quartile	AP Depth
Small	0%-25%	<3.0 cm
Moderate	25%-50%	3.0-10.0 cm
Large	50%-75% or 75%-100%	>10.0 cm

Moy MP, et al. *Chest* 2013;143(4):1054-1059.

Pleural Effusion

- This participant has a loculated right pleural effusion. Because it is not free-flowing, effusion must be measured. Use the tape measure icon from the top bar to measure.
- Measurements are around 3 cm so a free-flowing effusion would measure >3 cm and would therefore be defined by our study as "large".

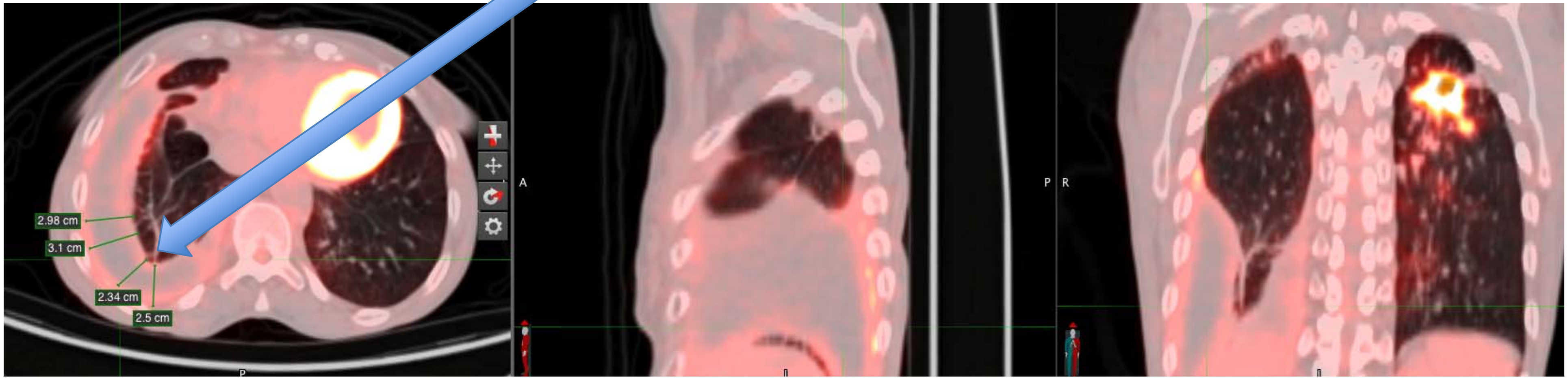

Tape measure icon

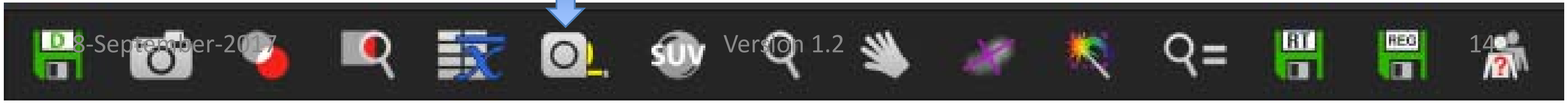

Pleural Effusion

- If the participant is determined to have a "large" pleural effusion (>25% of hemithorax or ≥3 cm), s/he should be withdrawn from the study and replaced.
- Note that estimating the proportion of the hemithorax taken up by the effusion is relatively qualitative and so should be estimated conservatively. If the proportion is close to 25%, the participant should be excluded.
- If the participant has a pleural effusion but does not meet the definition of "large," s/he should be included in the study. This participant will be moved to Arm A but **all other measurements should still be completed** for future analyses.
- Note that the pleural effusion should be obvious on CT to be moved to Arm A. If there is just a trace amount or if you are unsure if there is any, then the pleural effusion box in DataFax should be marked "no".

Excel Worksheet

- Complete the results for these 3 data elements (pleural thickening, collapse, pleural effusion) into the provided excel table.
- This is a continuous table which records image review results for all of your participants.
- The results for the quantitative elements that follow (cavity volume, hard volume, and TGA) should also be recorded on this worksheet.
- Table will automatically sum left and right lung hard volumes and TLG.
- Please use this worksheet to input the captured data into DataFax.
- Note that column heading names are also the standardized ROI names that should be used in MIM for the .csv file outputs.
- This excel file is located in Aspera in the following folder: LCID-TRS_Predict → PET-CT reader SOP → PredictTBReaderResults 2017-05-30.xlsx

Cavity Air

- It is easier to see cavities on the CT only scan because small cavities can sometimes be over-shadowed by bright PET activity so use the CT scan to measure cavity air.
- Unlike hard volume and TLG, a new ROI needs to be created for each cavity air.
- Click the green “plus” sign to create new region of interest (ROI), then select the “Region Grow” contour tool. If you don’t first create a new contour, the cavity air contour you create may be added to the last ROI you worked with.
- Select your starting seed point to grow your region by clicking in the cavity. Two green lines will appear to mark that point.

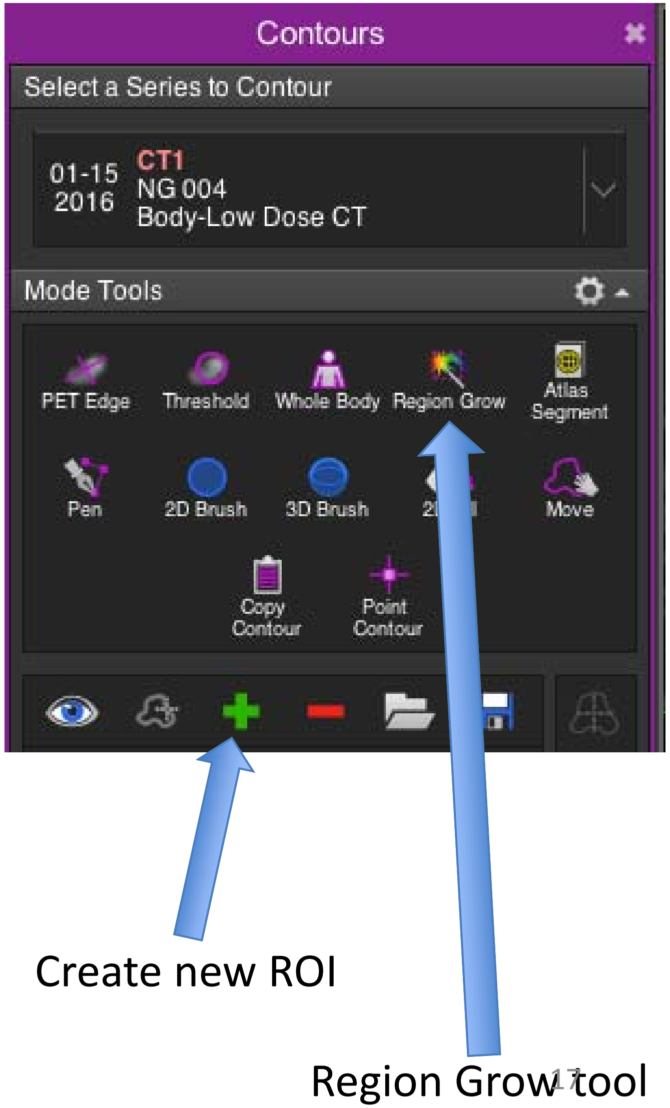

Cavity Air

- Click on the green flag on the right side of that view to start the region grow process.
- A boundary cube will appear shaded in the color of your new ROI and a new tool bar appears on the right side. Click on the wheel to open the region grow dialogue box.
- For large cavities, ensure that the entire cavity is included within the region grow cube.
- Input the following:
  - Upper threshold = -750
  - Lower threshold = -1024
  - Tendril diameter = 0
  - Fill holes = none
  - Click “OK”
- Click on the flag and select “Done” to complete the Region Grow.

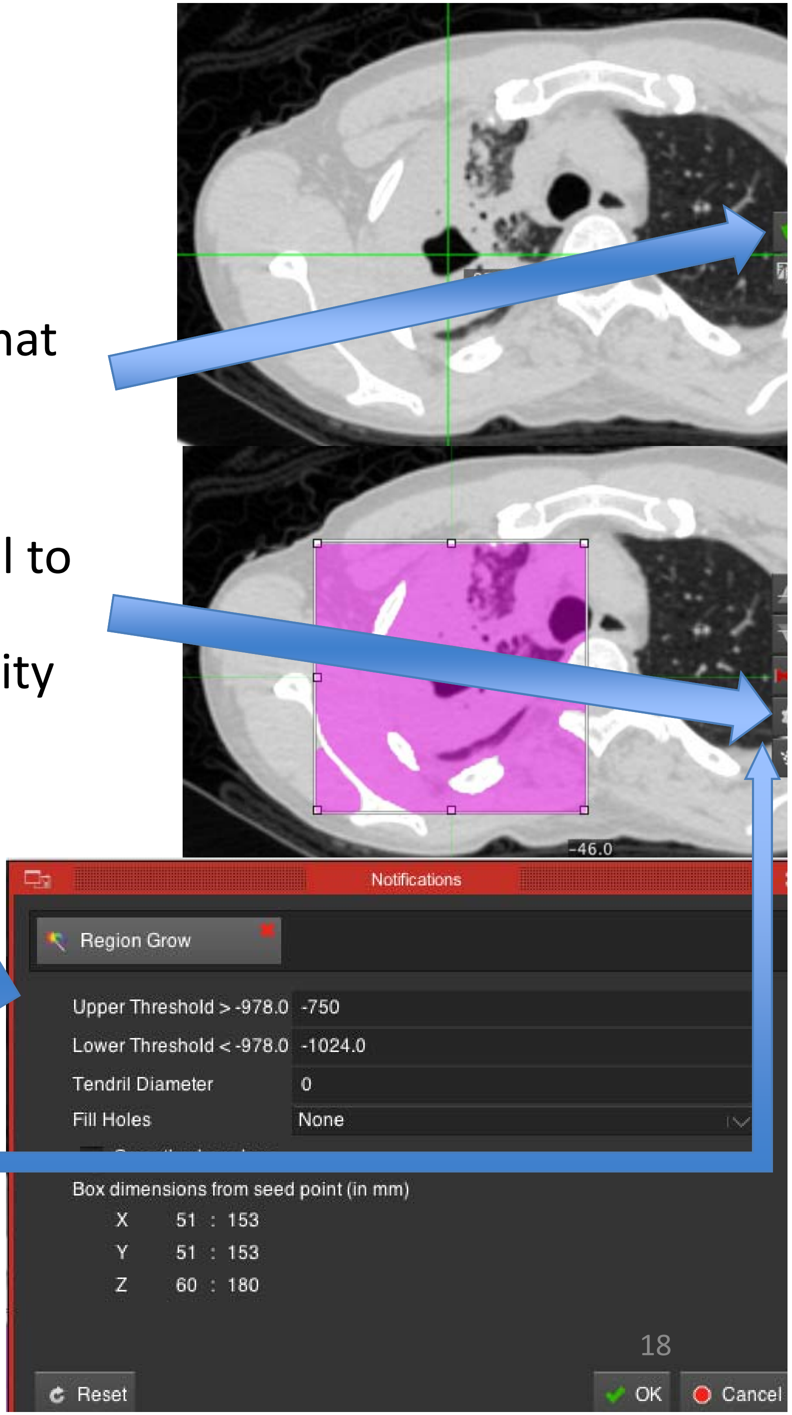

Cavity Air

- Sometimes (and especially after starting treatment) the Region Grow ROI will "spill out" of the cavity and into the surrounding lung. If this happens, you will need to adjust the tendril diameter.
- Tendril diameter is the length of a rod that can travel around the seed point to define the border of your ROI.
- A thick walled (untreated) cavity will contain this rod no matter how small (tendril=0) and your ROI will be defined by the rim of the cavity.
- During treatment, cavity walls break down and small holes appear. A small tendril diameter will allow your ROI to "escape" from the cavity.
- If your ROI extends outside of your cavity, increase the tendril diameter until the ROI remains contained within the cavity.

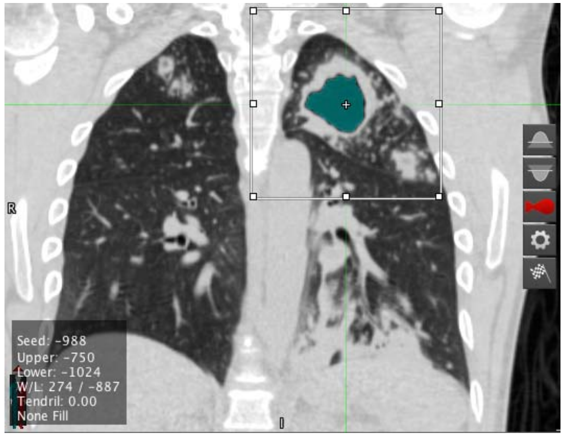

Baseline scan, tendril=0, with ROI contained within cavity

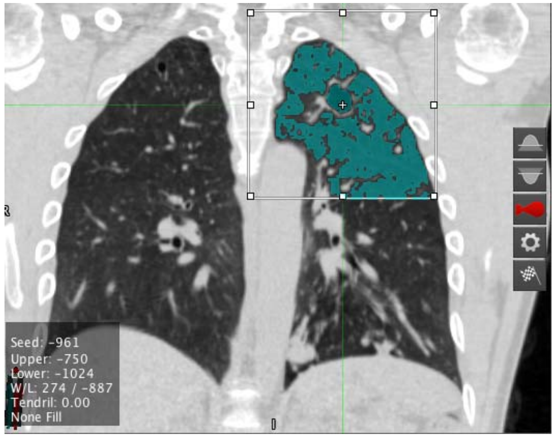

Month 6 scan, tendril=0, with ROI spilling out of cavity

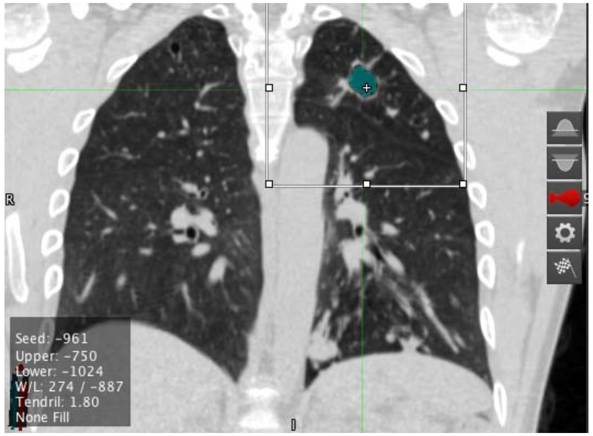

Month 6 scan, tendril=1.8, with ROI now contained within cavity

Cavity Air

- To change the tendril diameter:
  - Left click hold on the red tendril diameter icon.
  - Slowly move your mouse up (or down).
  - The tendril diameter can be seen in the lower left corner box and will slowly increase (or decrease).
- Slowly increase the tendril diameter until your ROI remains contained within the cavity.

Red icon to adjust tendril diameter

Box to see tendril diameter

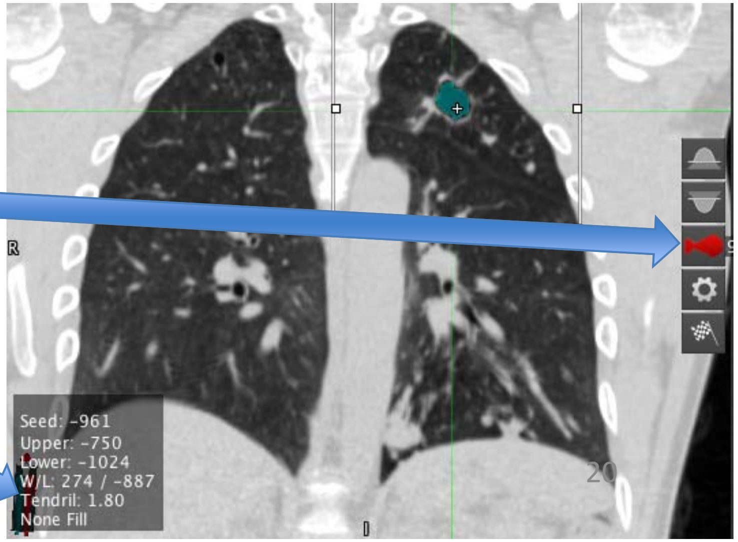

Cavity Air

- Once you click “done” in Region Grow, a new ROI will be created outlining the cavity.
- Rename this ROI “CavAir[# of cavity]_W[scan week #]”. If there are 3 cavities at baseline, these should be named CavAir1_W0, CavAir2_W0, and CavAir3_W0, etc. from largest to smallest.
- At week 4, these should be renamed CavAir1_W4, CavAir2_W4, and CavAir3_W4. At week 16 or 24, these would end with W16 or W24 (if still present).
- Cavities on the Week 4 and 16/24 scans should be named in the same order as on the baseline scan.
- **It is very important to use this specific naming convention for proper data transfer to DataFax.**
- Repeat the region grow process for each cavity you would like to measure, giving each ROI a unique name per the naming convention above.
- Start with the largest cavity and name 1 to 5 from largest to smallest.

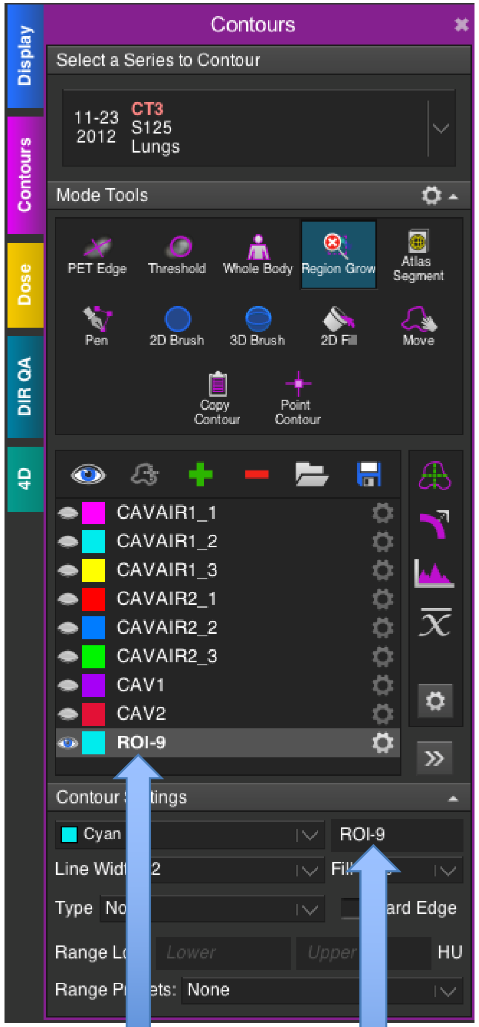

To rename contour, highlight name of desired contour, then type new name in box at bottom

Cavity Air

- Sometimes, patients will have several smaller cavities that are closely spaced. For this analysis, any cavity that is not captured within another cavity's Region Grow ROI should be considered a separate cavity and measured separately.
- Sometimes the cavity ROI created in Region Grow will extend into a connected airway. If this happens, erase the ROI from the airway, using the 3D Brush.
- Note that more than 5 cavities may need to be measured to determine the 5 largest cavities that are 2 mL or larger.
- **Name cavities from 1-5 in order of size on the baseline scan, from largest to smallest.** This is very important to allow comparison of cavity sizes between readers. Subsequent scans should maintain the same order as on the baseline scan.
- Cavities smaller than 2 mL do not need to be recorded but should be measured to confirm size. Give these a separate name for tracking purposes. **Do not use CavAir to name these small cavities.**

Cavity Air

- You can save the cavity air parameters (upper and lower thresholds, tendril diameter, no fill) to save time by selecting "save region template" after clicking the flag (instead of selecting "done").
- Name your new template (e.g. cavity air) and place into group "chest". Click "OK".
- The next time you use region grow, click "select region template" instead of the flag, then select "chest", then your new template name. Your cavity air parameters will automatically be applied.
- Click "Done".

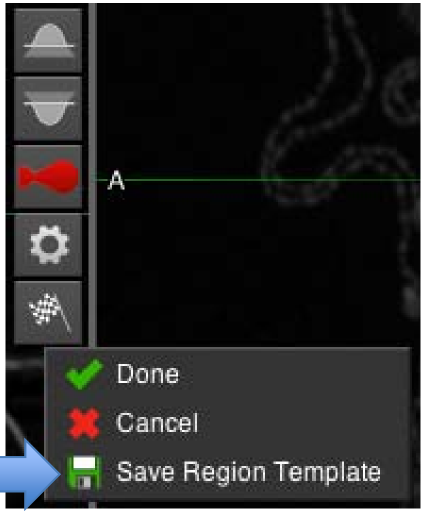

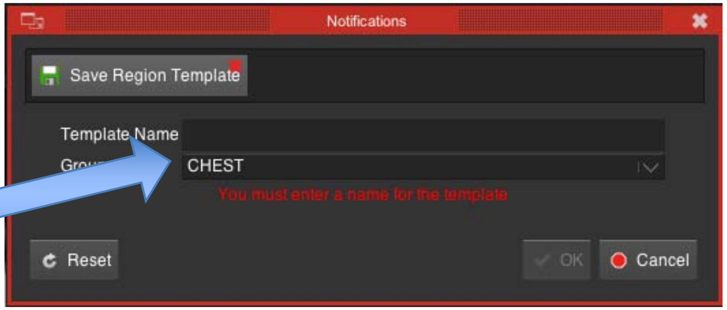

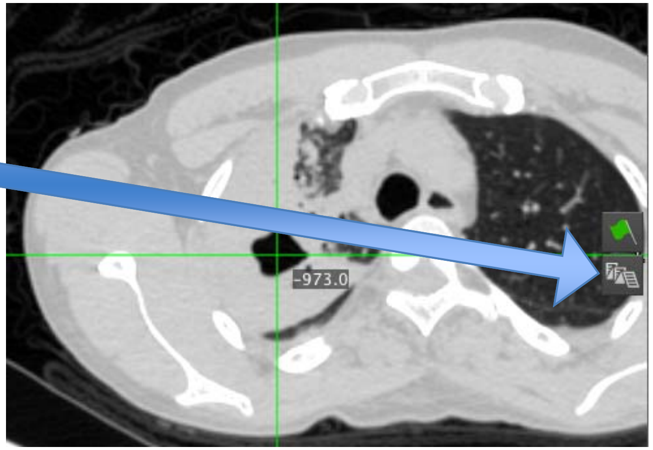

HU Hard Volume

- Draw two ROIs, one for each lung left and right, to include all areas of disease for each patient to capture hard volume and total lesion glycolysis (TLG).
- Use whichever contour tools you prefer.
  - Small, discrete, focal lesions may be more easily drawn with 3D Brush or PET Edge.
  - Extensive or diffuse lesions may be more easily drawn with 2D Brush and Interpolate Contour.
- The *Alt* (PC) or *option* (Mac) key allows you to draw on a different part of the lung with the same ROI.
- If you make a mistake, you can “undo” using the undo button at the top of the screen or Ctrl Z.
- **Be very careful** to exclude bones, intercostal spaces, mediastinum with large blood vessels, heart, and diaphragm from the ROI on all views (axial, sagittal, and coronal) as this will affect the results (see examples on next slide). Draw ROI conservatively even if this means you exclude some of the TB lesion.
- Using the Region Grow “lung mask” excludes dense lesions and includes too much of the border areas so is not recommended.
- PET hot lymph nodes located in the mediastinum should also be excluded.

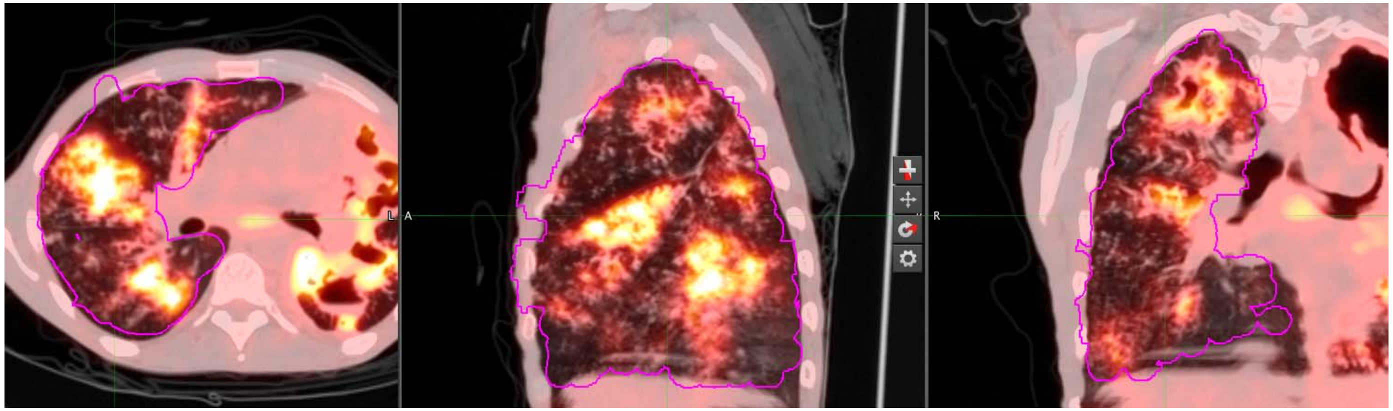

Hard vol: 207.9 mL

Example of total right lung ROI drawn with borders not cleaned up. ROI extends beyond lung borders to include ribs, mediastinum, and diaphragm. These all include areas of hard volume.

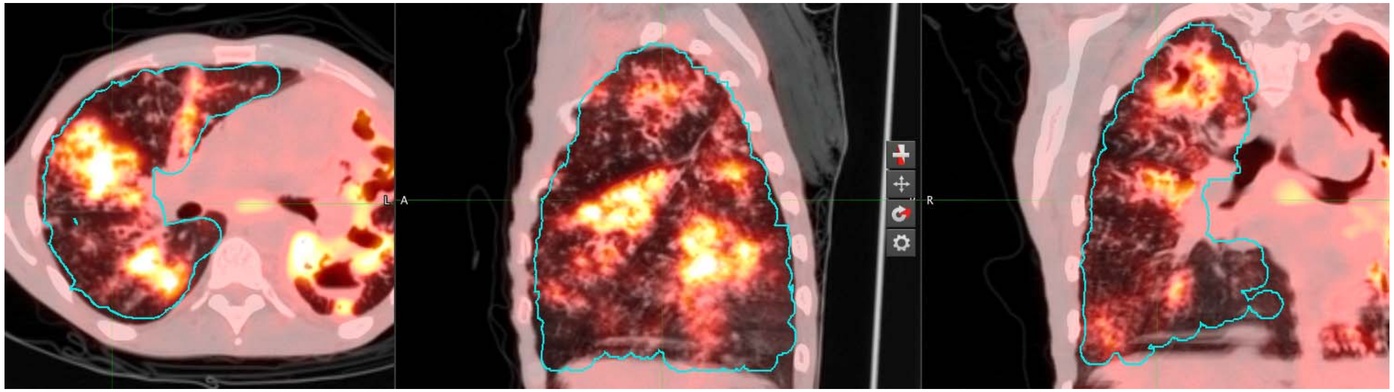

Excluding bone and intercostal spaces:
Hard vol: 159.1 mL

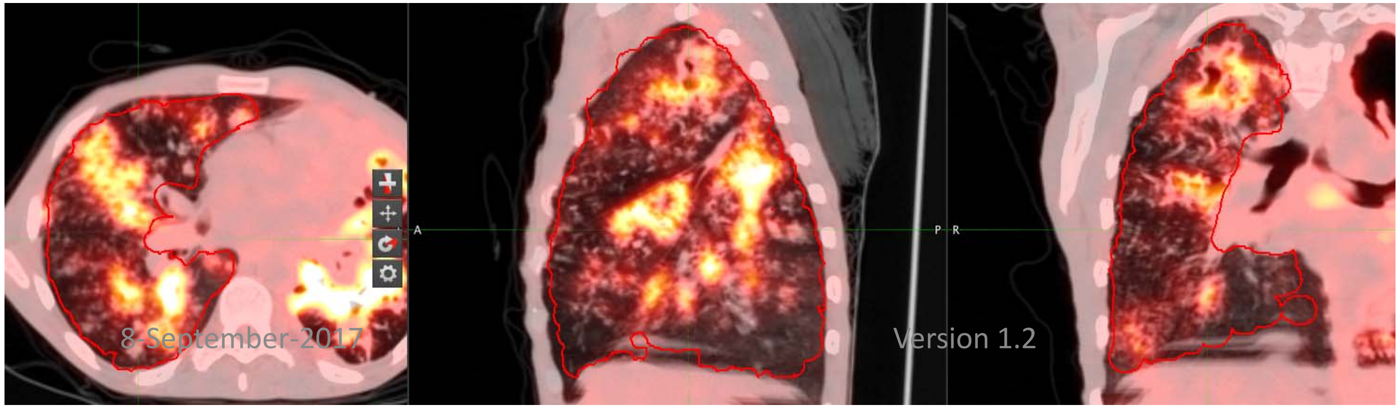

Excluding mediastinum, large vessels, diaphragm:
Hard vol: 127.3 mL

Drawing ROIs

- PET Edge: will automatically draw ROI for you but borders may require cleaning.
- 3D Brush: change size of brush using right click hold.
- 2D Brush with Interpolate Contour: change size of brush using right click hold.
  - Start drawing contour using axial view (original scanned view rather than reconstructed views).
  - Draw ROI, move 5-15 slices, then draw another ROI.
  - Click double arrow and select Interpolate Contour. MIM will automatically fill in ROI between the two contours.
  - Confirm borders on sagittal and coronal views.

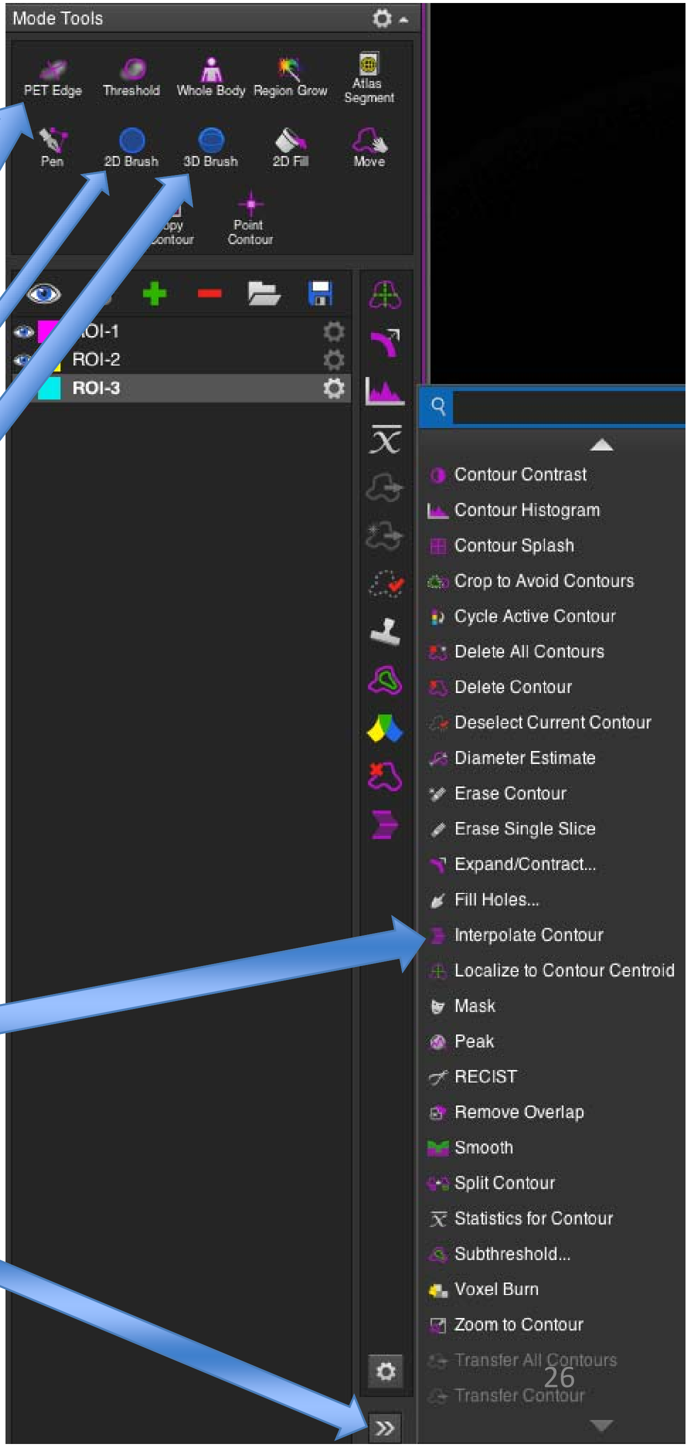

HU Hard Volume

- If there is a pleural effusion, exclude that from your ROI. Note that patients with large pleural effusions should already have been excluded.
- Note right pleural effusion that layers on sagittal view. Very small left pleural effusion too.

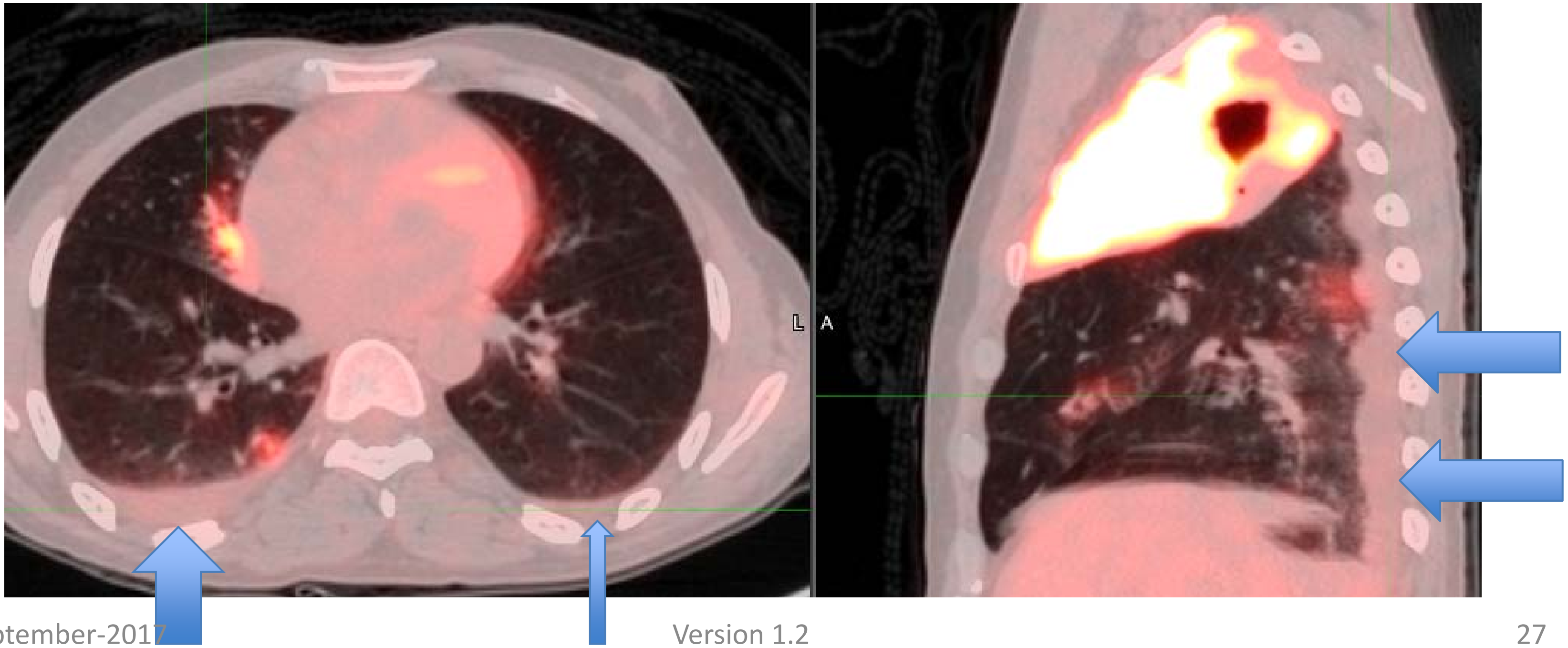

HU Hard Volume

- Normal lung has density primarily from -500 to -900 HU
- Dense TB lesions have HU primarily from -100 to +100 (hard density)
- Because there are almost no hard HU voxels in this range in normal lung, the size of the ROI drawn is not critical. A total lung ROI will give about the same hard volume as a lesion-focused ROI. (This is not true for PET SUV – explained later.)
- Once you finish drawing your ROI, rename as “RightLung_W0” and “LeftLung_W0”.
- “W0” becomes “W4”, “W16”, and “W24” at the later scan time points.

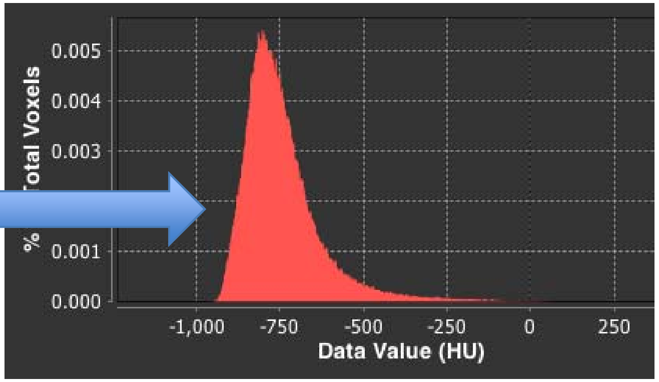

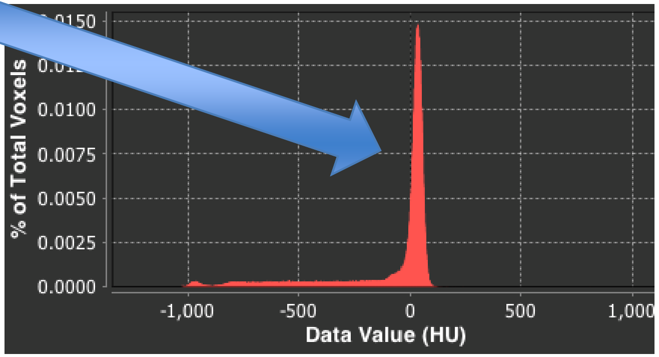

Total vol: 301.7 mL; Hard vol: 238.6 mL

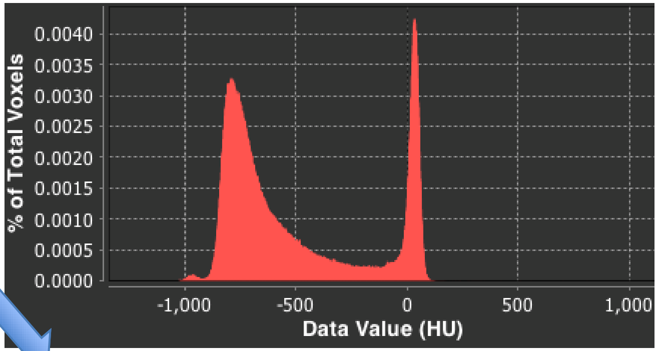

Total vol: 1025.7 mL; Hard vol: 244.4 mL

HU Hard Volume

- The easiest way to calculate hard volume requires more understanding of the ROIs drawn.
- In the 2 contour boxes to the right, the top is set to scan CT1 and the bottom to scan CT2.
- Contours are drawn on the scan showing in the contour box. When the selected scan is changed, ROIs are still shown but now are called “ghost” ROIs and have a “ghost” in the color square

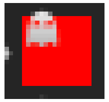

- Contours may be transferred to another scan using the “Transfer Contour” function by clicking the double arrows in the contour box. This will be required for some scans to use this alternative method, as described below.

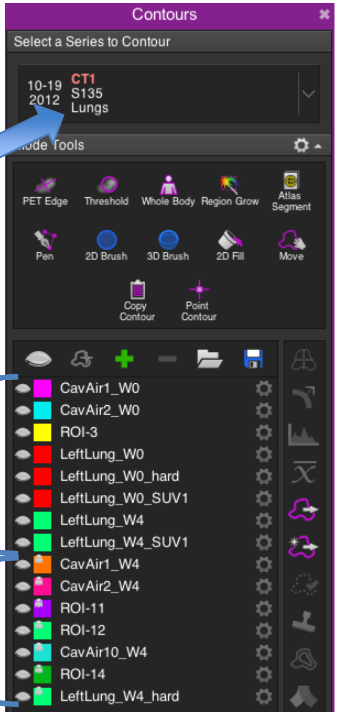

No ghosts because these ROIs drawn on CT1 scan

These ROIs are ghosts so were not drawn on CT1 (drawn on CT2, see below)

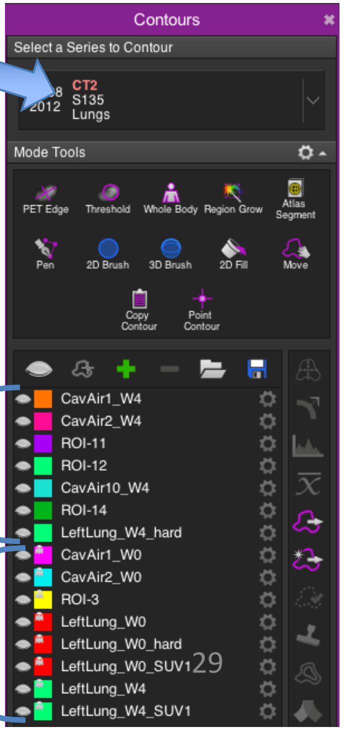

No ghosts because these ROIs drawn on CT2 scan

These ROIs are ghosts so were not drawn on CT2 (drawn on CT1, see above)

HU Hard Volume

- If you are reading the baseline scan and only have the baseline timepoint scan open, a hard volume ROI can be created using Range Lock:
  - Select LeftLung_W0 ROI
  - Select "Clone Contour"
  - New contour "LeftLung_W0(2)" is created.
  - Select new contour and rename "LeftLung_W0_hard"
  - In Range Lock, enter "-100" and "100" in the lower and upper fields; note unit is "HU"
  - Click "yes"
  - "LeftLung_W0_hard" contour now restricted to -100 to 100 HU
  - Repeat for right lung ROI
- Note Range Lock only works on contour drawn on selected scan. It does not work on ghost contours. In this example, ROI already drawn on CT1 scan so transferring contour not necessary.
- To use Range Lock for SUV1 requires transferring contour to the PET scan (described below).

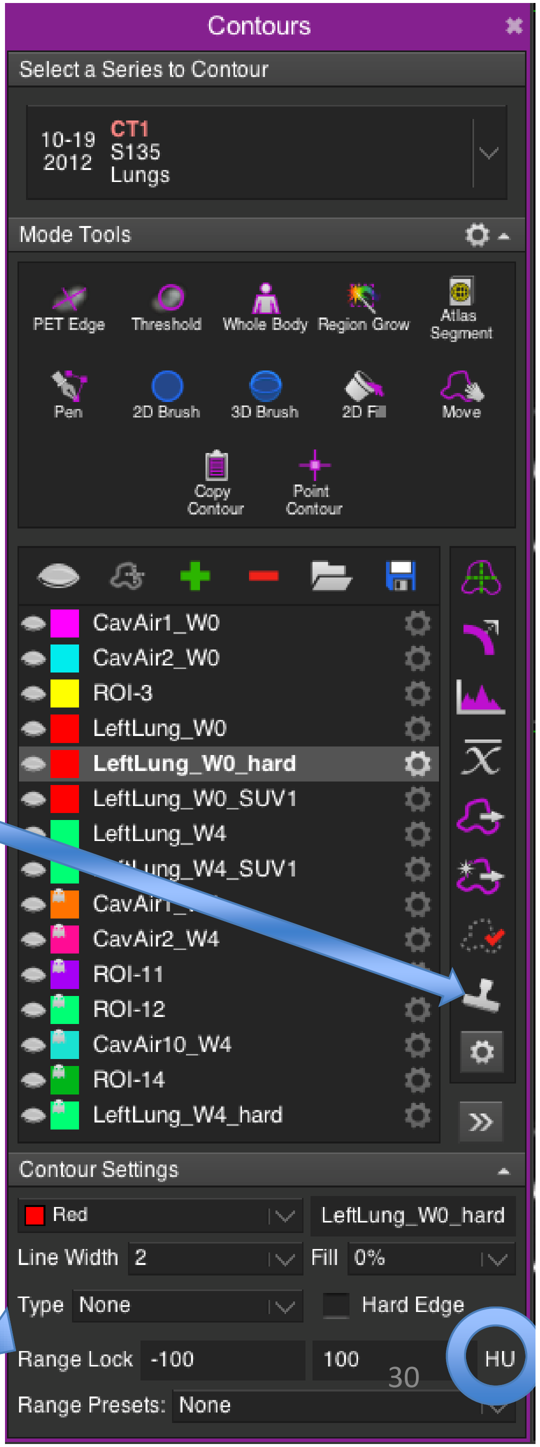

HU Hard Volume

- An alternative method to calculate hard volume but more complicated is to use Subthreshold.
- To Subthreshold:
  - Set scan at top of contour box to CT1
  - Select desired ROI to subthreshold
  - Click double arrow and select Subthreshold
  - Type “-100, 100” in “HU Threshold” dialogue box and click “OK”
  - 2 new ROIs will be generated, one with HU >-100 and another with HU >100

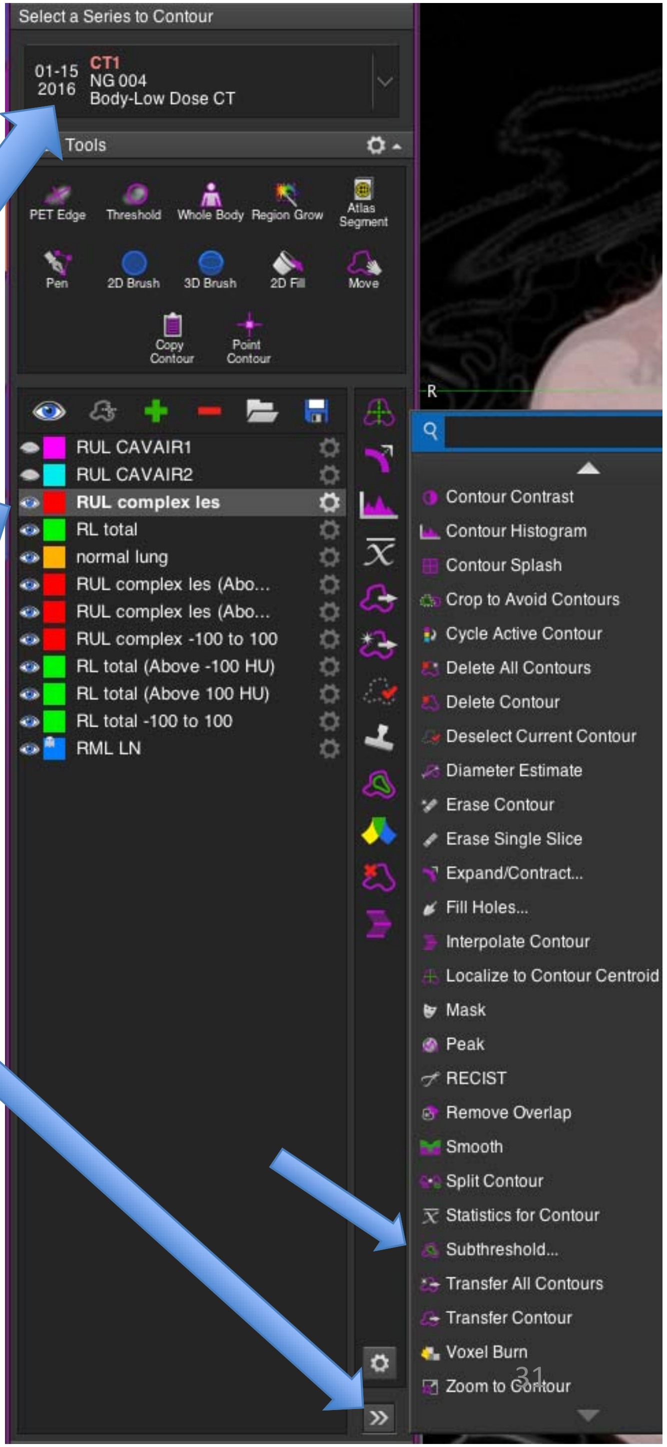

HU Hard Volume

- To calculate hard volume, click double arrow again and select Boolean Operations.
- Select the “above -100 HU” new ROI.
- Select the subtract button.
- Select the “above 100 HU” new ROI.
- Click the green check mark.
- A new ROI will be generated with just -100 to 100 HU volume.
- Change the ROI name in the Contour Settings box at the bottom of the contour panel to “RightLung_W0_Hard”.
- Delete the two subthresholded ROIs to decrease ROI clutter and confusion.
- Repeat subthreshold process for left lung ROI and rename “LeftLung_W0_Hard”.

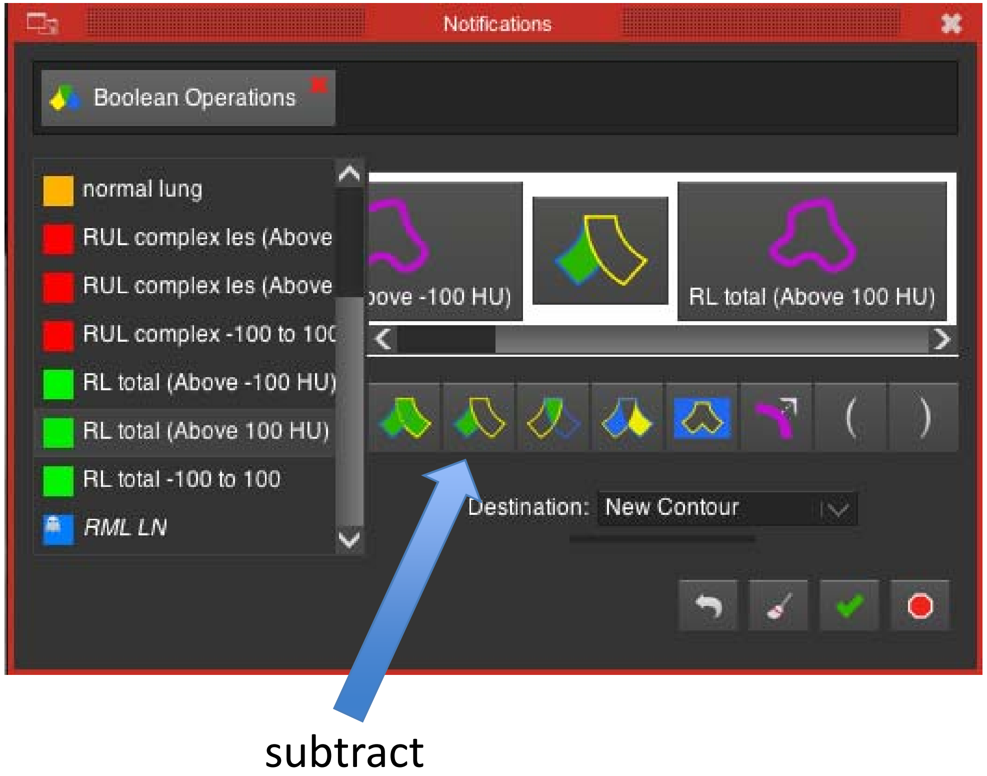

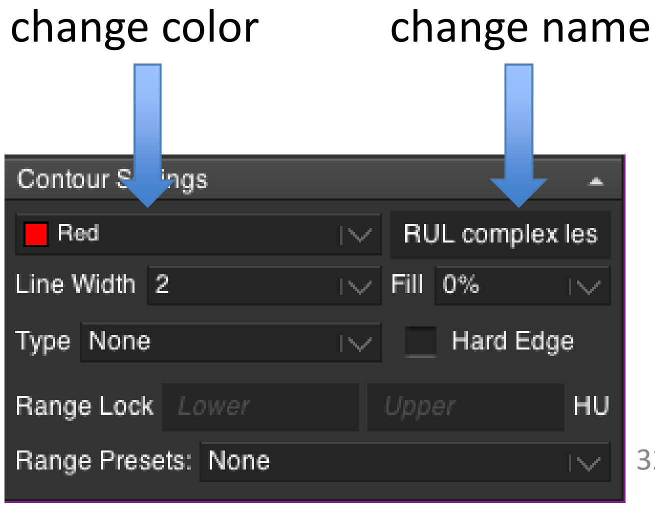

HU Hard Volume

- Note that you can place the Interpolate Contour, Subthreshold, and Boolean Operations tools into your quick access panel by clicking on the wheel

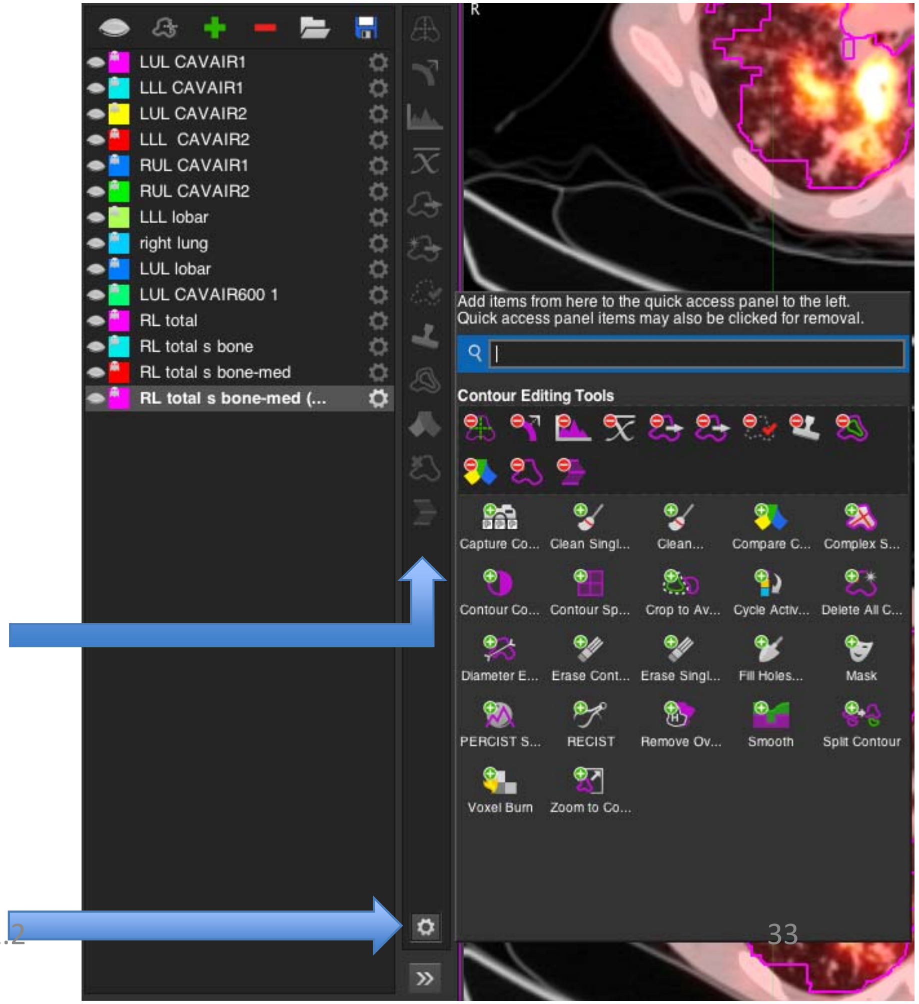

Total Lesion Glycolysis

- The same ROI may be used to analyze PET total lesion glycolysis (TLG) but with one difference.
- Unlike CT hard volume, normal lung has background PET activity, thus including too much normal lung will affect your TLG number.

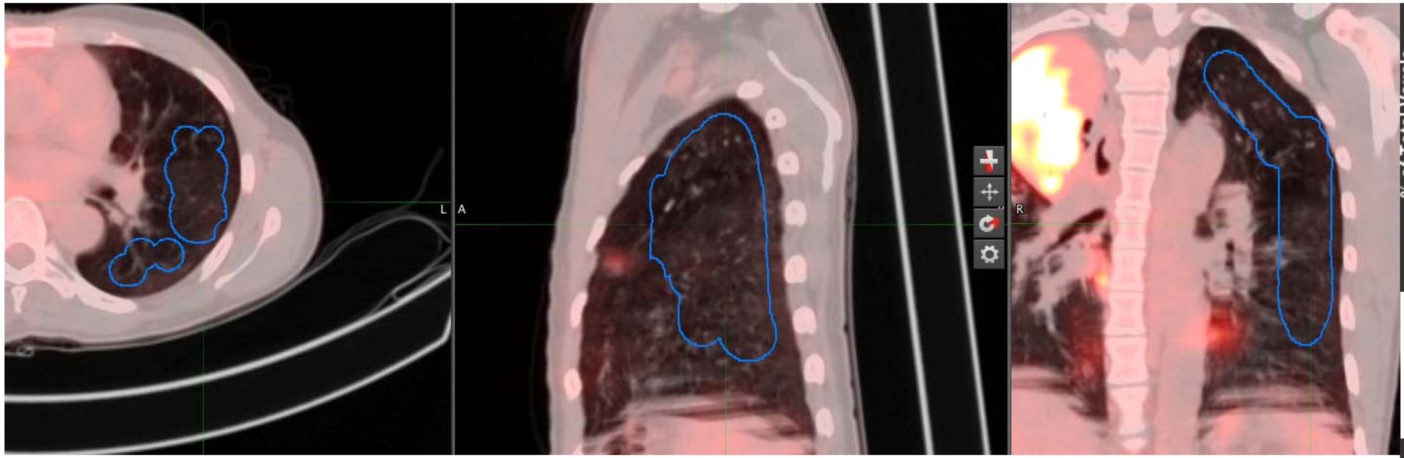

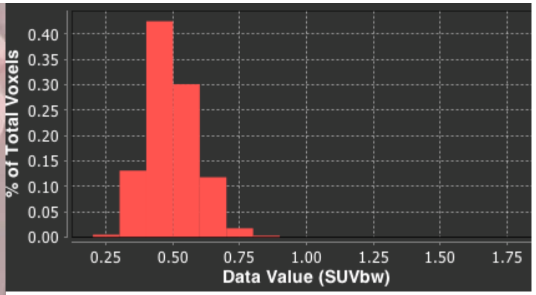

Vol: 674.8 mL

TLG: 343.8 SUVbw*mL

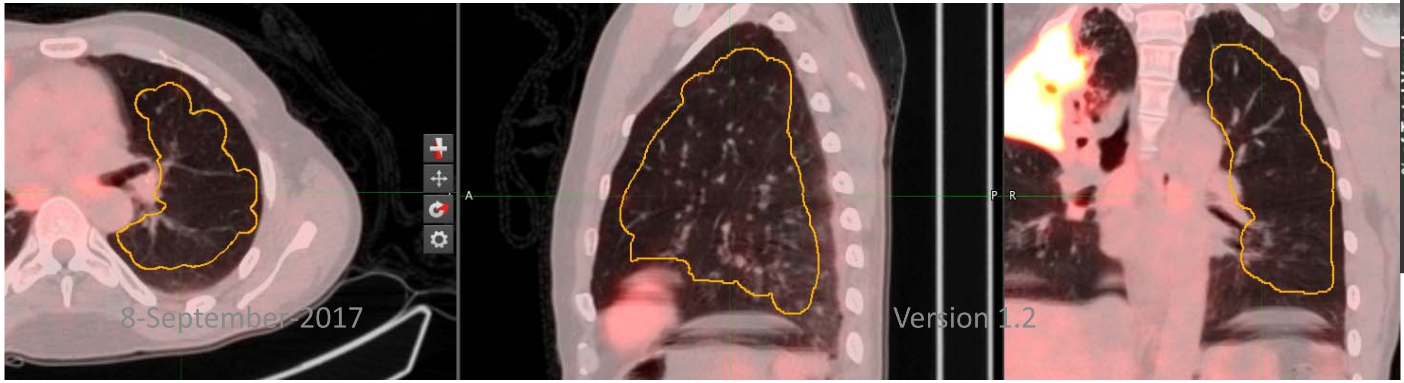

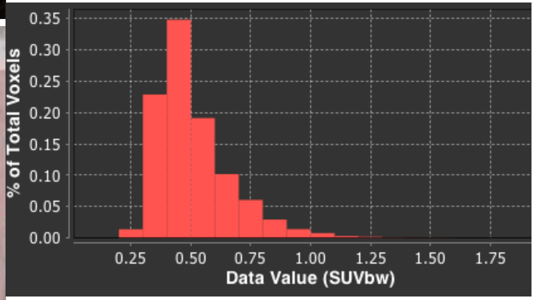

Vol: 305.9 mL

TLG: 151.6 SUVbw*mL

Total Lesion Glycolysis

- Background SUV of normal lung is generally below 1 so this can be excluded using Subthreshold.
- Dense TB lesions have very little PET activity below SUV 1 so excluding makes little difference.

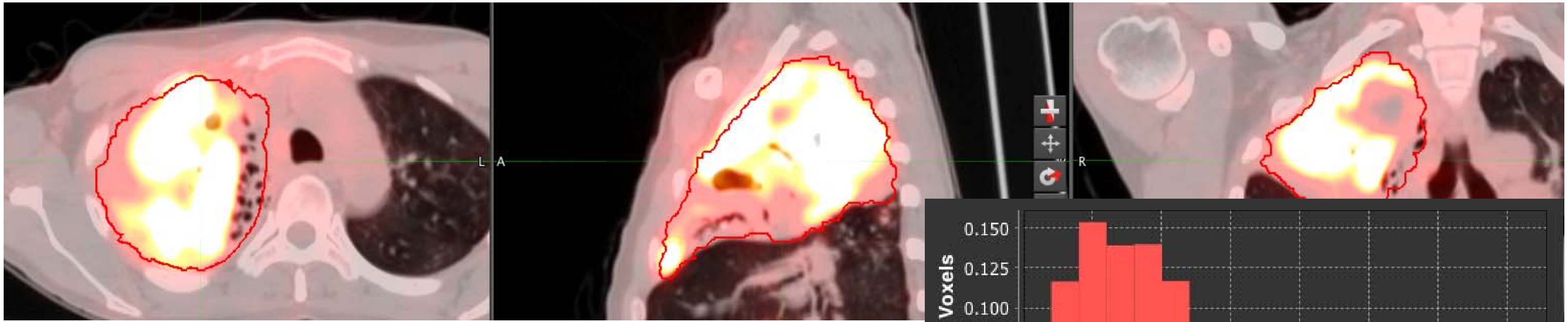

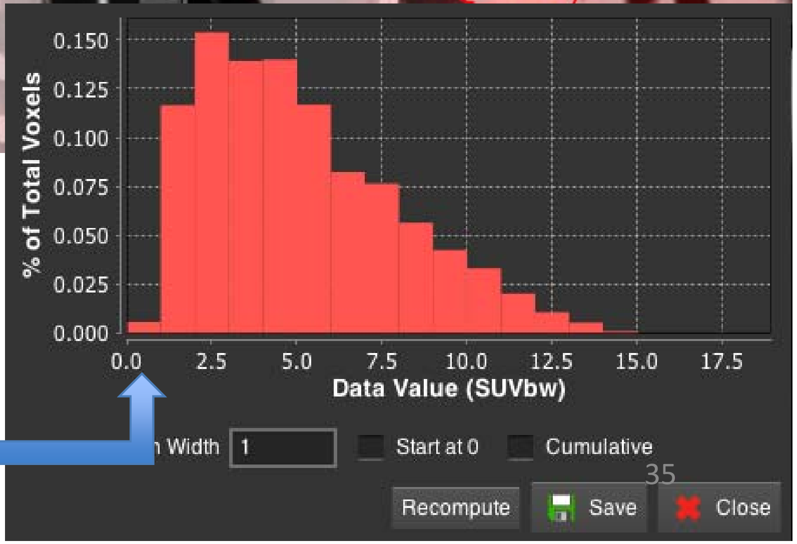

Vol: 401.4 mL
TLG: 2050.3 SUVbw*mL
TLG for SUV>1: 2048.4 SUVbw*mL

Note almost no PET activity below SUV 1

Total Lesion Glycolysis

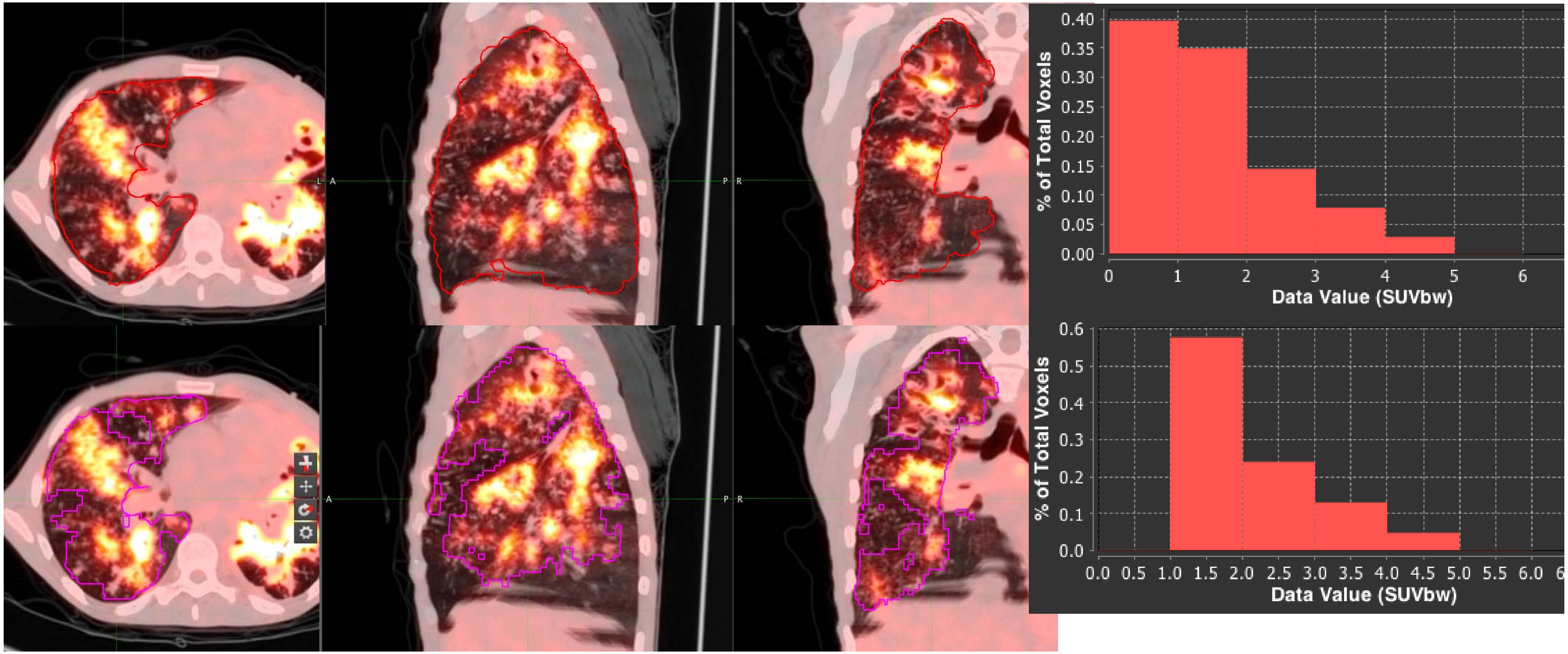

- Diffuse lesions, however, inevitably include a lot of normal lung in the ROI so excluding SUV<1 makes a bigger difference.
  - Total vol: 1956.4 mL
  - TLG: 2980.6 SUVbw*mL
  - TLG for SUV>1: 2448.7 SUVbw*mL

Total Lesion Glycolysis

- In contrast to hard vol, Subthreshold is easiest way to calculate SUV>1:
  - Set scan at top of contour box to PT1
  - Select desired ROI to subthreshold
  - Select Subthreshold from double arrow options (can place in quick access panel)
  - Type “1” in “SUVbw Threshold” dialogue box and click “OK”
  - New ROI will be generated only including SUV>1
- Use the Contour Settings box at the bottom of the contour panel to rename your new SUV >1 ROI “RightLung_W0_SUV1”.
- Repeat for left lung and rename “LeftLung_W0_SUV1”.

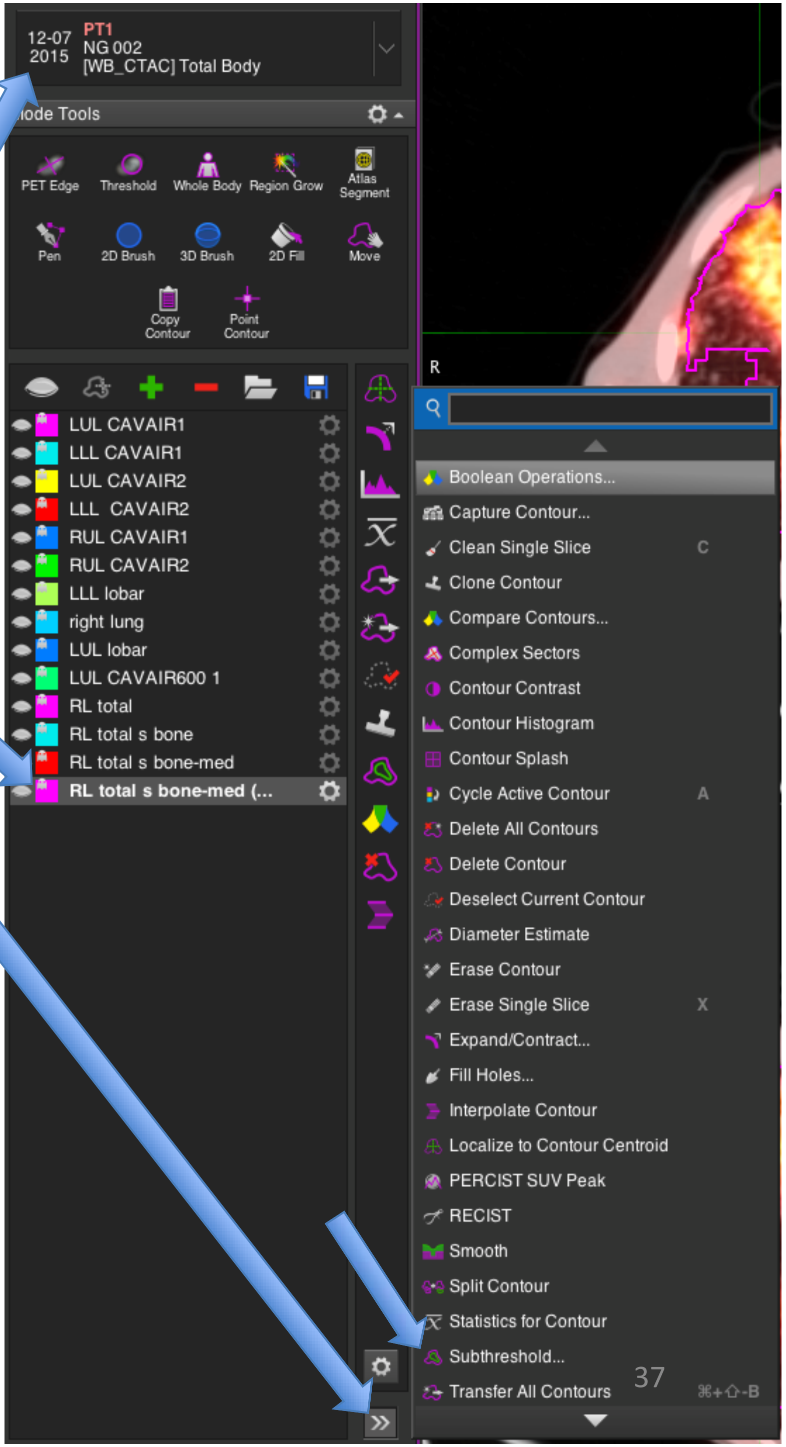

Total Lesion Glycolysis

- You can also use Range Lock instead of subthreshold but this requires more steps:
  - Select “LeftLung_W0” ROI
  - Select “Transfer Contour”; if you only have 1 scan timepoint open, the only other scan available is the PET scan so that scan is automatically selected
  - Note that scan being transferred to must be displayed on the screen
  - New ghost contour “LeftLung_W0” appears; select and rename “LeftLung_W0_SUV1”
  - Select PT1 scan at top of contour box; now “LeftLung_W0_SUV1” is no longer ghost contour and Range Lock unit now changed to SUVbw
  - In Range Lock *Lower* box, type “1”, then click “yes”
  - “LeftLung_W0_SUV1” contour now only includes SUV>1

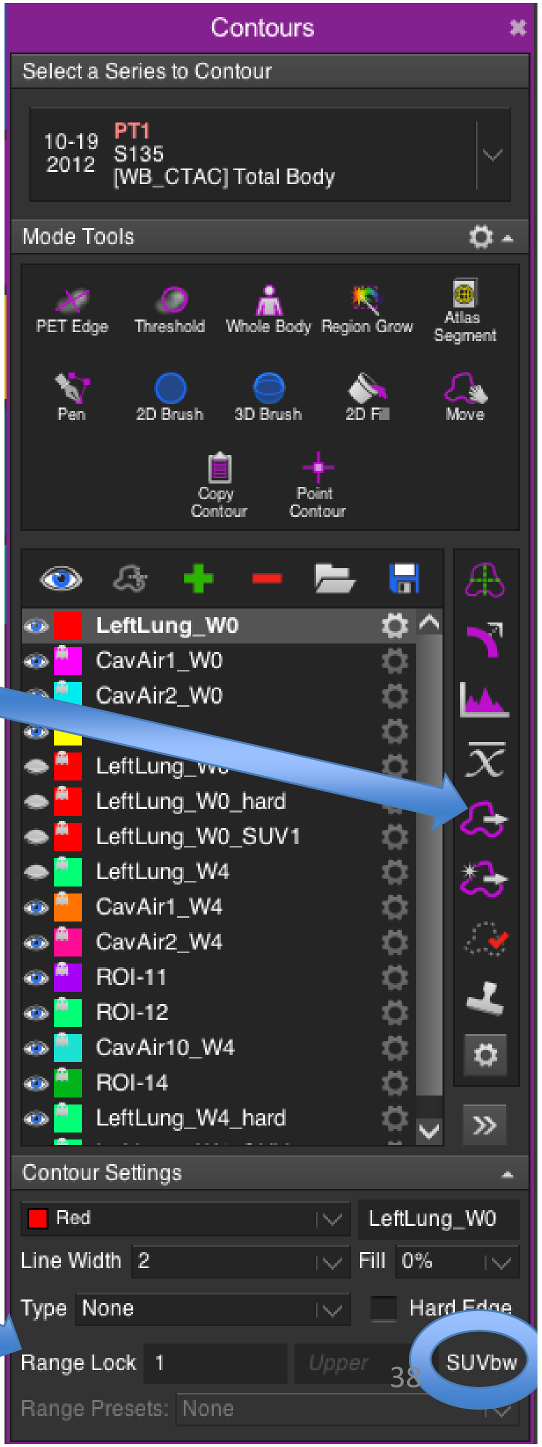

Old TB Disease

- Note that if you encounter what appears to be old TB disease, cavities or other lung parenchymal disease that have no PET uptake, do not include these old lesions in your ROI as they are unlikely to change with treatment.

Old right apical lesion with cavity

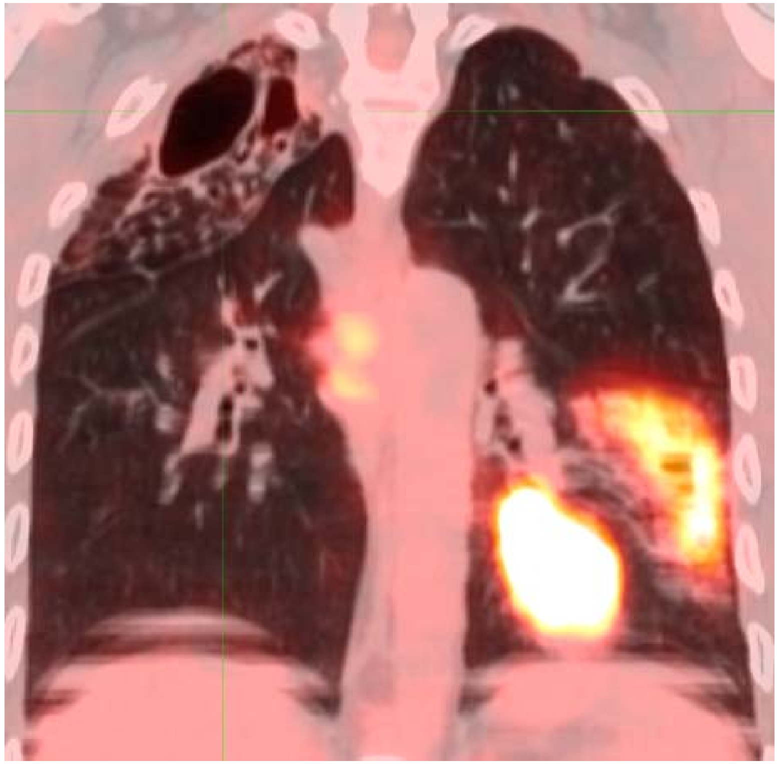

Reporting to DataFax

- Once measurements are complete, **be sure to save your session.** It is easiest to save the entire session rather than just the DICOM RTstruct.
- It is easier and less confusing to record results on the provided excel worksheet as you go by double clicking on the ROI name in the Countours box but make sure the correct scan and time point is set at the top of the box.
- Alternatively, you can click the Statistics Viewer button to see all results. This can be confusing because all results for all ROIs for all displayed scans and time points are shown so you need to be sure you are selecting the correct result.
- Note that if this button does not appear in the toolbar at the top of the screen, you can access it by clicking the double arrows to the right of this toolbar.
- You can place this button in your toolbar by clicking the wheel on the right side of the toolbar.

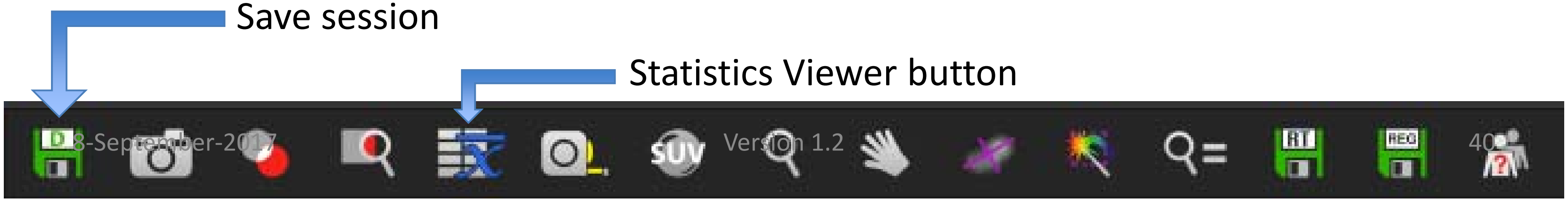

Reporting to DataFax

- To minimize confusion of recording the wrong result:
  - Be sure to only have one scan (the current scan) open in your MIM active page. The statistics viewer will show results for all active scans so this will reduce confusion.
  - After opening the statistics viewer, in the PET table select “toggle”, “statistics”, “deselect all”, then click on “total lesion glycolysis value”. This will only display TLG in the PET table.
  - In the CT table, select “toggle”, “statistics”, “deselect all”, then click on “volume value”.
  - PET TLG and CT volume are the only values you need so this will reduce the possibility of recording the wrong value.
- Record all results onto your excel worksheet.
  - File PredictTBReaderResults 2017-05-30 at Aspera [LCID-TRS_Predict](#) › [PET-CT_reader_training](#) › [PET-CT reader SOP](#) folder

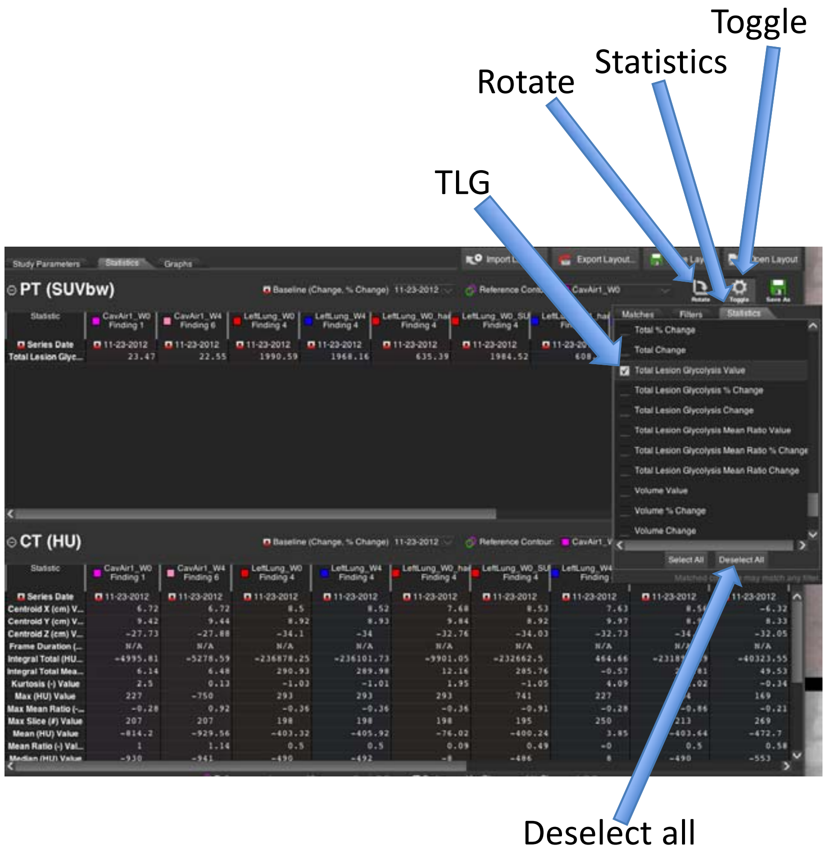

PET table now only shows TLG results. CT table has not been adjusted yet so shows all results.

Reporting to DataFax

- Log into DataFax and enter the results of your measurements (pleural thickening, collapse, pleural effusion, up to 5 cavity air volume measurements from largest to smallest, and total lung hard volume and TLG) onto the Image Reviewer form for that patient.
- Be careful to record the correct result from the correct scan and time point as hard volume and cavity air are volumes whereas TLG is not.
- Although volumes are reported in MIM on both the CT and PET scans, the results are slightly different. **For consistency, only record the CT scan volumes.**
- To double check your entered values and ensure no transcription errors were made, the system will compare your entered results with MIM.

Reporting to DataFax

- Export MIM .csv files for PET and CT statistics.
  - From the Statistics Viewer spreadsheet, select “save as” then choose “spreadsheet”.
  - Name file “[PtID]_[PET or CT]_W[week number]_[reader initials]”. Each participant should have 2 files for each scan: “S3_PET_W0_RC” and “S3_CT_W0_RC”. These will be .csv files.
  - If you “rotated” this table for viewing, be sure to rotate back to this view when you save.
- If you have Aspera Drive set up, you can place the csv files into the appropriate folder on your computer and that will automatically sync up to Aspera.
  - On my computer, this folder is in the Aspera Drive folder MIM csv file uploads > LCID-TRS_Predict_MIM_CSV_Files > rchen. This is a one-way upload folder.
  - If you do not have Aspera Drive, you will need to manually upload both files onto Aspera into your reader folder in LCID-TRS_Predict_MIM_CSV_Files. Note this folder is *different* from the folder used during the training process.
- The system will compare entered values in DataFax to the exported values in MIM:
  - If no discrepancies found, data will be released for randomization.
  - If descrepancies found, reader notified to verify and correct result in DataFax, then double check occurs again.
  - To maintain reading timelines, this correction must be completed within 24 hrs of email notification.
  - Note the QC check system is not case sensitive for the ROI names.

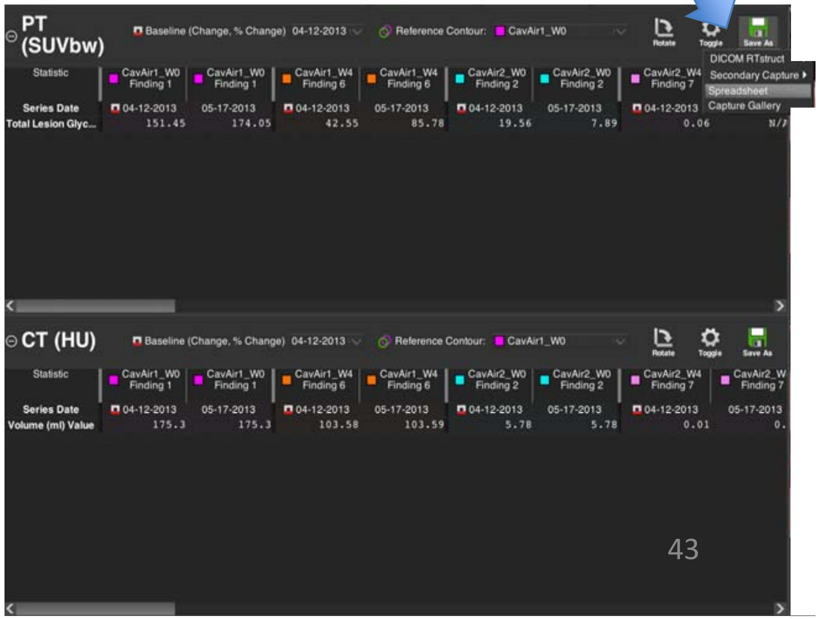

Reporting to DataFax

- After results are verified, DataFax will automatically stratify the participant as either high risk (Arm A) or low risk (Arm B/C).
- Every participant will be randomly assigned to 2 readers.
  - If the stratification by both readers agree, then image analysis is complete and stratification is assigned.
  - If stratification by both readers disagree, a 3rd reader is assigned and will be the tie-breaker.
- DataFax will automatically notify the participant's clinical team of the stratification result.

Week 4 Scan

Scan will be assigned to the same
2 readers as the baseline scan.

CT Scan Fusion

- Open previously saved session and new CTAC PET and CT scans simultaneously.
- Create a new window with just both CT scans.
- Select the "fuse two series" button from the top toolbar (may need to select the double arrow options button if not already in toolbar).
- Select the 2nd time point scan as the primary series. MIM will automatically do an alignment to fuse the 2 images but this may or may not align well.
- You can assess the alignment by comparing how well the bones (ribs, spine) overlap. Diaphragms often do not align well due to varying breath holds so don't worry about them.

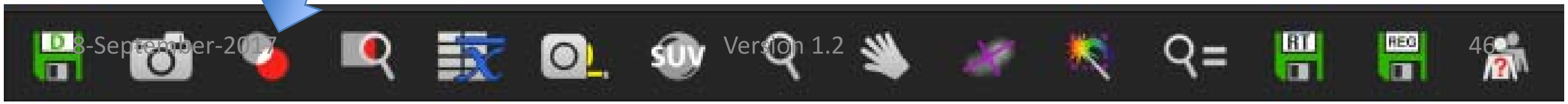

CT Scan Fusion

- Do a manual alignment by clicking the wheel on the right side of the viewer, then select “Run Rigid Assisted Alignment.” This may improve the alignment.
- Click the box below the wheel to run the alignment.
- Do a 2nd manual alignment by selecting “Box-Based Assisted Alignment” and fit the resulting box around the top half of the hemithorax, including the apices but not the diaphragms. Be sure NOT to include the scanning table in the box (note axial view).
- These manual alignments may be repeated until the scans are satisfactorily aligned.

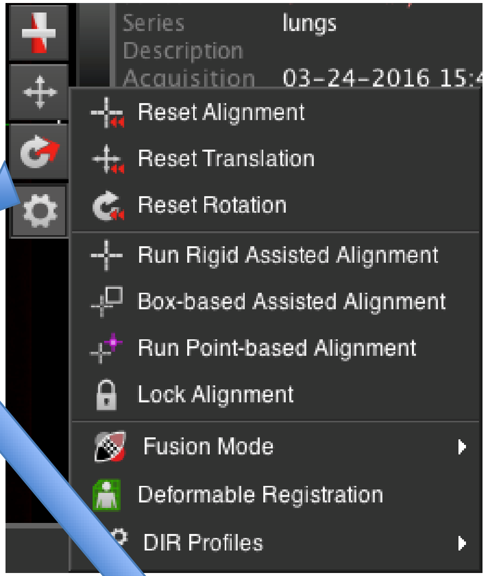

Box-based Assisted Alignment

Box-based Assisted Alignment

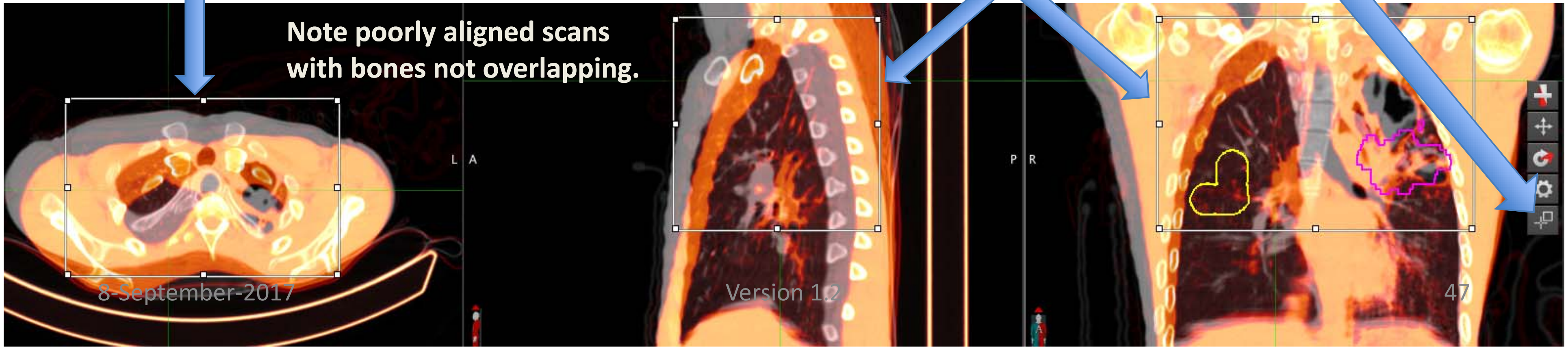

CT Scan Fusion

- Create a new window with just the fused PET/CT scan and CT-only scan for week 4.
- Resize the scans for closer viewing and change the contrast to lung window.
- You should now see the week 4 scan with your originally drawn ROIs.
- Make sure to set your PET viewing threshold to the same setting as the baseline scan. Adjust by left-click hold on this bar and sliding up or down.

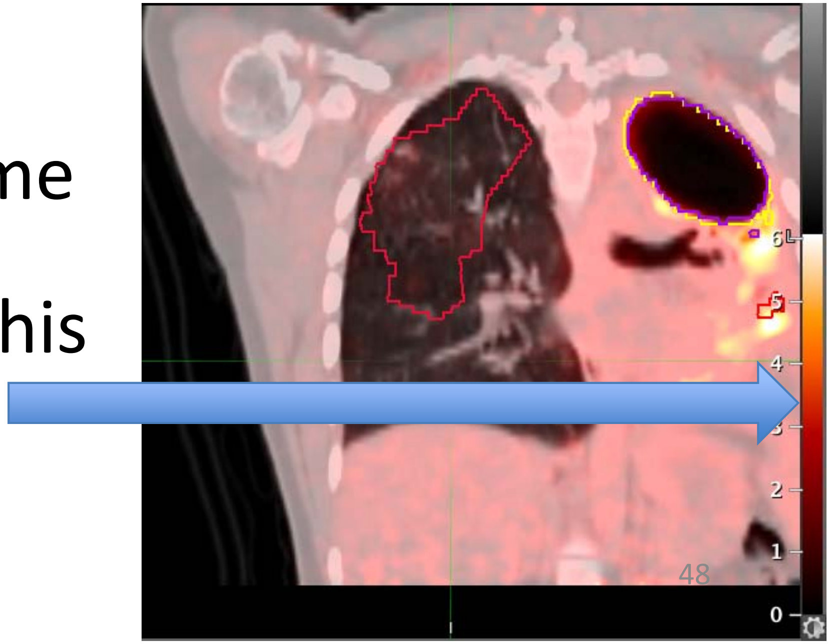

Week 4 Scan

- Clone (or transfer) your left/right lung ROIs and rename them with “W4”. CavAir contours do not need to be cloned. (Add Clone Contour to your quick access panel.)
- Note your cloned contours are still drawn on the CT1 scan; they are ghost contours on the week 4 scans.
- Revise the W4 ROIs on the week 4 scan to ensure inclusion of entire TB lesions with no overlap of bones, mediastinum, heart, or diaphragm in any view (axial, sagittal, or coronal).
- Note that sometimes it is faster to redraw the week 4 ROI from scratch than it is to revise the baseline ROI to fit the week 4 scan.
- Cavity airs need to be remeasured (new region grow ROIs) and CT hard volume and PET TLG need to be re-calculated on the week 4 scan using your new W4 left/right lung ROIs.

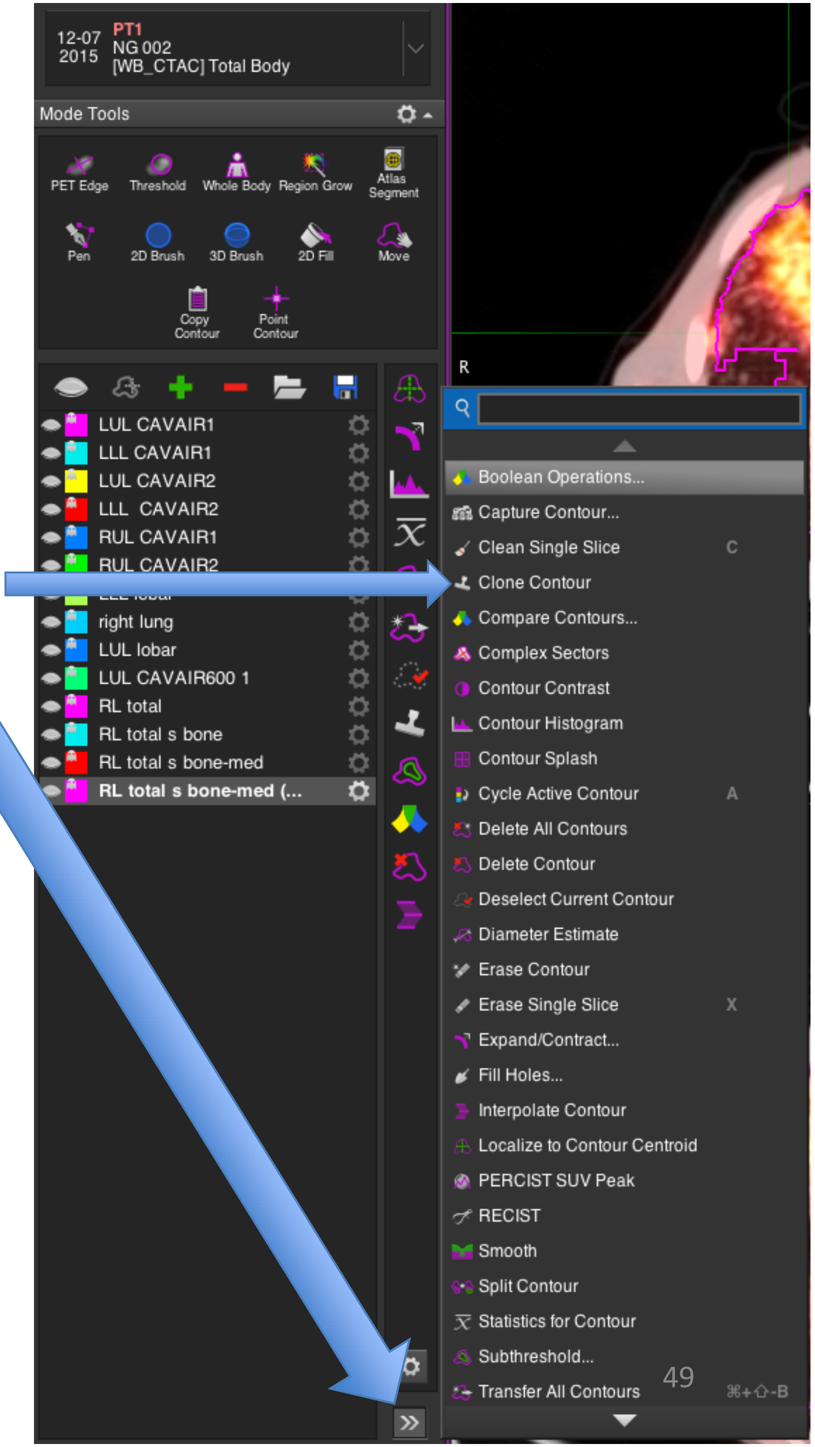

Cavity Air

- Cavity air for each cavity from the baseline scan needs to be re-measured at week 4.
- Create a new contour by clicking the green "plus" sign, then select the "Region Grow" contour tool. If you don't first create a new contour, the cavity air contour you create may be added to the last ROI you worked with.
- Select your starting seed point to grow your region by clicking in the cavity. ***Note your seed point must be on the week 4 scan cavity to measure this correctly.***
- Two green lines will appear to mark that point.

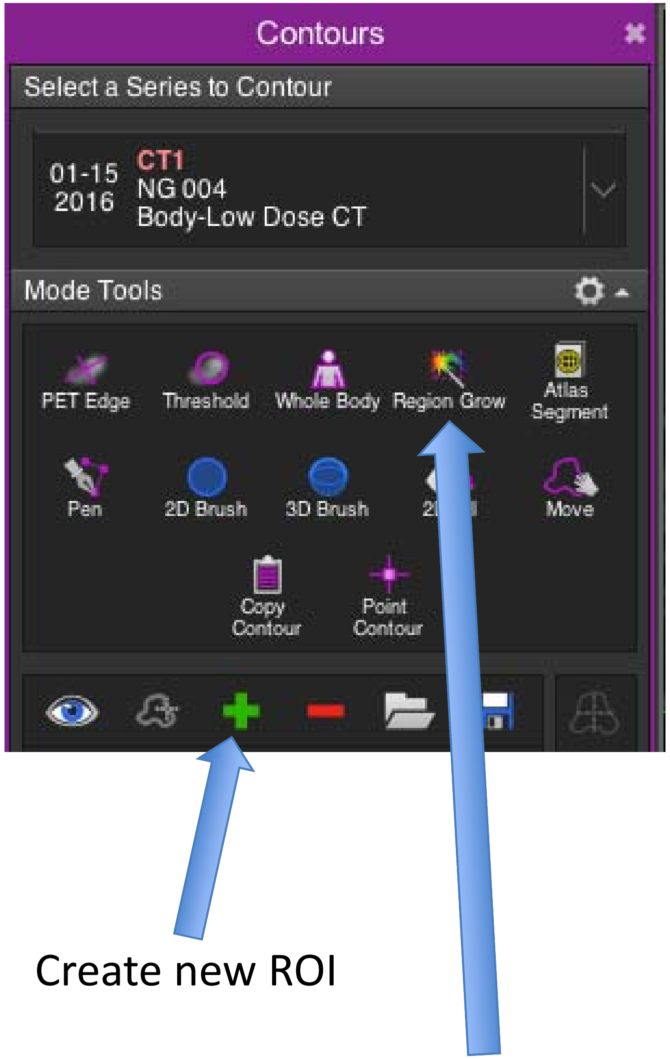

Create new ROI

Region grow tool

Cavity Air

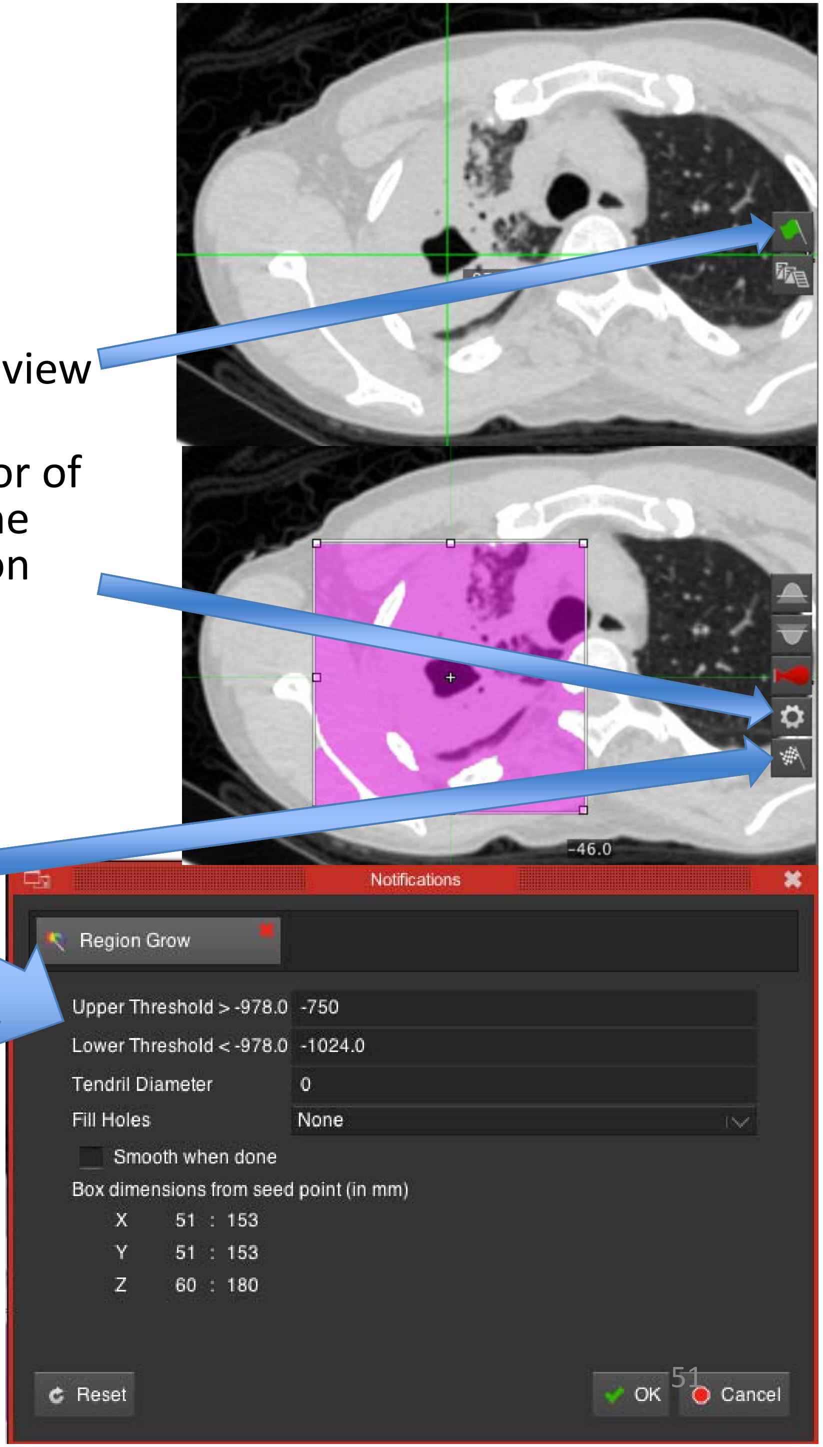

- Click on the green flag on the right side of that view to start the region grow process.
- A boundary cube will appear shaded in the color of your new ROI and a new tool bar appears on the right side. Click on the wheel to open the Region Grow dialogue box.
- Input the following:
  - Upper threshold = -750
  - Lower threshold = -1024
  - Tendril diameter = 0
  - Fill holes = none
  - Click "OK"
- Click on the flag and select "Done" to complete the region grow.
- Rename your ROI "CavAir1_W4" or similar.
- Note that if the W4 ROI does not include all of the cavity from the W0 ROI, repeat the region grow in the area not included to get a total volume equivalent to the W0 cavity.
- W4 cavities should be named in the same order as the baseline cavities.

Cavity Air

- In addition to tracking the (up to) 5 cavities noted on the baseline PET/CT scan, up to 2 new cavities may be recorded at week 4.
- If new cavities are noted, please name these ROIs CavAir10_W4 and CavAir11_W4, larger to smaller.
- If up to 2 new cavities are noted on the 3rd scan, name these ROIs CavAir20_W16 (or W24) and CavAir21_W16 (or W24), larger to smaller.
- If up to 2 new cavities are noted on the TB recurrence scan, name these ROIs CavAir30_WR and CavAir31_WR, larger to smaller.
- Note that new cavities (did not exist on previous scan) should be reported even if <2 mL.
- Persisting small cavities (cavities <2 mL at baseline that continue to be <2 mL) should still not be reported.

CT Scan

- To use Range Lock to calculate hard volume, you must first transfer your contour to the CT2 scan:
  - Make sure CT2 scan is displayed on screen
  - Select “LeftLung_W4” ROI
  - Select “Transfer Contour”
  - Select the CT2 scan (may be selected automatically)
  - A new “LeftLung_W4” contour is created on the CT2 scan; rename to “LeftLung_W4_hard”
  - Under “Range Lock”, enter “-100” and “100”; make sure unit is HU; click “yes”
  - Repeat for right lung contour

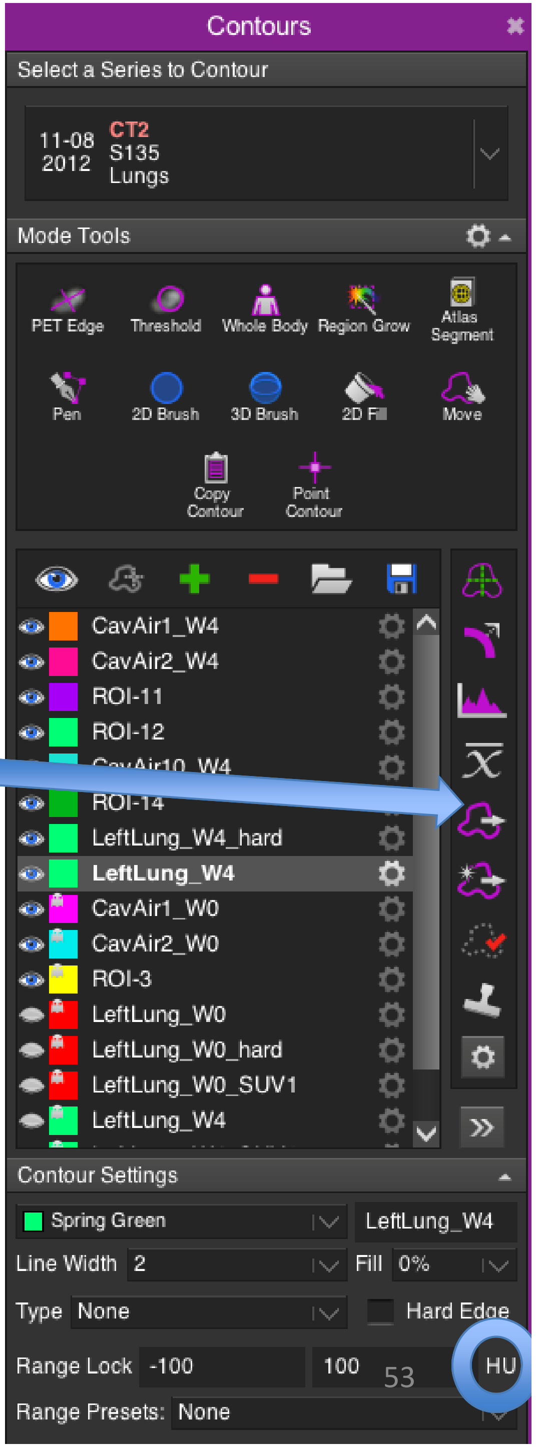

CT Scan

- To Subthreshold instead of Range Lock:
  - *****Set scan at top of contour box to CT2*****: this step is critically important to collect the correct data for the week 4 scan.
  - Select desired ROI to Subthreshold.
  - Click double arrow and select "Subthreshold."
  - Type "-100, 100" in "HU Threshold" dialogue box and click "OK."
  - 2 new ROIs will be generated, one with HU >-100 and another with HU >100.

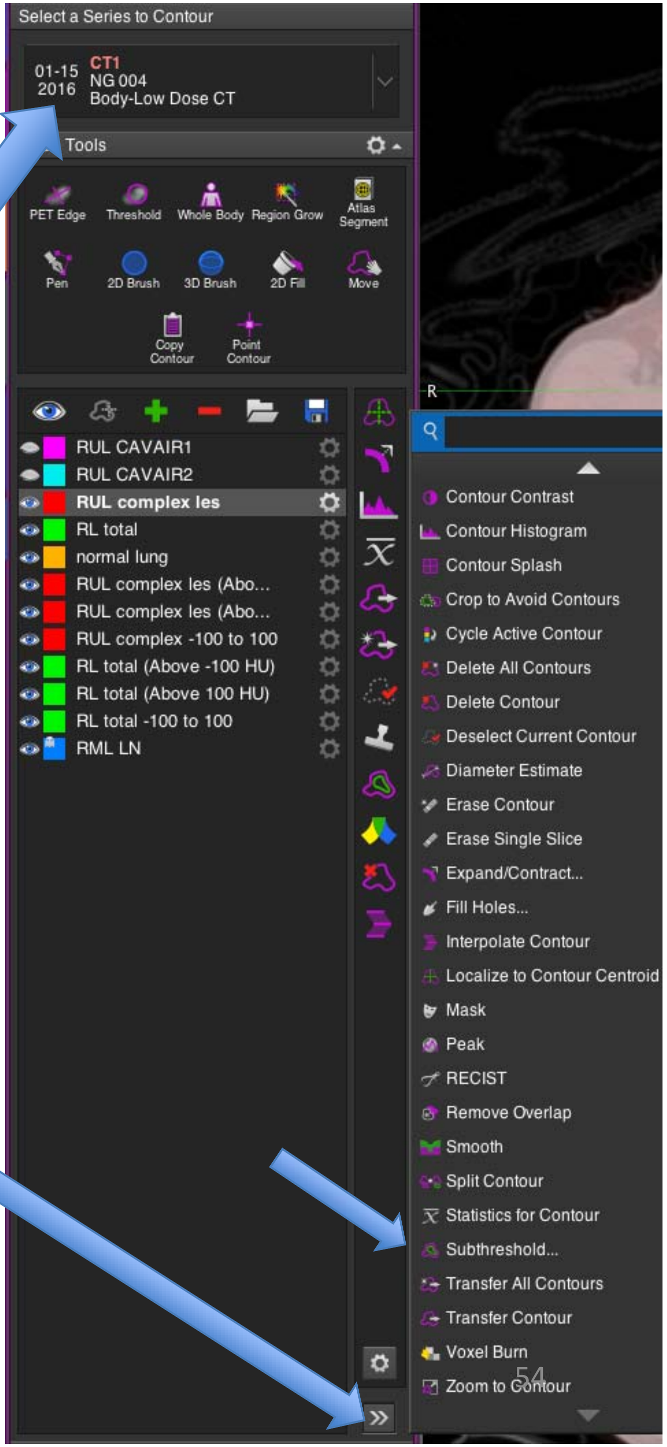

CT Scan

- To calculate hard volume, click double arrow again and select Boolean Operations.
- Select the “above -100 HU” new ROI.
- Select the “subtract” button.
- Select the “above 100 HU” new ROI.
- Click the green check mark.
- A new ROI will be generated with just -100 to 100 HU volume.
- Rename the new ROI “RightLung_W4_Hard”.
- Once you rename your new ROI, delete the two subthreshold ROIs to avoid confusion.

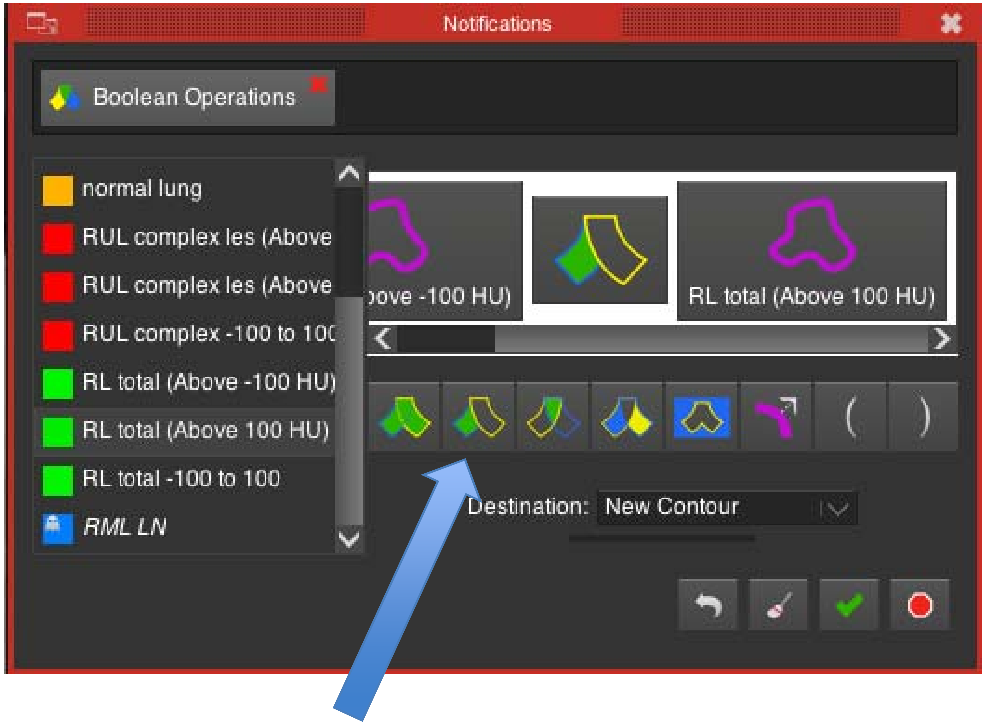

subtract

change color

change name

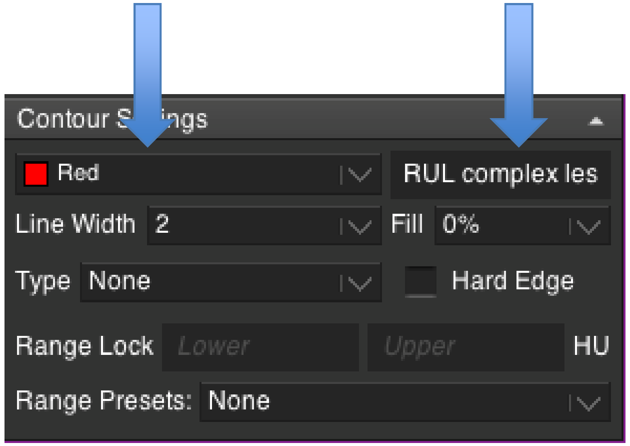

PET Scan

- To Subthreshold for SUV1:
  - *****Set scan at top of contour box to PT2.*****
  - Select desired ROI to subthreshold.
  - Select Subthreshold from double arrow options (can place Subthreshold in quick access panel).
  - Type “1” in “SUVbw Threshold” dialogue box and click “OK”.
  - New ROI will be generated only including SUV>1.
  - Rename new ROI “RightLung_W4_SUV1”.

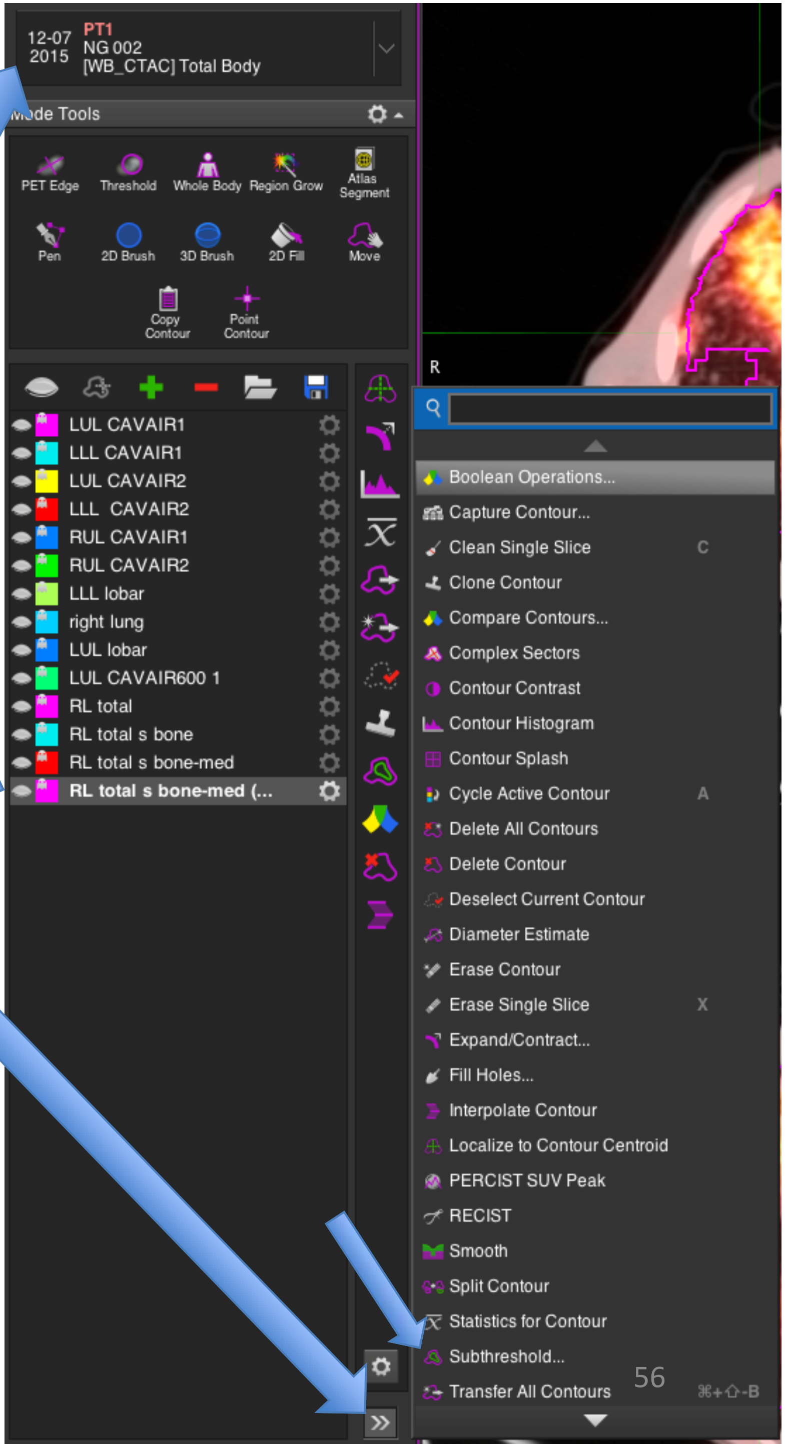

PET Scan

- To use Range Lock instead of subthreshold, you must first transfer your contour to the PT2 scan:
  - Make sure PT2 scan is displayed on screen
  - Select “LeftLung_W4” ROI
  - Select “Transfer Contour”
  - Select the PT2 scan
  - A new “LeftLung_W4” contour is created on the PT2 scan; rename to “LeftLung_W4_SUV1”
  - Under “Range Lock”, enter “1” in the *lower* box; make sure unit is SUVbw; click “ok”
  - Repeat for right lung contour

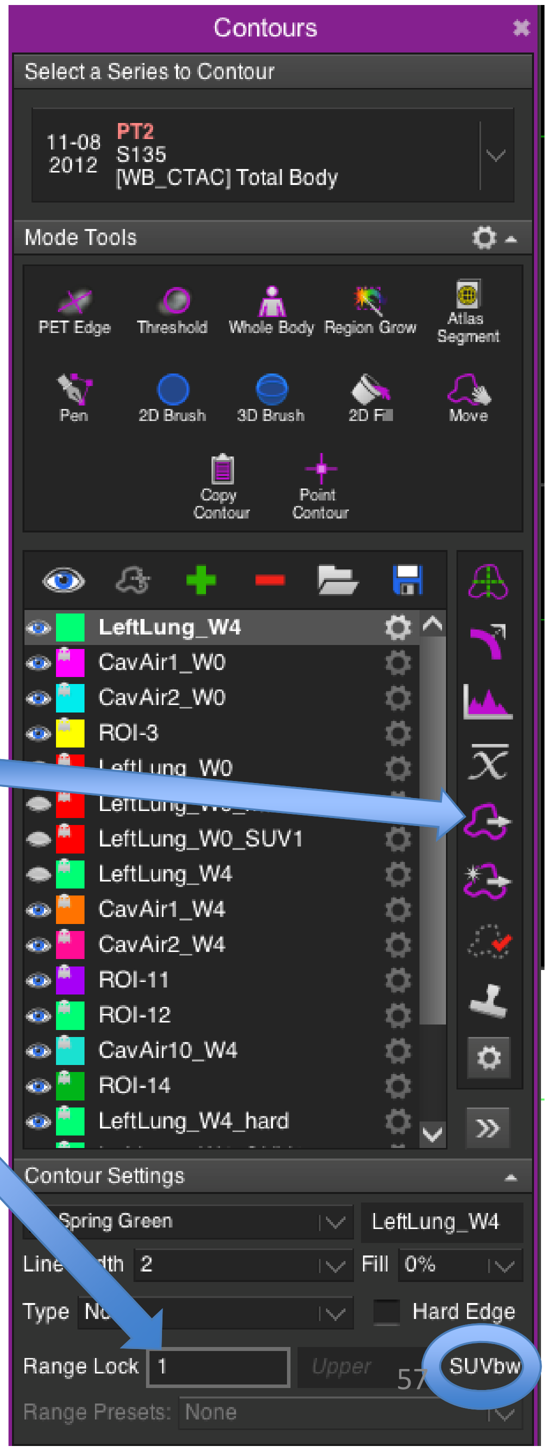

Reporting to DataFax

- Once measurements are complete, **be sure to save your session.** It is easiest to save the entire session rather than just the DICOM RTstruct.
- It is easier and less confusing to record results on the provided excel worksheet as you go by double clicking on the ROI name in the Countours box but make sure the correct scan and time point is set at the top of the box.
- Alternatively, you can click the Statistics Viewer button to see all results. This can be confusing because all results for all ROIs for all displayed scans and time points are shown so you need to be sure you are selecting the correct result.
- Note that if this button does not appear in the toolbar at the top of the screen, you can access it by clicking the double arrows to the right of this toolbar.
- You can place this button in your toolbar by clicking the wheel on the right side of the toolbar.

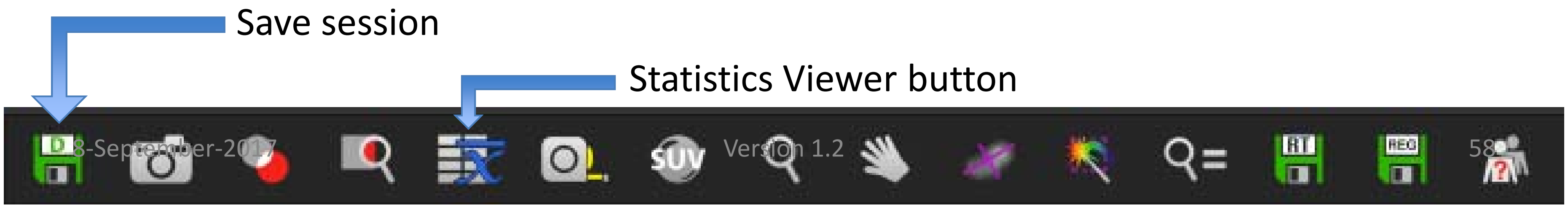

Reporting to DataFax

- To minimize confusion of recording the wrong result:
  - Be sure to only have one scan (the current scan) open in your MIM active page. The statistics viewer will show results for all active scans so this will reduce confusion.
  - After opening the statistics viewer, in the PET table select “toggle”, “statistics”, “deselect all”, then click on “total lesion glycolysis value”. This will only display TLG in the PET table.
  - In the CT table, select “toggle”, “statistics”, “deselect all”, then click on “volume value”.
  - PET TLG and CT volume are the only values you need so this will reduce the possibility of recording the wrong value.
  - You can “Rotate” the X and Y columns of the table if you find that easier to read.
- Record all results onto your excel worksheet.
  - File PredictTBReaderResults 2017-05-30 at Aspera LCID-TRS_Predict › PET-CT_reader_training › PET-CT reader SOP folder

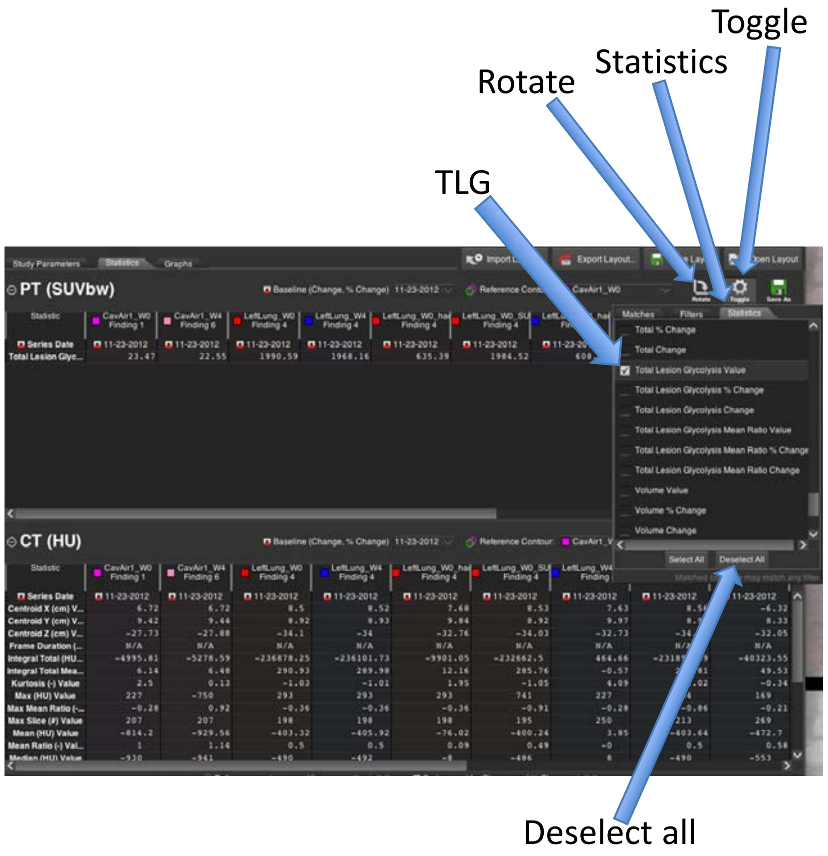

PET table now only shows TLG results. CT table has not been adjusted yet so shows all results.

Reporting to DataFax

- Log in to DataFax and input up to 5 cavity air measurements from week 4 **in the same order as the baseline measurements** (may not be ordered by size anymore; include measurements <2 mL), up to 2 additional cavities by size (include any new cavities even if <2 mL), CT hard volume, and PET total lesion glycolysis.
- It is recommended to record results as you generate them because this is less confusing.
- If you did not record results as you went, be sure you are recording the correct measurement in the Statistics Viewer as the viewer will show the results for all ROIs at all time points.
- To reduce confusion, it is easier to create a window with only a single time point when recording your results from the Statistics Viewer. If you display both baseline and week 4 scans, both time points will appear in the statistics viewer table and it is easy to confuse the two results.
- Remember that hard volume and cavity air are volumes whereas TLG is not. **Record all volumes from the CT scan, not the PET scan.**
- To double check your entered values and ensure no transcription errors were made, the system will compare your entered results with MIM.

Reporting to DataFax

- Export MIM .csv files for PET and CT statistics.
  - From the Statistics Viewer spreadsheet, select “save as” then choose “spreadsheet”.
  - Name file “[PtID]_[PET or CT]_W[week number]_[reader initials]”. Each participant should have 2 files for each scan: “S3_PET_W0_RC” and “S3_CT_W0_RC”. These will be .csv files.
  - If you “rotated” this table for viewing, be sure to rotate back to this view when you save.
- If you have Aspera Drive set up, you can place the csv files into the appropriate folder on your computer and that will automatically sync up to Aspera.
  - On my computer, this folder is in the Aspera Drive folder MIM csv file uploads > LCID-TRS_Predict_MIM_CSV_Files > rchen. This is a one-way upload folder.
  - If you do not have Aspera Drive, you will need to manually upload both files onto Aspera into your reader folder in LCID-TRS_Predict_MIM_CSV_Files. Note this folder is *different* from the folder used during the training process.
- The system will compare entered values in DataFax to the exported values in MIM:
  - If no discrepancies found, data will be released for randomization.
  - If descrepancies found, reader notified to verify and correct result in DataFax, then double check occurs again.
  - To maintain reading timelines, this correction must be completed within 24 hrs of email notification.
  - Note the QC check system is not case sensitive for the ROI names.

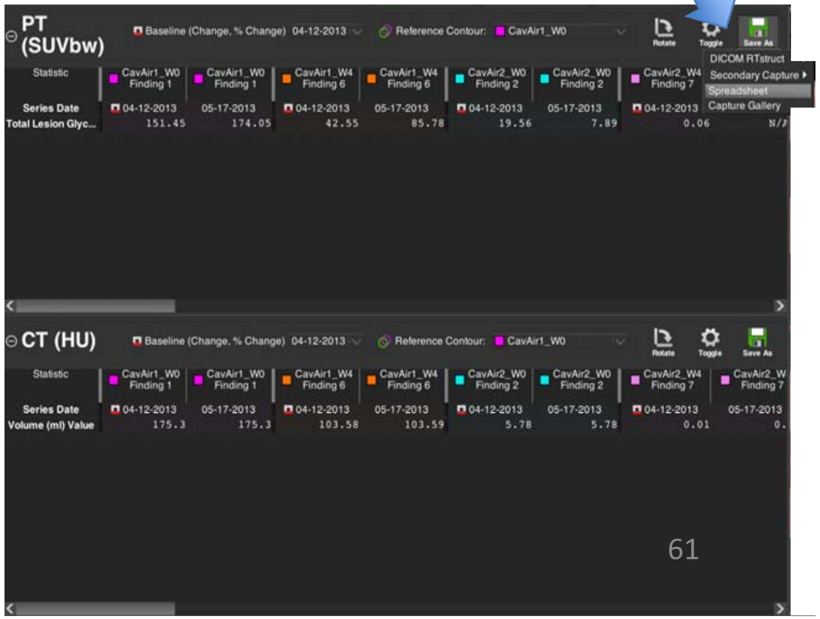

Reporting to DataFax

- After results are verified, DataFax will automatically stratify the participant as either high risk (Arm A) or low risk (Arm B/C).
- Every participant will be randomly assigned to 2 readers.
  - If the stratification by both readers agree, then image analysis is complete and stratification is assigned.
  - If stratification by both readers disagree, a 3rd reader is assigned and will be the tie-breaker.
- DataFax will automatically notify the participant's clinical team of the stratification result.
- Note the reading timeline for the week 4 scan is the most time sensitive to allow notification of sites by week 7 for the PK substudy introduction to participants at their week 8 visit.

3rd and Recurrent TB PET/CT Scans

- Repeat the same procedures to read the 3rd PET/CT scan, noting that the ROIs and files should be labeled with “W16” or “W24” as appropriate.
- Note that the cavity air Region Grow ROI will now often spill out of what is left of the cavity so you will likely need to increase the tendril diameter to contain the ROI within the cavity.
- If a participant develops recurrent TB and receives another PET/CT scan, the same procedures should be followed. This time point should be labeled “WR” for week recurrence in your ROI and file names.
  - If up to 2 new cavities are noted on the 3rd scan, name these ROIs CavAir20_W16 (or W24) and CavAir21_W16 (or W24), larger to smaller.
  - If up to 2 new cavities are noted on the TB recurrence scan, name these ROIs CavAir30_WR and CavAir31_WR, larger to smaller.
  - All new cavities should be captured even if <2 mL.
- On the recurrent scan image review form in DataFax, you will be asked whether or not the scan lesions are in the same locations as during the previous episode of active TB.
- Remember to upload MIM PET and CT .csv files for all scans onto Aspera.

Beware of Common Errors!

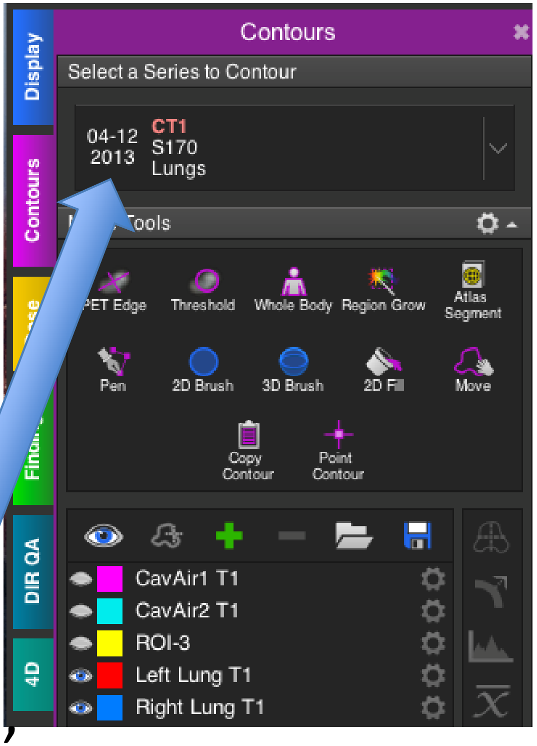

- For very large cavities, note that you may need to enlarge the Region Grow boundary cube to include the entire cavity. Double check on all views that you included the entire cavity.
- When subthresholding hard volume and TLG, make sure the scan in the upper left box is set to the appropriate scan and time point.
- Make sure you are adding the the appropriate unit – volume vs. TLG. **It is best to restrict your statistics output table to show only PET TLG and CT volume.**
- If you have 2 scan time points open simultaneously, beware of recording the result from the wrong scan or time point. It is better not to have 2 scan time points open in your active window simultaneously for this reason.

Expected Reading Timeline

Process	Time Frame
Confirmation of scan in DataFax and email from DataFax to two readers assigning PET/CT scan	2 days
Reading of PET/CT scan, electronic data entry into DataFax, and upload of MIM .csv files to Aspera	**7 days**
Data verification between DataFax and MIM outputs; email notification to reader to correct if there are discrepancies	2 days
Comparison of readers stratifications; email to 3rd reader if necessary	1 day
3rd reader reading of PET/CT scan, electronic data entry into DataFax, and upload of MIM .csv files to Aspera	**7 days**
Data verification between DataFax and MIM outputs; email notification to reader to correct if there are discrepancies	2 days
Total	21 days

- Due to required study procedures, timeline must be completed within 3 weeks.
- Readers must complete reading each scan within 7 days.
- If discrepancies are noted in comparing data entered in DataFax with MIM outputs (differences in results or ROI naming errors), readers must correct in DataFax ASAP.

MIM and Aspera QC Checks

- Image data will be downloaded from DataFax daily at 6pm ET (12 MN or 1am RSA/6 or 7am China) and from Aspera at 6:30pm ET.
- The QC check comparing the 2 sets of results will run at 7pm ET. Any discrepancies identified will be emailed to the specific reader by 7:30pm ET (1:30 or 2:30am RSA/7:30 or 8:30am China). If there are no discrepancies, there will not be an email.
- To maintain the reading timeline (previous slide), **discrepancies must be corrected within 24 hrs of the email notification**. Please make sure you are available to do this as needed the day after you upload your scan results.
- The QC check will run again the following day. Continued or new discrepancies will be emailed again. Resolved discrepancies will not be emailed.
