# Supplementary material for: Using biomarkers to predict TB treatment duration (Predict TB): a prospective, randomized, noninferiority, treatment shortening clinical trial
Source: Gates Open Res. 2017 Nov 6;1:9. [Version 1] doi: 10.12688/gatesopenres.12750.1 (PMC5841574; doi:10.12688/gatesopenres.12750.1)
Supplement: Peer review history

# Peer review: Referee response for version 1

**DOI:** 10.21956/gatesopenres.13810.r26115

**Published:** 2017-12-07

**Authors:** Payam Nahid, Beth Shoshana Zha

**Affiliations:** 1 University of California, San Francisco, San Francisco, CA, USA; 2 Division of Pulmonary and Critical Care Medicine, University of California, San Francisco, San Francisco, CA, USA

**Summary**

 Chen and colleagues have provided a well-written protocol describing a randomized multi-site trial to determine if 4 months of treatment is noninferior to standard 6 months in low-risk pulmonary tuberculosis patients. The overall aim of this study is to identify patients with earlier cure through previously researched biomarkers of GeneXpert cycle thresholds and imaging markers on PET/CT scans.

 Patients will be stratified by defined early treatment criteria, which incorporates initial PET/CT and imaging changes at 4 weeks, medication adherence, and GeneXpert cycle thresholds at 4 months. Those identified as low-risk will be randomized to 4 versus 6 months of treatment, while patients classified to have severe disease will complete a full 6 months of treatment. The primary endpoint is defined as treatment success at 18 months, with detailed attention to determine relapse versus repeat infection to clearly define treatment failure.

 Chen *et al* provide a sound rationale for the trial based on multiple pre-clinical and clinical studies. Methods are well written, and supplemental material provides further detail which would allow easy reducibility. Statistical analysis described are appropriate for the trial proposed. Overall, we have only a few minor comments.

**Details**

**Introduction**

 The authors provide a well-written summary of how standard-of-care for pulmonary tuberculosis was defined as 6 months, and recent trials conducted which demonstrate unacceptable rates of relapse when treatment is shortened to 4 months.

**Methods**

 The study design is clearly defined for study sites, inclusion and exclusion criteria, data collection and responsibilities, and methods to address adherence. In addition, statistical analysis is well explained, and power calculations provided for support of aimed sample size. A few clarifications would be helpful:

1. Method of randomization appears to be only in supplemental protocol, and would be useful to list it in the main proposal.
2. Are PET/CT readers blinded to the patient randomization?
3. The range of weight in inclusion criteria is broad – this could further be noted in the PK-MIC substudy.
4. Please consider adding additional info on analysis of PK samples (location, methods etc).

 

**Discussion**

 The discussion is well-written and provides further detailed insight into the design approach. With this discussion, the reader can understand the cut off of GeneXpert cycle threshold of >30. Additional detail on the reasoning for timing of PET/CT at 4 weeks rather than a later date would be helpful. Reference 26 demonstrated changes on PET at 8 weeks compared to baseline was a better predictor of cure than liquid culture in MDR-TB patients. While reference 27 utilized 4 week PET/CT rather than 8 week in drug-sensitive patients, the authors described persistent PET activity likely related to inflammation and not necessarily active infection, which would be ongoing after 4 weeks of treatment.

We have read this submission. We believe that we have an appropriate level of expertise to confirm that it is of an acceptable scientific standard.

# Response

**Published:** 2018-01-10

**Authors:** Ray Chen

We thank the reviewers for their review and comments. We have responded point by point below.

**Methods**

The study design is clearly defined for study sites, inclusion and exclusion criteria, data collection and responsibilities, and methods to address adherence. In addition, statistical analysis is well explained, and power calculations provided for support of aimed sample size. A few clarifications would be helpful:

1. Method of randomization appears to be only in supplemental protocol, and would be useful to list it in the main proposal.

**The method of randomization is described in the statistical analysis plan, although it is our practice to keep precise details (e.g., block size) restricted. In brief, participants who meet baseline, week 4, and week 16 early completion criteria are block randomized (stratified by site) to Arm B (continue treatment to week 24) or Arm C (complete treatment early at week 16).**

       2. Are PET/CT readers blinded to the patient randomization?

**For randomization to either arm B or C, blinding is not a concern, as patient randomization does not occur until after the reader scores are entered into the database. Indeed, these results are needed to determine risk classification, which determines eligibility for randomization. Furthermore, PET/CT readers are blinded to other reader’s results.**

**The other randomization element in this study occurs amongst arm A participants. The timing of imaging scans for arm A participants is randomized to either 16 or 24 weeks.  PET/CT readers are not blinded to the timing of the scan.**

The range of weight in inclusion criteria is broad – this could further be noted in the PK-MIC substudy.

**This will be noted in future publications related to the PK-MIC substudy.**

      3. Please consider adding additional info on analysis of PK samples (location, methods etc).

**In Cape Town, PK sample analysis will be performed on plasma samples shipped to the Rutgers New Jersey Medical School in Newark, New Jersey, USA. In China, PK sample analysis will be performed on plasma samples shipped to Shanghai Public Health Clinical Center in Shanghai. The samples will be analyzed by high pressure liquid chromatography coupled to a tandem mass spectrometer as previously described (** **Antimicrob Agents Chemother. 2012 Jan; 56(1): 446–457.** **doi: 10.1128/AAC.05208-11** **). The equipment and methods used by the two labs are harmonized currently and will be revalidated as producing similar results prior to protocol sample analysis. Data processing will be performed using a current version of Analyst Software (Applied Biosystems Sciex).**

**Discussion**

The discussion is well-written and provides further detailed insight into the design approach. With this discussion, the reader can understand the cut off of GeneXpert cycle threshold of >30. Additional detail on the reasoning for timing of PET/CT at 4 weeks rather than a later date would be helpful. Reference 26 demonstrated changes on PET at 8 weeks compared to baseline was a better predictor of cure than liquid culture in MDR-TB patients. While reference 27 utilized 4 week PET/CT rather than 8 week in drug-sensitive patients, the authors described persistent PET activity likely related to inflammation and not necessarily active infection, which would be ongoing after 4 weeks of treatment.

**Our decision to use a week 4 PET/CT scan was based primarily on data from reference 27 which concerned drug-sensitive patients; the 8-week changes in reference 26 were in MDR patients. The overall slower response in MDR patients compared to drug susceptible patients suggests that a similar outcome-predictive timepoint would be shorter than 8 weeks. Therefore, we chose to use the 4-week changes in drug susceptible patients for this study. Although inflammation is still ongoing at week 4 compared to later time points, there appeared to be enough change from baseline to week 4 to be predictive of final treatment outcomes. Later time points may have less inflammation and possibly be more predictive but a certain amount of inflammation still persists even 1 year after completion of treatment in apparently cured patients, as noted in ref 27. Our goal therefore was to identify an early time point that was still predictive of final treatment outcomes. We hypothesized that a week 4 scan would accomplish this.**

# Peer review: Referee response for version 1

**DOI:** 10.21956/gatesopenres.13810.r26111

**Published:** 2017-12-01

**Authors:** Andrew J. Nunn, Conor Tweed

**Affiliations:** 1 Medical Research Council UK Clinical Trials Unit, University College London, London, UK

**General Comments**

 This is a well-designed study addressing an issue that has challenged researchers ever since the successful demonstration of short course chemotherapy in the 1970s and Wallace Fox’s paper *Whither Short Course chemotherapy?* in which he reported on favourable outcomes in a high proportion of patients treated for less than 6 months with the SHRZ/HR regimen. Fox made the point that many patients were being treated for an unnecessarily long time and highlighted the benefits there would be if appropriate prognostic factors could be identified to permit selection of those patients requiring longer treatment. In a subsequent study conducted in Hong Kong in patients with smear negative disease low relapse rates were found after treatment with a 4-month regimen, which was recommended for treatment of smear-negative disease whatever the initial culture status.

 It is of note that policy of treating patients with smear negative disease for the reduced duration of 4 months was subsequently discontinued in the interests of a standard 6-month regimen for all patients.  

**Inclusion criteria**

 It is of note that patients are included based on results of a GeneXpert test but without reference to their smear status, it would be beneficial to include the results of the baseline smear in the analysis to ascertain whether it predicts outcome as well as it did in the Hong Kong study. Additionally, it may be worth considering including a minimum cycle threshold for inclusion into the trial.

 There is no discussion of why HIV-infected patients have been excluded from the study. Published reviews such as those by Khan in 2010 and 2012 suggested that HIV-positive patients may require a longer duration of chemotherapy than those who are uninfected although much of the data derived from studies before the era of universal ART for patients who were coinfected. Inclusion of patients from South Africa with HIV coinfection receiving ART would have been particularly informative in view of the high proportion of patients affected. A lower bacillary load and limited evidence of cavitated disease may well have different implications in this patient group.

**Adherence criteria**

 Reference 7 on which the rationale for requiring approximately 90% of prescribed treatment to have been received is based on a study of patients with MDR-TB. Recent work by Rada Savic and colleagues has demonstrated this to be the case in patients receiving standard treatment as in the current trial. Use of MERM will presumably provide valuable data on the extent to which patients have opened the electronic pill box throughout the course of treatment. It does not mean the patients will always have taken their treatment every time they open the box! Are there any plans to ask patients about adherence either during the trial or at an exit interview, which can prove to be a more informative measure.

**Treatment outcome definitions**

 I note the fact that solid media is being used for the primary analysis but liquid media may be used for secondary analysis. What was the rationale for using solid media?

 With regard to patients who have a single positive culture either at their last scheduled visit or prior to defaulting presumably this will be sufficient to classify such patients as having an unfavourable outcome, failure during treatment, or recurrence after stopping?

 How will other patients who are lost are during treatment be classified? Unassessable or unfavourable? Will those lost during follow-up be regarded as unassessable in the analysis?

We have read this submission. We believe that we have an appropriate level of expertise to confirm that it is of an acceptable scientific standard.

## References

[1] FoxW: Whither short-course chemotherapy?. *Br J Dis Chest*.1981;75(4) :331-57 703037710.1016/0007-0971(81)90022-x

[2] A controlled trial of 3-month, 4-month, and 6-month regimens of chemotherapy for sputum-smear-negative pulmonary tuberculosis. Results at 5 years. Hong Kong Chest Service/Tuberculosis Research Centre, Madras/British Medical Research Council. *Am Rev Respir Dis*.1989;139(4) : 10.1164/ajrccm/139.4.871 871-6 10.1164/ajrccm/139.4.871 2648911

[3] KhanFAMinionJPaiMRoyceSBurmanWHarriesADMenziesD: Treatment of active tuberculosis in HIV-coinfected patients: a systematic review and meta-analysis. *Clin Infect Dis*.2010;50(9) : 10.1086/651686 1288-99 10.1086/651686 20353364

[4] Ahmad KhanFMinionJAl-MotairiABenedettiAHarriesADMenziesD: An updated systematic review and meta-analysis on the treatment of active tuberculosis in patients with HIV infection. *Clin Infect Dis*.2012;55(8) : 10.1093/cid/cis630 1154-63 10.1093/cid/cis630 22820541

[5] NunnAAnywaineZSeeleyJMunderiPLevinJKasiryeRKamaliAAbaasaAGrosskurthH: Exit interviews administered to patients participating in the COSTOP placebo controlled randomised trial in Uganda. *Contemp Clin Trials Commun*.2016;3: 10.1016/j.conctc.2016.05.008 142-146 10.1016/j.conctc.2016.05.008 27536738PMC4918030

# Response

**Published:** 2017-12-05

**Authors:** Ray Chen

We thank the reviewers for their review and comments. We have responded point by point below.

**Inclusion criteria**

It is of note that patients are included based on results of a GeneXpert test but without reference to their smear status, it would be beneficial to include the results of the baseline smear in the analysis to ascertain whether it predicts outcome as well as it did in the Hong Kong study. Additionally, it may be worth considering including a minimum cycle threshold for inclusion into the trial.

***Our goal is to include participants with a wide range of TB disease severity. Hence, we use GeneXpert positive as an inclusion criterion regardless of smear status. We do collect enrollment smear status so this can be analyzed. For GeneXpert cycle threshold, the inclusion criterion states that this test must be positive, which equates to a cycle threshold of <39.***

There is no discussion of why HIV-infected patients have been excluded from the study. Published reviews such as those by Khan in 2010 and 2012 suggested that HIV-positive patients may require a longer duration of chemotherapy than those who are uninfected although much of the data derived from studies before the era of universal ART for patients who were coinfected. Inclusion of patients from South Africa with HIV coinfection receiving ART would have been particularly informative in view of the high proportion of patients affected. A lower bacillary load and limited evidence of cavitated disease may well have different implications in this patient group.

***We agree that studying the differences in HIV positive vs negative TB patients is critically important. However, for this initial proof-of-concept trial, we were unsure of the possible confounding effect that HIV infection might have on PET/CT scanning response and immunological and other biomarker responses. Thus, for this initial study, we elected to exclude HIV infected participants.***

**Adherence criteria**

Reference 7 on which the rationale for requiring approximately 90% of prescribed treatment to have been received is based on a study of patients with MDR-TB. Recent work by Rada Savic and colleagues has demonstrated this to be the case in patients receiving standard treatment as in the current trial. Use of MERM will presumably provide valuable data on the extent to which patients have opened the electronic pill box throughout the course of treatment. It does not mean the patients will always have taken their treatment every time they open the box! Are there any plans to ask patients about adherence either during the trial or at an exit interview, which can prove to be a more informative measure.

***Detailed adherence data will be collected at every visit while the participant is on treatment, including participant self-reported adherence, pill counts, and MERM data.***

**Treatment outcome definitions**

I note the fact that solid media is being used for the primary analysis but liquid media may be used for secondary analysis. What was the rationale for using solid media?

***Solid medium was selected as the primary endpoint to reduce variability associated with false positive liquid culture results possibly due to laboratory cross-contamination. We note that the reviewer’s recent publication (Phillips et al. BMC Medicine (2017) 15:207) comparing solid vs. liquid culture results from the REMoxTB trial found that such laboratory cross-contamination did sometimes occur, although additional factors also may have played a role. Using liquid culture results as the primary endpoint would also have been a reasonable choice.***

With regard to patients who have a single positive culture either at their last scheduled visit or prior to defaulting presumably this will be sufficient to classify such patients as having an unfavourable outcome, failure during treatment, or recurrence after stopping?

***Participants who default will not be eligible for randomization. Participants who are randomized but then have a single positive culture on their last visit and cannot be brought back for a confirmatory culture will have their single positive culture strain typed. If the strain type is consistent with their original TB strain, this participant will be classified as “unfavorable.” If the strain type is consistent with a new TB infection, the participant will be classified as “favorable.”***

How will other patients who are lost are during treatment be classified? Unassessable or unfavourable? Will those lost during follow-up be regarded as unassessable in the analysis?

***As indicated above, participants who are lost during treatment, are not eligible for randomization and will not be included in the primary analysis. Participants lost during follow-up will be considered “unassessable” for the primary analysis. The number of randomized participants who are lost during follow-up is anticipated to be small, given the eligibility requirements for randomization. However, this will be closely monitored during the study. Additionally, sensitivity analyses will evaluate the impact of considering these as unassessable considering the time at which the participant was lost to follow-up and their status at that time.***

# Peer review: Referee response for version 1

**DOI:** 10.21956/gatesopenres.13810.r26114

**Published:** 2017-11-27

**Authors:** Toyin Togun, Emily MacLean

**Affiliations:** 1 McGill International TB Centre and Department of Epidemiology and Biostatistics, McGill University, Montreal, QC, Canada; 2 Department of Epidemiology, Biostatistics and Occupational Health, McGill University, Montreal, QC, Canada

**Summary**

 The protocol of this PREDICT TB study by Chen and colleagues describes a prospective, multi-site, non-inferiority, randomized trial of treatment shortening in pulmonary TB (PTB) patients stratified by a refined early treatment completion criteria. The aim is to identify low-risk PTB patients who could be cured with a four-month course of anti-TB therapy (compared to the standard six-month regimen) by a combination of radiographic characteristics and markers of residual bacterial load i.e. PET/CT scan results at baseline and changes after one month of treatment and GeneXpert Ct values - a predictor of bacterial load.

 All eligible PTB patients will commence the standard treatment regimen, while participants who meet ALL criteria for early treatment completion as defined will be randomized at 16 weeks to either continue treatment to the full 24 weeks or to complete treatment at 16 weeks with clearly defined follow up protocol. PTB patients who did not qualify for early treatment completion criteria at baseline, or fail early treatment completion criteria at 1-month follow-up will continue standard treatment to 24 weeks. The primary outpoint was treatment success rate at 18 months between the randomized groups.

 This is a very well designed RCT with clear and unambiguous hypothesis, which is based on rational and credible scientific data. The study is justified and attempts to answer a very topical question relating to the quest for shortened treatment of pulmonary TB patients.

**Specific comments**

**Introduction:**

 - The protocol presents a good background to the proposed study with a review of previous studies that have attempted to shorten duration of TB treatment and the outcome. Background is well written, hypothesis is clear and credible and the authors have provided a clear justification for the RCT. However, we will suggest a revision of the hypothesis to state clearly that the focus of the study is specifically ‘pulmonary tuberculosis’.

**Methods:**

 The design of the RCT is clear and appropriate for the study aims and objectives in general. Particularly important is the very clear and unambiguous definition of criteria for early treatment completion. Having stated this, there are a number of minor methodological issues that need to be stated more clearly in the protocol:

- What was the sampling strategy for eligible participants at the study sites?
- There should be some statement about the qualification or specialization of the PET/CT readers to specify or indicate their minimum qualification and experience.
- Will the PET readers be blinded to the other patient characteristics including the clinical and laboratory results or not?
- Will the third reader who is to resolve any discrepant PET/CT scan be a more senior expert or this is to be assumed?
- There is no explicit mention in the protocol of what happens to patients who pass the early treatment completion criteria at baseline and at 4 weeks follow up, but found smear/culture and/or Xpert positive at 8 weeks or at 12 weeks follow-up (smear/culture only). One can assume that the likelihood of this happening might be low especially among the subjects with possible less severe disease at baseline, but this is still a very possible scenario that should be considered in the study schematic.
- ‘PK-MIC sub study’ – kindly spell out PK-MIC at first mention. Although we believe it refers to ‘pharmacokinetic’.

 

***Statistical analysis***

 The statistical plan is well described and very appropriate methods to assess the comparisons of the primary end points between the trial arms. Detailed sample size calculations also given.

**Discussion:**

 Very well written

**Conclusion:**

 This protocol by Chen clearly describes a well design randomized trial of well-refined severity stratification criteria that could potentially identify low-risk pulmonary TB patients who are cured within 4 months of standard TB therapy. Overall, this is a well-written and justifiable clinical trial protocol and we will recommend that it be accepted with minor corrections.

We have read this submission. We believe that we have an appropriate level of expertise to confirm that it is of an acceptable scientific standard.

# Response

**Published:** 2017-12-05

**Authors:** Ray Chen

We thank the reviewers for their comments. We have responded point by point below.

**Specific comments**

**Introduction:**

- The protocol presents a good background to the proposed study with a review of previous studies that have attempted to shorten duration of TB treatment and the outcome. Background is well written, hypothesis is clear and credible and the authors have provided a clear justification for the RCT. However, we will suggest a revision of the hypothesis to state clearly that the focus of the study is specifically ‘pulmonary tuberculosis’.

***We will make this clear in subsequent publications.***

**Methods:**

The design of the RCT is clear and appropriate for the study aims and objectives in general. Particularly important is the very clear and unambiguous definition of criteria for early treatment completion. Having stated this, there are a number of minor methodological issues that need to be stated more clearly in the protocol:

- What was the sampling strategy for eligible participants at the study sites?

***In RSA, staff from the local TB clinics are aware of the study inclusion/exclusion criteria and refer newly diagnosed pulmonary TB patients who appear to fit the criteria to study staff for informed consent and screening. In China, local TB clinic staff are our study staff and evaluate newly diagnosed TB patients for study eligibility. Those who appear eligible are asked to sign study informed consent for formal study screening.***

- There should be some statement about the qualification or specialization of the PET/CT readers to specify or indicate their minimum qualification and experience.

***All PET/CT scan readers are physicians trained to read scans using the standard operating procedure in Supplementary File 5. The results of all reads from the 15 practice scans at baseline and week 4 (30 scans total) must be within 2 standard deviations of the results from all other readers before a particular reader is qualified to read scans for the study.***

- Will the PET readers be blinded to the other patient characteristics including the clinical and laboratory results or not?

***Although the readers are not specifically blinded, they are generally not located at the enrolling clinic site so have no access to any participant characteristics. The exception is if the reader is located at the clinic site that enrolled that particular participant. The reader randomization process precludes 2 readers from a single institution being assigned to the same participant so this should mitigate any potential issues of bias.***

- Will the third reader who is to resolve any discrepant PET/CT scan be a more senior expert or this is to be assumed?

***The 3 reader is drawn from a pool of three of the most experienced readers.***

- There is no explicit mention in the protocol of what happens to patients who pass the early treatment completion criteria at baseline and at 4 weeks follow up, but found smear/culture and/or Xpert positive at 8 weeks or at 12 weeks follow-up (smear/culture only). One can assume that the likelihood of this happening might be low especially among the subjects with possible less severe disease at baseline, but this is still a very possible scenario that should be considered in the study schematic.

***We address the scenario in the clinical MOP sec 7.1 and 7.2 as it relates to week 16 but the same principles apply at earlier time points. We do not respond to smear results, whether positive or negative, as positive smears may persist beyond culture conversion. The worst-case scenario would be a participant who at week 16 had no positive sputum cultures (had already converted to negative), met study randomization criteria, and was randomized to stop treatment at week 16. Subsequently, the week 16 (or earlier) culture becomes positive. The participant would be called back for clinical evaluation and to collect another sputum sample for confirmation. If the participant is clinically asymptomatic, no further action is taken pending sputum culture positive confirmation. If the participant has symptoms concerning for recurrent TB, the treating physician has discretion to restart TB treatment for participant safety, with strong encouragement to collect multiple sputum samples before treatment is re-initiated. A 2 positive sputum culture is required to confirm study endpoint as recurrent TB.***

- ‘PK-MIC sub study’ – kindly spell out PK-MIC at first mention. Although we believe it refers to ‘pharmacokinetic’.

**Yes, PK is pharmacokinetic. MIC is minimum inhibitory concentration.**
